# Malleable Machines in Transcription Regulation: The Mediator Complex

**DOI:** 10.1371/journal.pcbi.1000243

**Published:** 2008-12-19

**Authors:** Ágnes Tóth-Petróczy, Christopher J. Oldfield, István Simon, Yuichiro Takagi, A. Keith Dunker, Vladimir N. Uversky, Monika Fuxreiter

**Affiliations:** 1 Institute of Enzymology, Biological Research Center, Hungarian Academy of Sciences, Budapest, Hungary; 2 Center for Computational Biology and Bioinformatics, Department of Biochemistry and Molecular Biology, Indiana University School of Medicine, Indianapolis, Indiana, United States of America; 3 Institute for Intrinsically Disordered Protein Research, Indiana University School of Medicine, Indianapolis, Indiana, United States of America; 4 Institute for Biological Instrumentation, Russian Academy of Sciences, Pushchino, Moscow Region, Russia; University of California San Francisco, United States of America

## Abstract

The Mediator complex provides an interface between gene-specific regulatory proteins and the general transcription machinery including RNA polymerase II (RNAP II). The complex has a modular architecture (Head, Middle, and Tail) and cryoelectron microscopy analysis suggested that it undergoes dramatic conformational changes upon interactions with activators and RNAP II. These rearrangements have been proposed to play a role in the assembly of the preinitiation complex and also to contribute to the regulatory mechanism of Mediator. In analogy to many regulatory and transcriptional proteins, we reasoned that Mediator might also utilize intrinsically disordered regions (IDRs) to facilitate structural transitions and transmit transcriptional signals. Indeed, a high prevalence of IDRs was found in various subunits of Mediator from both *Saccharomyces cerevisiae* and *Homo sapiens*, especially in the Tail and the Middle modules. The level of disorder increases from yeast to man, although in both organisms it significantly exceeds that of multiprotein complexes of a similar size. IDRs can contribute to Mediator's function in three different ways: they can individually serve as target sites for multiple partners having distinctive structures; they can act as malleable linkers connecting globular domains that impart modular functionality on the complex; and they can also facilitate assembly and disassembly of complexes in response to regulatory signals. Short segments of IDRs, termed molecular recognition features (MoRFs) distinguished by a high protein–protein interaction propensity, were identified in 16 and 19 subunits of the yeast and human Mediator, respectively. In *Saccharomyces cerevisiae*, the functional roles of 11 MoRFs have been experimentally verified, and those in the Med8/Med18/Med20 and Med7/Med21 complexes were structurally confirmed. Although the *Saccharomyces cerevisiae* and *Homo sapiens* Mediator sequences are only weakly conserved, the arrangements of the disordered regions and their embedded interaction sites are quite similar in the two organisms. All of these data suggest an integral role for intrinsic disorder in Mediator's function.

## Author Summary

Intrinsically disordered proteins/regions do not adopt well-defined three dimensional structures; instead, they function as conformational ensembles. They are distinguished in molecular recognition and involved in various regulatory processes. Several components in the transcription machinery–for example, the transactivator domains of transcription factors–are disordered. Mediator, which is a large complex that transduces regulatory information from activators/repressors to the core apparatus, was found to contain a preponderance of intrinsically disordered regions in its various subunits. Such disordered regions are commonly involved in conformational changes coupled to functional transitions, in protein–protein interactions, or in posttranslational modifications. Several such predicted recognition sites were in good agreement with experimental data. Intrinsically disordered regions illuminate a novel aspect of Mediator's regulation and could explain its versatility and specificity in handling transcriptional signals. Their integral role in Mediator function is further underscored by the conserved arrangements of ordered/disordered segments and of the embedded interaction sites.

## Introduction

**Figure 1:**
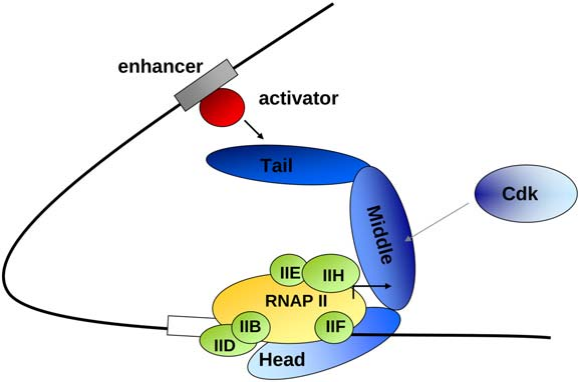
Mediator transmits regulatory signals from gene-specific activator proteins to the general transcription machinery, including RNA polymerase II (RNAP II, yellow), and general transcription factors (IIB, IID, IIE, IIF, IIH, light green). The Tail interacts with a variety of activators/repressors and the regulatory signals are transferred via the Middle module to the Head that physically contacts RNAP II. The Middle also receives signals from the CDK module that dissociates prior to transcription. The shades of the blue colors correlate to the level of disorder in the different modules in *Saccharomyces cerevisiae* as computed in the present work.

The Mediator complex is a gigantic (1 MDa) multi-protein complex that plays a number of essential roles in eukaryotic gene regulation [1]. It functions as a co-activator, a co-repressor as well as a general transcription factor by transmitting information from the regulatory factors bound at enhancers to the RNAP II transcription machinery [1],[2]. Mediator is recruited by promoter- and/or enhancer-bound activators [3] followed by association of general transcription factors and RNAP II with the promoter *in vivo* [4],[5] (Figure 1). Mediator dissociates from RNAP II after initiation, and remains attached to the promoter [6],[7] providing a pre-formed scaffold for the reinitiation [8].

Interactions with RNAP II and regulatory proteins induce dramatic conformational changes in Mediator [9],[10]. Activator induced specific rearrangements in Mediator expose cryptic RNAP II binding site and modulate the assembly of the pre-initiation complex (PIC) [11],[12]. This suggests that activators/repressors regulate transcription by altering the structure of the RNAP II holoenzyme. These conformational changes were thus proposed to underlie the regulatory mechanism of Mediator [13].

Mediator consists of 20–30 subunits that are organized in a modular fashion, with Head, Middle, and Tail regions [14] (Figure 1). The Tail can serve as the main target for activators/repressors [15]. The Med9 submodule of the Middle may connect the regulatory signals to the Head [16], which could in turn interact directly with RNAP-TFIIF for pre-initiation complex formation [17]. The Middle also receives repression signals from the CDK module, which dissociates prior to transcription [18]. The functions of the individual subunits however, are rather obscure apart from the reported kinase activity of the Cdk8 [19] and the histone acetyltransferase activity of the Med5 [20], which are non-essential for Mediator's function. Mediator protein sequences are highly variable with the exception of a few subunits [21]. The majority of the subunits have no apparent domains, not even the expected domains for chromatin modification such as chromo [22] or bromo domains [23] (Y.T. unpublished data). Nevertheless, based on cryo-electron microscopy, the overall structural organisation of several eukaryotic Mediator complexes is similar [24].

The low sequence conservation of Mediator proteins and the absence of known globular domains suggest the presence of disordered regions in Mediator. Such disordered regions might be responsible for similar structural characteristics in different organisms observed in EM studies [24] despite the lack of sequence conservation. IDRs can contribute to Mediator's function in three different ways: they can provide flexible target sites that can adapt to different partners with variable architectures; they can act as malleable linkers connecting globular domains that impart modular functionality on the complex; and they can also facilitate assembly and disassembly of complexes in response to regulatory signals.

To understand whether IDRs play a role in transcription regulation of the Mediator, 340 sequences of 30 subunits were collected (Table S1) and their tendencies for intrinsic disorder were predicted using bioinformatics approaches [25],[26]. Out of the 27 eukaryotic organisms *Saccharomyces cerevisiae* and *Homo sapiens* sequences were analyzed in detail and the results were corroborated using all available sequences (shown in the Supporting Information, Figures S1, S2, S3, S4, S5 and S6). The estimated level of disorder increases from yeast to man and in both organisms the propensity of disordered regions substantially exceeds that of signaling proteins and also that of multi-protein complexes of similar size. Subunits that interact with activators/repressors or function in regulatory signal transfer, located mostly in the Tail and Middle modules, are most abundant in IDRs. Overall, 43 sites for protein-protein interactions were predicted in 16 subunits in *Saccharomyces cerevisiae* and 79 sites in 19 subunits in *Homo sapiens* Mediator. In yeast, 11 of the predicted molecular recognition features (MoRFs) overlap with experimentally detected binding sites or post-translational modification sites, out of which those in Med7/Med21 [27] and Med8/Med18/Med20 [28] complexes have been structurally confirmed. The arrangement of ordered/disordered regions and location of disordered interaction sites are similar in *Saccharomyces cerevisiae* and *Homo sapiens*, although sequences of IDRs are only weakly conserved. All these results suggest that Mediator functions as a malleable machine in transcription regulation with an integral role for intrinsically disordered regions for the gene-specific regulatory functions.

## Results

### Overall Disorder of Mediator Proteins

**Figure 2:**
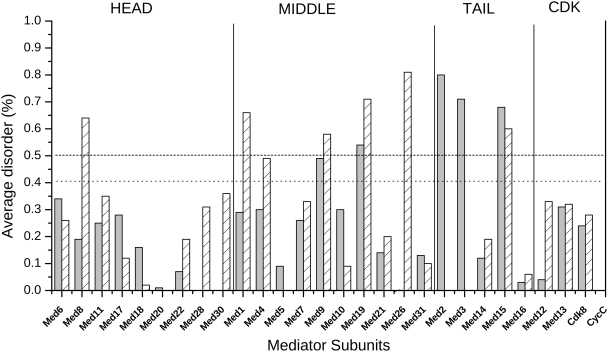
Average disorder of the available Mediator subunits in *Saccharomyces cerevisiae* (grey) and in *Homo sapiens* (crosshatched) as computed by the IUPred algorithm [26]. 0.5 (dashed line) is the threshold for disordered state and 0.4 (dotted line) is the average disorder of all disordered segments in the DisProt database [29]. Subunits belonging to the different modules (Head, Middle, Tail, Cdk) are separated by vertical lines.

Preference of Mediator proteins for intrinsic disorder was assessed by two independent bioinformatics approaches: PONDR-VSL1 that is a support vector machine algorithm [25] and IUPred that utilizes statistical inter-residue potentials [26]. Disorder predictions for Mediator proteins were carried out by both techniques at the amino acid level using sequences of individual proteins and the disorder scores were averaged over the entire sequence. As the two prediction methods provided consensus results, in the following only those obtained by the IUPred algorithm will be detailed. A preponderance of intrinsic disorder (average disorder above the 0.5 threshold value) was found in 4 and 6 out of 25 subunits in *Saccharomyces cerevisiae* and *Homo sapiens,* respectively (Figure 2). In addition, Med9 (in yeast) and Med4 (in man) have a level of disorder that is comparable to the disordered proteins assembled in the DisProt database [29]. These proteins likely lack a well-defined tertiary structure in the free form, but can partly or fully fold upon interacting with their partners [30]. The inherent flexibility of these subunits however, can contribute to structural organisation and molecular interactions of the complex. Overall, the levels of disorder (as averaged over all subunits) are higher in man than in yeast, suggesting an increase in the propensity or length of disordered regions. In *Saccharomyces cerevisiae* the Tail is most enriched in subunits with preference for intrinsic disorder (Med2, Med3, Med15), while in *Homo sapiens* the Middle module appears to be most abundant in malleable proteins (Med1, Med9, Med19, Med26). In the Head only Med8 is predicted to be disordered in *Homo sapiens*. Disorder scores averaged over sequences from all available organisms also indicate large variations in some subunits (please note, that in this case the number of sequences/subunits differ; Figure S1). This might implicate functional changes of various Mediator proteins during evolution.

**Figure 3:**
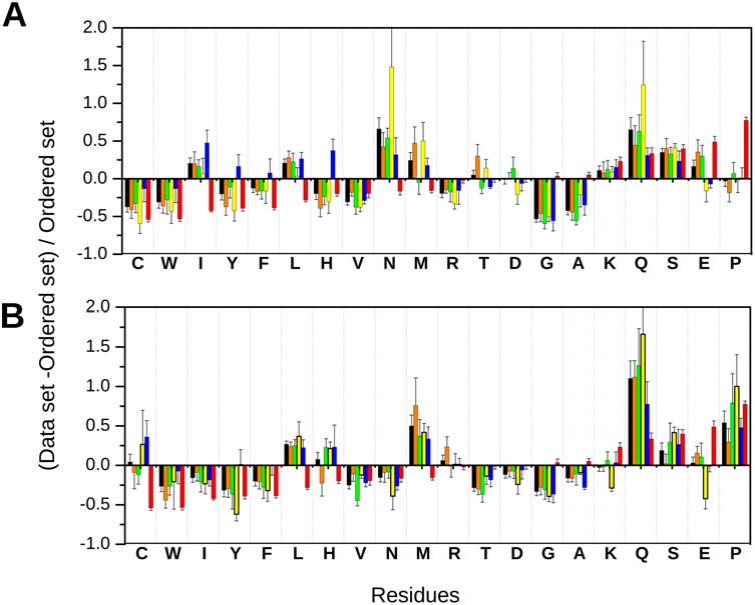
Amino acid compositions, relative to the set of globular proteins, of the Mediator (black), and its modules, Head (orange), Middle (green) and Tail (yellow) CDK (blue) in *Saccharomyces cerevisiae* (A) and in *Homo sapiens* (B). Compositional profiling of intrinsically disordered proteins from the DisProt database is shown for comparison (red). The arrangement of the amino acids is by peak height for the set of disordered proteins from DisProt [29]. Confidence intervals were estimated using per-protein bootstrapping with 1,000 iterations.

The amino acid compositions of Mediator proteins in *Saccharomyces cerevisiae* and *Homo sapiens* are also incompatible with a folded structure [31] (Figure 3), although they exhibit some variations. As compared to globular proteins, yeast and human Mediator proteins are depleted in hydrophobic (I, L, V), aromatic (W, Y, F) and C residues (designated as order-promoting); and enriched in polar (Q, N, T, S), charged (E, D) and structure-breaking (P) residues (designated as disorder-promoting). Such a composition resembles the general characteristics of intrinsically disordered proteins [32]. Various subunits, like the Med4 and Med15 are abundant in potential post-translational modification sites (S and T) that are preferably embedded in disordered regions [33]. Generally disordered polyQ and polyN regions frequently appear in various subunits, such as Med1, Med9, Med10, Med12 and Cdk8 (Figure S2). The Q-rich region in Med15 in *Saccharomyces cerevisiae* for example is involved in glucocorticoid receptor transactivity [34]. The propensity of Q-rich regions also increases from yeast to man. Repeat expansion may contribute to rapid evolutionary changes of Mediator proteins and may have created linkers between globular segments [35].

### Disordered Regions in Mediator Subunits

**Figure 4:**
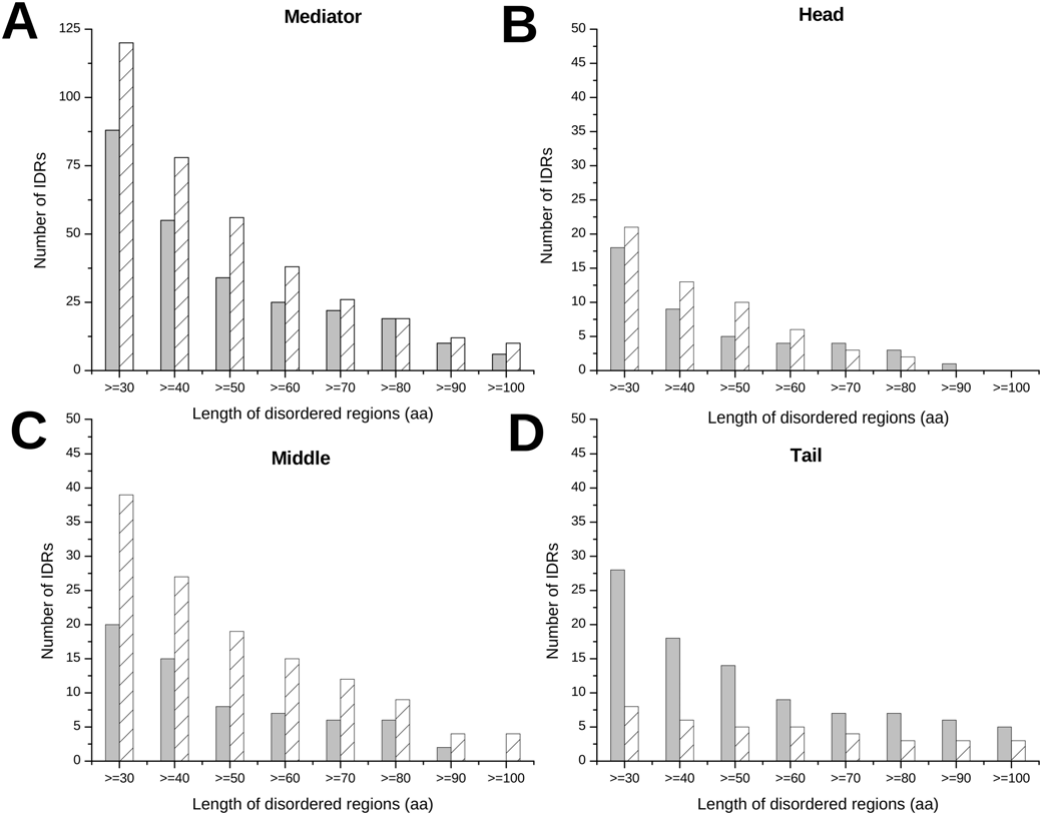
Abundance of IDRs in the Mediator complex and its modules. The number of disordered segments of given length in *Saccharomyces cerevisiae* (grey) and in *Homo sapiens* (crosshatched) as computed by the IUPred algorithm [26] is shown in the Mediator complex (A), in the Head (B), Middle (C) and Tail (D) modules.

**Figure 5:**
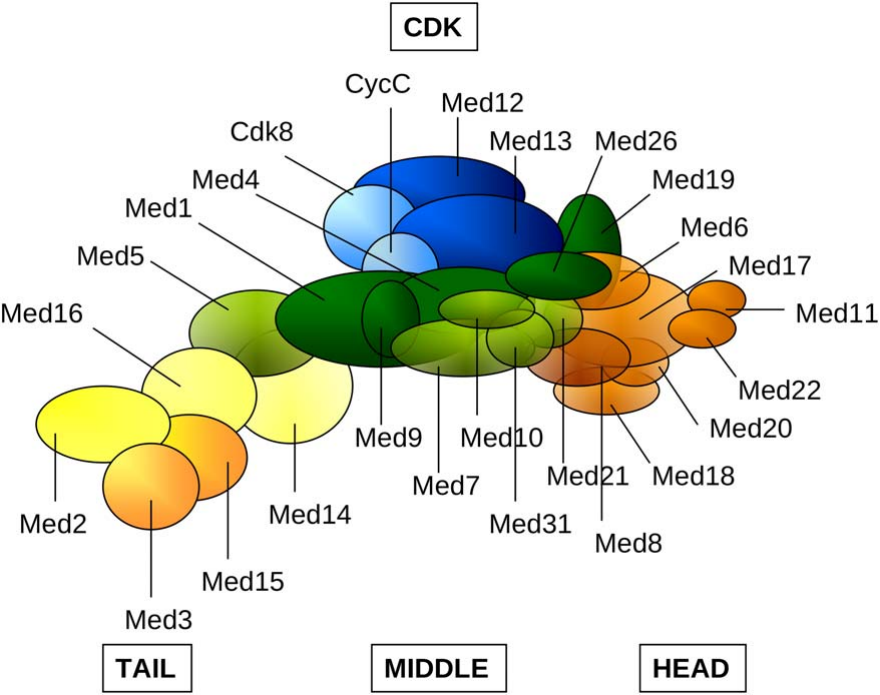
Schematic representation of the Mediator complex: Head (orange), Middle (green), Tail (yellow), CDK (blue). Subunits with higher than 50% average overall disorder (Med2, Med3 in Tail; Med9, Med19, Med26 in Middle and Med8 in Head) or subunits containing intrinsically disordered regions longer than 100 residues (Med12, Med13 of the CDK, Med1, Med9, Med26 of the Middle and Med15 of the Tail) in either *Saccharomyces cerevisiae* or in *Homo sapiens* are displayed by darker colors. Med19 and Med26 was assigned to the Middle module according to reference [80].

Intrinsically disordered regions of any length have been observed to be involved in biological functions, but those of 30 residues or longer have been especially well studied [36]. The function of these regions are diverse but are frequently related to molecular recognition [37]. IDRs are usually exploited for regulatory purposes as 66±5% of cell-signaling proteins [38], and 90% of transcription factors were predicted to contain IDRs (longer than 30 aa) [39],[40]. In *Saccharomyces cerevisiae* 80% of Mediator subunits have predicted IDRs equal to or longer than 30 residues, and 24% have IDRs above 100 residues in length [25] (Figure S3). In *Homo sapiens*, IDRs longer than 30 and 100 residues appear in 75% and 32% of Mediator proteins, respectively (Figure S3). This suggests that the length of IDRs increased from yeast to man. The number of disordered segments is also higher in the human complex than in the yeast complex (Figure 4). This is mostly due to the discrepancy in the number of IDRs in the Middle. This module is the most abundant in disordered regions in *Homo sapiens*. In the Head the propensity of IDRs is also slightly higher (below 70 residues in length) in man than in yeast. In *Saccharomyces cerevisiae*, disordered regions are preferably located in the Tail, some exceeding 100 residues in length. Along these lines, the longest IDRs in yeast are found in Med2 (334), Med3 (256), Med15 (263) of the Tail, whereas in human Mediator, Med1 (645), Med9 (241), Med26 (261) of the Middle are equipped with the longest IDRs (Figure 5 and Table S2). Med13 of the CDK appears to have a long IDR in both organisms: 226 and 162 in yeast and human, respectively.

Large multi-protein complexes generally take advantage of the plasticity of their components; i.e., the population of intrinsically disordered segments increases with complex size [41]. Multi-protein complexes of 11–100 proteins fulfilling various functions, have IDR propensity with median value of 12%, which estimates the percentage of disorder required to assemble a complex of a given size. The percentage of amino acids in IDRs is 32% and 33% in yeast and human Mediator, respectively (Figure S4), and these values considerably exceed those obtained for other complexes of similar size. One possibility is that the Mediator IDRs perform additional (eg., regulatory) tasks besides the self-assembly of the complex. Indeed, the level of disorder in Mediator is even higher than in signaling proteins (Figure S3).

### Molecular Recognition Features (MoRFs) in Mediator Proteins

Molecular recognition by IDRs is achieved by short, distinguishable segments, such as preformed elements [42], molecular recognition features [43], primary contact sites [44] and linear motifs [45],[46]. Preformed elements [42] and molecular recognition features [43] are predisposed to fold upon binding, and this reduces the entropy penalty of the recognition process. Primary contact sites [44] or linear motifs [45] are usually short, exposed segments that facilitate formation of highly specific interactions. In general all these recognition sites have higher local hydrophobicity than their environment and often exhibit transient secondary structure [46].

In *Saccharomyces cerevisiae* and *Homo sapiens* Mediators, we focused on those recognition sites that are biased for an α-helical conformation, termed α-MoRFs. These segments fold onto an α-helix in the bound form and can be predicted from the irregularities in computed disorder patterns using a neural network algorithm with 0.87±0.08 accuracy [47]. A prototypical example of an α-MoRF is the short α-helical segment in the disordered transactivator domain of p53 that mediates binding to Mdm2 [48],[49]. Multiple, tandem binding sites can be found in the BRCA1 protein that serve a scaffold function [50]. In yeast, predictions indicate the presence of 43 α-MoRFs in total, distributed over 16 subunits (Table 1). Some subunits have multiple α-MoRF regions, with Med15 of the Tail (11 α-MoRFs) and Med13 of the CDK module (6 α-MoRFs) in yeast having the largest numbers of these regions. In accord with the increased level of disorder, 79 interaction sites were identified in 19 subunits in *Homo sapiens* (Table S2). Most interaction sites were located in Med3 of the Tail (18 α-MoRFs) and Med1 of the Middle (14 α-MoRFs) and Med13 of the CDK (8 α-MoRFs).

**Table 1:** α-Helical molecular recognition features (MoRFs) predicted in *Saccharomyces cerevisiae*.

Mediator Subunit	MoRF_start	MoRF_end	Reference
**MED1**	373	390	
	459	476	
**MED2**	95	112	
	153	170	
	403	420	
**MED3**	333	350	[51]
	380	397	
**MED4**	235	252	[55]
**MED5**	1076	1093	
**MED6**	276	293	
**MED7**	21	38	
	195	212	[27], C
**MED8**	193	210	[28],[52]
**MED9**	125	142	C
**MED10**	140	157	C
**MED11**	1	18	
	99	116	C
**MED13**	376	393	
	439	456	[54]
	536	553	[54]
	614	631	[54]
	716	733	
	783	800	
**MED14**	1	18	
**MED15**	122	139	[34]
	212	229	[34], C
	285	302	
	319	336	[53]
	350	367	
	389	406	
	508	525	
	723	740	
	839	856	
	1022	1039	
	1061	1078	
**MED17**	12	29	[2]
	77	94	
	106	123	
	197	214	C
	600	617	
**Cdk8**	1	18	
	132	149	
	517	534	

References indicate experimentally confirmed protein binding sites. MoRFs marked by C correspond to α-helical regions that were found to be highly conserved from yeast to man [21].

The predicted α-MoRFs in *Saccharomyces cerevisiae*, which may serve as potential target sites for protein-protein interactions or for post-translational modifications, were compared to experimentally verified binding sites reported in literature or assembled in protein-protein interaction databases. So far 11 out of the of the 43 predicted α-MoRFs in yeast have been experimentally corroborated (Table 1). For example, the α-MoRF encompassing residues 333–350 of Med3 likely corresponds to the Gcn4 target site [51], while the α-MoRF 195–212 predicted in Med7 serves as a contact site with Med10 [52]. Specific mutation sites in Med17 at the interaction sites with the Middle and Tail modules [2] (and Y.T. unpublished data) also coincide with the identified MoRFs. The region 116–255 of Med15 that interacts with Gal4 [53] contains two predicted α-MoRFs. The 261–351 segment of Med15 that is responsible for transcriptional activation of glucocorticoid receptor also contains one α-MoRF that matches the observed interaction site [34]. The region 396–655 of Med13 contains 3 predicted α-MoRFs and has been observed to contact various partners: Caf1, Crc4, Not2 as well as Cdk8 [54]. The predicted phosphorylation site at T237 in Med4, which might play role in enhancement of RNAP CTD phosphorylation by TFIIH [55], matches the experimentally determined position.

**Figure 6:**
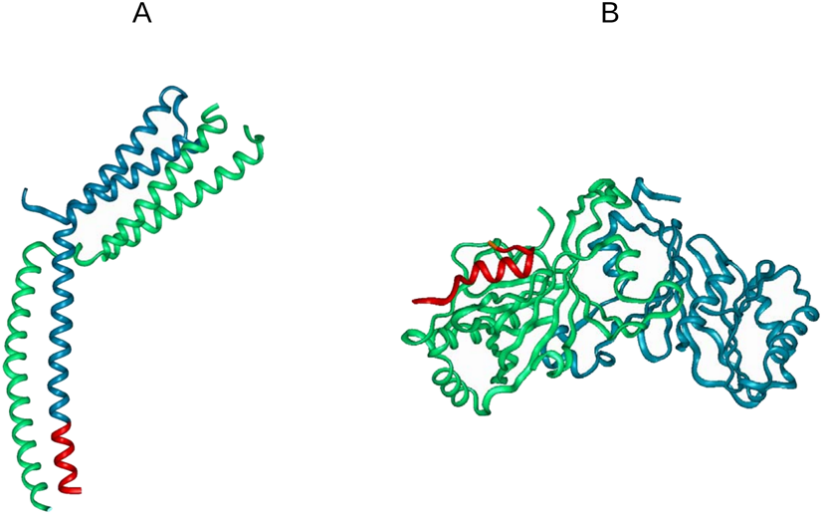
Location of α-MoRFs predicted by the PONDR VL-XT algorithm in Med7 and Med8 subunits of *Saccharomyces cerevisiae* in (A) Med7/Med21 (1yke) [27] and (B) Med8/Med18/Med20 (2hzs) [28] complexes. The recognition motifs in Med7 (195–212) and Med8 (193–210) that are biased for an α-helical conformation in the bound state are shown by red.

In the case of Med7 and Med8, the available crystal structures of the Med7/Med21 [27] and the Med8/Med18/Med20 [28] complexes can be used for structural validation of α-MoRFs (Figure 6). The Med7/Med21 heterodimer serves as a hinge that was proposed to be responsible for large scale changes in the Mediator's structure [27]. In the complex three α -helices of Med7 were observed that constitute a coiled-coil. The predicted α-MoRF 195–212 is located at the C terminal end of α3 that makes contacts with α3 helical region of Med21. In accord with its predicted increase in flexibility, this segment has elevated B-factors in the bound form. Of course the elevated B-factor values might simply stem from its terminal location. The C-terminal fragment encompassing residues 193–210 of Med8, which was predicted as an α-MoRF, adopts an α-helical conformation in the Med8/Med18/Med20 complex [28]. While 27 residues of Med8 were used for crystallization, only 16 were observed in the complex, indicating the presistance of disorder even in bound form. This segment is embedded in a larger disordered region, encompassing the linker between the C and N terminal of Med8. This linker exhibits enhanced sensitivity to proteolytic digestion in the free protein corroborating its disordered state. This region was shown to be essential for transcription in *vivo* by harboring elongin B and C [56].

An independent argument for the functional importance of the predicted α-MoRFs in 6 subunits (Med7, Med9, Med10, Med11, Med15, Med17, cf. Table 1) is underscored by their overlap with helical regions that have been proposed to be highly conserved from yeast to man [21].

### Conservation of Intrinsically Disordered Regions

**Figure 7:**
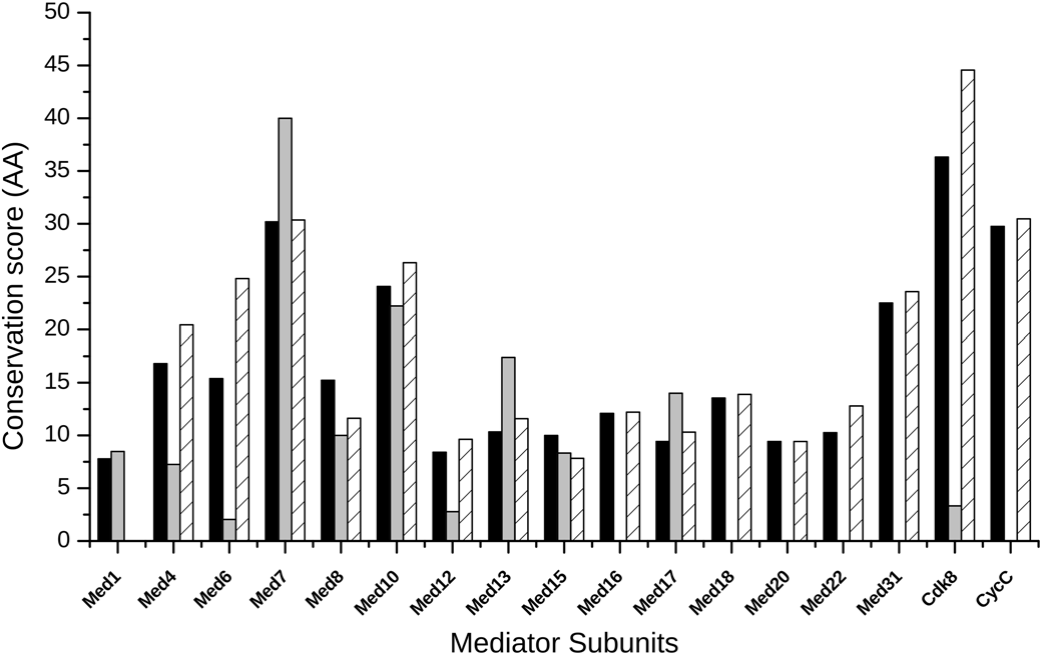
Amino acid conservation of ordered (crosshatched) and disordered (gray) regions in *Saccharomyces cerevisiae* and in *Homo sapiens*. Total amino acid conservation shown in black.

IDRs in homologous proteins often exhibit remote sequence relationships. The functioning of IDRs likely relies on their biased amino acid composition and their short motifs [43],[44],[46], the latter of which enables a rapid evolution of IDRs [57],[58]. Hence, the presence of IDRs might account for the weak sequence conservation of Mediator proteins despite their similar functions or architectures [14],[24]. As anticipated, a remarkable difference between the sequence conservation of disordered and ordered regions were also seen in *Saccharomyces cerevisiae* and *Homo sapiens* Mediators (Figure 7). This distinction can also be observed if Mediator subunits from all available organisms are aligned (Figure S5). In contrast to the sequence behaviors, the propensities of order and disorder promoting amino acids in IDRs were found to be highly conserved (Figure S5).

**Figure 8:**
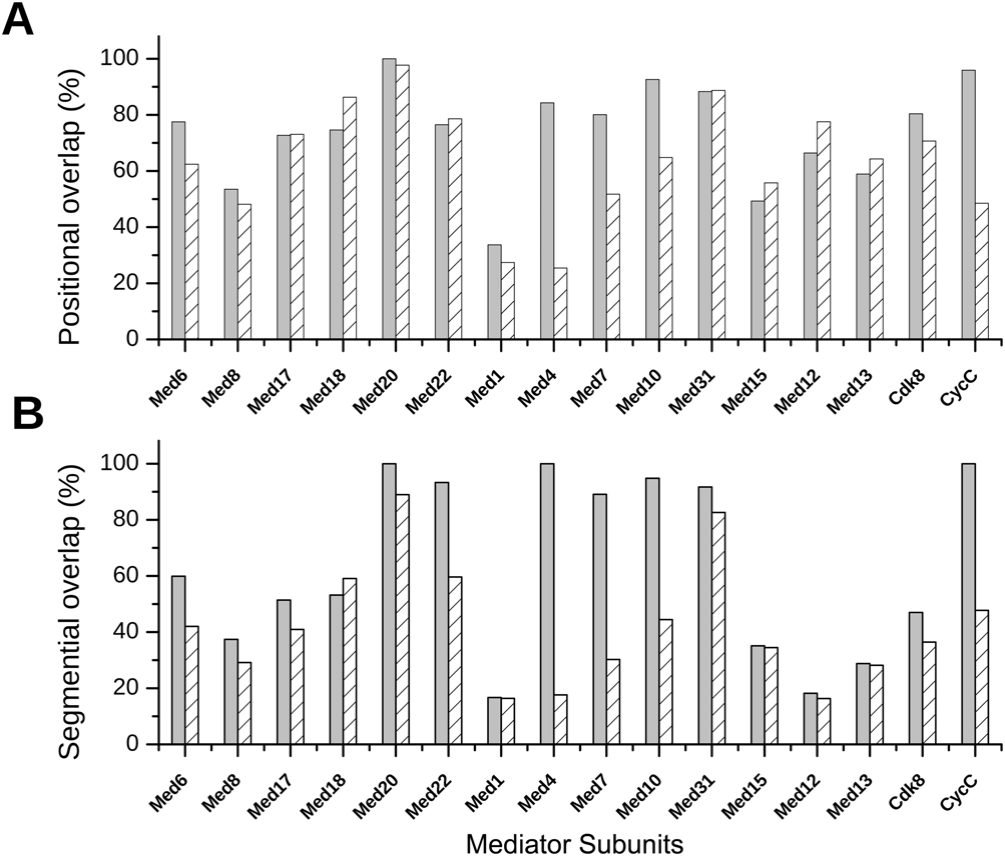
Conservation of disordered regions in *Saccharomyces cerevisiae* and in *Homo sapiens*. The arrangement of ordered/disordered segments is compared to each other using positional (A) segmental overlap (B) measures on the actual Mediator protein sequences in MED_ALSEQ dataset (grey) and on the corresponding randomized MED_ALRAN dataset (crosshatched).

Recently we introduced a method to assess the conservation of IDRs based on the arrangements of ordered and disordered segments, as predicted by the IUPred algorithm, in different sequences [59]. This can be evaluated at the level of residues, i.e., by computing the percentage of residues designated as ordered or disordered at the same position in sequence alignments. On the average 74.5% of residues are located in regions with the same character (disordered or ordered) in *Saccharomyces cerevisiae* and *Homo sapiens* (Figure S6). Alternatively, the overlap between ordered and disordered segments in different sequences can be measured by adopting the accuracy measures of secondary structure predictions [59],[60]. In this case the arrangement of ordered/disordered segments in different sequences is compared to each other in terms of the persistence of their location in different organisms. The overlap between the patterns of ordered/disordered regions in yeast and human Mediator is 73.2%. This value significantly exceeds the corresponding value determined from randomized sequences with the same amino acid composition (Figure 8). Thus it appears that, in contrast to the sequences themselves, the arrangements (patterns) of disordered regions are conserved in different organisms, providing a further support for their functional importance.

## Discussion

Transcriptional control requires an intimate interplay between the enhancer- and repressor-bound factors and the basal transcription machinery. In eukaryotic organisms large co-activators, such as the Mediator complex [1] or CBP/p300 [61] are responsible for transducing regulatory information to the core apparatus and link chromatin remodeling to m-RNA synthesis. The mechanism by which these large assemblies impart versatility and specificity on transcription regulation however, remains to be uncovered. It has been proposed that dramatic conformational changes that occur upon interactions with regulatory proteins [10]–[13] as well as with RNAP II [9] could serve as a basis of the Mediator's control mechanism [13]. Such large-scale structural rearrangements could be facilitated by highly flexible/malleable segments that can serve as molecular “hinges” [10]. Furthermore, based on the abundance of intrinsically disordered proteins in signaling [36], we reason that the signal transducer function of Mediator is also intertwined with IDRs. IDRs mediating specific, transient interactions were observed at various checkpoints of transcription [62], like in histone tails [63], transactivator domains of transcription factors [64] and the C-terminal domain of RNAP II [65].

In this study, bioinformatics approaches were employed to assess the preference of Mediator proteins for intrinsic disorder, focusing on the comparison of *Saccharomyces cerevisiae* and *Homo sapiens* Mediator complexes. Various subunits, located mostly in the Middle (Med1, Med9, Med19, Med26) in human and in the Tail (Med2, Med3, Med15) in yeast are predicted to be enriched in disordered regions (Figure 2 and Figure 4). As the level of disorder in these proteins is higher than that of proteins assembling into other complexes of similar size, IDRs are likely exploited for additional, regulatory functions besides facilitating the self-assembly of the complex. Along these lines, the propensity of disordered regions in both yeast and human Mediator exceed that in signaling proteins. Results obtained on all available Mediator sequences (340) presented in Supporting Information (Figures S1, S2, S3, S4, S5 and S6) also corroborate the results obtained on the two organisms emphasized here.

Because the predictions were performed on individual sequences, we cannot exclude the possibility that regions predicted to be intrinsically disordered adopt a well-folded structure upon interacting with other Mediator subunits or with regulatory proteins. Electron microscopy results however indicate the pliability of the complex at low ionic strength (Francisco Asturias, private communication) that argues against the complete loss of disordered state in the Mediator complex. An independent argument comes from the structure-function analysis of complexes of intrinsically disordered proteins. In many cases IDRs were found to remain disordered even bound to their partners and yet critically affect binding affinity or specificity [66]. In these ‘fuzzy’ complexes IDRs interact via short segments, while the embedding regions may remain structurally variable.

To probe if IDRs are utilized for macromolecular communication, sites of protein-protein interactions were predicted in disordered regions and are biased for an α-helical conformation. In total 43 α-MoRFs were identified in yeast Mediator, with 79 α-MoRFs in human Mediator. The roles of α-MoRFs as protein-protein interaction sites is also suggested by the overlap of the predicted and experimentally observed binding regions. For example, in *Saccharomyces cerevisiae* 11 α-MoRFs were predicted in Med15 of the Tail that is likely to be the main sensor for regulatory proteins, while 6 α-MoRFs in Med13 of CDK is embedded in a region that hosts various trancriptional proteins (Table 1). Overall, the functional importance of 11 predicted α-MoRFs either as interaction sites or post-translational modification sites have been experimentally confirmed in yeast. In the cases of the Med7/Med21 [27] and the Med8/Med18/Med20 [28] complexes, structural data corroborate the role of the predicted α-MoRFs as recognition sites that adopt an α-helical structure in the bound state. Although less experimental data are available for human Mediator, 5 α-MoRFs predicted in Med1 fall into regions interacting with various transcriptional proteins (Table S2). For example, the N-terminal 306 residues of Med1 is involved in the transactivator function of BRCA1 [67], while the 433–803 region (with 4 predicted α-MoRFs) hosts the nuclear receptor LXRb and KIF1a [68].

So how does intrinsic disorder contribute to the function of Mediator? IDRs represent an ensemble of conformations [69] that imparts extreme flexibility onto the complex. In response to regulatory signals IDRs can adopt different conformations [70] and thereby induce functional transitions. In this way they could contribute to the observed pleomorphism of Mediator. IDRs with multiple binding sites indicated by the MoRFs may provide a scaffold-like function and thereby can be important to organize the complex. IDRs can also serve as malleable linkers between globular domains and may underlie modular functionality of the Mediator complex that enable it to interpret different combinations of transcriptional inputs [71]. IDRs can also facilitate assembly/disassembly of large complexes [37], for example association of Mediator with TFIID triggers assembly of the PIC. IDRs can be involved in complex signaling events [72] due to their adaptability. The same IDR can accommodate different partners [73] that may exert different, even opposite outcomes on transcription [74]. For example, the disordered N-terminal region of Med3 can host both Gcn4 and Tup1 proteins [51], or the C-terminal 100 residues of Med19 are involved in both transcriptional activation and repression [75]. IDRs are also preferred environments for post-translational modification sites [33] that provide a further regulatory tool for the Mediator complex (cf. T237 in Med4 [55]).

The presence of disordered regions also highlight an evolutionary aspect of Mediator's function. We observe that the propensity of disordered regions as well as the number of embedded interaction sites increases from yeast to man. This not only argues for an integral role of IDRs in Mediator's function, but may explain why the human Mediator is capable of processing a significantly larger number of regulatory signals (eg. the number of transcription factors increase by one order of magnitude from yeast to man [76]). Even if IDRs are conserved, as it was demonstrated by their similar arrangements in *Saccharomyces cerevisiae* and *Homo sapiens* their sequences are tolerant to substantial changes as long as the amino acid composition is biased for disorder [58],[66]. Only sequences of short segments that serve as recognition sites need to be restrained, as seen in case of 6 α-MoRFs [21]. On the other hand it is very easy to turn on and off the functionalities carried by these short motifs [45].

In conclusion, we propose that conserved intrinsically disordered regions contribute to the gene-specific regulatory function of the Mediator. IDRs with weak sequence restraints can provide an evolutionarily economic solution for the Mediator to handle a steadily increasing amount of complex regulatory signals. These results argue for the functional conservation of the Mediator and may account for the evolution of its regulation complexity.

## Materials and Methods

### Databases

Mediator protein sequences were extracted from the UniProt and NCBI databases using a large number of Mediator subunit names. Overall 556 sequences were identified out of which the redundant ones above 90% identity were removed by the CD-hit program [77]. In addition, a PSI-BLAST [78] search was performed using the 196 sequences from 10 organisms in the reference [21]. All resulting sequences were assembled in the MED_ALSEQ database that contained 340 sequences of 30 Mediator subunits derived from 27 eukaryotic organisms (Table S1). The corresponding randomized sequences (50 times each) were collected in the MED_ALRAN database. As a nomenclature for the Mediator subunits we adopted the unified convention proposed in reference [79]. Med19 and Med26 was assigned to the Middle module according to the reference [80].

### Disorder Calculation

Intrinsic disorder preferences of sequences in the MED_ALSEQ and MED_ALRAN databases were predicted at amino acid level using the IUPred (http://iupred.enzim.hu) [26] and PONDR VSL1 [25] algorithms. Intrinsically disordered segments were defined as regions with more than 30 subsequent residues with predicted disorder above 0.5, allowing a maximum of 3 residue long ordered gaps. MoRFs were computed using the reported algorithm [47]. Likely phosphorylation sites were identified using the DisPhos program [33].

### Calculation of Amino Acid Composition

The fractional difference is calculated as (C_X_−C_ordered set_)/C_ordered set_, where C_X_ is the averaged content of a given amino acid in a protein set and C_ordered set_ is the corresponding averaged content in a set of ordered proteins from the PDB.

### Alignment Algorithms

Due to the presence of low-complexity regions, an iterative PSI-BLAST [78] based profile generation algorithm was performed to align full-length sequences of Mediator proteins [59]. Groups of homologous sequences were defined based on mutual sequence similarity (below the treshold of E = 10^−5^) between all members of the group. The final multiple alignment was generated by the CLUSTALW algorithm [81] using the BLAST profiles extracted from sequence groups. The performance of the alignment as compared to previous alignments [21],[27] are presented in Tables S3 and S4.

### Sequence Conservation

The sequence conservation of the Mediator proteins was evaluated comparing individual amino acid types (AAcons) using a simple Sum-of-Pairs (SP) score formula [82]. The score was 1 if identical residue was present in each positions of the alignment, otherwise it was 0 and these scores were averaged over the entire sequence.

### Overlap of Disordered Regions

Similarity between patterns of disordered and ordered regions was assessed using accuracy measures of secondary structure predictions [59],[60]. The overlap between ordered and disordered motifs (excluding gap positions) at residue level (Q) was characterized by the accuracy matrix defined as Q_2_ = 100 (M_OO_+M_DD_)/N, where M_OO_ and M_DD_ are the number of positions associated with the same motif type. Overlap between the segments were computed as
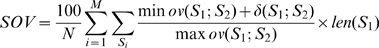
where S_1_ and S_2_ stand for segments in two distinct sequences, respectively, min*ov*(S_1_; S_2_) is the length of the overlap between S_1_ and S_2_, max*ov*(S_1_; S_2_) is the total extent of S_1_ and S_2_ in the given conformational state and len(S_1_) is the length of the segment in the reference sequence. δ(S_1_; S_2_) is the minimum of [(max*ov*(S_1_; S_2_)–min*ov*(S_1_; S_2_); min*ov*(S_1_; S_2_); int(len(S_1_)/2); int(len(S_2_)/2)]. The normalization factor N is given by the number of residues in conformational state *i* and the second summation runs over all M conformational states. Q and SOV values obtained for each possible pair within a given group of aligned sequences were averaged. The significance of the results was probed against the overlap values computed on the MED_ALRAN database.

## Supporting Information

Figure S1 Average disorder of Mediator subunits computed on sequences from all available organisms by PONDR VSL1 (grey) and IUPred (crosshatched). 0.5 (dashed line) is the threshold for disordered state and 0.4 (dotted line) is the average disorder of all disordered segments in the DisProt database [29]. Error bars represent standard deviations of organisms. Subunits belonging to the different modules (Head, Middle, Tail, Cdk) are separated by vertical lines.
(0.02 MB PDF)

Figure S2 Alignment of sequences of Mediator subunits from all available organisms (Table S1). Disordered regions are highlighted by yellow, alpha-MoRFs predicted in *Homo sapiens* and *Saccharomyces cerevisiae* are marked by orange. PolyQ, polyN and repeat regions (above 10 residues in length) are marked by boxes. Groups of similar amino acid residues are colored as R/K/H (cyan) A/S/T (green), I/L/V/M/C/F/Y/W (blue), G/P (magenta) and E/D/N/Q (red). Graphical representation was prepared by the ALSCRIPT program.
(1.76 MB PDF)

Figure S3 Abundance of IDRs in the Mediator complex and its modules in *Saccharomyces cerevisiae* (A) and in *Homo sapiens* (B). Percentages of proteins from the Mediator (black) and its different modules: Head (orange), Middle (green), Tail (yellow) with long disordered regions of given length. Corresponding data for signaling proteins (red) are shown for the comparison.
(0.02 MB PDF)

Figure S4 The ratio of the total length of all intrinsically disordered regions (IDRs, black) as determined by the IUPred algorithm and the longest unstructured segment (grey) relative to the full length of the protein in *Saccharomyces cerevisiae* (A) and in *Homo sapiens* (B) and averaged over all available organisms (C). IDRs were considered as a continuous stretches of more than 30 residues that are predicted to be disordered with a maximum gap length of 3 ordered residues. Error bars represent the standard error of the mean values. Vertical lines separate subunits belonging to different modules.
(0.02 MB PDF)

Figure S5 Amino acid conservation of Mediator subunits in all available organisms in ordered (gray) and disordered (crosshatched) regions (A). Propensities of order-promoting (grey) and disorder-promoting (crosshatched) amino acids in IDRs of homologous Mediator protein sequences (B). Small error bars indicate a high conservation of disorder/order promoting amino acid composition.
(0.02 MB PDF)

Figure S6 Conservation of intrinsically disordered regions (IDRs) as computed at amino acid (A) and segmental (B) level. Positional and segmental overlap obtained on the actual Mediator protein sequences (MED_ALSEQ, crosshatched) is compared to the overlap between IDRs in the corresponding randomized sequences (MED_ALRAN, grey). The IDRs are defined based on the scores by the IUPred algorithm.
(0.02 MB PDF)

Table S1 Sequences of Mediator subunits in the MED_ALSEQ database. Uniprot or NCBI codes are reported. Sequences, which were obtained as the Supplementary material of the reference [21] (and no corresponding sequences are found in Uniprot or NCBI by BLAST search), are marked by their reference number.
(0.06 MB XLS)

Table S2 α-Helical molecular recognition features (MoRFs) predicted in the Mediator complex in *Homo sapiens*
(0.10 MB DOC)

Table S3 Conservation scores computed on *Homo sapiens* and *Saccharomyces cerevisiae* sequences aligned by the reference [27] and also by the present iterative alignment scheme. Scores were obtained using groups of similar amino acid residues: R/K/H, A/S/T, I/L/V/M/C/F/Y/W, G/P and E/D/N/Q.
(0.03 MB DOC)

Table S4 Conservation scores computed on full sequences aligned by the reference [21] and the present iterative algorithm using the same sequences. AAcons was obtained using individual amino acid residues. For consistency, sequences only from those organisms were used that were found to be homologous by the present algorithm.
(0.04 MB DOC)
