# Supplementary figures and images for: Malleable Machines in Transcription Regulation: The Mediator Complex
Source: PLoS Comput Biol. 2008 Dec 19;4(12):e1000243. doi: 10.1371/journal.pcbi.1000243 (PMC2588115; doi:10.1371/journal.pcbi.1000243)

Figure S1

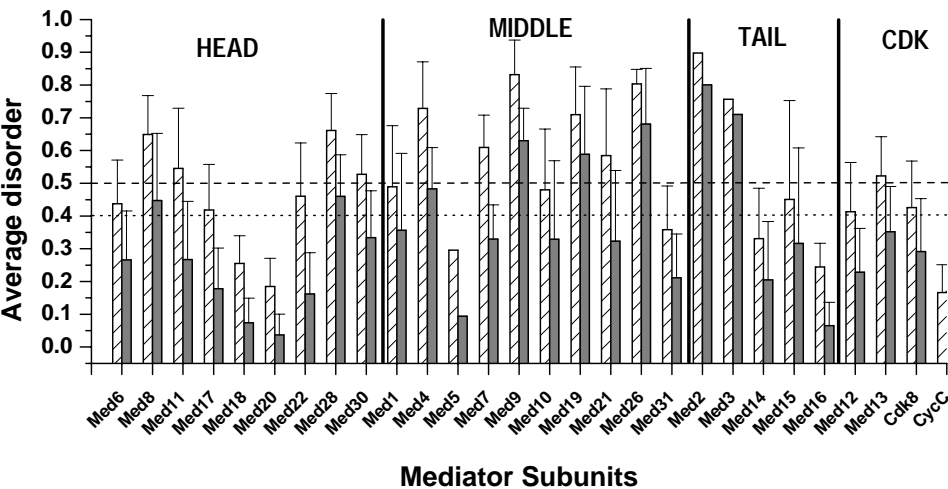

Supplement: Figure S1 — Average disorder of Mediator subunits computed on sequences from all available organisms by PONDR VSL1 (grey) and IUPred (crosshatched). 0.5 (dashed line) is the threshold for disordered state and 0.4 (dotted line) is the average disorder of all disordered segments in the DisProt database [29]. Error bars represent standard deviations of organisms. Subunits belonging to the different modules (Head, Middle, Tail, Cdk) are separated by vertical lines. (0.02 MB PDF) [file pcbi.1000243.s001.pdf]

Figure S3

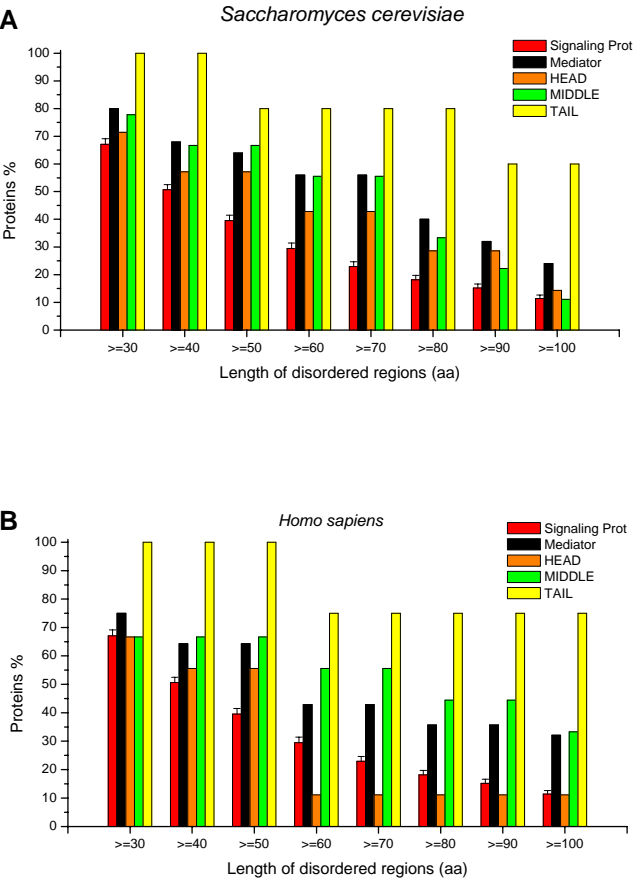

Supplement: Figure S3 — Abundance of IDRs in the Mediator complex and its modules in Saccharomyces cerevisiae (A) and in Homo sapiens (B). Percentages of proteins from the Mediator (black) and its different modules: Head (orange), Middle (green), Tail (yellow) with long disordered regions of given length. Corresponding data for signaling proteins (red) are shown for the comparison. (0.02 MB PDF) [file pcbi.1000243.s003.pdf]

Figure S4

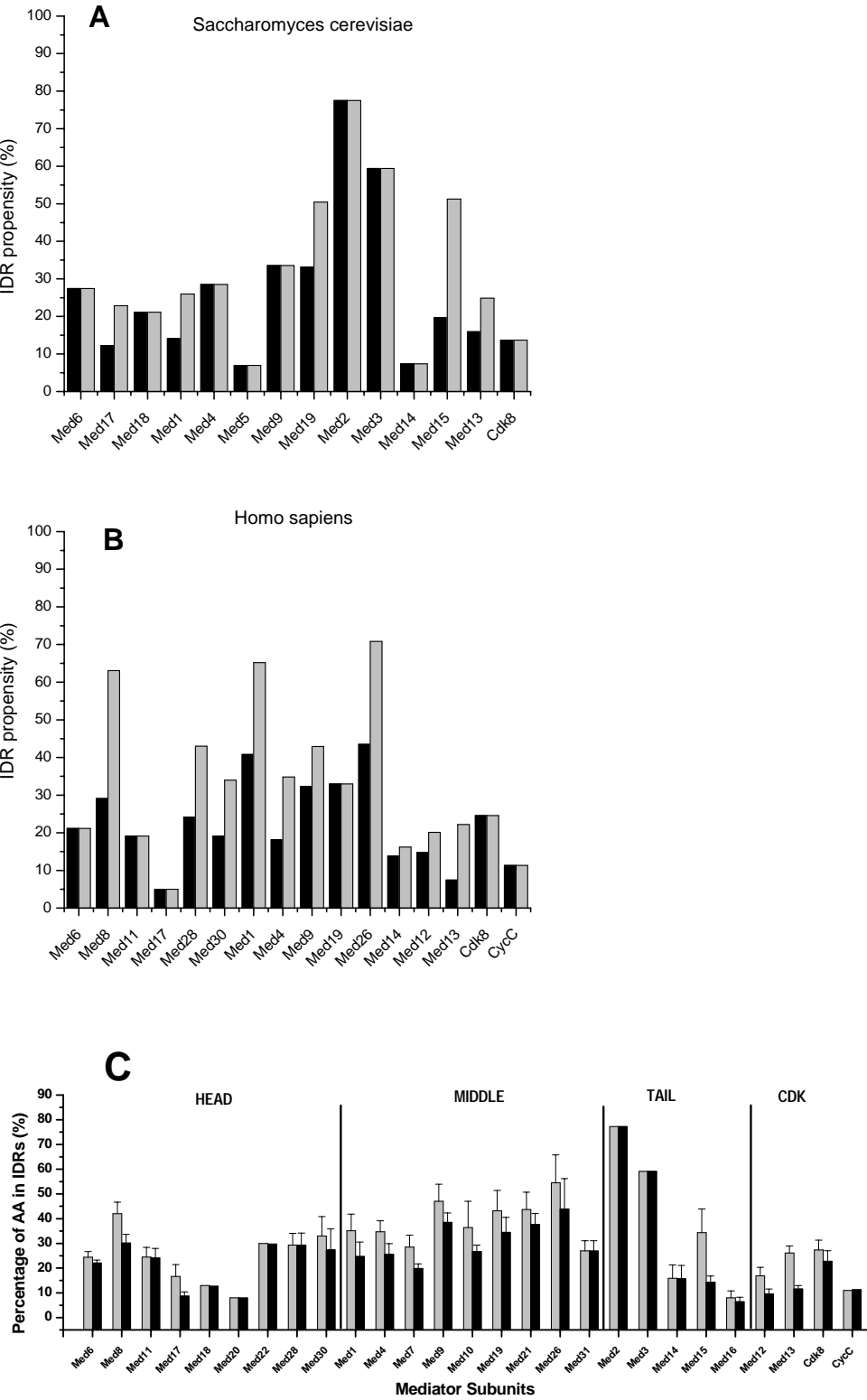

Supplement: Figure S4 — The ratio of the total length of all intrinsically disordered regions (IDRs, black) as determined by the IUPred algorithm and the longest unstructured segment (grey) relative to the full length of the protein in Saccharomyces cerevisiae (A) and in Homo sapiens (B) and averaged over all available organisms (C). IDRs were considered as a continuous stretches of more than 30 residues that are predicted to be disordered with a maximum gap length of 3 ordered residues. Error bars represent the standard error of the mean values. Vertical lines separate subunits belonging to different modules. (0.02 MB PDF) [file pcbi.1000243.s004.pdf]

Figure S5

A

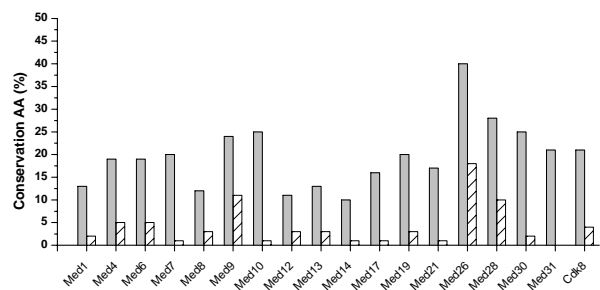

B

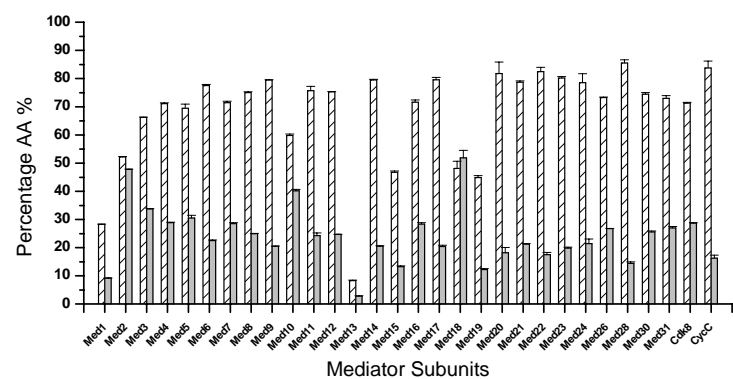

Supplement: Figure S5 — Amino acid conservation of Mediator subunits in all available organisms in ordered (gray) and disordered (crosshatched) regions (A). Propensities of order-promoting (grey) and disorder-promoting (crosshatched) amino acids in IDRs of homologous Mediator protein sequences (B). Small error bars indicate a high conservation of disorder/order promoting amino acid composition. (0.02 MB PDF) [file pcbi.1000243.s005.pdf]

Figure S6.

A

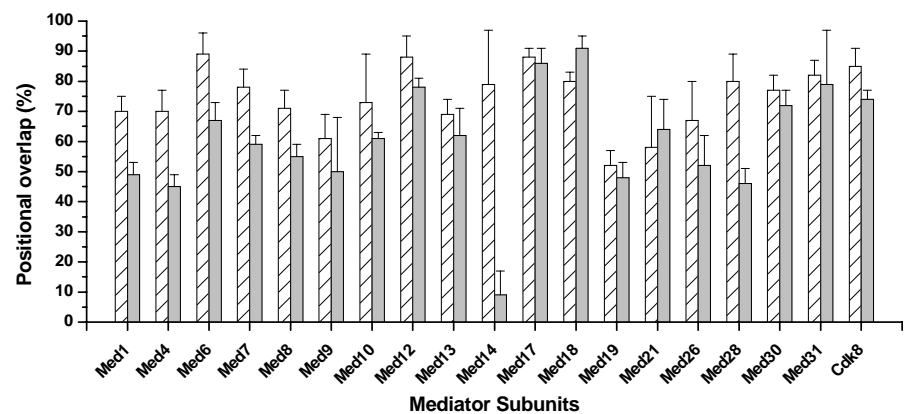

B

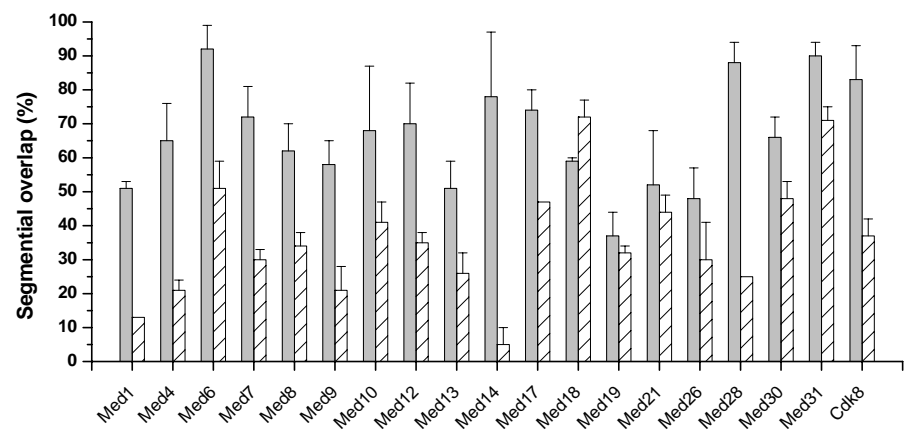

Supplement: Figure S6 — Conservation of intrinsically disordered regions (IDRs) as computed at amino acid (A) and segmental (B) level. Positional and segmental overlap obtained on the actual Mediator protein sequences (MED_ALSEQ, crosshatched) is compared to the overlap between IDRs in the corresponding randomized sequences (MED_ALRAN, grey). The IDRs are defined based on the scores by the IUPred algorithm. (0.02 MB PDF) [file pcbi.1000243.s006.pdf]
