# Supplementary material for: Malleable Machines in Transcription Regulation: The Mediator Complex
Source: PLoS Comput Biol. 2008 Dec 19;4(12):e1000243. doi: 10.1371/journal.pcbi.1000243 (PMC2588115; doi:10.1371/journal.pcbi.1000243)
Supplement: Figure S2 — Alignment of sequences of Mediator subunits from all available organisms (Table S1). Disordered regions are highlighted by yellow, alpha-MoRFs predicted in Homo sapiens and Saccharomyces cerevisiae are marked by orange. PolyQ, polyN and repeat regions (above 10 residues in length) are marked by boxes. Groups of similar amino acid residues are colored as R/K/H (cyan) A/S/T (green), I/L/V/M/C/F/Y/W (blue), G/P (magenta) and E/D/N/Q (red). Graphical representation was prepared by the ALSCRIPT program. (1.76 MB PDF) [file pcbi.1000243.s002.pdf]

Med1_1

```
    1        10        20        30        40        50        60
Ca  ------------------------------------------------------------
Af  ------------------------------------------------------------
Sp  ------------------------------------------------------------
Nc  ------------------------------------------------------------
Sc  ------------------------------------------------------------
Cg  ------------------------------------------------------------
Ce  MIIEERKNHELNLESIDRDLRMENVRQSAEKLGWEHFARSVRRNLLEKRQTLDARTRLDV

             70        80        90        100       110       120
Ca  ------------------------------------------------------------
Af  ------------------------------------------------------------
Sp  ------------------------------------------------------------
Nc  ------------------------------------------------------------
Sc  ------------------------------------------------------------
Cg  ------------------------------------------------------------
Ce  LGSLVFMKEHLPIDNDATIARKVQQLSEGLGEHCVFSTQNSGFSIRNPDVTIDIGVAEDQ

             130       140       150       160       170       180
Ca  ------------------------------------------------------------
Af  ------------------------------------------------------------
Sp  ------------------------------------------------------------
Nc  ------------------------------------------------------------
Sc  ------------------------------------------------------------
Cg  ------------------------------------------------------------
Ce  ISTCKIGYFGQPLTDAPGALALMKNGEFSKLRDSITAILSSLPKEITLSEKNACKEAFRA

             190       200       210       220       230       240
Ca  ------------------------------------------------------------
Af  --------------------------REHPNPPDFSPRPTGAHMARPIAHKSPSPRTPSA
Sp  ------------------------------------------------------------
Nc  -----------------------------------MTTPTPMKHTLSQQGSGRTPSQTQH
Sc  ------------------------------------------------------------
Cg  ------------------------------------------------------------
Ce  LDQLLIRDAADSSFTAINTGKYGFYAPRNELRSGRIYYVAEPVLIRLAEKEGKTKADASY

             250       260       270       280       290       300
Ca  ------------------------------------------------------------
Af  SGHGHGHQPSVSSHQYATLLAATTGIEDTVTFSSP--SALLALGGYGGISP------SPA
Sp  ------------------------------------------------------------
Nc  GAAATPPVSTPFSMAHAALSPHGSRLSPQAVKKSPAISAAALLNHP--TSTSAVSFDSPS
Sc  ------------------------------------------------------------
Cg  ------------------------------------------------------------
Ce  LDLLPYIEISFFKHDKPSKLPVFTQTGEWSTFNDANVCICVRFNQGFLLSQQTIRKLSHI

             310       320       330       340       350       360
Ca  --------------------------------------------MPGTKSALDKSLQLLY
Af  GHDALVVPSMN------------EHDIQALG-MQGIKLGSARDNDEERKRHIEDVVQLLQ
Sp  ----------------------------MDRPHLFSEVHFRRNASNPNQINWTRYWIDCL
Nc  AAAAFSNLQLS------------DLDFKSLGGLAGLQVG--RNTEDEKAKRLDEVISILS
Sc  -----------------------------------------MVEGDSYVETLDSMIELFK
Cg  -------------------------------------------MADQYVEVLGDLIGLLK
Ce  VPKSPLVRNYVNFYRYMTGRVGVKSNLKLLTQFPDEGDIQQQYQVDSKMLTKEDDVVAME

             370       380       390       400       410       420
Ca  NYSTNFQVSIELIQKLAQQLKLETFID-----SLGFNLDDSKP-----------------
Af  TRISGRSVCREGIERLVQLEGFESIWQ---------------------------------
Sp  SSVPWNDISVTGFEYLAKKYGLEVFQDSSKPNE---------------------------
Nc  QNRG--YVSEAGLERLAQKLELDCMWDEHMAGE---------------------------
Sc  DYKPG-SITLENITRLCQTLGLESFTEELSN-----------------------------
Cg  DYKPG-TITIENITKLCQSMGLESFIDELDN-----------------------------
Ce  MYLSDFRDLSKLIETLRSEWKHASLWESILAICESSKGEQKEVNAVDMQLVLRRTEFFLQ

             430       440       450       460       470       480
Ca  --------------GQKTQ-------KLSIAGSSILLDIDF-ETDNKIVGLSLSVNAG--
Af  ------------------------EDNINIAGNFVDLEIEFHRGQNVVKDVSLSYATPDA
Sp  -------------------------VVLSLAGKIILIDITVPINAS-PKDINVVLAFAN-
Nc  -----------------------SKRTLIIAGSALELLIGF--TDNIVQSLTLAFPESSE
Sc  -----------------------ELSRLSTASKIIVIDVDYNKKQDRIQDVKLVLASNFD
Cg  -----------------------DISRLSTASKIIVIDIDYNKTQSTVQDVKLVLASNFD
Ce  FDTDYGSMTMQIFEKSPSNYVVKTTSQITGESPSLDFDKMLTEKLNSTWSIPATLTFAIT

             490       500       510       520       530       540
Ca  --------DSLEQ---QNNHREIGPPNHTTNGQSSSHISIVTDELGIHHVKLNCNNNPIS
Af  TDGERRE----------------------------EATAVLKRDLVQN------------
Sp  --------------ASGEQYNN-------------PVAEKLLKDAIYN------------
Nc  S---------------VNKHAE-------------DAGKILLENLKLQ------------
Sc  N-----------FDYFNQRDGE------------HEKSNILLNSLTKYP-----------
Cg  N------------FDYSNEQLEEAG---------DKQSNILLNSLTNYD-----------
Ce  DLKCRIN-SVLNCVISFDSSSSSDKQHQNYWSISNNNTQLMQSAAQKSAAKCTKKKVTVL

             550       560       570       580       590       600
Ca  FLNKPNDLNKTQVEQILLRNLQASKLGNFPVNLQRLATIDKFTNYN--------------
Af  ----PGDGERGSWKS----------LAGFHGNLQWLSKHDRLS-----------------
Sp  -----------------------CNTPLFEKNVKWLATFDHSSP----------------
Nc  ----PGQSPLT------------KQLDKFSVNFERLAILDKLSIAS--------------
Sc  ------------------------DLKAFHNNLKFLYLLDAYSHIES-------------
Cg  ------------------------SLKQFHHNLEFLYLLDTYSSVESDNQ--------IS
Ce  EVGPAIEPTQLVYTRENDEEFQGSLLSSFGEVDDYYVKTSTIGRTRPQPRPKREPNQLTS

             610       620       630       640       650       660
Ca  --------------PDLFIYVESIALLFNTIFEIE--------QQSNSSNWEIEN---GL
Af  ------------QEVNCFEAIEGLYQSLKRVWDEE--------QKLPQFSGVYEH---LC
Sp  -----------SVQQSCFQYLDSLSSSLNAIYEAELS-------LLAQEENVIMH-----
Nc  -------------VLNLYEAIAGVFDSLSRLHQWELQKVREDAS-LAGRTDEYLRNIILC
Sc  -----------DSTSHNNGSSDKSLDSSNASFNNQGK---LDLFKYFTELSHYIRQCFQD
Cg  SGNNLGSGNGTNGSSLTNKTDKKSVSSGNGLDSKLNEGKLNLFKYFRELKKYINTFFKES
Ce  MDMMSAELDKSRHTMEGSMRDAQTSSFLDAARVRMKQSIGAAHAVGLASHQPPASPVAAA

             670       680       690       700       710       720
Ca  SSSIGKVNINNEDRLGVT----LDFWQDYR------------------------------
Af  AGSIGRPSLHKGSRLGLS----LDYW----------------------------------
Sp  --GNGKPLSNYEGQLGLR----IVYW----------------------------------
Nc  NRS-GTPAMNARGKVGMR----IDYWKN--------------------------------
Sc  NCCDFKVRTNLNDKFGIYILTQGINGKEVP------------------------------
Cg  CDNRFKVVANVDNRFGLYIYETHGYSMRSR------------------------------
Ce  AAAPGGPMRHNSYPSGFEPPPSVAPYDFPADPSKKQRKQRAKKQPGEEPPAGRGKGGKKG

             730       740       750       760       770       780
Ca  ---------------------------------------FINHEYEIVKNQSGLLVGDYY
Af  ------------------------------------------------IHQAKVLDAKHA
Sp  ----------------------------------------------------KLLDKTYS
Nc  ------------------------------------------------------------
Sc  ---------------------------------------LAKIYLEENKSDSQYRFYEYI
Cg  ---------------------------------------PLAKVYFERAKVPQQRFYEYI
Ce  RGAGAVGAAAAGGRKSAGGAGENPFGMDPMRPPMLQRSSSEQHNPNPHQMSQYQMQQYQQ

             790       800       810       820       830       840
Ca  TIQVDIVEAS----KQTDYLLDNQVQEWDISMSKYKFEFDSNSLKPLVLDGDGFLAWSLQ
Af  NSPPDAMDIDTPANRTLDEVAEPQNGKWFLMIEC--------EEGYPSLRVSK--EWVDS
Sp  TQ----IFMDNLSHESLPHLLFGYN-----------------LLNSPPILQSSKINWALE
Nc  ------KHLQPPTNPQTAAYVEKHEKIWGILVGCAPVNRN-LDVNVHPVRVSN--HWISE
Sc  YSQETKSWINESAENFSNGISLVMEIVANAKESNYTDLIWFPEDFISPELIIDKVTCSSN
Cg  YSSETGSWINENSENYSVGVNLILEVNFDDTDEDVFWFPKEFVPTDLFIDEKDQLTSRKV
Ce  NQQFRMHQMQQQQQQQFQMQSPKNPLVPAYTDEDSDEECDPPPPPKPQVSTSSRMSSVPS
```

Med1_1

```
                                      850       860       870       880       890       900
Ca   FKLNHLVYLPVYILEYFSLALGNNYTLDTGDHNCCNVFAKINEGESFEQVLNIDSNSVSF
Af   DV--LTVVNNAANDSSSNEAAASDLAVVNWADP---------------------------
Sp   NTLTPIPTTMELVFDDINLIIPEQGVKP----------------------------LLDL
Nc   NV----ELPHIPGDLQTS-------PMVHWLEP---------------------------
Sc   SSSSPPIIDLFSNNNYNSRIQLMNDFTTKLINIKKFDISNDNLDLISEILKWVQWSRIVL
Cg   SDVLCHAFYELGSSKKQTIELMNDFTTDLIQIRRFNINNDNLDLIADILNWTIWYRTVLR
Ce   HPPPPPQQLGNPMVGYPGMPLQSPNHLPLTPSPLSAPPKPFSPEQQHHFGMKMRETSYWK

                                      910       920       930       940       950       960
Ca   QLTNDIPGKFVPLKSFHINKLVDISKLIPILRNFLVLINLCRTVIKSKLTQLPVFNSKRR
Af   PPTLNSGGSDAMALDSNMLGSSALN-------RRFVARMEPPLDVPILAAS---------
Sp   LHVHDITIPWPVQNYSHMLGLPSKSTVNYKFINKNQAIQLTGFDVSARSLR---------
Nc   --EDTIVPPDPEKAGDSLLGPRLPS-------VTFSATFDPPIHIS------------FG
Sc   QNVFKLVSTPSSNSNSSELEPDYQAPFSTSTKDKNSSTSNTEPIPRSNRHGSVVEASRRR
Cg   PIYLKLVSSISDDDDQHIRADIANTKDEFTVENTNPEIMKGPTNKNASFMQRNSMSLQRR
Ce   ELERLETKPDVEKLKQQLSSSSSSQAEASDPPSTSTEAQDPPKPSSSSAPPPPKKKLGL

                                      970       980       990      1000      1010      1020
Ca   RSSRFLGIPTTERELTEEFKHKLKESLKLPDDVTDEEILGLSAISETANYSTIPPANKDD
Af   --DIYRHLGIQLPHEFRMVTYDGLLVP-------------------------GSSPLSAT
Sp   ----------------------------------------------------HVPFHHPK
Nc   LWEQLRSMGIGIPAMDVSKSFDN----------------------------------LVF
Sc   RSSTNKSKRPSITEAMMLKEEGLQQFN----------------------------LHEIL
Cg   RRSSNKAKRPSMSEAVVFKDEGLQQFS---------------------------------
Ce   EAAISKIRGQQEQALQKQLQQQESVESELATPHPGSSGAPPLAPHQVNRARNLNDIFDDE

                                     1030      1040      1050      1060      1070      1080
Ca   IDLESFLGNEDTTNSGDVDTNGKRGTDHLPVNGNNYFLLKLESIDLSSRGDLQLAFESQF
Af   GAVGLGAEDITQPGRKRRRMS---VQGFDQE---GNPCTKHHSYTFQAFES---------
Sp   QIRGILAIVRQYLLLQLILENIKSADLAETAASSSLHLSLYFKEHPIVHAQYQEINKLNN
Nc   PIAPGGYYDPTEPREIHHTKDIN-----YQLPGSPAWVSRKHANSLYIYKP---------
Sc   SEPAIEEENGDSIKEHSTTMDGANDLGFTASVSNQENAGTDIVMEDHGVLQGTSQNYGTA
Cg   ---------------LHEIMADTTTEPDQKPDNLNISNNSESFIGQQELMEGSNIINDDK
Ce   ADDSSPTADIKPSLQALQKPTDTSSQSTSSEPPPKKEPADEQPEREKEKLILKIPKMLKP

                                     1090      1100      1110      1120      1130      1140
Ca   KSEEVYIPFRISNGEIVKLQNSDDMETDDTPSSKFIKALNLTEDIISSYKAVYSN-----
Af   -----VAGRTMRDIPFSHPRQLADVFPILRQYALLANLIYKIFNSPQN--DDGSKKDAES
Sp   SEQIIIVLSVLSDGRLNVDYMSSGGKELSKQQSHVFQKLIQQTCNIGLAIEVFIKKVVN-
Nc   -----VYGKTLTEASFSHPQHLIAMLPYLRQYAFLSTLLENSFKEDTGPKRTPPQTTAHP
Sc   TADDADIEMKDVSSKPSKPESSVLQLIVSEDHIILDTISECNLYDDVKCWSKFIEKFQDI
Cg   MDIDEEAINDDDSDSQSGEEGNIPVLLVNEDHVSFKGLGSCSFYDDKDKWAKFVEDLFNV
Ce   VVDDRREDRKERDRDRDRDRDRDEDREKVRDKEDKAQREKDKKEKERERRRQRDRDRTEQ

                                     1150      1160      1170      1180      1190      1200
Ca   ------------------------------------------------------------
Af   VTEGTFRQANISKAIMPTKKNKVIVLSNKNPNEEK---LDFLLNGTNTSNATATGLGKDI
Sp   ------------------------------------------------------------
Nc   DSFSTKTSTNRDEFNRFMSLSPTLVKPPSDEAPTPMDISTSMSIDASSQPNHSGTVPMSI
Sc   VS----------------------------------------------------------
Cg   FS----------------------------------------------------------
Ce   KKSDREKEPSKKRKLEKKDEKEKDRREPERKKGKSDGKEYSKASTTSLIPTLSLKNFRIP

                                     1210      1220      1230      1240      1250      1260
Ca   ------------------------------------------------------------
Af   TMSPSISLRDPATHQED-------------------------------------------
Sp   ------------------------------------------------------------
Nc   DGGASQHIFPSQAAHSQPTSHLSSNNNNNQNHQVPPTSSGGPNNHSTLTHTFTSGPDPEA
Sc   ------------------------------------------------------------
Cg   ------------------------------------------------------------
Ce   KKDTVEEEKKDVKEEAPGPSSSTESKKEPPSAAPITRKESTTAPVAPAVQQQQQRKESFT

                                     1270      1280      1290      1300      1310      1320
Ca   ------------------------------------------------------------
Af   ------------------------------------------------------------
Sp   ------------------------------------------------------------
Nc   IKIDINLTMVHAPRLQIAFPFRASETAIVVLEIRENGQVHVESQNVLDERNSVAPNGRER
Sc   ------------------------------------------------------------
Cg   ------------------------------------------------------------
Ce   ALPSLPEQQQHHREPLLKKKPPLQPPPPPQMTGPPIGIYPGPPAPGNLPGSSRPSGNRKQ

                                     1330      1340      1350      1360      1370      1380
Ca   ------------------------------------------------------------
Af   ------------------------------------------------------------
Sp   ------------------------------------------------------------
Nc   RVEDLGKMLETVEDIGKWVEFVRTRWA---------------------------------
Sc   ------------------------------------------------------------
Cg   ------------------------------------------------------------
Ce   PLPPPPPMIRGPPPDQMYRDRSGGGGGSMRGGFPPASHYHGGTSGVNKQVASYAQGLPPG

                                     1390      1400      1410      1420      1430      1440
Ca   ------------------------------------------------------------
Af   ------------------------------------------------------------
Sp   ------------------------------------------------------------
Nc   ------------------------------------------------------------
Sc   ------------------------------------------------------------
Cg   ------------------------------------------------------------
Ce   MGPPVVKPHGNNYQASQWVRPPTHRDSSHSYHGMPSLGPPQLQREPPAPPPPQMIPLPKD

                                     1450      1460      1470
Ca   ------------------------------------
Af   ------------------------------------
Sp   ------------------------------------
Nc   ------------------------------------
Sc   ------------------------------------
Cg   ------------------------------------
Ce   PPVLREHPREHRAHRGGADDDGPDSPEEGTLRIDDE
```

Med1_2

```
    1 10 20 30 40 50 60
Mm  ------------------------------MSSLLERLHAKFNQNRPWSETIKLVRQVME
Dm  MSGSNAKSSGTGFGSHIPSIEEKNKQIQQETMMEKLRAKYRNKPKSYEEIKKSVRMYFIE
Hs  ---------------MKAQGETEESEKLSKMSSLLERLHAKFNQNRPWSETIKLVRQVME

    70 80 90 100 110 120
Mm  KRVVMSSGGHQHLVSCLETLQKALKVTSLPAMTDRLESIARQNGLGSHLSASGTECYITS
Dm  KHYPLDPLCKATLQSALDKLQHYIKVTSRHGLVERLESLSRQ--LGLKFMEDQQLLFIST
Hs  KRVVMSSGGHQHLVSCLETLQKALKVTSLPAMTDRLESIARQNGLGSHLSASGTECYITS

    130 140 150 160 170 180
Mm  DMFYVEVQLDPAGQLCDVKVAHHGENPVSCPELVQQLREKNFEEFSKHLKGLVNLYNLPG
Dm  DMFYVEILLDAAGSLSDVKVHHECKIEQQSSELVACLKSGDFADFTVQLEGLSSIYQLNA
Hs  DMFYVEVQLDPAGQLCDVKVAHHGENPVSCPELVQQLREKNFDEFSKHLKGLVNLYNLPG

    190 200 210 220 230 240
Mm  DNKLKTKMYLALQSLEQDLSKMAIMYWKATNAAPLDKILHGSVGYLTPRSGGH-----LM
Dm  EPKVKKKAFVALQAMETDIQSLYQLHLQGHSGDSYSLMTSSSVGLVLPRRGGH-----PM
Hs  DNKLKTKMYLALQSLEQDLSKMAIMYWKATNAGPLDKILHGSVGYLTPRSGGHLMNLK--

    250 260 270 280 290 300
Mm  NMKYYASPSDLL--DDKTASPIILHEKNVPRSLGMNASVTIEGTSA--MYKLPIAPLIMG
Dm  RLTYFCPPLHLPEGDPKLASGDFTIDQVMRSSYGLSATINLEGSSANKLQTLPTVTLVRD
Hs  ---YYVSPSDLL--DDKTASPIILHENNVSRSLGMNAS--VTIEGTSAVYKLPIAPLIMG

    310 320 330 340 350 360
Mm  SHPADNKWTPSFSAVTSANSVDLPACFFLKFPQPIPVSKAFVQKLQNCTGIPLFETPPTY
Dm  AQ--TGLEVPTYAQLNQNNSLLMPATFVLRLNKPMPVCLESLKALG----LPGLDSVATP
Hs  SHPVDNKWTPSFSSITSANSVDLPACFFLKFPQPIPVSRAFVQKLQNCTGIPLFETQPTY

    370 380 390 400 410 420
Mm  LPLYELITQFELSKDPDPLPLNHNMRFYAALPGQQHCYFLNKDAPLPDGQSLQGTLVSKI
Dm  PGPPTTVLNLIVQTASKQAIKNTQRGLYVNLPKETHCYFFT------DNRKLQGTLVSSL
Hs  APLYELITQFELSKDPDPIPLNHNMRFYAALPGQQHCYFLNKDAPLPDGRSLQGTLVSKI

    430 440 450 460 470 480
Mm  TFQHPGRVPLILNMIRHQVAYNTLIGSCVKRTILK-EDSPGLLQFEVCPLSESRFSVSFQ
Dm  PFTEPAQVPRIVAFLKKQALFYTLLASCVREQQKQYNDMDSTVILEVTAVSFNQITVELQ
Hs  TFQHPGRVPLILNLIRHQVAYNTLIGSCVKRTILK-EDSPGLLQFEVCPLSESRFSVSFQ

    490 500 510 520 530 540
Mm  HPVNDSLVCVVMDVQDSTHVSCKLYKGLSDALICTDDFIAKVVQRCMSIPVTMRAIRRKA
Dm  HPYEESLATVDFLLEDG-QPTCSVY-CLTNEYELLSQKLTRTARKVVSIPMVIYKLLKCW
Hs  HPVNDSLVCVVMDVQDSTHVSCKLYKGLSDALICTDDFIAKVVQRCMSIPVTMRAIRRKA

    550 560 570 580 590 600
Mm  ETIQADTPALSLIAETVEDMVKKNLPPASSPGYGMTTGNNPMSGTTTPTN--TFPGGPIT
Dm  DEEHEFKLHGAIGPGSGSGAIGGGGMSGGGPVSGVGNNFSQFSMDTPTPSDGSLPGGGFA
Hs  ETIQADTPALSLIAETVEDMVKKNLPPLAGPGYGMTTGNNPMSGTTTPTN--TFPGGPIT

    610 620 630 640 650 660
Mm  TLFNMSMSIKDRHESVGHGEDFSKVSQNPILTSLLQITGNGGSTIGSSPTPPHHTPPPVS
Dm  NINNLKMDAKSRSLADAFAASTSAAAAIAGLINLKRETDPQSGSSASGTTVSGSSSSSGS
Hs  TLFNMSMSIKDRHESVGHGEDFSKVSQNPILTSLLQITGNGGSTIGSSPTPPHHTPPPVS

    670 680 690 700 710 720
Mm  SMAGNTKNHPMLMNLLKDNPAQDFSTLYGSSPLERQNSSSGSPRMEMCSGSNKAKKKKSS
Dm  AKTSDHDIADKYKNIWKDKTPNLKHCVSITPIPGDGKSGSAG------GVSGVEVQRTGG
Hs  SMAGNTKNHPMLMNLLKDNPAQDFSTLYGSSPLERQNSSSGSPRMEICSGSNKTKKKKSS

    730 740 750 760 770 780
Mm  RVPPDKPKHQTEDDFQRELFSMDVDSQNPMFDVSMTADALDTPHITPAPSQCSTPPATYP
Dm  IEIIPLNAQAAIAGGGVTASSSATPTTITITPITGKDPSKDSTKKSTAASAGVGVAAKRP
Hs  RLPPEKPKHQTEDDFQRELFSMDVDSQKPIFDVNMTADTLDTPHITPAPSQCSTPPTTYP

    790 800 810 820 830 840
Mm  QPVSHPQPSIQRMVRLSSSDSIGPDVTDILSDIAEEASKLPSTSDDCPPIGTPVRDSSSS
Dm  HESSTSSSSTSGCSGSGSSMSSSASSGSSDTQKEKKRKKKRDDSPMGPPEKIYSRQNSPA
Hs  QPVPHPQPSIQRMVRLSSSDSIGPDVTDILSDIAEEASKLPSTSDDCPAIGTPLRDSSSS

    850 860 870 880 890 900
Mm  GHSQSALFDSDVFQTNNNENPYTDPADLIADAAGSPNSDSPTNHFFPDGVDFNPDLLNSQ
Dm  GGADASATGGVVRKFSSPSSSPKAGGGGQGLMAGVPTARPSPKHSPVYSSPKHNTASNSP
Hs  GHSQSTLFDSDVFQTNNNENPYTDPADLIADAAGSPSSDSPTNHFFHDGVDFNPDLLNSQ

    910 920 930 940 950 960
Mm  SQSGFGEEYFDESSQSGDNDDFKGFASQALNTLGMPMLGGDNGEPKFKGSSQADTVDFSI
Dm  KSPFGTHSPKHGSSGKPSMSTLKSAATAATILSPKGDKSSSAVGNTSSGPSASSGSSGAT
Hs  SQSGFGEEYFDESSQSGDNDDFKGFASQALNTLGVPMLGGDNGETKFKGNNQADTVDFSI

    970 980 990 1000 1010 1020
Mm  ISVAGKALGAADLMEHHSGSQSPLLTSGELGKEKTQKRVKEGNGTGASSGSGPGSDSKPG
Dm  GLVRSFASVGAPPPPPPIPPLASSSGSISSSQSLKKEKTSSASGSSSTSSSATAGVASGG
Hs  ISVAGKALAPADLMEHHSGSQGPLLTTGDLGKEKTQKRVKEGNGTSNSTLSGPGLDSKPG

    1030 1040 1050 1060 1070 1080
Mm  KRSRTPSNDGKSKDKPPKRKKADTEGKSPSHSSSNRPFTPPTSTGGSKSPGS---SGRSQ
Dm  GISPASVAAAVAALKSSQQQMKSVASLSHLAAGGGLGSYAAPSGAGASGAAAVVVGAGAG
Hs  KRSRTPSNDGKSKDKPPKRKKADTEGKSPSHSSSNRPFTPPTSTGGSKSPGS---AGRSQ

    1090 1100 1110 1120 1130 1140
Mm  TPPGVATPPIPKITIQIPKGTVMVGKPSSHSQYTSSGSVSSSGSKSHHSHSSSSSSLASA
Dm  AGAGASGLELSALRKGMAGGAVSLMTSTAALAPTIPAPTTTVAAGSAASLVSPVSAVVGQ
Hs  TPPGVATPPIPKITIQIPKGTVMVGKPSSHSQYTSSGSVSSSGSKSHHSHSSSSSS--SA

    1150 1160 1170 1180 1190 1200
Mm  STSGKVKSSKSEGSSSSKLSGSMYASQGSSGSSQSKNSSQTGGKPGSSPITKHGLSSGSS
Dm  GQETAGAAAAATLATATILQQQPQPGAAPTSSCLTTSGGSSDSAGSINPAGASTEYMVKP
Hs  STSGKMKSSKSEGSSSSKLSSSMYSSQGSSGSSQSKNSSQSGGKPGSSPITKHGLSSGSS

    1210 1220 1230 1240 1250 1260
Mm  STKMKPQGKPSSLMNPSISKPNISPSHSRPPGGSDKLASPMKPVPGTPPSSKAKSPISSG
Dm  SSQEGLKLTINKTGSSKSSGTGSGSSSSSGLQAKAKSSSSGATSFAGSTGSTKKQHTGLK
Hs  STKMKPQGKPSSLMNPSLSKPNISPSHSRPPGGSDKLASPMKPVPGTPPSSKAKSPISSG

    1270 1280 1290 1300 1310 1320
Mm  SSGSHVSGTSSSSGMKSSSGSASSGSVSQKTPPASNSCTPSSSSFSSSGSSMSSSQNQHG
Dm  PGVNSGPASKKATAAVSSATASSSKHFFQKANSSGNLSSKLSGSGSGGGIPLTKSNSTNS
Hs  SGGSHMSGTSSSSGMKSSSGLGSSGSLSQKTPPSSNSCTASSSSFSSSGSSMSSSQNQHG

    1330 1340 1350 1360 1370 1380
Mm  SSKGKSPSRNKKPSLTAVIDKLKHGVVTSGPGGEDPIDSQMGASTNSSNH-----PMSSK
Dm  FQEHNAPRRRPSMGALASGSSGGGSGQRKLGSASGGGSGSSGSVSPALSGSMSQPPPRFD
Hs  SSKGKSPSRNKKPSLTAVIDKLKHGVVTSGPGGEDPLDGQMGVSTNSSSH-----PMSSK

    1390 1400 1410 1420 1430 1440
Mm  HNMSGGEFQSKREKSDKDKSKVSASGGSVDSSKKTSESKNVGSTGVAKIIISKHDGGSPS
Dm  HHTDMMTILQYASPTMAASMEGFIKGLHNKFQIPKLSQRGSGGNTTSGRSTPSGSSEPAL
Hs  HNMSGGEFQGKREKSDKDKSKVSTSGSSVDSSKKTSESKNVGSTSVAKIIISKHDGGSPS

    1450 1460 1470 1480 1490 1500
Mm  IKAKVTLQKPGESGGDGLRPQIASSKNYGSPLISGSTPKHERGSPSHSKSPAYTPQNVDS
Dm  AGTSSSILGPIASSTGLTEPEAKPPVPPSQSGNEGLLNLSSTAGTPSADGIDEELLASLA
Hs  IKAKVTLQKPGESSGEGLRPQMASSKNYGSPLISGSTPKHERGSPSHSKSPAYTPQNLDS
```

Med1_2

```
                                       1510                1520                1530                1540                1550                1560
Mm    E S E S G S S I A E R S Y Q N S P S S E D G I R P L P E Y S T E K H K K H K K E K K K V R - - - - - - - - D K D R D K K
Dm    G E - - - - - - - - - - - - - - - - - - - - - - - - - - - - - - - - - - - - - - - - - - - - - - - - - - - - - - - - - -
Hs    E S E S G S S I A E K S Y Q N S P S S D D G I R P L P E Y S T E K H K K H K K E K K K V K D K D R D R D R D K D R D K K

                                       1570                1580                1590                1600                1610
Mm    K S H S M K P E N W S K S P I S S D P T A S V T N N P I L S A D R P S R L S P D F M I G E E D D D L M D V A L I G N
Dm    - - - - - - - - - - - - - - - - - - - - - - - - - - - - - - - - - - - - - - - - - - - - - - - - - - - - - - - - - -
Hs    K S H S I K P E S W S K S P I S S D Q S L S M T S N T I L S A D R P S R L S P D F M I G E E D D D L M D V A L I G N
```

Med4

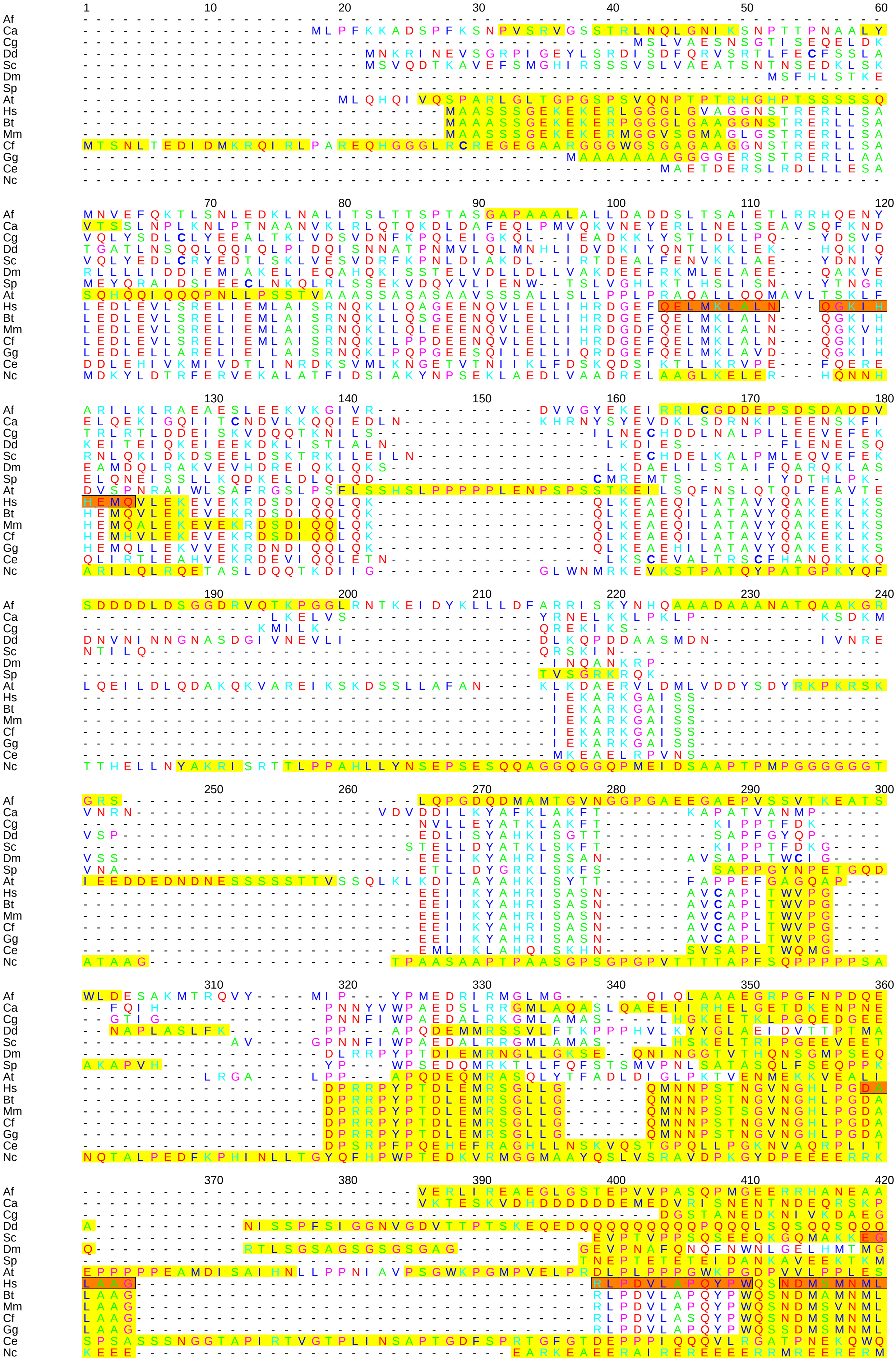

Med4

```
            430       440       450       460       470       480
Af   EAAAQAGAASSGTMAAPAPAPKPKATLDLDLYDPDDDEMM--------------------
Ca   PAASEHDTSKRKEEQNQQPVDLNLDLFDPDDEYSD-------------------------
Cg   AEGEIRQDDKKEDDSFVFGANANDAEGDEDKNAGEDEDEAMDSDLDLFNPDEF-------
Dd   TESELQPIQSILQPPQQLNIDLDLNPDLDSSGDDDDEDDDDEESEEVEWD----------
Sc   TP---------KTDSFIFDGTAKEVGDEADNTKDKEKEENNDDALDLDLDLFDPDDF---
Dm   ASGNTVALETRAQDDVEVMSTDSSSSSSSDSQ----------------------------
Sp   NYPASPTFTTQEENKEVESPANKDVFAGFDLFDPEMEEDF--------------------
At   IAAPRAEDHQHMRPSQGLHRPPDVIQVRAVQLDILEDDDSSDYSSDDASSDDED------
Hs   PPN--------HSSDFLLEPPGHNKENEDDVEIMSTDSSSSSSESD--------------
Bt   PPN--------HSHDFLLEPPGHNKENEDDVEVMSTDSSSSSSDSD--------------
Mm   PPN--------HSSDFLLEPPGHNKENEDDVEVMSTDSSSSSSDSD--------------
Cf   PPN--------HSNDFLLEPPGHNKENEDDVEVMSTDSSSSSSDSD--------------
Gg   PPN--------HSNDFMLEPPGHNKENEDDVEVMSTDSSSSSSDSD--------------
Ce   NPGVSGATSTQSPYNRVSQSPSSSPNVKLKITGLPNRPGGIDQVQDVRDVEQMSSDSSNS
Nc   ARERERARREEAERSGSISGPAAGAPAAAASSAGGGGRSQFTFLDGMDDDDDDDED----

            490       500       510       520       530       540
Af   ------------------------------------------------------------
Ca   ------------------------------------------------------------
Cg   ------------------------------------------------------------
Dd   ------------------------------------------------------------
Sc   ------------------------------------------------------------
Dm   ------------------------------------------------------------
Sp   ------------------------------------------------------------
At   ------------------------------------------------------------
Hs   ------------------------------------------------------------
Bt   ------------------------------------------------------------
Mm   ------------------------------------------------------------
Cf   ------------------------------------------------------------
Gg   ------------------------------------------------------------
Ce   SDSSDDEGASKKTGNMVTEMSHAESIKRVVDQKLSSHEGFESHMFKIGSYNEAVGESSPF
Nc   ------------------------------------------------------------

            550       560       570       580       590       600
Af   ------------------------------------------------------------
Ca   ------------------------------------------------------------
Cg   ------------------------------------------------------------
Dd   ------------------------------------------------------------
Sc   ------------------------------------------------------------
Dm   ------------------------------------------------------------
Sp   ------------------------------------------------------------
At   ------------------------------------------------------------
Hs   ------------------------------------------------------------
Bt   ------------------------------------------------------------
Mm   ------------------------------------------------------------
Cf   ------------------------------------------------------------
Gg   ------------------------------------------------------------
Ce   ALPYDDSTMALLILSTPDMFDVAFRKWVVQKTMDFGSFDEVCEMVSSPIQSFLEDRLEIM
Nc   ------------------------------------------------------------

            610       620       630       640       650       660
Af   ------------------------------------------------------------
Ca   ------------------------------------------------------------
Cg   ------------------------------------------------------------
Dd   ------------------------------------------------------------
Sc   ------------------------------------------------------------
Dm   ------------------------------------------------------------
Sp   ------------------------------------------------------------
At   ------------------------------------------------------------
Hs   ------------------------------------------------------------
Bt   ------------------------------------------------------------
Mm   ------------------------------------------------------------
Cf   ------------------------------------------------------------
Gg   ------------------------------------------------------------
Ce   SEKLRKVEENFEILHDYSMTPQRRPKILMQTCGHVAGAAFYYQPCHFQEDGVTWPPAGRM
Nc   ------------------------------------------------------------

            670       680       690       700       710       720
Af   ------------------------------------------------------------
Ca   ------------------------------------------------------------
Cg   ------------------------------------------------------------
Dd   ------------------------------------------------------------
Sc   ------------------------------------------------------------
Dm   ------------------------------------------------------------
Sp   ------------------------------------------------------------
At   ------------------------------------------------------------
Hs   ------------------------------------------------------------
Bt   ------------------------------------------------------------
Mm   ------------------------------------------------------------
Cf   ------------------------------------------------------------
Gg   ------------------------------------------------------------
Ce   GPNLKFIGLSLHPIYGGHFAFRSVLIFPNVKIPEFCEKEPRPILTASEDVRTALEKFNYN
Nc   ------------------------------------------------------------

            730       740       750       760
Af   -----------------------------------------------
Ca   -----------------------------------------------
Cg   -----------------------------------------------
Dd   -----------------------------------------------
Sc   -----------------------------------------------
Dm   -----------------------------------------------
Sp   -----------------------------------------------
At   -----------------------------------------------
Hs   -----------------------------------------------
Bt   -----------------------------------------------
Mm   -----------------------------------------------
Cf   -----------------------------------------------
Gg   -----------------------------------------------
Ce   WKDSGFRDFGNPTRRYSTTQMEFFGRPVAERWEVLRPWVDGGAKNID
Nc   -----------------------------------------------
```

Med6

```
    1        10        20        30        40        50        60
Cp  ------------------------------------------------------------
Af  ------------------------------------------------------------
Sc  ------------------------------------------------------------
Nc  ------------------------------------------------------------
Ce  ------------------------------------------------------------
At  ------------------------------------------------------------
Ca  ------------------------------------------------------------
Dm  ------------------------------------------------------------
Gg  VLESMYDVRPYFTPQCLPVWVWRSHARELELSGPGGMQASSCCLQQQADSNKGNPVLKRA
Os  ------------------------------------------------------------
Su  ------------------------------------------------------------
Bt  ------------------------------------------------------------
Cf  ------------------------------------------------------------
Hs  ------------------------------------------------------------
Pt  ------------------------------------------------------------
Rn  ------------------------------------------------------------
Mm  ------------------------------------------------------------
Dd  ------------------------------------------------------------
Sp  ------------------------------------------------------------

             70        80        90        100       110       120
Cp  ------------------------------------------------------------
Af  ---------------------------------------------------MAGPQDASL
Sc  -------------------MNVTPLDELQWKSPEWIQVFGLRTENVLDYFAESPFFDKTS
Nc  --------------------------------------------------MAFDPAANAG
Ce  -------------------------------------------------------MMPRM
At  ----------------------------------------MDSSLLSAATADTFNGNAAD
Ca  ---------------------------------------------------------MES
Dm  ------------------------------------------------------------
Gg  PAAAIVAATSVRSSLWHFSRRSLGLPALSGAAPSRRGGVRLPAGVPAPLCPTSGARRAGG
Os  ------------------------------------------------------MSGTPL
Su  ------------------------------------------------------------
Bt  ------------------------------------------------------------
Cf  ------------------------------------------------------------
Hs  ------------------------------------------------------------
Pt  ------------------------------------------------------------
Rn  ------------------------------------------------------------
Mm  ------------------------------------------------------------
Dd  --------------------------------------------------------MISD
Sp  --------------------------------------------------MASGAPPSVD

             130       140       150       160       170       180
Cp  -------------------IFKENLGSTLSKFLVRIIHHFSGLYYSLLRSLCILAKKFE-
Af  EEILWRSPPHVQMMGGYLHSNNILFYFAESPFFDATSNNASLAIQANYNEAFRHFVETR-
Sc  NNQVIKMQRQFSQLNDPNAAVNMTQNIMTLPDGKNGNLEEEFAYVDPARRQILFKYPMY-
Nc  PPLDEIQWHTPPQFEAGIHSNSILYYFAQSPFYDKTSNNEVVFQQGLNNQAMSQYLATR-
Ce  GPPAAARQDNPLHVSFRNPQWPPNFINKDNVLDYFCNQANAFYEMNSCNQQIRMQNIVN-
At  QIPPPLQPPGTDMTGISFRDQLWINSYPLDRNYIFDYFALSPFYDTTCNNEILRRRSIH-
Ca  LDEIQWKSPEFIQERGLNTNNVLEYFSLSPFYDRTSNNQVLMMQFQYQQIQIPPGVSFH-
Dm  ---MASRQMTNDHLRLSWHDTQMMATLSPQTVMDYFCRKSNPFYDHMCNNETVR---MQ-
Gg  AGAMAAVDVRDNLLGISWVDSAWIPILNNGSVLDYFSERSNPFYDRTCNNEVVKMQR---
Os  PPPALPPPPGTDMTGICFRDQLWLNTYPLDRNLVFDYFALSPFYDLTCNNESLRSRQIH-
Su  ------MSTDDNSLHISWHDSAWIPHLNQANIMDYFSNRSNPFYDRTCNNEIIKMQR---
Bt  ---MAAVDIRDNLLGISWVDSSWIPILNSGSVLDYFSERSNPFYDRTCNNEVVKMQR---
Cf  ---MAAVDIRDNLLGISWVDSSWIPILNSGSVLDYFSERSNPFYDRTCNNEVVKMQR---
Hs  ---MAAVDIRDNLLGISWVDSSWIPILNSGSVLDYFSERSNPFYDRTCNNEVVKMQR---
Pt  ---MAAVDIRDNLLGISWVDSSWIPILNSGSVLDYFSERSNPFYDRTCNNEVVKMQR---
Rn  ---MAAVDIRDNLLGISWVDSSWIPILNSGSVLDYFSERSNPFYDRTCNNEVVKMQR---
Mm  ---------------------------------------------------------MQ-
Dd  TSTPRILEKDPDLTQVMWRDPLWLQMYPLNPQTILQYFSYSQFYDKNCNNEQLKMQRLDN
Sp  LTSIQWRMPEWVQSMGGLRTENVLEYFSQSPFYSHKSNNEMLKMQSQFNALDLG------

             190       200       210       220       230       240
Cp  -------FILFHIFSASEGFEYRITYINEEGLGNVPQPPGSTWNTPNQFGIFIIQKFFIE
Af  -------EAFEGRLRTMQGLEFVVAYDPLQ-----AAAQTDTQFAHEPSNVWVIRKQMRR
Sc  -------MQLEEELMKLDGTEYVLSSVREP-------------------DFWVIRKQRRT
Nc  -------ELFESRLKEMSGLEFIVAQEPAETG------------PGMGTGVWVINKQTRR
Ce  -------RTVEECLRTMPGIQYVLWYSQP--------------------PLFIICKQRRN
At  -------PLDLSHLSKMTGLEYMLTDATEP-------------------NLFVFRKQKRD
Ca  -------QYFQSRLSEMTGIEFVIAYTKEP-------------------DFWIIRKQKRQ
Dm  -------RLGPEHLHNMIGLEYILLHVAEP-------------------ILYVIRKQHRH
Gg  --------MTLDHLNQMVGVEYILLHAQEP-------------------ILFIIRKQQRQ
Os  -------PLDMSHLTKMTGMEYVLSDVMEP-------------------HLFVIRKQRRE
Su  --------LSPEQLKSMTGLEYILLHVQEP-------------------ILYVVRKQHRH
Bt  --------LTLEHLNQMVGVEYILLHAQEP-------------------ILFIIRKQQRQ
Cf  --------LTLEHLNQMVGVEYILLHAQEP-------------------ILFIIRKQQRQ
Hs  --------LTLEHLNQMVGIEYILLHAQEP-------------------ILFIIRKQQRQ
Pt  --------LTLEHLNQMVGIEYILLHAQEP-------------------ILFIIRKQQRQ
Rn  --------LTLEHLNQMVGIEYILLHAQEP-------------------ILFIIRKQQRQ
Mm  -------RLTLEHLNQMVGIEYILLHAQEP-------------------ILFIIRKQQRQ
Dd  QQQPQYIKTNIFKFRNMDGLEYELIKFVEP-------------------SFFLIAKQTRI
Sp  --------DLNSQLKRLTGIQFVIIHERPP-------------------FLWVIQKQNRL

             250       260       270       280       290       300
Cp  F--------------QREIPLNIYYILSGTIYEAPTLSNVFLHRTAEAFLSMEKIFDTLS
Af  KRAG---------QEDEVVVLATFFVVGDCIYMAPSVASVVGNRIVRRRWFCFAHVRKGM
Sc  NNSGVG-----SAKGPEIIPLQDYYIIGANIYQSPTIFKIVQSRLMSTSYHLNSTLESLY
Nc  KRPPTNPARPEDGPPDEIIVHSVYFVVGENIYMAPTLADVLSSRIGAIATAITKTIPLVD
Ce  N-------------VTNVSPIAYYYVINGSVHQAPDMYSLVQSRLLGALEPLRNAFGEVT
At  G-------------PEKVTPMLTYYILDGSIYQAPQLCSVFAARVSRTIYNISKAFTDAA
Ca  D-------------PQNTVTLQDYYIIGANVYQAPRIYDVLSSRLLASVLSIKNSTDLLN
Dm  N-------------PSEATPIADYYIIGGTVYKAPDLANVINSRILNTVVNLQSAFEEAS
Gg  S-------------PTQVIPLADYYIIAGVIYQAPDLGSVINSRVLTAVHGIQSAFEEAM
Os  S-------------PEKSNAMLAYYILDGSIYQAPQLCSVFASRISRAMHHISKAFTTAC
Su  S-------------LEESTPLADYYIVAGRVYQAPDICAVINARMTTAVHNIRSAFEESL
Bt  S-------------PTQVIPLADYYIIAGVIYQAPDLGSVINSRVLTAVHGIQSAFDEAM
Cf  S-------------PTQVIPLADYYIIAGVIYQAPDLGSVINSRVLTAVHGIQSAFDEAM
Hs  S-------------PAQVIPLADYYIIAGVIYQAPDLGSVINSRVLTAVHGIQSAFDEAM
Pt  S-------------PAQVIPLADYYIIAGVIYQAPDLGSVINSRVLTAVHGIQSAFDEAM
Rn  S-------------PAQVIPLADYYIIAGVIYQAPDLGSVINSRVLTAVHGIQSAFDEAM
Mm  S-------------PAQVIPLADYYIIAGVIYQAPDLGSVINSRVLTAVHGIQSAFDEAM
Dd  S-------------PTDVLINTLYYVINGNIYQAPELHVVFKSRVSQSISHLSEAFNSIS
Sp  N-------------ENEVKPLTVYFVCNENIYMAPNAYTLLATRMLNATYCFQKALTKIE

             310       320       330       340       350       360
Cp  EMSRFSLYEGYTWDNLEVTSKINSDIENADSKNTNSGEEESLGTFLLFLFCVFVKLNYFN
Af  ADQLASALCCHIANQSFENRLDAANLHPGPWAYLSTTSSETR------------------
Sc  DLIEFQPSQGVHYKVPTDTSTTATAATNGNNAGGGSNKSSVRPTGGANMATVPSTTNVNM
Nc  EVSDWAPAVGRRYITPAQPSAGAGAASGTTNYTASRTATPLPDGLPSTATTNKPGAKAGG
Ce  NYSRYNTAKGYYWEFKNKPNVKKREEEKKEDEEEKLEDRSTNFQKTRTMMLLNQLFSEMP
At  SKLETIRQVDTENQNEPAESKPASETVDLKEMKRVDVILTSLYRKLAPPPPPPPFPEGYV
Ca  DMTSYHISDGGHSYINSIHGSSSKPSQSSAVSKPLSTNTGTNATTTPITLTTPLGATVPS
Dm  SYARYHPNKGYTWDFSSNKVYRSKSDKKDANSAKDENSGTLFQKQRVDMLLAELLRKFPP
Gg  SYCRYHPSKGYWWHFKDQEEREKAKPKAKKKEEPSSIFQRHRVDALLLDLRQKFPPRFVQ
Os  SKLEKIGHVETEPDTAASESKTQKEAIDLKELKRVDHILMSLQRKLQPAPPPPPFPEG--
Su  SYSRYHPSKGYWWEFKEKDSETEKKKRKKPKEELSSAFQRKRVDILLGDLSRKFPFKVIQ
Bt  SYCRYHPSKGYWWHFKDHEEQDKVKPKVKRKEEPSSIFQRQRVDALLLDLRQKFPPKFVQ
Cf  SYCRYHPSKGYWWHFKDHEEQDKVKPKAKRKEEPSSIFQRQRVDALLLDLRQKFPPKFVQ
Hs  SYCRYHPSKGYWWHFKVHEEQDKVRPKAKRKEEPSSIFQRQRVDALLLDLRQKFPPKFVQ
Pt  SYCRYHPSKGYWWHFKDHEEQDKVRPKAKRKEEPSSIFQRQRVDALLLDLRQKFPPKFVQ
Rn  SYCRYHPSKGYWWHFKDHEEQDKVKPKAKRKEEPSSIFQRQRVDALLIDLRQKFPPRFVQ
Mm  SYCRYHPSKGYWWHFKDHEEQEKVKPKAKRKEEPSSIFQRQRVDALLIDLRQKFPPRFVQ
Dd  SIVNWDIANGY-------------------------------------------------
Sp  KFPQYNPQEGYTYPKLSNDNLEVDHSNTNEPADENKNQSIENADYSFSPEDFSVVRAFMQ
```

Med6

```
           370       380       390       400       410       420
Cp  ------------------------------------------------------------
Af  ------------------------------------------------------------
Sc  TVNTMGTGGQTIDNGTGRTGNGNMGITTEMLDKLMVTSIRSTPNYI--------------
Nc  GTTTNDPLLDSLLMEEALLTHERYGTEYMDENPITGKPGDFHLTSTGRKTQTKSALTLKE
Ce  AEDALEREEKEEVEEEEEETLKTEEPTTSTDEPKFAEPTARTTSKQ--------------
At  SQEALGEKEELGTQGGESQPPQVDPIIDQGPAKRMKF-----------------------
Ca  TVSNGISTSTEIASGVFDTLLNDVVMNDDHLYIDEIPLYGEGSTLERLGLKGNKDAGLSL
Dm  PIPPMLQNLQQPPPAGDDLNTARNASEMNNATGPLDIKTEGVDMKPPPEKKSK-------
Gg  QKPGEKPIPVDQIKKEPEPAPEAVKTEEKETVKNAQQSAAAKGPPEKRMRLQ--------
Os  YVPSEQEKASDDLLASEALPPQVDPIIDQGPAKRPRFQ----------------------
Su  PKPGDRPVPVEGMSSESASARPVIKEEIKEENNQSQNNANITSTGSSSVPSMAASVTSHS
Bt  QKSGEKPVPVDQTKKEAEPLPETVKSEEKETAKNVQQTVGTKGPPEKRMRLQ--------
Cf  QKSGEKPVPVDQTKKEAEPIPETVKSEEKETTKNVQQIVSTKGPPEKRMRLQ--------
Hs  LKPGEKPVPVDQTKKEAEPIPETVKPEEKETTKNVQQTVSAKGPPEKRMRLQ--------
Pt  LKPGEKPVPVDQTKKEAEPVPETVKPEEKETTKNVQQTVSAKGPPEKRMRLHYIILSIKQ
Rn  QKSGEKPVPVDQAKKEAEPLPETVKSEEKESAKSAQQTVSTKGPPEKRMRLQ--------
Mm  QKSGEKPVPVDQAKKEAEPLPETVKSEEKESTKNIQQTVSTKGPPEKRMRLQ--------
Dd  ------------------------------------------------------------
Sp  SLHSSKEAPDVK------------------------------------------------

           430       440       450       460       470       480
Cp  ------------------------------------------------------------
Af  ------------------------------------------------------------
Sc  ------------------------------------------------------------
Nc  AAAALPALNTKGLGAAGSNPLAKGAAAAAAANANAVTGKETKSPKTPGGGGPPKPKRRKS
Ce  ------------------------------------------------------------
At  ------------------------------------------------------------
Ca  ------------------------------------------------------------
Dm  ------------------------------------------------------------
Gg  ------------------------------------------------------------
Os  ------------------------------------------------------------
Su  NTVTGQAAAAAAAGAAAAAAAAAGLSSSTNTTSSASGTFFTNTGPSNMKPSLAKKRKIMG
Bt  ------------------------------------------------------------
Cf  ------------------------------------------------------------
Hs  ------------------------------------------------------------
Pt  LLTFWKMKKRKNLTHQYHYPINTVTVL---------------------------------
Rn  ------------------------------------------------------------
Mm  ------------------------------------------------------------
Dd  ------------------------------------------------------------
Sp  ------------------------------------------------------------

           490
Cp  ----------
Af  ----------
Sc  ----------
Nc  KNVITTPGAA
Ce  ----------
At  ----------
Ca  ----------
Dm  ----------
Gg  ----------
Os  ----------
Su  K---------
Bt  ----------
Cf  ----------
Hs  ----------
Pt  ----------
Rn  ----------
Mm  ----------
Dd  ----------
Sp  ----------
```

Med7

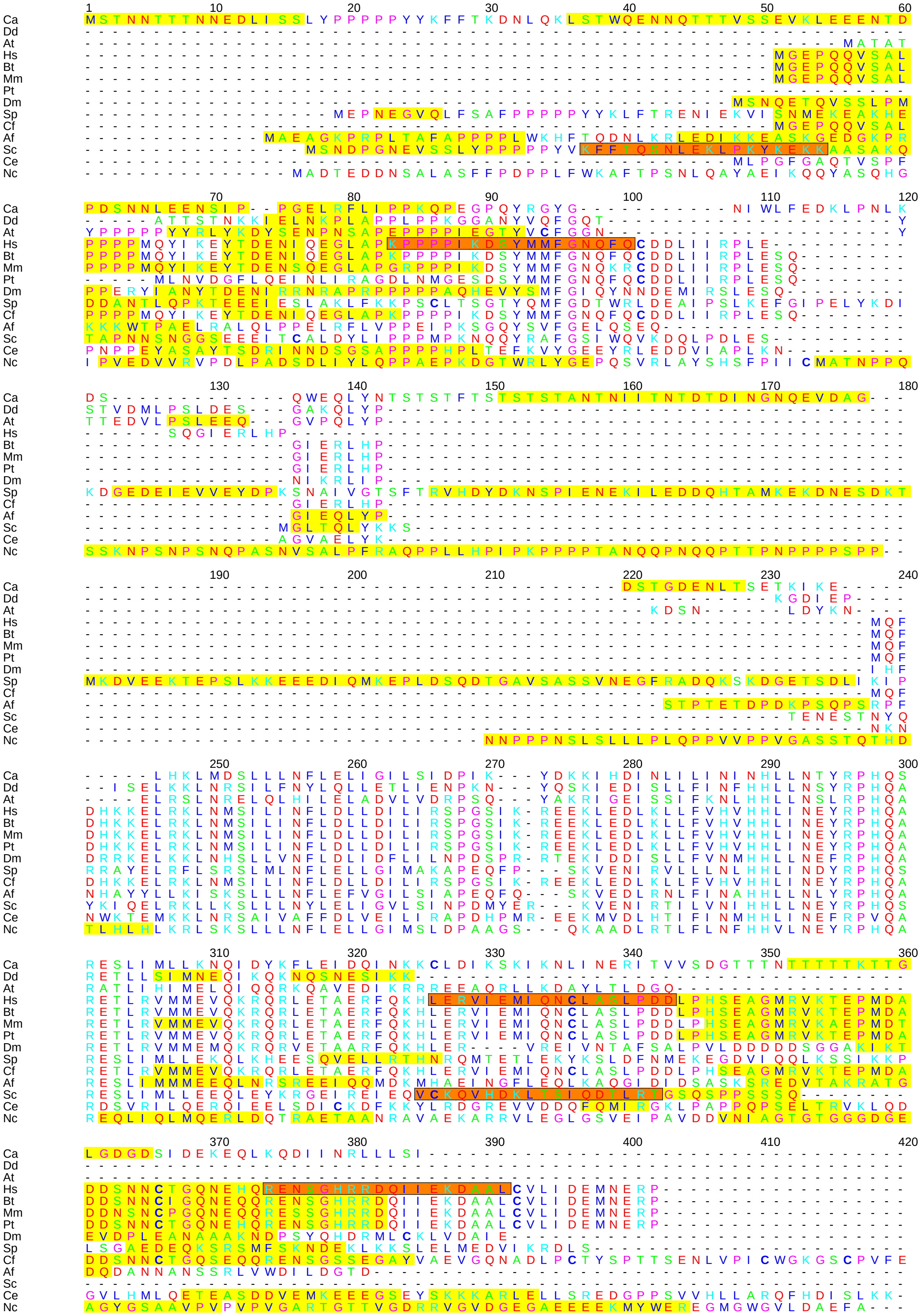

Ca
Dd
At
Hs
Bt
Mm
Pt
Dm
Sp
Cf
Af
Sc
Ce
Nc
1
10
20
30
40
50
60
70
80
90
100
110
120
130
140
150
160
170
180
190
200
210
220
230
240
250
260
270
280
290
300
310
320
330
340
350
360
370
380
390
400
410
420

Med7

```
                                  430       440       450       460       470       480
Ca   ------------------------------------------------------------
Dd   ------------------------------------------------------------
At   ------------------------------------------------------------
Hs   ------------------------------------------------------------
Bt   ------------------------------------------------------------
Mm   ------------------------------------------------------------
Pt   ------------------------------------------------------------
Dm   ------------------------------------------------------------
Sp   ------------------------------------------------------------
Cf   CHNTVLSTDGRNLKYQTSNRYRLMRNFHKGDVSLTIENVTLADSGTYCCRIQFPGLMNDK
Af   ------------------------------------------------------------
Sc   ------------------------------------------------------------
Ce   ------------------------------------------------------------
Nc   ------------------------------------------------------------

                                  490       500       510       520       530       540
Ca   ------------------------------------------------------------
Dd   ------------------------------------------------------------
At   ------------------------------------------------------------
Hs   ------------------------------------------------------------
Bt   ------------------------------------------------------------
Mm   ------------------------------------------------------------
Pt   ------------------------------------------------------------
Dm   ------------------------------------------------------------
Sp   ------------------------------------------------------------
Cf   KSNLELVIKPAKVIATWTPWRDFTAPFPLMLTTKGHGSETQTLVALHDKQTQIPTLANEL
Af   ------------------------------------------------------------
Sc   ------------------------------------------------------------
Ce   ------------------------------------------------------------
Nc   ------------------------------------------------------------

                                  550       560       570       580       590       600
Ca   ------------------------------------------------------------
Dd   ------------------------------------------------------------
At   ------------------------------------------------------------
Hs   ------------------------------------------------------------
Bt   ------------------------------------------------------------
Mm   ------------------------------------------------------------
Pt   ------------------------------------------------------------
Dm   ------------------------------------------------------------
Sp   ------------------------------------------------------------
Cf   EDAGATTRLGVYIGAGISAGLALIFIIGALILTWYSYSKEKLQNSSLVTLANPSPLGLAN
Af   ------------------------------------------------------------
Sc   ------------------------------------------------------------
Ce   ------------------------------------------------------------
Nc   ------------------------------------------------------------

                                  610       620       630
Ca   --------------------------------------
Dd   --------------------------------------
At   --------------------------------------
Hs   --------------------------------------
Bt   --------------------------------------
Mm   --------------------------------------
Pt   --------------------------------------
Dm   --------------------------------------
Sp   --------------------------------------
Cf   TGAEGMRSQENIYIIEENIYEMEDPYEYYCYVNSEQQS
Af   --------------------------------------
Sc   --------------------------------------
Ce   --------------------------------------
Nc   --------------------------------------
```

Med8

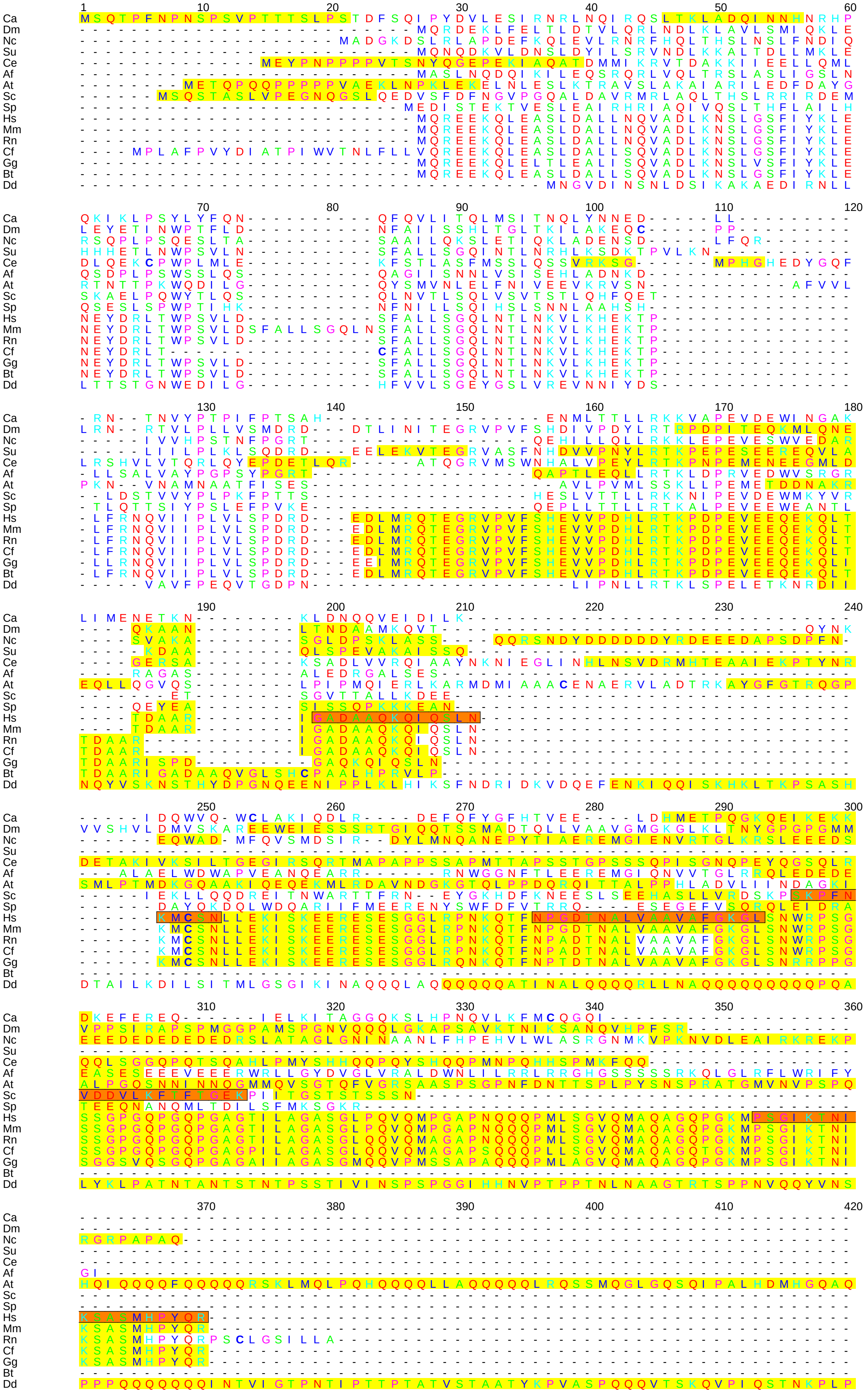

Med8

```
                    430       440       450       460       470       480
Ca  ------------------------------------------------------------
Dm  ------------------------------------------------------------
Nc  ------------------------------------------------------------
Su  ------------------------------------------------------------
Ce  ------------------------------------------------------------
Af  ------------------------------------------------------------
At  QKFQTSHGQHQMPYSQPMGAHQQFQARQLSGGHIQHSMSQGQLNPAMNRHLNQFSGGANS
Sc  ------------------------------------------------------------
Sp  ------------------------------------------------------------
Hs  ------------------------------------------------------------
Mm  ------------------------------------------------------------
Rn  ------------------------------------------------------------
Cf  ------------------------------------------------------------
Gg  ------------------------------------------------------------
Bt  ------------------------------------------------------------
Dd  QQQPSIQIQPQQSIVQMVQNVLPNTTSPPVNNNNQSPINSGIGGGQFKPSAINIANANSG

                    490       500       510       520       530       540
Ca  ------------------------------------------------------------
Dm  ------------------------------------------------------------
Nc  ------------------------------------------------------------
Su  ------------------------------------------------------------
Ce  ------------------------------------------------------------
Af  ------------------------------------------------------------
At  ALFTSAQGSPSSQMIPNMSSMQSQTLVPRMQQFGVSGTNPQRSHSSQMLGDQMFNTSGMM
Sc  ------------------------------------------------------------
Sp  ------------------------------------------------------------
Hs  ------------------------------------------------------------
Mm  ------------------------------------------------------------
Rn  ------------------------------------------------------------
Cf  ------------------------------------------------------------
Gg  ------------------------------------------------------------
Bt  ------------------------------------------------------------
Dd  TSTIQQQIQLQQQQLQQQIQLQQQLAQQQQQNQQNQQNQQNQQNQQNQQIHQHIQHLTPQ

                    550       560       570       580       590       600
Ca  ------------------------------------------------------------
Dm  ------------------------------------------------------------
Nc  ------------------------------------------------------------
Su  ------------------------------------------------------------
Ce  ------------------------------------------------------------
Af  ------------------------------------------------------------
At  QTQQTQIQQSQQQQQQQQQGGYGNMQTNQQSLQPNNMMQNAQQRHQNPQ-----------
Sc  ------------------------------------------------------------
Sp  ------------------------------------------------------------
Hs  ------------------------------------------------------------
Mm  ------------------------------------------------------------
Rn  ------------------------------------------------------------
Cf  ------------------------------------------------------------
Gg  ------------------------------------------------------------
Bt  ------------------------------------------------------------
Dd  QQAQLQQQMLQQQQLQQQFQQQQLSQQLLQQQQLHQQQQPNQTIQNPFHNFQANNFRVQN

                    610
Ca  ----------
Dm  ----------
Nc  ----------
Su  ----------
Ce  ----------
Af  ----------
At  ----------
Sc  ----------
Sp  ----------
Hs  ----------
Mm  ----------
Rn  ----------
Cf  ----------
Gg  ----------
Bt  ----------
Dd  PTNFNPPQNQ
```

Med9

```
1 10 20 30 40 50 60
Hs -MDVSGQETDWRSTAFRQKLVSQIEDAMRKAGVAHSKSSKDMESHVFLKAKTRDEYLSLV
Dm ------MTEDWQSQKFRQNVISKIHDLLPPNAQDQTKNAGVMENHIFRKSRTKDEYLGLV
Ce MSEEDWPSPKFREHVIQRLEPELARNRQNAPNLPVPGDARQVEEYVFAKCMSKDEYMRTI
Mm ------------------------------------------------------------

70 80 90 100 110 120
Hs ARLIIHFRDIHNKKSQASVSDPMNALQSLTGGPAAGAAGIGMP-PRGPGQSLGGMGSLGA
Dm AKLFMHYK--DMSRKSQQQQQQQQQQGGPPPNAEMGGGQNMMQDPLNALQNLASQGNRNP
Ce AKVINAIN--CNSKSAAVPSVLQPSQFHSPPCTTADPQPTSAQ-ARNPPVTVATTQASTT
Mm -----------MASSGVAGGRQAEDTLQPPP----ELLPESKPPPPPQPLPVAALPPPAA

130 140 150 160 170 180
Hs MGQPMSLSG-QPPPGTSGMAPHSMAVVSTATPQTQLQLQQVALQQQQQQQQFQQQQQAAL
Dm QMMPMGAGGGAPVPGGPGTASNLLQSLNQQRPGQQQMQPMSNIRGQMPMGAGGAGAQQMM
Ce PSAPNPPGG-LPAPSASATAAVAAAVASFPSPDTSIRPGGQITPGSQAPGGGPTPAPNVP
Mm PRPQSPAGAKEENYSFLPLVHNVIKCMDKDSPDLHQDLNALKTKFQELRKLIGTMPGIHV

190 200 210 220 230 240
Hs QQQQQQQQQQQFQAQQSAMQQQFQAVVQQQQQLQ-QQQQQQQHLIKLHHQNQQQIQQQQQ
Dm QVQQMQQGGNAPGVMNVMGAGGGQNQGQIVGNPGQQMGVGVGMPNQMVGPGPNSGPAVGG
Ce FPNGSSQMNGGPAMGQPPPQMGAPNMGGPPNGYGGYGMMNGPPGSGAPMGGNPYNQQIKK
Mm SPEQQQQQ-------LHSLREQVRTKNELLQKYKSLCMFEIPKD----------------

250 260 270 280 290 300
Hs QLQRIAQLQLQQQQQQQQQQQQQQALQAQPPIQQPPMQQPQPPPSQALPQQLQQMHHTQH
Dm AGGPNAAPGAGGPGPNQMQGGPMNVNAMQQMPPMQQIQQNQLGMGMNPMMRMGQGNGMGG
Ce DMDQARPWDPSAHMYQQQPQWGAMPPQQPHGYPGRPMNGQGATPTGPSSVLESLINQPQQ
Mm ------------------------------------------------------------

310 320 330 340 350 360
Hs HQPPPQPQQPPVAQNQPSQLPPQSQTQPLVSQAQALPGQMLYTQPPLKFVRAPMVVQQPP
Dm PQGMPGQGMQGMPQGPHNVVGGPAGQQQVGGAGLPPNAVQQGGMNPMGGMGVNMPPNLQQ
Ce YPGHHNQMGPPGDRNVAAQRAAAQQQQQQQQQQQQQRPGMVPNQGMMSSEDQTVYSAKLR
Mm ------------------------------------------------------------

370 380 390 400 410 420
Hs VQPQVQQQQTAVQTAQAAQMVAPGVQVSQSSLPMLSSPSPGQQVQTPQSMPPPPQPSPQP
Dm KPNMPMGQAGQMFPGNRGGVGVGGQQPGQPFMRSSPSPADAQQLQQQAQLQQMQQQQQQL
Ce NMRGSCDSLRTRARQCRHEGNHEAAHKLEVMLSVLEGKRVVSLEYLNHLEMWIARKQDFL
Mm ------------------------------------------------------------

430 440 450 460 470 480
Hs GQPSSQPNSNVSSGPAPSPSSFLPSPSPQPSQSPVTARTPQNFSVPSPGPLNTPVNPSSV
Dm VVGNQTPTQQPPTPQMPTPNMIPSPALVPQSSPQMMQMQNSQRNIRQQSPSASINTPGQV
Ce NIAPMSQNQNHMGMNDPMMNGEHAMLGNGQVPNPYGGHPGYGHQQYMGPPPPHMQMHQPP
Mm ------------------------------------------------------------

490 500 510 520 530 540
Hs MSPAGSSQAEEQQYLDKLKQLSKYIEPLRRMINKIDKNEDRKKDLSKMKSLLDILTDPSK
Dm TGNSPFNPQEEALYREKYKQLTKYIEPLKRMLAKISNDGTNVEKMTKMSKLLEILCNPTQ
Ce MWHQQQHQQQQRMMPQDHMMMQGGGGPVHGMYRGDMGHDPMTSPVNNHRHAPYPNPAAMR
Mm ------------------------------------------------------------

550 560 570 580 590 600
Hs RCPLKTLQKCEIALEKLKNDMAVPTPPPPPVPPTKQQYLCQPLLDAVLANIRSPVFNHSL
Dm RVPLETLLKCEKALEKMDLISYSGQQFGKSS---------NPLLEVINTTLQSPVANHTL
Ce NNMRMPNGPGPIGRDRNSMSGSSMSGPSSGAPSMNPMGTPNQKMGTPGSMGGMSGLDDLN
Mm ------------------------------------------------------------

610 620 630 640 650 660
Hs YRTFVPAMTAIHGPPITAPVVCTRKRRLEDDERQSIPSVLQGEVARLDPKFLVNLDPS-H
Dm YRTFRPTLELLFGTDITAPVPAKRPRVEEKSTSFEQEVPHVLQGEIARLDTKFKVKLDTT
Ce YDDFLPNPTPIDALQPTLHVGQNSMNAGPPVQRSNLNETARKELQILDARF--EIDPN-H
Mm ------------------------------------------------------------

670 680 690 700 710 720
Hs CSNNG-TVHLICKLDDKDLPSVPPLELSVPADYPAQSPLWIDRQWQYDANPFLQSVHRCM
Dm SQINNKAIRLICCLDDKRLPSVPPVSVSVPEEYPWQAPDCSLAEQEYSATPFLQTVQQAL
Ce QRHDANHIIVVCKIRNQQFP---PLRLVVPTTYPAGN--VTVDRAVIDLDAYLYDDLQNS
Mm ------------------------------------------------------------

730 740 750 760 770
Hs TSRLLQLPDKHSVTALLNTWAQSVHQACLSAA--------------------
Dm IARISKLPKNYSLSHLLDTWEMAVRQACSPQSKPRAVCELSTLLGV------
Ce VYERLSRPGLSSITDYLNAWEEQVNQYQNQTSGGLDVAFNVGNDFFYDNLNL
Mm ----------------------------------------------------
```

Med21

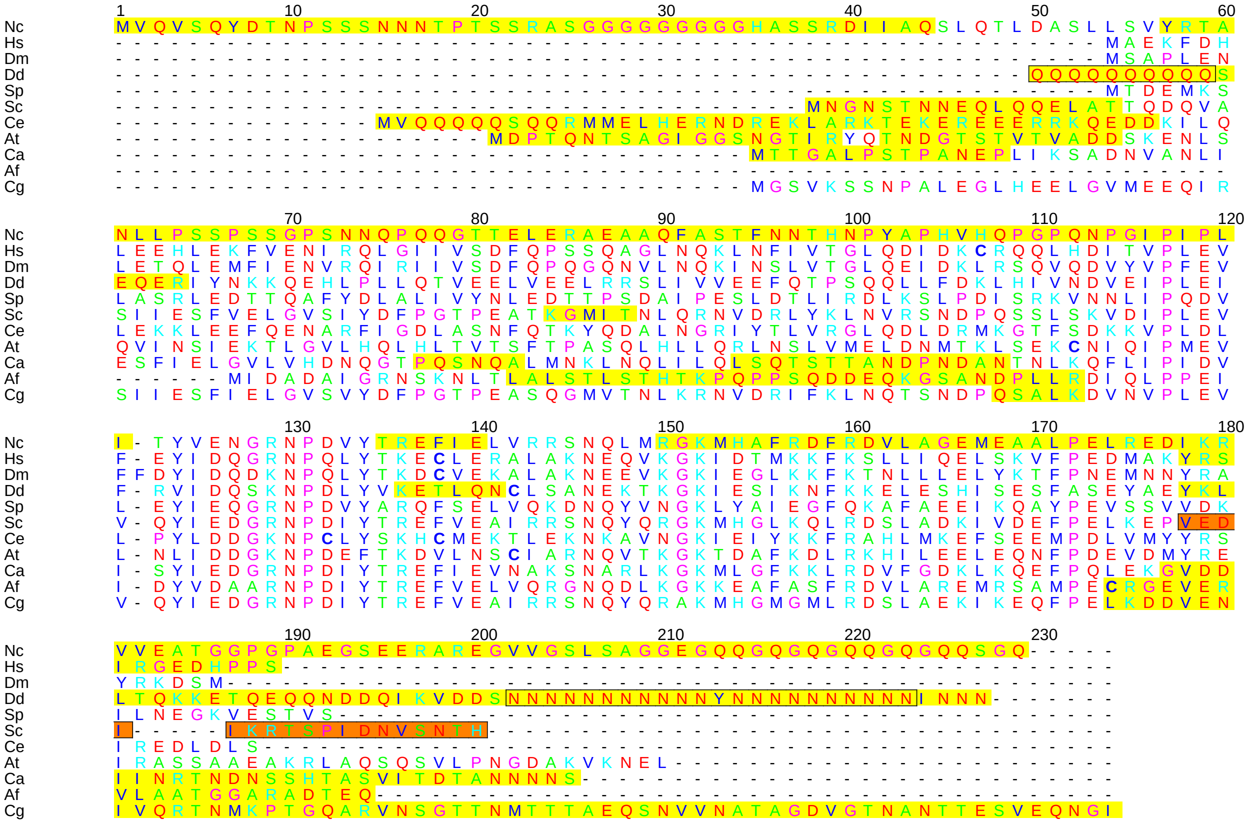

Med11_1

```
    1        10        20        30        40        50        60
Dm  -----------------------------------MLAISQQNPHKEINIVQLSRLGQET
Hs  --------------------MSTPPLAASGMAPGPFAGPQAQQAAREVNTASLCRIGQET
At  MLQKQVSTTTTMTTQELAMEGEKQLEETIEAAFQIISAMNDELCNPSLWSTSATPSSAAT

             70        80        90        100       110       120
Dm  VQDIASRFQEVFASLKGIQPTS-------------HRENSSEKKVQEYFRTIRLLFKRVR
Hs  VQDIVYRTMEIFQLLRNMQLPNGVTYHTGTYQDRLTKLQDNLRQLSVLFRKLRLVYDKCN
At  TTGSNGSALVSADAAAIDGTSH-------------HSESAGGGGGGGSGNSVLDEASLRY

             130       140       150       160       170       180
Dm  IIYEKCNDAGMDYMSAESLIPYRDEPEPRIEPSLCDEYRKVLQENHELIETVKLKNRQLR
Hs  ENCGGMDPIPVEQLIPYVEEDGSKNDDRAGPPRFASEERR---EIAEVNKKLKQKNQQLK
At  KNSVTSLRAVLAAIPNSQKAKASEMQNGLGSPESEDEIEKLEEQALSLRMEIAKKNVHVK

             190       200
Dm  EIIDRTRIIIWEINTMLAMRRS
Hs  QIMDQLRNLIWDINAMLAMRN-
At  ELIDKLRELIADISTWQSPCSV
```

Med11_2

```
    1                 10                  20                  30                  40                  50                  60
Sc  M Q V L N T K S E T K Q E N E T M Q P P Y I Q E R L K S L N D I E T Q L C S M L Q E A S Q V T F I F G E L K R G N E S V
Ca  - - - - - - - - - - - - - - - M S S L D N D K T E N F I Q E R L D S L H E I D C K V V T L L D Q F S S I F Q S F Y T K S

                      70                  80                  90                  100                 110                 120
Sc  K P Q F E N H V K Q F Y E R L D K S T T Q L R K E I Q L L D E N V G T - - - - - - - - - R L L P I - N V N K K A L G Q D T
Ca  K E D F S Q Q T S D I Y S T L S K V A I D L R K E I K I M D D N I G A Y D K N D D N V M I L P I S N V D Q K N T K L G R

                      130                 140                 150                 160                 170                 180
Sc  E K M E E Q L D L L S A I L D P S K S K - - - - - - - - - - - - - - - - - - - - - - - - - - - - - - - - - - - - - - - -
Ca  K R L D L E L A E L K R L I S D E K Q V E T L E N E S N N E I Q P K T E S D T N Q V E T N E N G N D I N N K E S E D I E

Sc  - - -
Ca  M K E
```

Med12

```
    1                10                  20                  30                  40                  50                  60
Nc  ------------------------------------------------------------
Af  ------------------------------------------------------------
At  ------------------------------------------------------------
Dd  ------------------------------------------------------------
Sc  ------------------------------------------------------------
Ca  --------------------------------------------MSNKNRNRNSLLSSHN
Su  ------------------------------------------------------------
Dm  ------------------------------------------------------------
Sp  ------------------------------------------------------------
Hs  ------------------------------------------------------------
Mm  ------------------------------------------------------------
Cf  ------------------------------------------------------------
Bt  ------------------------------------------------------------
Ce  ------------------------------------------------------------

    70 80 90 100 110 120
Nc  ------------------------------------------------------------
Af  ------------------------------------------------------------
At  -----------------------------------------------------MQRLGPP
Dd  ------------------------------------------------------------
Sc  ------------------------------------------------------------
Ca  RSSHTNSTSKDELLKLKYLMETPNLPIYPLNDSSIINSKTNDNLKNSPYQKPKSQSQPQA
Su  ------------------------------------------------------------
Dm  ------------------------------------------------------------
Sp  ------------------------------------------------------------
Hs  ---------------------------------------------------------MKQ
Mm  ------------------------------------------------------------
Cf  ------------------------------------------------------------
Bt  ------------------------------------------------------------
Ce  ------------------------------------------------------------

    130 140 150 160 170 180
Nc  ---------------------NYGCADFFPWSGTAGNHPEDQFNDTAIRVGYSEKQFVTP
Af  ----------------------------------MGNHPEDVLNEQTAKQGYYDRTQVSQ
At  DFHPPTSNSPEENLTKEYVQFGYKETVDGLKESEEIILSQVHTFSKPVVHKCKEAVRKCL
Dd  ----MRGGGLKKYEIRKPADKKELGPDLGVPDFYPLDDKSKEDTLPQEFILGGFCERYQH
Sc  -MNNGSGRYLLTPPDDLHPYVPSSKPQEQVYPDFKPWEHTAAEDQILANFVAKGFYHTPM
Ca  QGQSQSQPENSLVYPDFMPWKDHTQLPQDETEIEHQKLSNVSYLNKGYFEPPQVANEYYS
Su  ------------------------------------------------------------
Dm  ----------------------MLSMLQEKRPLKRTRLGPPDIYPQDAKQREDELTPTNV
Sp  -MSNRDSANPPGFNDAVPLAICYIDSSTSSHDEYLTLLQSKVSAPDKNFRDETVDGAPFL
Hs  SMPSLHTKKILFCYFHLTNSWCLRRYGLGKMAAFGILSYEHRPLKRPRPRLGPPDVYPQD
Mm  ------------------------------------------------------------
Cf  ------------------------------------------------------------
Bt  --------------------------------MAAFGILSYEHRPLKRPRLGPPDVYPQD
Ce  -----------------MLRKTNLRMGTADVYKQNKNQEEDMLTTERLKKGYQMPLTQYE

    190 200 210 220 230 240
Nc  ETQSAKTVLFHGLKQ-RSGLNALSIFFAGVLWHR--RNAGQVTAPSTFKP----------
Af  NESNTARPSLYAQLKHRSGLQILSSVFAAALEKR--QGHNMVTAPSTFKP----------
At  RAINESRALKRKVIKRLSDSNPFQFIFCAEFRAG--QVYGVPLSGSLLCKPG--------
Dd  DECKSSSSIFKNFDFPKVKEQLLQSIYQVEWKKS--STQKSGQPPVMN--------KSGN
Sc  VNFESISARSSVHESLVTQSNILSQQFDKIIKIR-EDHINKIPSNSTTTLHG-------P
Ca  ARNLIQATVFLSTDNCTNVINELSIHLSNAYKSR-NEIINKIKFDSNK------------
Su  ----------------------MPMIRAAWYLKMMAAN----------------------
Dm  KHGFTTTPPLSDEFGTAHNSNVNASKVSAFFSGVLAKKEELMTLPDTGRK----------
Sp  AGDAISKGHRYSPPVRSDRASAQGLLQQHLEKSSTLENLKRYSSTLLGRQNDAVIDEPFA
Hs  PKQKEDELTALNVKQGFNNQPAVSGDEHGSAKNVSFNPAKISSNFSSIIAEKLRCNTLPD
Mm  ------------------------------------------------------------
Cf  ------------------------------------------------------------
Bt  PKQKEDELTALNVKQGFNNQPAVSGDEHGTAKNVNFNPAKISSNFNSIITEKLRCNTLPD
Ce  LQSLVFNASENKLIRLEKAQKRSQSMIASVIHKKGEFHMNYDRERKIP------------

    250 260 270 280 290 300
Nc  ----PPRVTLTDT----------KKEVWLKDLANS---------AIPLRKLSRSIPHGVR
Af  ----PPRVTLTDN----------KREAWLRDLANP---------NVPLRKLSRTIPHGIR
At  ---FPEQRSCGEET----------KKRWIESLSQQ---------HKRLRSLADNIP-GYR
Dd  KRPVPEQKAWSAN----------HRESWVLKLAGN----------EPLQKLASSVPHGYK
Sc  GFQLPNRITLTDHR----------KETWLHELSSS---------HTSLVKIGKFIPHGLK
Ca  -FKLPTRVTLTLSK----------KEIWLNELSNP---------NVSMTKLGEKIPHGIR
Su  ------QAAIQET---------KMKKRQPNDPSQAN-------------KSKGQVPVFHR
Dm  ----KQQINCKDNFWPVSPRRKCTVDAWFKDLAGN----------KPLLSLAKRAPSFNK
Sp  PFRPPPRVTLSESR----------RDRWLQGLSDP---------MVPLSSLIKTIPHGLW
Hs  TGRRKPQVNQKDNFWLVTARSQSAINTWFTDLAGT----------KPLTQLAKKVPIFSK
Mm  -------MNQKDNFWLVTARSQSAINTWFTDLAGT----------KPLTHLAKKVPIFSK
Cf  ------------------------------------------------------------
Bt  IGRRKPQVNQKDNFWLVTARSQSAINTWFTDLAGT----------KPLTQLAKKVPIFSK
Ce  ----KEQLPCFAP------------HNWKRILSDRVKFFSALARGTPLQQLSQRIPGFRR

    310 320 330 340 350 360
Nc  NKVLLDQCLNKNVPIDRAVWLAK---CVG----------TNEL---RTFRRLGKGNNASV
Af  GRVLLDQCLTKWVPVGRAVWLAKCVG-------------ANEIR---AFKRKGTSGALTI
At  RKTLFEVLIRNNVPLLRATWFIKVTYLNQVHCWAINWCDLVLLLFLQEHLTRHKLLGVSN
Dd  GETLLKMFLDNQVPLIRATWYTK---IVYG------------------------------
Sc  RRQVIEQCYLKFIPLKRAIWLIK---CCY----------FIEWKSNHKKKRSNAAGADDA
Ca  NKVLIDCICTKKIPINRSIWFTKCVLYGELLALRRKHQNRLSTIPNNNSNTGGGGGGGGS
Su  KEDIFMTLCENNMPMIRAAWYLK----------------MMAANQAAIQETKMKKRQPND
Dm  KEEIFITLCENQVNMQRATWFIK-----------LSAAYTLSFTESKNKKRSIYDPAAEW
Sp  GEDILRMLVKFRVPFTRAIWFIR---CAG----------------VNEARSFLRKVQTTD
Hs  KEEVFGYLAKYTVPVMRAAWLIKMTCAYY-----------AAISETKVKKRHVDPFMEWT
Mm  KEEVFGYLAKYTVPVMRAAWLIKMTCAYYAAMS-------------ETKVKKKNTADPFT
Cf  ---------------MRAAWLIKMTCAYYAAIT-------------ETKVKKRHVIDPFM
Bt  KEEVFGYLAKYTVPVMRAAWLIKMTCAYY-----------AAISETKVKKRHIDPFTEWT
Ce  KELLFLEFSDYNISYDRAIWCVKLLCILGFNSSSKTQKKTLLDVATLELSQACNKTTIWL

    370 380 390 400 410 420
Nc  LE----------------------------------------GERKWLKDWTLVIENFIE
Af  G-----------------------------------------LEAKWVRDWTANVQQFLE
At  GQKMLLNICNTSWMNFCHGIAHFLLSKLEIGHHRCFIQDQCKRIVQHQQAFTARKHLYIL
Dd  -----------------------------------------NMVFEPSNDWTKTIVQS--
Sc  ------------------------------------------ISMHLLKDWTDTFVYILE
Ca  GNSLTGMMDYN---------------------------TAEKFEIHWLQEWTQQVSDYIY
Su  PSQDWC-----------------------------------LIITKFLKSQLDKIVEYNN
Dm  TG----------------------------------------NMIKFMKELLPKLQEYYQ
Sp  -------------------------------------------ITEWVKNWTDVAAGFLI
Hs  -----------------------------------------QIITKYLWEQLQKMAEY--
Mm  EWT--------------------------------------QIITKYLWEQLQKMAEY--
Cf  EW--------------------------------------TQIITKYLWEQLQKMAEY--
Bt  -----------------------------------------QIITKCLWEQLQKMAEY--
Ce  VTQLYKSFRNPIKRREATKTWNYLTG-----FMKYMFDDGVLDKQEFLNDLTEVFNQLFL
```

Med12

```
                      430       440       450       460       470       480
Nc  R--------------------------------VFFSFGE--------------------
Af  G--------------------------------VITSCGVADWK----------------
At  NG----------GIWCVFYSGTMLKGFFFLISLLIGFSSSYSFVNFHFALLQEKEIFEIL
Dd  ----------------------LFALIR-------NTTGENNSSGSSSSNNVLGGNSGSG
Sc  K------------------------LIFDMTNHYNDSQQLRTWKR---------------
Ca  KF----------SREMNN---------------IANLDGKNQYMG---------------
Su  TA----------SLVSAQQAASANTKQSSSSTTSSMLGGGMGSMG---------------
Dm  QN----------HDKSSSNGTTSGSLTAAGNGPASNGSTGTSSINSVTGSSASTNVIPVP
Sp  S--------------------------------FISSFLNADIYS---------------
Hs  ---------------YRPGPAGSGGCGSTIGPLPHDVEVAIR------------------
Mm  ---------------YRPGPAGSGGCGSTIGPLPHDVEMAIR------------------
Cf  ---------------YRPGPTGSGGCGSTIGPLPHDIEVAIR------------------
Bt  ---------------YRPGPAGSGGCGSTIGPLPHDVEMAIR------------------
Ce  QKNFKNPSVLSTFMKYYVYFVEDLSSSLVLARRSSTLLCQALGFLFEMAQDKRKIDAYYD

                      490       500       510       520       530       540
Nc  --QNWK---------------------------------------------------AKV
Af  ---------------------------------------------------------MKV
At  QLLLPIVYGVLESIVLSQTYVQSLVAIAVRFIQEPAPGGSDLVDNSRRAYTLSALIEMVR
Dd  VVKKATSS------------------------------------------------YTGI
Sc  ----------------------------------------------------------QI
Ca  ----------------------------------------------------------KL
Su  -----MGSGTMGGSTIELEK-----------------------------------CVKLW
Dm  SMASPLPPIHSPANGQQAAPGGGVNAGSVMPTTGSLGGVVGGPGSSVVGGAAGAGAAVPP
Sp  -------------------------------------------------------FADDY
Hs  ----------------------------------------------------------QW
Mm  ----------------------------------------------------------QW
Cf  ----------------------------------------------------------QW
Bt  ----------------------------------------------------------QW
Ce  SANKTFIKKEFENEDGEIYEFEDYDDFIDMLDEIPDVPYFDETDKTPEAQAEELIKQHEK

                      550       560       570       580       590       600
Nc  NYAIQLAAHLYAEG---------------------LMDREHYLDWTVSSLES--------
Af  TYAVNLTSRLFFEQ---------------------LLDHDQYLEWFLTSLEA--------
At  YLVLAAPDTFVASDF----------FPLPPSVAACGPNDVSYTSKAYENLEKLRSNSAEI
Dd  LYIERLIKWNFSEG---------------------LLNKDILLSMLIDHLN---------
Sc  SYFLKLLGNCYSLR---------------------LINKEIFHHWLVEFIN--------K
Ca  SYLLNYIQSLYVES---------------------LLDKSLFLSLIMKFLKDG----LCL
Su  QYTTELARYLYQEG---------------------MLECHDYLSWLVDTLEKVN------
Dm  GSTISGIGSQFEDSRNALKYWKYCHQLSKYMYEESLLDRQEFLNWILDLLDKMRTQASFD
Sp  TYLLKLFGRLLAEELVS---------------------PKHFLLRIVSFSGD--------
Hs  DYTEKLAMFMFQDG---------------------MLDRHEFLTWVLECFEKIRPGEDEL
Mm  DYNEKLALFMFQDG---------------------MLDRHEFLTWVLECFEKIRPGEDEL
Cf  DYNEKLAMFMFQDG---------------------MLDRHEFLTWVLECFEKIRPGEDEL
Bt  DYNEKLAMFMFQDG---------------------MLDRHEFLTWVLECFEKIRPGEDEL
Ce  RKAGSLKKSERRRRRGLSKNRPKKMPQNPSNEASLDHDKVQIKQEPMDYDEYGGQQSDNS

                      610       620       630       640       650       660
Nc  -SHHS----QLPMWIIIAQIYW----KDLLRLRKYG-----------------RRLATA-
Af  -APFN----TLPVWLLMLGIYWS----NILRYRKRG-----------------RRLAEL-
At  SAQFQGRGVLSRFEFLSFDYTISTIQRSADDLAKIASAGYPQHNVAKAVQALDKALSDGD
Dd  ETDRP----EEAVLISSLLLYYSDTLILSTYHSMKFLN----------------------
Sc  MENFEF----LPLSLHILMIFWN----DICQIDTNAPVAATITSSQKEPFFLVTKITDML
Ca  QSKYINGLIYSSMEENSDDYNDDDDDDDDEEDANNAENPESTSKSKKRNLAWMDEIDMNY
Su  TNDDSTLRFLLPFVVQYLEEFLKSQMLSRRLAYVCARRLMSMCSETRPHSPHLNTSSNVQ
Dm  EPLKKLVLSFALQYMHDFVQSERLCRKMAYIVSKKLAQLLNTVVEQQTIKELDEPKLQQD
Sp  -SSLKS----FSLHFFALQFFS----TSLIQYTHICR-----------------KCVIT-
Hs  LKLLLPLLLRYSGEFVQSAYLSRRLAYFCTRRLALQLDGVSSHSSHVISAQSTSTLPTTP
Mm  LKLLLPLLLRYSGEFVQSAYLSRRLAYFCTRRLALQLDGVSSHSSHVIAAQSTSSLPTTP
Cf  LKLLLPLLLRYSGEFVQSAYLSRRLAYFCTRRLALQLDGMSTHSSHVISAQSTSTLPTTP
Bt  LKLLLPLLLRYSGEFVQSAYLSRRLAYFCTRRLALQLDGVSSHSSHVMSAQSTSTLPTTP
Ce  MDYDSFSHQYDPSYEPLPFKPLHTDIPKKLTDDEEFKYMKIGKRSVTPERISGRYGSMTY

                      670       680       690       700       710       720
Nc  ---------------------------------------LISHYHVIHAHEDRDIYVP--
Af  -------------------------------------------------LLDKLQLAIKS
At  IRAAYSYLFEDLCNGAVDEAWITDVSPCLRSSLRWIGAISTSFVCSVFFLIEWATCDFRD
Dd  ------------------------------------------------------------
Sc  LHK-----------------YYIVSSSKSMINDENYIINDIK-------KNNKIKLN---
Ca  GQRLVSLTLIKMFWNDILKFDYLSKELSELLLLNYFFINKLLLFSVNNDNGNSTAVIPDG
Su  LPPN--------------------------------------------------------
Dm  PYELALQEQMSCPHHRDIVLYLSTILQIITIECPTALVWSGIAAHRAPSSLLGSPLDHLP
Sp  ------------------------------------------------ILQSYQQLIVDQ
Hs  APQPPTSSTPSTPFSDLLMCPQHRPLVFGLSCILQTILLCCPSALVWHYSLTDSRIKTGS
Mm  APQPPTSSTPSTPFSDLLMCPQHRPLVFGLSCILQTILLCCPSALVWHYSLTDSRIKTGS
Cf  APQPPTSSTPSTPFSDLLMCPQHRPLVFGLSCILQTILLCCPSALVWHYSLTDSRIKTGS
Bt  APQPPSSSTPSTPFSDLLMCPQHRPLVFGLSCILQTILLCCPSALVWHYSLTDSRIKTGS
Ce  PFWIQQGGVGIDPPKDEVLDFSNPTPTETVNTQPQIDFESSPSASPALSVDKENECEKKE

                      730       740       750       760       770       780
Nc  --------LLSKLS-HLLGTLMVATPENFVSP-SVWVKYRDALK---------TCLPQGD
Af  DSAPSLRPLIDRLS-LHIRKLTLEHTSSMVLPQS-WEKYKDLLS---------SCLNLND
At  FRAGVPKDIKFSGRKDCSQVYLVIQLLKQKILGGEFTARKGKNCRNNFLGVSKPSGSMDA
Dd  ------KAYEKLMKITISSAAAVSAASSSSSSSSTQSNITALSNPKLQQLGHGHGHGHGH
Sc  --------ILKILSSLILKIFQEQSLEVFIFPTSNWEIYKPLLFEIVSN----ADTNQNS
Ca  LQSKILKLISDCVRYLFEYNCNIFIIPNHWTIGIGLNIVMKVLILNLKSELNNSNNTNNS
Su  ------TPPHSQHALASILAEFRSCPQHHSIILGLSCVLQAITLRCPSALVWINLGESKN
Dm  LAPSVLPMPTRCPRTNHEIRRQLRAAESDIVLRTQHAEQRWFAAKWLSAGKNQYTSVLAT
Sp  PANLLKFSLLSKKVSHFLFTLAQKNIESFFFPTEWDKLKPTIILLWKDFPN---------
Hs  PLDHLPIAPSNLPMPEGNSAFTQQVRAKLREIEQQIKERGQAVEVRWSFDKCQEATAGFT
Mm  PLDHLPIAPSNLPMPEGNSAFTQQVRAKLREIEQQIKERGQAVEVRWSFDKCQEATAGFT
Cf  PLDHLPIAPSNLPMPEGNSAFTQQVRAKLREIEQQIKERGQAVEVRWSFDKCQEATAGFT
Bt  PLDHLPIAPSNLPMPEGNSAFTQQVRAKLREIEQQIKERGQAVEVRWSFDKCQEATAGFT
Ce  DESKTKEKNKDKEKDKEKEKSVDEHTNDLDVPINPDDAEMADANDKTDASEKQKLVEEEP

                      790       800       810       820       830       840
Nc  EAR--------------HRSFAGINYRNEQ-----LVASANRSQ----------PAARII
Af  NVH--------------RTVFQILAERNARVQKPPKCEGTTQQP------------PQQR
At  FESPGPLHDIIVCWIDQHEVHKGGAKRLQLLVFELIRSGIFNPIAYVRQLIVSGMIDVIQ
Dd  HSHSHSHNQQQPQLNITSTQTIPSINSIASSLSPSNQKIYSILCIISKQISMNLPDTFMS
Sc  DMK---------------KKLELISYRNESLKNNSSIRNVIMSAS--------------N
Ca  SNLGTSKRSVRSGSFSNYTTTPIKPISNTTIEQDYSFINRNSDDVLNIVEKLDNLKLNKE
Su  YSVPGSPLDILPCPPSLLPLPGSVSMQNHQIRSSIRQAENQIKMRSRRAEVRWSSDKCQE
Dm  LDHLDTHCFDRMEHNNSIDTLYAQIFPSPTVSRRREEDQVEPRPPYEPKQDKDTVRILCE
Sp  ----------------YSTLLSIMQERNSK---------------------AMYMYKPVT
Hs  IGRVLHTLEVLDSHSFERSDFSNSLDSLCNRIFGLGPSKDGHEISSDDDAVVSLLCEWAV
Mm  IGRVLHTLEVLDSHSFERSDFSNSLDSLCNRIFGLGPSKDGHEISSDDDAVVSLLCEWAV
Cf  IGRVLHTLEVLDSHSFERSDFSNSLDSLCNRIFGLGPSKDGHEISSDDDAVVSLLCEWAV
Bt  IGRVLHTLEVLDSHSFERSDFSNSLDSLCNRIFGLGPSKDGHEISSDDDAVVSLLCEWAV
Ce  TGKENEDDTSSKTAKTSTSAEKSEAPSIVDSNDKIDKEPNASSTSNDETSKDDTVPMESD
```

Med12

```
           850       860       870       880       890       900
Nc  LVRNLDQVILEKPMPDEFPAQCWTVSKDK-------------------------AALVRA
Af  VIQLFDSICSSHDITSVSAASLRAIDDK--------------------------AALVLK
At  PAVDPERRMRHHRILKQLPGCFVHETLEEAQLFGGDKLSEAVRTYSNERRLLLRELLVEK
Dd  FKFFRELVSLIWPSNLIDRIEYNQLSF---------------------------------
Sc  ANDFQLTIVTCKQFPKLSCIQLNCIDTQFTKLLDDNPTEFDWPTYVDQNPLTMHKIIQLI
Ca  FASFLKPTKKCNANWKLNLKVLLFWCVSPWRNSTNSNEDILFICNFLKNTVVKSLPKSLR
Su  SAAGHTISRVLQTLEVLDRHCFDRCDASNSIDALYNKIFLNSQKQEAPEPLQSDDPIVLL
Dm  WAVSGQRWGEHRAMVVAILLDKRQIDVTSTPADQQSSDKDDKDSLASGAGLIDGLPVFQH
Sp  SSIRFLQIISCLSFPVGWRTLAKDLFKLLP------------------------------
Hs  SCKRSGRHRAMVVAKLLEKRQAEIEAERCGESEAADEKGSIASGSLSAPSAPIFQDVLLQ
Mm  SCKRSGRHRAMVVAKLLEKRQAEIEAERCGESEAADEKGSVASGSLSAPSAPIFQDVLLQ
Cf  SCKRSGRHRAMVVAKLLEKRQAEIEAERCGESEAADEKGSIASGSLSAPSAPIFQDVLLQ
Bt  SCKRSGRHRAMVVAKLLEKRQAEIEAERCGESEAADEKGSIASGSLSAPSAPIFQDVLLQ
Ce  PPAATEKPKESTEITTEEPLEVDKAPEVDKSEKEHEDDIMIIESNKKADEDDCVVIAVVD

           910       920       930       940       950       960
Nc  LLEWCTTPYRPGLAKVYVAHRILMHWS---------------------------------
Af  LLEWAATPFRYGVSRVYTGARLLRKWKIAG------------------------------
At  GKYWNNLVLSDQKSKKISTSLSSVIFPRACNAKSNSKGPRKHTKSSVDIRELKERISALL
Dd  ------------------------------------------------------------
Sc  L--WSIHPSRQFDHYESNQLVAKLLLLRINS-----------------------------
Ca  TEFDNELLEIIYHIAEDNNSEEGDNLQFTSSR----------------------------
Su  LCQWAVCPER--------------------------------------------------
Dm  VLMHFLDHDAPVLDEHVSSPQQRTEFTNLVQLFSALIRHDVFSHNAYMHTLISRGDLLLE
Sp  ----------------------VYTGVPLLLH-------WCINCRSIFSGDRNFIVSSIF
Hs  FLDTQAPMLTDPRSESERVEFFNLVLLFCELIRHDVFSHNMYTCTLISRGDLAFGAPGPR
Mm  FLDTQAPMLTDPRSESERVEFFNLVLLFCELIRHDVFSHNMYTCTLISRGDLAFGAPGPR
Cf  FLDTQAPMLTDPRSESERVEFFNLVLLFCELIRHDVFSHNMYTCTLISRGDLAFGAPGPR
Bt  FLDTQAPMLTDPRSESERVEFFNLVLLFCELIRHDVFSHNMYTCTLISRGDLAFGAPGPR
Ce  PDQEQTPESEKKKDGEEERDKNKDTDVADNEPEKIYTDEELAEIKIRKEKEMKIATLASH

           970       980       990      1000      1010      1020
Nc  -----TLRLDVTSAVLGFMEGEALEEMECKD-----------------------------
Af  --------VDVDTCIISFLGESQMSDQLNMDN----------------------------
At  QFPGMSCGVETPVRDEFQNSVKRSSGSVYSKMDQPEATPGCEDCRRAKRPKMNDEKSSCY
Dd  ------------------------------------------------------------
Sc  ---------------------------------------------------------TDE
Ca  ------------------------------------------------------------
Su  ------------------------------------------------------------
Dm  SVLVIKSGTTATKTSPPPPAPPPTTTHGFDDDGFGGGLDFKHNEFDDSNVDDDLDKLVQN
Sp  DNANFDRNLIVDLTLSFVLKLHPLEYNECVA-----------------------------
Hs  PPSPFDDPADDPEHKEAEGSSSSKLEDPGLSESMDIDPSSSVLFEDMEKPDFSLFSPTMP
Mm  PPSPFDDPTDDPERKEAEGSSSSKLEDPGLSESMDIDPSSTVLFEDMEKPDFSLFSPTMP
Cf  PPSPFDDPADDSERKETEGSSSSKLEDPGLSESMDIDPSSSVLFEDMEKPDFSLFSPTMP
Bt  PPSPFDDPADDPERKEAEGSSSSKLEDPGLSESMDIDPSSSVLFEDMEKPDFSLFSPTMP
Ce  IKEKMVSDKKWKEKTSHAGWRTLDQCSQFSEALHLLSSMVQYMACVTPESFVWNDLSVQQ

          1030      1040      1050      1060      1070      1080
Nc  ------------------------------------------------ALYHLVSELVRS
Af  -------------------------------------------------VYHIVSELVRS
At  QGNSPIASDEEDNWWIKKGSKTVESSLKVDPQIEITKQVPRGRQKMARKTQSLAQLQAAR
Dd  ------------------------------------------------------------
Sc  DLHEFQIEDAIWSLVFQLAKNFSAQKRVVSYMMPS--------------LYRLLNILITY
Ca  -------------------------------------------------LYVLINELYQL
Su  -------------------------------------------------TSEHRALVVAQ
Dm  IKEKGQQHEAPDSPKIGPPGDGETNPGGSISRHYVYTKHFPIPQDDPSMSSYSSESNQRY
Sp  -------------------------------------------------AAQLLDHLAAC
Hs  CEGKGSPSPEKPDVEKEVKPPPKEKIEGTLGVLYDQPRHVQYATHFPIPQEESCSHECNQ
Mm  CEGKGSPSPEKPDVEKEVKPPAKEKIEGTLGILYDQPRHVQYATHFPIPQEESCSHECNQ
Cf  CEGKGSPSPEKPDVEKEVKPPPKEKIEGTLGVLYDQPRHVQYATHFPIPQEESCSHECNQ
Bt  CEGKGSPSPEKPDVEKEVKPPPKEKLEGTLGVLYDQPRHVQYATHFPIPQEESCSHECNQ
Ce  EERRHRILPQLCGSPLDYLPCELHKLPVMEGVEEVVDCLRLRHCEIVRRSQAAEDRWLPN

          1090      1100      1110      1120      1130      1140
Nc  GIFLVSQYIQWLITRGGLTDP--------------QLVMPDGPPFSRLLAELPSHILGSS
Af  QTFSVGKYLQWLMAKGVADFS-------------RSSDHQPLSGDLALLVQLPVSRLPEH
At  IEGSQGASTSHVCDNKVSCPHHGPGVEGENQKVVDVFRTSTPVDMVSVGNSLKQLQFVDK
Dd  ------------------------------------------------------------
Sc  GIIKVPTYIRKLISSGLLYLQ----------------DSNDKFVHVQLLINLKISPLMKS
Ca  KLITIASYLRKLIASGIFYLE---------------PGSTVDQSLNNLPRIMEVHLKILE
Su  LLEKYVIDMEAEKMAEMEVMEDKEMAPISPMPTASTIPVFQVLLQHFLDIRAPVIDDTSS
Dm  ILLFGVGKERDEKKHAVKKMSKEIGKLFTKKFSIDVAAAGHVKKHSRNEFNFEATTSKCQ
Sp  GYFFFSKYIARLASLGYLRES--------------MLNSSFMDDQRKILVQLPILRMSQQ
Hs  RLVVLFGVGKQRDDARHAIKKITKDILKVLNRKGTAETDQLAPIVPLNPGDLTFLGGEDG
Mm  RLVVLFGVGKQRDDARHAIKKITKDILKVLNRKGTAETDQLAPIVPLNPGDLTFLGGEDG
Cf  RLVVLFGVGKQRDDARHAIKKITKDILKVLNRKGTAETDQLAPIVPLNPGDLTFLGGEDG
Bt  RLVVLFGVGKQRDDARHAIKKITKDILKVLNRKGTAETDQLAPIVPLNPGDLTFLGGEDG
Ce  AAFLQSFGRIIDTCVDVIGIMDNIDVEKPNAITNAGLRLFAFREKFEKQEALLKTMLMFK

          1150      1160      1170      1180      1190      1200
Nc  QK-----RSRDALLRRAGFRIADDAQDADMAIR--HLRHALGLP---------------A
Af  VHN-----LRNTLLHRAGVEPSKEAS----TIAILKASIAERLPRIFG-----------S
At  RSIAVWLTTAVRQLVEEPQKSSVRVGQFNRGAPVEEKNTIRWKLGADELYSILFLLDISL
Dd  ------------------------------------------------------------
Sc  QYNMVLRNVMEYDVKFYEIFNFDQLVEITEQIKMRILSN---------------------
Ca  NLPVLNNRQCDSILKRWTPMGFDFKGKFSQGQSILQDSIITKICNNQLTDDIDSQCIGYI
Su  SADDKKAFANLILLFGELIRCEVFSHDSYMCTLIS-------------------------
Dm  QMAYFDQHVVTAQCAANVLEQLNGFALGNNNYLPVQEHVAFLFDLMELALNIYSLLELCD
Sp  LKNKIYYILSKGN-----------------------------------------------
Hs  QKRRRNRPEAFPTAEDIFAKFQHLSHYDQHQVTAQVSRNVLEQITSFALGMSYHLPLVQH
Mm  QKRRRNRPEAFPTAEDIFAKFQHLSHYDQHQVTAQVSRNVLEQITSFALGMSYHLPLVQH
Cf  QKRRRNRPEAFPTAEDIFAKFQHLSHYDQHQVTAQVSRNVLEQITSFALGMSYHLPLVQH
Bt  QKRRRNRPEAFPTAEDIFAKFQHLSHYDQHQVTAQVSRNVLEQITSFALGMSYHLPLVQH
Ce  WCITEEREGSFRATYIAKLLRFGMDQNPENTIGGWQVMDLFFKFMSTEGPKHGSKMYQAH

          1210      1220      1230      1240      1250      1260
Nc  GVTDAVSMGRPFPIKKIARRIEDASRARKAEIASWLRNTVFG--------GELEHSSLNA
Af  VATSAVSCDPLP---------SDLTWTVKSELGQWIRRGVTEFGRDPRRAFQGLHSTT--
At  DLVSAGGRNLVTVPRNVENNMCEIGEAILVSSLRRYENILLSADLVPEAMTALMNRAASL
Dd  ------------------------------------------------------------
Sc  ------------------------------DITNLQLSKTPLSIKIMVAEWYLSHLCSGI
Ca  DSLSVGLRFLLINWLTNELKLKIKSTNKLIHFTPTVITKLYQFYAKNSGLTVFFKVVIQT
Su  -------------------------------------------------------RGDLT
Dm  SLLKELPEVEHQLQLKKSNLVRSYTTSLALYIVSILRRYHSCLLLSPEQTLSVFEGVCRT
Sp  ------------------------------------------------------------
Hs  VQFIFDLMEYSLSISGLIDFAIQLLNELSVVEAELLLKSSDLVGSYTTSLCLCIVAVLRH
Mm  VQFIFDLMEYSLSISGLIDFAIQLLNELSVVEAELLLKSSDLVGSYTTSLCLCIVAVLRH
Cf  VQFIFDLMEYSLSISGLIDFAIQLLNELSVVEAELLLKSSDLVGSYTTSLCLCIVAVLRH
Bt  VQFIFDLMEYSLSISGLIDFAIQLLNELSVVEAELLLKSSDLVGSYTTSLCLCIVAVLRH
Ce  FDSTVAIMIEMMREKIFSITDILRELEKDSDLDYNAPLMERQRKQKIPKISKRHRKPETP
```

Med12

```
                     1270      1280      1290      1300      1310      1320
Nc   FGQHDLSTR--LFNSIRTVFEAAQEFSVFADVLRMLTKCSNPEVLASIADTITRYTFVFA
Af   -SAEHFALTPGEFYTVRDILESFGDLSILADVLKQATVCNDGIVLASAADTVNYHFRSFC
At   MSSNGKISGSAALVYTRYILKRYGSLPSVVEWHNNFKATSEKKLLSELDHTRSGNGEYGN
Dd   ------------------------------------------------------------
Sc   LSSVNRTVLLKIFKIFCIDLEVFHHFFKWIEFIVYHQLLSDIESLEALMDILLCYQKLFS
Ca   ILKNDGGMIIFYLDSLYLISKLVIRHYKLIKSIAGASELSAAYDLFKLIIQVYKDLNTRE
Su   TVPTQAQTGAFVGGPGSQRNDRGAGDKNEAKDDGEEKQDSSDNEDYGYLDDKPPPAPAPP
Dm   IRHVSNPSECTSAERCIIAYLSDLHESCVLLQGKEQSTEYYQQLQCIKRFKDIFNTPEQL
Sp   --------YFVDWSICDEYVKRFKEDHFSFMFKKEENYAIITLSLVKIASTPMSKLYEDY
Hs   YHACLILNQDQMAQVFEGLCGVVKHGMNRSDGSSAERCILAYLYDLYTSCSHLKNKFGEL
Mm   YHACLILNQDQMAQVFEGLCGVVKHGMNRSDGSSAERCILAYLYDLYTSCSHLKSKFGEL
Cf   YHACLILNQDQMAQVFEGLCGVVKHGMNRSDGSSAERCILAYLYDLYTSCSHLKSKFGEL
Bt   YHACLILNQDQMAQVFEGLCGVVKHGMNRSDGSSAERCILAYLYDLYTSCSHLKSKFGEL
Ce   DDTKLVHFTTEYTPKRLFMGKKMDLLERMIIILPQLDVDEDTDEYRLRRLLLFGLKPAAN

                     1330      1340      1350      1360      1370      1380
Nc   ALSCLKNLFSILHSRLREVQEQGIGARPLLASLAYLASRVPGMDEVAMQLKSDLAVSDRH
Af   VIGATTDLFKRLVEAYARLKRLGSTSLDLIFSLIDLGLRLPGELNTVALLRQDLSRIESK
At   PLGVPAGVDNPDDYLRKKISIGGARPSRVGLSMRDVLQRHVEEATHYLKKLIGTGTMKAS
Dd   ------------------------------------------------------------
Sc   QFINDHILFTKTFIFIYKKVLKEKDVPAYNVTSFMPFWKFFMKNFPFVLKVDNDLRIELQ
Ca   YDCFKFSSIWNFINAVIEKSSPTSSATTTATTSVSISSLYLTMGKPKNSKLKSVQGLHVK
Su   PRKESTSFQEKFLESIQSKSDSQSDISDDEKDSPMDGEKSDEEKKSDMDYRSPSPEAPRV
Dm   DLPPQGYNPLLLQELFMAPRRGGKLDPHWLGTLHESPANVYSFVSNALIAVCRETDNERL
Sp   LVMLFAFHYSMFQVMTKLIADNLVHFSFQSCACQTLFFICSVVPKTESQKLLLNEMGKLF
Hs   FSDFCSKVKNTIYCNVEPSESNMRWAPEFMIDTLENPAAHTFTYTGLGKSLSENPANRYS
Mm   FSDFCSKVKNTIYCNVEPSESNMRWAPEFMIDTLENPAAHTFTYTGLGKSLSENPANRYS
Cf   FSDFCSKVKNTIYCNVEPSESNMRWAPEFMIDTLENPAAHTFTYTGLGKSLSENPANRYS
Bt   FSDFCSKVKNTIYCNVEPSESNMRWAPEFMIDTLENPAAHTFTYTGLGKSLSENPANRYS
Ce   VYFRRARAIYKSITKEFTTRLYIEFDRSSKVTTAHKKINQNRLDDLLRQFRAQTYHDQHL

                     1390      1400      1410      1420      1430      1440
Nc   NPVDACSPVSDNMVSRLSDHDGDLHEEVEKLLANGTSLDRNTMDRLFSAVIQRLQAYWSK
Af   SSMAAPSPLSDHIPSSFNENDPLFLLKLDQLLSSASGIDESTLDTIFNVLIKQLE--SSG
At   LAEKNDDGYQVAQQIVVGLMDCIRQTGGAAQEGDPSLVSSAVSAIINSVGLSVARITDFS
Dd   ------------------------------------------------------------
Sc   SVYNDEKLKTEKLKNDKSEVLKVYSMINNSNQAVGQTWNFPEVFQVNIRFLLHNSEIIDT
Ca   QTMDSPMCINTVDESHTHDTRSGAKRYTSTDFRNDLYFLVTLQPRALTSEEKQEVFDLIE
Su   NRHQLYVKHFPISTEDSSAYHESNQRSVLLYGFGRARTEARQQVKHVQKDLLKILTQGGG
Dm   NDVALACAELTASCNVLSEEWIYALQSLCSGSKSPRYPHLGGQVDIGQLKTHNALAVFVC
Sp   QVELNFSYDSPDVNLLIEQFYEITSYESNYDDAFVEYKDATVANRKDFIEFLFHNITVSS
Hs   FVCNALMHVCVGHHDPDRVNDIAILCAELTGYCKSLSAEWLGVLKALCCSSNNGTCGFND
Mm   FVCNALMHVCVGHHDPDRVNDIAILCAELTGYCKSLSAEWLGVLKALCCSSNNGTCGFND
Cf   FVCNALMHVCVGHHDPDRVNDIAILCAELTGYCKSLSAEWLGVLKALCCSSNNGTCGFND
Bt   FVCNALMHVCVGHHDSDRVNDIAILCAELTGYCKSLSAEWLGVLKALCCSSNNGTCGFND
Ce   ILERIVYNFIDGIGGFLKKNCDDVPAPEVANIICEMYEFSMDITSIFDFFEMVNPYLKAV

                     1450      1460      1470      1480      1490      1500
Nc   GDEQQRVYG--ILLKRLRMFNPQHFDELMTRWLLYVRGLDTRATILHLYPLLVSIGCLEM
Af   GHAKFSVNEICRYLSYLRPFHPKRFDTMIVRWVCGLLRSSTGGILSQVLPPLIGVGCVTI
At   LGNIYQNHPSGVDSSNIARYILRIHITCLCLLKEALGERQSRVFEIALATESSTALTGVF
Dd   ------------------------------------------------------------
Sc   NTSKQFQKARNNVMLLIATNLKEYNKFMSIFLKRKDFTNKNLIQLISLKLLTFEVTQNVL
Ca   IELKDLDSALEFWYNNFELLGEEKEINLMKIIKSLAVTTYNDHLSQFNEKLKQFVISKVG
Su   KGEVSKGKKVSKLDEPTVNKEETMQRFLALCYFDQHMVTGSCANGVLDQVQAFSMGNRDT
Dm   ILVARHCFSLADFVSKFALPTLARSVSAGGAELSVDAEAGARLTCHLVLKLFKTLEIPQP
Sp   KHTAVIFTSDLLMVLKIALNHPPYFDLATTTFSSLLKRDECTILSFFKILQFYGCKLLSV
Hs   LLCNVDVSDLSFHDSLATFVAILIARQCLLLEDLIRCAAIPSLLNAACSEQDSEPGARLT
Mm   LLCNVDVSDLSFHDSLATFVAILIARQCLLLEDLIRCAAIPSLLNAACSEQDSEPGARLT
Cf   LLCNVDVSDLSFHDSLATFVAILIARQCLLLEDLIRCAAIPSLLNAACSEQDSEPGARLT
Bt   LLCNVDVSDLSFHDSLATFVAILIARQCLLLEDLIRCAAIPSLLNAACSEQDSEPGARLT
Ce   DDKIAHFRMDVLPDMYYTETAFIFVSFFMKHWQRFLLHPRACAIVNQCFVLIQDMIRADD

                     1510      1520      1530      1540      1550      1560
Nc   PAILATASESPNALGAGNSRHPQSAVATSQIVQTTFRTRYMQEVLQLFMAPVSSESLLTP
Af   QAFVFLVRR---LLKSDNKISNQRDLR--------------IDLLQLLVPPPAGQSRYFD
At   APVKGSRGQHQLSPESYDSNANNSTIDMSNGTGKMALSRATKITAAVSALVIGSITHGVI
Dd   ------------------------------------------------------------
Sc   GLEYIIRLLPINLENNDGSYGLFLKYHKEQFIKSNFEKILLTCYELEKKYHGNECEINYY
Ca   ESIDHSKLSNFLKKLAIFDLLRINDLVKLIQSIQRDEINHIDDTISFIYELIWGELDKAH
Su   PPTFDQVTLVFELLENALNINGLLEFAVKHVSNVTNCTSAERCILAYLYDLYTSCSHLKS
Dm   GMYSVSTSPNPLHAVGNDFSIRLSCDRHLLVGAHKTIPIAAVLAVLKAILIVVDNAALKT
Sp   DQIWAVVSDVYEAQDNNTTLKQFFNYLLDESTWPEGYLEERHWRSILCKEARKHDSGLKL
Hs   CRILLHLFKTPQLNPCQSDGNKPTVGIRSSCDRHLLAASQNRIVDGAVFAVLKAVFVLGD
Mm   CRILLHLFKTPQLNPCQSDGNKPTVGIRSSCDRHLLAASQNRIVDGAVFAVLKAVFVLGD
Cf   CRILLHLFKTPQLNPCQSDGNKPTVGIRSSCDRHLLAASQNRIVDGAVFAVLKAVFVLGD
Bt   CRILLHLFKTPQLNPCQSDGNKPTVGIRSSCDRHLLAASQNRIVDGAVFAVLKAVFVLGD
Ce   HMITCWGRTVAIFVFHARKAIANAGLQNEEFLAEDSHFWRVFPNAQHVDLDVGYFNEDFA

                     1570      1580      1590      1600      1610      1620
Nc   EERYRFGILQEQAKKESPRELLCLIQLALAEYTCARAQNDLEELPLDK-----------E
Af   LVTYRFHLSRKEFLFKHPEEAFDIIRDAIALIDSESQEGNYRQVDLGHSAMVLLQILLTK
At   TLERIVGLLRLKDYLDFVQFVRRTKSSSNGSARSMGASKVESPIEVYVHWFRLLVGNCKT
Dd   ------------------------------------------------------------
Sc   EILLKILITYGSSPKLLATSTKIIMLLLNDSVENSSNILEDILYYSTCPSETDLNDIPLG
Ca   FSCSQLVLLQISRRMYQEKSSYQCIILLQQRLLKNKESQLQTNPFFIKYKADLLSFFHKA
Su   KFSDLFSSVANKVKQALYTSITPSESNSRWNQDFMLEYIDNP------------------
Dm   PLASGSGTSSGGLGGAFGSGKRSGFNTPVHPGSTPKSNEQRPADLSQILGTSDLQLGSSL
Sp   FKLGIKLCTRNEQIMKTIWSFIHVHDCNVISEVIPDQRFLRTLTEHFMIDLRQLDIVTCL
Hs   AELKGSGFTVTGGTEELPEEEGGGGSGGRRQGGRNISVETASLDVYAKYVLRSICQQEWV
Mm   AELKGSGFTVPGGTEELPEEEGGGGSSGRRQGGRNISVETASLDVYAKYVLRSICQQEWV
Cf   AELKGSGFTVTGGTEELPEEEGGGGSGGRRQGGRNISVETASLDVYAKYVLRSICQQEWV
Bt   AELKGSGFTVTGGTEELPEEEGGGGSGSRRQGGRNISVETASLDVYAKYVLRSICQQEWV
Ce   GVKLQLRGGTLRYDSYNDFKWLVSNMKPNLKKKPHLKRPNTRYSFVVRAFMEARQHGRNF

                     1630      1640      1650      1660      1670      1680
Nc   ANQDRLLRLLKLLVLKDPVG----VAKALQIRGPDAQIDGWIDRMTTKLLVTAAGEGTR-
Af   NPESAVRHCSEKLIGQHPSAVTVLTRALDSLLGLDSKAGGHLFTSNDTFIFIPIDTGPAT
At   VSEGLVLELVGESSVVAISRMQRMLPLKLVFPPAYSIIAFVLWRPFVSNSNSNSSVHEDT
Dd   ------------------------------------------------------------
Sc   SGQPDNDTVVTNDDKSDDDDHTVDEIDHVEYYVMMDFANLWVFQAFTCFCIKKIMENNEP
Ca   IITNTKAIQSQFFDKLSHSDSLQLLNVLLQRQENEYIN---TIQDFSLFIKHINEFNLPI
Su   ------------------------------------------------------------
Dm   TSEPEALQQPSVGGMEQISLLEFAQAVLKQICAQEHVLERCLKNAEQLCDMIIDEMLTAK
Sp   KKALVTLDEFSAPLYATWLTTLDDDELSELTDDVVQKKVLESLDNYKSGIWKLVLSGLPN
Hs   GERCLKSLCEDSNDLQDPVLSSAQAQRLMQLICYPHRLLDNEDGENPQRQRIKRILQNLD
Mm   GERCLKSLCEDSNDLQDPVLSSAQAQRLMQLICYPHRLLDNEDGENPQRQRIKRILKNLD
Cf   GERCLKSLCEDSNDLQDPVLSSAQAQRLMQLICYPHRLLDNEDGENPQRQRIKRILQNLD
Bt   GERCLKSLCEDSNDLQDPVLSSAQAQRLMQLICYPHRLLDNEDGENPQRQRIKRILQNLD
Ce   DRINELANYCANITANDPPLSEYWIGAIKGLCFLSLDAPYPFKEMSQQIDISDCSTHYSL
```

Med12

```
         1690      1700      1710      1720      1730      1740
Nc  ---VTFEQVLNLTNEFTLPFCHVKLLLSLSSNDQQQNAADSGERVPSNVELFMNAMEKSM
Af  PDISVAEKVIELTNDFSLPFCQLKLQLLFN-------AESKGNVRNEIVDVMFKAAVADS
At  HRLYQSLTMAFHDVIKHLPFRDVCFRDTQGLYELIVADSTDAEFASVFESHGLDMHLKSV
Dd  ------------------------------------------------------------
Sc  AMAMEDLKNFIFQIIEITNSNDLCSQIFDQLKDMQTIEMITQIVEKDFCTSCLQNNNQKI
Ca  CQVLLNVLFNNLVVDNDVGIKVQLEAFVNTLLQEIKFEFSNENSFFGELFNFMPWNQKNY
Su  ------------------------------------------------------------
Dm  QAQRVLHMICYPEPEFNIISELDQRSMIVRILENLGQWTLRISWLDLQLMYRQSLSNNAE
Sp  CKTVFEHLLLFSLEERLDLPAAFLQDLIGASAYVMEQVPDSWFLEKLPCPLTQSLQSFSH
Hs  QWTMRQSSLELQLMIKQTPNNEMNSLLENIAKATIEVFQRSAETGSSSGSTASNMPSSSK
Mm  QWTMRQSSLELQLMIKQTPNTEMNSLLENIAKATIEVFQQSAETGSSSGSTASNMPSSSK
Cf  QWTMRQSSLELQLMIKQTPNNEMNSLLENIAKATIEVFQQSAETGSSSGSTTSNMPSSSK
Bt  QWTMRQSSLELQLMIKQTPNNEMNSLLENIAKATIEVFQQSAETGSSSGNAASNMPSSSK
Ce  TTFITCLAGKSAFYIPRLLAELTKHVFPLMLRHDGRLTSQKTHDVKRKVASKTTESRTLS

         1750      1760      1770      1780      1790      1800
Nc  DAQRVSWIGMLPSLSPD-----LTHHLKNQAQARFLNLLPSARNPPSMTLDRSMLQMRLQ
Af  RSRRSNWVGLVSLMSHDAVRQVKCHDIRERAEKNFFATPLFEESPDGCSSFAADNSSSLE
At  AFAPLRARLFLNSLIDCKVPSSGYSHEGVSEAKNRHQGNGTKLVDKLVSVLDCLQPAKFH
Dd  ------------------------------------------------------------
Sc  DDNYIVVVIEIITSLSMRFQRETSGMIVISMENYHLLIKIIRQLSELNEGNLSKREIQID
Ca  ILDILETKFLLETNLDNNQIKLIINDRNLLPPLNDFFKKFSSSSSSSTSSSSSSLVQTIE
Su  ------------------------------------------------------------
Dm  LNVWLDTVARAAIDVFHMEEVVLPGAVKATHKPKPSTWLVAPLIAKLTPAVQGRILRVAG
Sp  LSNHIEVLDSTRQSRLTFLCHLLLHMHGFVELTDQLATLESLTIRKCIYRNQELLDLLLF
Hs  TKPVLSSLERSGVWLVAPLIAKLPTSVQG--HVLKAAGEELEKGQHLGSSSRKERDRQKQ
Mm  TKPVLSSLERSGVWLVAPLIAKLPTSVQGHVLKAAG--EELEKGQHLGSSSRKERDRQKQ
Cf  TKPVLSSLERSGVWLVAPLIAKLPTSVQGHVLKAAGEELEKG--QHLGSSSRKERDRQKQ
Bt  TKPVLSSLERSGVWLVAPLIAKLPTSVQGHVLKAAGEELENGQHLDSSS--RKERDRQKQ
Ce  EAEPGVCLCLLIICGLCCVGDEPFGLSVHYRGIEKKKKRFNNTADERIMHLFHWFEMDHA

         1810      1820      1830      1840      1850      1860
Nc  KAENFLAIMDT---IIRGSGPMGFRQHQLVPVMVERFTDILELLQTLTPFPSQAGGLGGW
Af  AAKLYLAIIEKLAYSIPDVGPQ-----AVVPVLTEKLDLLLQRLIS-----MQASCSGTT
At  WQWVELRLLLNEQALAEKLENHDMPLTDAIRSSCPTSEKPDASENEKNFIQILLTRLLVR
Dd  ------------------------------------------------------------
Sc  AVLKIFSFHQDSIFQRIIADLSADKPTSPFIDSICKLFDKISFNLRLKLFLYEILSSLKS
Ca  TDSSFFLNLFNFIGNLVNVANSQELILEQANSINDTISIFLRILIIHKLYLCSMISRNTN
Su  ------------------------------------------------------------
Dm  QVLESMNYFSKVSKSDCNSSGSGDEREKSNSCHSSNSYGLGGVPARNKKMPLDYQPFLGL
Sp  SIHLVKPNVETNDEVCNTLKAWENIESRPYTIDFPEALQQYSPRIVLHLFSFFYWKCQHD
Hs  KSMSLLSQQPFLSLVLTCLKGQDEQREGLLTSLYSQVHQIVNNWRDDQYLDDCKPKQLMH
Mm  KSMSLLSQQPFLSLVLTCLKGQDEQREGLLASLHSQVHQIVINWRENQYLDDCKPKQLMH
Cf  KSMSLLSQQPFLSLVLTCLKGQDEQREGLLTSLYSQVHQIVNNWRDDQYLDDCKPKQLMH
Bt  KSMSLLSQQPFLSLVLTCLKGQDEQREGLLTSLYSQVHQIVTNWRDDQYLDDCKPKQLMH
Ce  MFRTLGHISQLLEALQSRCRDANLVLPKNFPIKNPPKHQQELHREKAPYRPQYLFNIAKT

         1870      1880      1890      1900      1910      1920
Nc  GGQGASAAAAGGDSHIDLKSDILNHWLPLFLNFLTLHAQTFDTSKPGNEVRG--------
Af  ELSHGVDAEQMIRSRTQFER-ALAFWFSALLRMIVLHR--TAFSVPSAAVRPNALPEQTR
At  PDAVPLFSEVVHLFGRSVEDSMLKQAEWFLAGQDVLFGRKTIRQKLIIVGESKGLPTKPQ
Dd  ------------------------------------------------------------
Sc  FAIYSSTIDAPAFHTSGKVELPKKLLNLPPFQVSSFVKETKLHSGDYGEEEDADQEESFS
Ca  EENVILFVTKLIDLLNSDYLTNHNDKLRILLYDLLLLMKSSVSEEVNSTRELNENLQGNR
Su  ------------------------------------------------------------
Dm  ILTCLKGQDEYKENLLVSLYAQLSQCLQSFAELDTIGGIDEPQAREEILDALQLRFSLVG
Sp  DYLQENTSSKNNLLQKEWILITKKKVILEQCEYVILDILNFLKFSGIPTQSKKRLEVHML
Hs  EALKLRLNLVGGMFDTVQRSTQQTTEWAMLLLEIIISGTVDMQSNNELFTTVLDMLSVLI
Mm  EALKLRLNLVGGMFDTVQRSTQQTTEWAQLLLEIIISGTVDMQSNNELFTTVLDMLSVLI
Cf  EALKLRLNLVGGMFDTVQRSTQQTTEWAVLLLEIIISGTVDMQSNNELFTTVLDMLSVLI
Bt  EALKLRLNLVGGMFDTVQRSTQQTTEWAVLLLEIIISGTVDMQSNNELFTTVLDMLSVLI
Ce  VQFVICEQDWVTLRMFRFFQTRKMEAFNQDKLKQNCLGQQILRMALRRRTERENVHKLFE

         1930      1940      1950      1960      1970      1980
Nc  -RALMVCAGLIR----ELDLIHGPD--------FDTRQLGG-----RIFDLACVLVDNLA
Af  LLISIFCISLARLPDSVLRLFPAADYFPHSMRAADCRPCPGILLQTHALDVAASLIDMFP
At  FWKPWGWCNSSSSDHITANKAGKKRKFEITSIEEGEVIEEGSGSRKVLLPRVLDENSPSV
Dd  ------------------------------------------------------------
Sc  LNLGIGIVEIAHENEQKWLIYDKKDHKYVCTFSMEPYHFISNYNTKYTDDMATGSNDTTA
Ca  NGTGTSGGGSNAISPNQDSILDDQNTNSTGIGKGQKSQIGVTNNNDMLDVAPTVSLIQVL
Su  ------------------------------------------------------------
Dm  GMFEAIQKNSTPTTDWAILLAQLVCQGVVDLSCNRELFTTVVDMLATLVHSTLVSDDERH
Sp  NFISSTLYLKVSFCSSNFLFKTISVLVYDLIT----------------------------
Hs  NGTLAADMSSISQGSMEENKRAYMNLAKKLQKELGERQSDSLEKVRQLLPLPKQTRDVIT
Mm  NGTLAADMSSISQGSMEENKRAYMNLVKKLQKDLGERQSDSLEKVHQLLPLPKQNRDVIT
Cf  NGTLAADMSSISQGSMEENKRAYMNLVKKLRKELGERQSDSLEKVRQLLPLPKQTRDVIT
Bt  NGTLAADMSSISQGSMEENKRAYMNLVKKLRKELAERQSDSLEKVYQLLPLPKPTRDVIT
Ce  AHKISKKATVDKVLSFMNLWNFRATLFDLMLMIKEISPDGNSRHAQQGAIAADALMSEIG

         1990      2000      2010      2020      2030      2040
Nc  EDARLQCIQALKS--------PSDVKLR-YIFSYQDNPHA--------------------
Af  DEARHQCARYLREKCPPFARVQNDSRFLYLLGPLGDSPSS--------------------
At  GYGITTERAFVQLVLPCIDQSSDESRSTFVNELVRQFSNI--------------------
Dd  ------------------------------------------------------------
Sc  FNDSCVNLSLFDARFERKNPH---------------------------------------
Ca  FDLPEPNASNPFKEFIDDKLVKCSIMLEEEELKYGGDYHI--------------------
Su  ------------------------------------------------------------
Dm  YMNLMKKLKKEIGEKNNASIRVIRQLLPLYKQPTEVIACE--------------------
Sp  ------------------------------------------------------------
Hs  CEPQGSLIDTKGNKIAGFDSIFKKEGLQVSTKQKISPWDL--------------------
Mm  CEPQGSLIDTKGNKIAGFDSIFKKEGLQVSTKQKISPWEL--------------------
Cf  CEPQGSLIDTKGNKIAGFDSIFKKEGLQVSTKQKISPWDL--------------------
Bt  CEPQGSLIDTKGNKIAGFDSIFKKEILFPLLQAFKVCVVFSKFQQLTISHFLEQGLQVST
Ce  KCCRDMFLSAYKTKIKMPIAKTLTDFRLSDINKFWLIAPLVRMCPKPINIPPQYANTTVG

         2050      2060      2070      2080      2090      2100
Nc  --------NLMLCHKERPTAMAIG-TATGTGSPAPGTTPTHTPGVTPGPQNAVGAGSAVS
Af  --------NIT-----LPVSIPSP-AASG-STPAPTPSGNSTGGFSHPQQPAFVSGVPPG
At  --------EQQLSSVTNRSTTSNK-QMGTASSGSEISSNKGSTRKGLRGGSPSLARRSSA
Dd  ------------------------------------------------------------
Sc  ------------------------------------------------------------
Ca  --------FNNTGLYLKSTKNESV-SLPPFVTGLNKTAGDYDSSSARMIKQPFKMKSFEI
Su  ------------------------------------------------------------
Dm  --------HSGMDTKGNKICDIDK-KQLRISDKQRISVWDILEGHKNPAPLSWVWFGAVK
Sp  ------------------------------------------------------------
Hs  --------FEGLKPSAPLSWGWFG-TVRVDRRVARGEEQQRLLLYHTHLRPRPRAYYLEP
Mm  --------FEGLKPSTAPLSWAWFGTVRVDRRVARGEEQQRLLLYHTHLRPRPRAYYLEP
Cf  --------FEGLKPSAPLSWGWFG-TVRVDRRVARGEEQQRLLLYHTHLRPRPRAYYLEP
Bt  KQKISPWDLFEGLKPSAPLSWGWFGTVRVDRRVARGEEQQRLLLYHTHLRPRPRAYYLEP
Ce  TVAAKFLREAALLMDTPPTTPKERLLQCSWAMSKVPFINMILTCLQCEKMQQSKDVFLQS
```

Med12

```
           2110      2120      2130      2140      2150      2160
Nc   GAGGSGNGGSYFSLPGHTQQAQAQGWHAAQQQQQQQQQQQSQQQQQQQQQQQYQLQQQQQ
Af   LPDGLNCAASHLCLQYRGRVIGAYPVRPWELLEDAAPIAGTNDTAVSLGYFDARRVRV--
At   NTTDTSPPPSPAALRASMSLRLQFLLRLLPVICGEPSFKNTRHALASTIVRLLGSRVVYE
Dd   ------------------------------------------------------------
Sc   ------------------------------------------------------------
Ca   LEDVNNNKNSVNDGCINLRLFDAYTTKENPR-----------------------------
Su   ------------------------------------------------------------
Dm   LERKPLTYEEAHRNLKYHTHSLVKPSSYYYEPLPLPPEDIEPVPEKICIKDEMKADTPSS
Sp   ------------------------------------------------------------
Hs   LPLPPEDEEPPAPTLLEPEKKAPEPPKTDKPGAAPPSTEERKKKSTKGKKRSQPATKTED
Mm   LPLPPEDEEPPAPALLEPEKKAPEPPKTDKPGAAPPSTEERKKKSTKGKKRSQPATKNED
Cf   LPLPPEDEEPPAPTLLEPEKKAPEPPKTDKPGAAPPSTEERKKKSTKGKKRSQPAPKTED
Bt   LPLPPEDEEPPAPTLLEPEKKAPEPPKTDKPGAAPPSTEERKKKSTKGKKRSQPAAKTEV
Ce   LYTQLQRETLRDHHRRSNWTNRREHRDCTIFRITLIGYIYKEILKATHVETWGLLIFQLM

           2170      2180      2190      2200      2210      2220
Nc   -------------------------------------QGGTGPIPSPAISINTNLTHLNL
Af   ------------------------------------------------------------
At   -------------------------------------DYAVCSPRSELSKAETESTIDPS
Dd   ------------------------------------------------------------
Sc   ------------------------------------------------------------
Ca   ------------------------------------------------------------
Su   ------------------------------------------------------------
Dm   -------------------------------------VDQSPSAVVGGTGRGRGKGTTTR
Sp   ------------------------------------------------------------
Hs   -------------------------------------YGMGPGRSGPYGVTVPPDLLHHP
Mm   -------------------------------------YGMGPGRSGPYGVTVPPDLLHHA
Cf   -------------------------------------YGMGPGRSGPYGVTVPPDLLHHA
Bt   STAAAIPVHAIPVHVFPSPTLFLILVVGGLGAFEEGDYGMGPGRSGPYGVTVPPDLLHHA
Ce   FHGIINPVREKLIYETCFDMLHHMVLWTLVDGDSMNQHDRYGSIRVRWPQYAGLMKKIRK

           2230      2240      2250      2260      2270      2280
Nc   GNNSNPPTPSPMNPTRPAMMMVANPWGGYSWVPTMGGPQERLTPFNFRQWEMLSEPTSQV
Af   ------------------------------------------------------------
At   SMADLSSEVLFDRRELTGLSTNFKKSLKVCLLGCKNELSRMQLPDTIRWRIQAAMPILLP
Dd   ------------------------------------------------------------
Sc   ------------------------------------------------------------
Ca   ------------------------------------------------------------
Su   ------------------------------------------------------------
Dm   KRKPKNPKTPPVVNTQQQQPQLAQQPQQPQNVQQQQLQQQQQQQQHMQQQHMQQQQMQPN
Sp   ------------------------------------------------------------
Hs   NPGSITHLNYRQGSIGLYTQNQPLPAGGPRVDPYRPVRLPMQKLPTRPTYPGVLPTTMTG
Mm   NPGSISHLSYRQSSMGLYTQNQPLPAGGPRVDPYRPVRLPMQKLPTRPTYPGVLPTTMST
Cf   NPSSISHLSYRQGSIGLYTQNQPLPAGGPRVDPYRPVRLPMQKLPTRPPYTGVLPTTMTG
Bt   NPSSISHLSYRQSSIGLYTQNQPLPAGGPRVDPYRPVRLPMQKLPTRPPYPGVLPTTMTG
Ce   EMQERFTDQTRNSLHRFLPIGKMQMSTISYKKYQKRPKVNQKMSKKFLAGEGLKNGKYSF

           2290      2300      2310      2320      2330      2340
Nc   GENDTAVNLM--LFESRKVQ----------------------------------------
Af   ------------------------------------------------------------
At   SLRCSLSCQP--HSVPPTALTLVQPSGSTAAAGTNQRNSPAISKSGTAAAQGKLKPTMLA
Dd   ------------------------------------------------------------
Sc   ------------------------------------------------------------
Ca   ------------------------------------------------------------
Su   ------------------------------------------------------------
Dm   QMGQMPMNMP--MNMQQFAPNPNNMMQQNAMLQQQQQQQMQQMGNNPMQQQLNVGGGNGQ
Sp   ------------------------------------------------------------
Hs   VMGLEPSSYK--TSVYRQQQPAVPQGQRLRQQLQQSQGMLGQSSVHQMTPSSSYGLQTSQ
Mm   VMGLEPSSYK--TSVYRQQQPTVPQGQRLRQQLQQSQGMLGQSSVHQMTPSSSYGLQTSQ
Cf   VMGIEPSYKTSVYRQQQPTVPQGQRLRQQLQAKIQSQGMLGQSTVHQMTPSSSYGLQTSQ
Bt   VMGLEPASYKTSVYRQQQPAVPQGQRLRQQLQAKISQGMLGQSSVHQMTPSSSYGLQTSQ
Ce   LPEEKAKTNAFEHTDHLGDLIVKGGWKFRMFQTTRLDKVAKNVQNVLRSNMHHTHVLEFN

           2350      2360      2370      2380      2390      2400
Nc   ------------------------------------------------------------
Af   ------------------------------------------------------------
At   PHQQQEADNTDVVDPWTLLEDGTSSGLSSSNASNSSDMANLRATCWLKGAVRVRRTDLTY
Dd   ------------------------------------------------------------
Sc   ------------------------------------------------------------
Ca   ------------------------------------------------------------
Su   ------------------------------------------------------------
Dm   PNPQMNFMQQGPGGGGAGPQGMPGQQQQWHNAPQQQQPPQPYHNQYAPHQQNMQSNRIER
Sp   ------------------------------------------------------------
Hs   GYTPYVSHVGLQQHTGPAGTMVPPSYSSQPYQSTHPSTNPTLVDPTRHLQQRPSGYVHQQ
Mm   LSSPSLQGYTSYVSHVGLQQHTGPADPTRHLQQRPSGYVHQQAPTYGHGLTSTQRFSHQT
Cf   GYTPYVSHVGLQQHTGPAGTMVPPSYSSQPYQSTHPSTNPTLVDPTRHLQQRPSGYVHQQ
Bt   GYTPYVSHVGLQQHTGPAGTMVPPSYSSQPYQSTHPSTNPTLVDPTRHLQQRPSGYVHQQ
Ce   RPQLLMSDNMFDDIFLAPPDIEITKIIEQPVPVIDEEEAKKRAEEEKEAAEKKEESKNAE

           2410      2420      2430      2440      2450      2460
Nc   ------------------------------------------------------------
Af   ------------------------------------------------------------
At   VGSVDDDS----------------------------------------------------
Dd   ------------------------------------------------------------
Sc   ------------------------------------------------------------
Ca   ------------------------------------------------------------
Su   ------------------------------------------------------------
Dm   PPLNANSKQALSQMLRQRQPFQQQA---------QQGPGGGFNPMQQQPQASQQQPGPQQ
Sp   ------------------------------------------------------------
Hs   APTYGHGLTSTQRFSHQTLQQTPMI---------STMTPMSAQGVQAGVRSTAILPEQQQ
Mm   LQQTPMMGTMTPLSAQGVQAGVRST---------SILPEQQQQQQQQQQQQQQQQQQQQQ
Cf   APTYGHGLTSTQRFSHQTLQQAPMIGTMTPLGAQGVQAGVRSASILPEQQQQQQQQQQQQ
Bt   APTYGHGLTSTQRYPSEPGMAETLRLSIWENLGLDVTLAPAQLPIPDSLWFFTDTQPQPS
Ce   DEKNKNNAENKKDTKEGEKGKSKDKEKDGEKEKCKDASKKDDVTSEKNELEKRASDAAAA

           2470      2480      2490      2500      2510      2520
Nc   ------------------------------------------------------------
Af   ------------------------------------------------------------
At   ------------------------------------------------------------
Dd   ------------------------------------------------------------
Sc   ------------------------------------------------------------
Ca   ------------------------------------------------------------
Su   ------------------------------------------------------------
Dm   QMNPNQMRQQQMNPQQNPQSVAAFNAMQQQPQQNAQQQQMNPNQQQQQQFMRGGNMRPGM
Sp   ------------------------------------------------------------
Hs   QQQQQQQQQQQQQQQQQQQQQQQYHIRQQQQQQILRQQQQQQQQQQQQQQQQQQQQQQQQ
Mm   QQQQQQQQQQQQYHIRQQQQQQQMLRQQQQQQQQQQQQQQQQQQQQQQQQQQQPHQQQQQ
Cf   QQQQQQQQQQQQQQQQQQQQQQYHIRQQQQQQILRQQQQQQQQQQQQQQQQQQAHQQQQQ
Bt   TNIFGRY-----------------------------------------------------
Ce   TNAPETNKDMDTSTPKPAPVTRSPATRGRGGGRKRNSGARGGGPRAKRANSRADTAQAAA
```

Med12

```
                    2530      2540      2550      2560      2570      2580
Nc   ------------------------------------------------------------
Af   ------------------------------------------------------------
At   ------------------------------------------------------------
Dd   ------------------------------------------------------------
Sc   ------------------------------------------------------------
Ca   ------------------------------------------------------------
Su   ------------------------------------------------------------
Dm   APNQMNQMNMGGQGMSQNPMMQQQIPQNMVGMVNPNANQMMQSGGAQGGNGVGVGVGVGV
Sp   ------------------------------------------------------------
Hs   QQHQQQQQQQAAPPQPQPQSQPQFQRQGLQQTQQQQQTAALVRQLQQQLSNTQPQPSTNI
Mm   AAPPQPQPQSQPQFQRQGLQQTQQQQQTAALVRQIQQQLSNTQPQPSTNIFGRY------
Cf   AAPPQPQPQSQPQFQRQGLQQTQQQQQTAALVRQLQQQLSNTQPQPSTNIFGRY------
Bt   ------------------------------------------------------------
Ce   ATTQWNAPIANTSNPAAGGNFHAAMRGNQPPMSNGSSDETKVHIRNLLNRKKEEKRNSLA

                    2590      2600      2610      2620      2630      2640
Nc   ------------------------------------------------------------
Af   ------------------------------------------------------------
At   ------------------------------------------------------------
Dd   ------------------------------------------------------------
Sc   ------------------------------------------------------------
Ca   ------------------------------------------------------------
Su   ------------------------------------------------------------
Dm   GGAGNNPNMGMGGMPQQGMIQQQPQQQPQQQVQFQNFQNQYQQQQQQGMQQQGGGAGVGV
Sp   ------------------------------------------------------------
Hs   FGRY--------------------------------------------------------
Mm   ------------------------------------------------------------
Cf   ------------------------------------------------------------
Bt   ------------------------------------------------------------
Ce   DASAAAAAANSNAMGNTSSMPPSGPPMPMGSSMQSAGATQQLQGMQKHQMGGSMSGMNQN

                    2650      2660      2670      2680      2690      2700
Nc   ------------------------------------------------------------
Af   ------------------------------------------------------------
At   ------------------------------------------------------------
Dd   ------------------------------------------------------------
Sc   ------------------------------------------------------------
Ca   ------------------------------------------------------------
Su   ------------------------------------------------------------
Dm   GVGMAPNQQQQQQANMMGNFNPQMQQGNRNNPDFMAAAAVAQQQQQQQQQQRVVPGGMMA
Sp   ------------------------------------------------------------
Hs   ------------------------------------------------------------
Mm   ------------------------------------------------------------
Cf   ------------------------------------------------------------
Bt   ------------------------------------------------------------
Ce   MGGMNQSMSHQAPPPYSSTNEMNRPLMNQYGGPHFAAPNPGPLNRSSGPVSSETRQQIME

                    2710      2720      2730      2740      2750      2760
Nc   ------------------------------------------------------------
Af   ------------------------------------------------------------
At   ------------------------------------------------------------
Dd   ------------------------------------------------------------
Sc   ------------------------------------------------------------
Ca   ------------------------------------------------------------
Su   ------------------------------------------------------------
Dm   GNRNQYMNQAPNVTMSTMMGPGPGGVVGQVPPYARQQSAGGGKPGVLNTQQQFQQQQQQQ
Sp   ------------------------------------------------------------
Hs   ------------------------------------------------------------
Mm   ------------------------------------------------------------
Cf   ------------------------------------------------------------
Bt   ------------------------------------------------------------
Ce   QQMREKLAAHHQLVEQQKQRDAREREAREREAREHQERMQREAYMKEQQLLERKRAIEEN

                    2770      2780      2790      2800      2810      2820
Nc   ------------------------------------------------------------
Af   ------------------------------------------------------------
At   ------------------------------------------------------------
Dd   ------------------------------------------------------------
Sc   ------------------------------------------------------------
Ca   ------------------------------------------------------------
Su   ------------------------------------------------------------
Dm   QQLRHQMMQLQGMGGGAGGGMGAGPQQGGGAVGGGAGGGMVPQQQSMNQQQTPNLVAQLQ
Sp   ------------------------------------------------------------
Hs   ------------------------------------------------------------
Mm   ------------------------------------------------------------
Cf   ------------------------------------------------------------
Bt   ------------------------------------------------------------
Ce   NRIMEEQQREREMEAARKEAARRAAEEAYAAEQQRLELLRRQEEERLRKEAEERMRIQRE

                    2830      2840      2850      2860      2870      2880
Nc   ------------------------------------------------------------
Af   ------------------------------------------------------------
At   ------------------------------------------------------------
Dd   ------------------------------------------------------------
Sc   ------------------------------------------------------------
Ca   ------------------------------------------------------------
Su   ------------------------------------------------------------
Dm   RQNMMGQQQYQPPPY---------------------------------------------
Sp   ------------------------------------------------------------
Hs   ------------------------------------------------------------
Mm   ------------------------------------------------------------
Cf   ------------------------------------------------------------
Bt   ------------------------------------------------------------
Ce   NEERVRQEQMRLEAEERERIRRAEEERIQKELEDKVRREKEEAARQEKERQEQEARMREA

                    2890      2900      2910      2920      2930      2940
Nc   ------------------------------------------------------------
Af   ------------------------------------------------------------
At   ------------------------------------------------------------
Dd   ------------------------------------------------------------
Sc   ------------------------------------------------------------
Ca   ------------------------------------------------------------
Su   ------------------------------------------------------------
Dm   ------------------------------------------------------------
Sp   ------------------------------------------------------------
Hs   ------------------------------------------------------------
Mm   ------------------------------------------------------------
Cf   ------------------------------------------------------------
Bt   ------------------------------------------------------------
Ce   REAELSRQRMEQQRRSQQNPYMNQQGQYSQQPPPSYQQSSYPNNYQPGQQGNQPPNYQQP
```

Med12

```
                           2950      2960      2970      2980      2990      3000
Nc  ------------------------------------------------------------
Af  ------------------------------------------------------------
At  ------------------------------------------------------------
Dd  ------------------------------------------------------------
Sc  ------------------------------------------------------------
Ca  ------------------------------------------------------------
Su  ------------------------------------------------------------
Dm  ------------------------------------------------------------
Sp  ------------------------------------------------------------
Hs  ------------------------------------------------------------
Mm  ------------------------------------------------------------
Cf  ------------------------------------------------------------
Bt  ------------------------------------------------------------
Ce  SHQSMQQGHQAGYQQTSNQMQMNMQQQQNRQQGGPQQSFSGPGGINQPSQPGYSGYNQQG

                           3010      3020      3030      3040      3050      3060
Nc  ------------------------------------------------------------
Af  ------------------------------------------------------------
At  ------------------------------------------------------------
Dd  ------------------------------------------------------------
Sc  ------------------------------------------------------------
Ca  ------------------------------------------------------------
Su  ------------------------------------------------------------
Dm  ------------------------------------------------------------
Sp  ------------------------------------------------------------
Hs  ------------------------------------------------------------
Mm  ------------------------------------------------------------
Cf  ------------------------------------------------------------
Bt  ------------------------------------------------------------
Ce  GQGQQGQMQQQRNPFGNQQDMQQPGAAKLMHAKPNEAHAQQYQHTQNQLSLAQKEKEKQY

                           3070      3080      3090      3100      3110      3120
Nc  ------------------------------------------------------------
Af  ------------------------------------------------------------
At  ------------------------------------------------------------
Dd  ------------------------------------------------------------
Sc  ------------------------------------------------------------
Ca  ------------------------------------------------------------
Su  ------------------------------------------------------------
Dm  ------------------------------------------------------------
Sp  ------------------------------------------------------------
Hs  ------------------------------------------------------------
Mm  ------------------------------------------------------------
Cf  ------------------------------------------------------------
Bt  ------------------------------------------------------------
Ce  FQAKNLQASQANAQQQQQRFGDVVAGNVAGYGRPYGQQQLGASDQMGTSQLPGASTSRMN

                           3130      3140      3150      3160      3170      3180
Nc  ------------------------------------------------------------
Af  ------------------------------------------------------------
At  ------------------------------------------------------------
Dd  ------------------------------------------------------------
Sc  ------------------------------------------------------------
Ca  ------------------------------------------------------------
Su  ------------------------------------------------------------
Dm  ------------------------------------------------------------
Sp  ------------------------------------------------------------
Hs  ------------------------------------------------------------
Mm  ------------------------------------------------------------
Cf  ------------------------------------------------------------
Bt  ------------------------------------------------------------
Ce  QGSSNPQGGMQSYQQQQPVLGQPGPIQTGQSTQQQIPAQSQQQYNSGRPQMHTTPTKNDM

                           3190      3200      3210      3220      3230      3240
Nc  ------------------------------------------------------------
Af  ------------------------------------------------------------
At  ------------------------------------------------------------
Dd  ------------------------------------------------------------
Sc  ------------------------------------------------------------
Ca  ------------------------------------------------------------
Su  ------------------------------------------------------------
Dm  ------------------------------------------------------------
Sp  ------------------------------------------------------------
Hs  ------------------------------------------------------------
Mm  ------------------------------------------------------------
Cf  ------------------------------------------------------------
Bt  ------------------------------------------------------------
Ce  SARAPSGAMGQIANRMGHGTNPQGYGSTGQNVPGGYQQGQQQSGQGSYPQAQQQQPNQYS

                           3250      3260      3270      3280      3290      3300
Nc  ------------------------------------------------------------
Af  ------------------------------------------------------------
At  ------------------------------------------------------------
Dd  ------------------------------------------------------------
Sc  ------------------------------------------------------------
Ca  ------------------------------------------------------------
Su  ------------------------------------------------------------
Dm  ------------------------------------------------------------
Sp  ------------------------------------------------------------
Hs  ------------------------------------------------------------
Mm  ------------------------------------------------------------
Cf  ------------------------------------------------------------
Bt  ------------------------------------------------------------
Ce  GSNQQVGQQAQQQQQQPLNQNVSQSQSAAQFGRPSQDSAYQQSGYNQTGNQSYQRPDQQQ

                           3310      3320      3330      3340      3350      3360
Nc  ------------------------------------------------------------
Af  ------------------------------------------------------------
At  ------------------------------------------------------------
Dd  ------------------------------------------------------------
Sc  ------------------------------------------------------------
Ca  ------------------------------------------------------------
Su  ------------------------------------------------------------
Dm  ------------------------------------------------------------
Sp  ------------------------------------------------------------
Hs  ------------------------------------------------------------
Mm  ------------------------------------------------------------
Cf  ------------------------------------------------------------
Bt  ------------------------------------------------------------
Ce  QGAQQNQWSGSNQAQNQLRSQQQAQQPLQQPQQSQQFQQPAQQAKNPMAQSAQYGGFGGQ
```

Med12

```
                                3370      3380      3390      3400      3410      3420
Nc    ------------------------------------------------------------
Af    ------------------------------------------------------------
At    ------------------------------------------------------------
Dd    ------------------------------------------------------------
Sc    ------------------------------------------------------------
Ca    ------------------------------------------------------------
Su    ------------------------------------------------------------
Dm    ------------------------------------------------------------
Sp    ------------------------------------------------------------
Hs    ------------------------------------------------------------
Mm    ------------------------------------------------------------
Cf    ------------------------------------------------------------
Bt    ------------------------------------------------------------
Ce    QQGYDQQQQGQIAPQQAQNPQASQSYGQQQTQQNRYGMGSSGYTANSGGSSNILNQSMEE

                                3430      3440      3450      3460      3470      3480
Nc    ------------------------------------------------------------
Af    ------------------------------------------------------------
At    ------------------------------------------------------------
Dd    ------------------------------------------------------------
Sc    ------------------------------------------------------------
Ca    ------------------------------------------------------------
Su    ------------------------------------------------------------
Dm    ------------------------------------------------------------
Sp    ------------------------------------------------------------
Hs    ------------------------------------------------------------
Mm    ------------------------------------------------------------
Cf    ------------------------------------------------------------
Bt    ------------------------------------------------------------
Ce    SGLNQGFSGASSNASSQQGGSSQMQQSGYGMPGNQMQMQQNQKQQVQRGMPTGMGQTNMG

                                3490      3500      3510      3520      3530      3540
Nc    ------------------------------------------------------------
Af    ------------------------------------------------------------
At    ------------------------------------------------------------
Dd    ------------------------------------------------------------
Sc    ------------------------------------------------------------
Ca    ------------------------------------------------------------
Su    ------------------------------------------------------------
Dm    ------------------------------------------------------------
Sp    ------------------------------------------------------------
Hs    ------------------------------------------------------------
Mm    ------------------------------------------------------------
Cf    ------------------------------------------------------------
Bt    ------------------------------------------------------------
Ce    QSGMGQSGMGQTGMSRSGLGGGIGQQGQQSQQPQQPQVSQQQNQRGMNPGAQLPPYSTGQ

                                3550      3560      3570      3580      3590      3600
Nc    ------------------------------------------------------------
Af    ------------------------------------------------------------
At    ------------------------------------------------------------
Dd    ------------------------------------------------------------
Sc    ------------------------------------------------------------
Ca    ------------------------------------------------------------
Su    ------------------------------------------------------------
Dm    ------------------------------------------------------------
Sp    ------------------------------------------------------------
Hs    ------------------------------------------------------------
Mm    ------------------------------------------------------------
Cf    ------------------------------------------------------------
Bt    ------------------------------------------------------------
Ce    QQHQPQQSQISQQQQQQDQYRRMQAAQMQQQPTAQGQQNRMGMPSQQQSGAAYSNQMQFQ

                                3610      3620      3630      3640      3650      3660
Nc    ------------------------------------------------------------
Af    ------------------------------------------------------------
At    ------------------------------------------------------------
Dd    ------------------------------------------------------------
Sc    ------------------------------------------------------------
Ca    ------------------------------------------------------------
Su    ------------------------------------------------------------
Dm    ------------------------------------------------------------
Sp    ------------------------------------------------------------
Hs    ------------------------------------------------------------
Mm    ------------------------------------------------------------
Cf    ------------------------------------------------------------
Bt    ------------------------------------------------------------
Ce    GVRQGQQGMGGMGGSGQQQPQTQPHGSNQYYQQQQDQRMQQQPQQPGQQQQHGYGMGQYP

Nc    --------
Af    --------
At    --------
Dd    --------
Sc    --------
Ca    --------
Su    --------
Dm    --------
Sp    --------
Hs    --------
Mm    --------
Cf    --------
Bt    --------
Ce    NQQPPNQY
```

Med13

```
     1        10        20        30        40        50        60
Dm  ------------------------------------------------------------
Pt  ------------------------------------------------------------
Dd  ------------------------------------------------------------
Cf  ------------------------------------------------------------
Bt  ------------------------------------------------------------
Hs  ------------------------------------------------------------
Su  ------------------------------------------------------------
Gg  MASKALPSGDNSSQFSSSLDVQKIEEGKIGSLSLVLFSSSAKGITSFFKRALSSSVLDEL
At  ------------------------------------------------------------
Sp  ------------------------------------------------------------
Dr  ------------------------------------------------------------
Ce  ------------------------------------------------------------
Ca  ------------------------------------------------------------
Nc  ------------------------------------------------------------
Af  ------------------------------------------------------------
Sc  ------------------------------------------------------------

              70        80        90       100       110       120
Dm  ------------------------------------------------------------
Pt  ------------------------------------------------------------
Dd  ------------------------------------------------------------
Cf  ------------------------------------------------------------
Bt  ------------------------------------------------------------
Hs  ------------------------------------------------------------
Su  ------------------------------------------------------------
Gg  SLLNCSEVVRRSRAVCGERGRSGRELSASVPLLEEQQQAALRNIKMFCISVLCCLHWEQS
At  ------------------------------------------------------------
Sp  ------------------------------------------------------------
Dr  ------------------------------------------------------------
Ce  ------------------------------------------------------------
Ca  ------------------------------------------------------------
Nc  ------------------------------------------------------------
Af  ------------------------------------------------------------
Sc  ------------------------------------------------------------

             130       140       150       160       170       180
Dm  ---------------------------MTHQNHQTNGASLEDCHTNFYALTDLCGIKWRK
Pt  ----------------------------MPNSLSALLLDITGESVVAELTGIKWRRYNFG
Dd  ------------------------------------------------------------
Cf  ----------------MPVNQHLFGVTPGNKRDQLEQNLEEMFTECAADLTGIKWKRYVW
Bt  ------------------------MEGHNLVVANFLVTESITLTTVQADLTGIKWKRYVW
Hs  -------------------------MSASFVPNGASLEDCHCNLFCLADLTGIKWKKYVW
Su  ------------------------------------------------------------
Gg  LSASLESVSVTDWKNMENSTFAAGHVCLLKHVLQGAVLKWQCFQSASHRPGADSKLSITC
At  ------------------------------------------------------------
Sp  ------------------------------------------------------------
Dr  ------------------------------------------------------------
Ce  -------------------------MKLQKRPEWNNGGSLEDCISNVYALLELPGIKWKC
Ca  ------------------------------------------------------------
Nc  ------------------------------------------------------------
Af  ------------------------------------------------------------
Sc  ------------------------------------------------------------

             190       200       210       220       230       240
Dm  FVNGERPNASSDPLADPILRSYSRCIQADMLCVWRRVQSTKTDHADPNALTFEMTTSTKV
Pt  GHGDCGPIISAPAQDDPILLSFIRCLQANLLCVWRRDVKP--------------------
Dd  ------------------------------------------------------------
Cf  QGPTSAPILFPVTEEDPILSSFSRCLKADVLGVWRRDQRP--------------------
Bt  QGPTSAPILFPVTEEDPILSSFSRCLKADVLGVWRRDQRP--------------------
Hs  QGPTSAPILFPVTEEDPILSSFSRCLKADVLGVWRRDQRP--------------------
Su  ------------------------------------------------------------
Gg  CLGVIAPHTACVLLRSHRRAYDVLVPDMYQLSDSLQFPCLFFLGAGLLLVLGQHQTIVER
At  ------------------------------------------------------------
Sp  ------------------------------------------------------------
Dr  ------------------------------------------------------------
Ce  YRTKPNAPRGVALTADFVLKAYSKCLLDGILCTWRRKPLPPTDNK---------------
Ca  ------------------------------------------------------------
Nc  ------------------------------------------------------------
Af  ------------------------------------------------------------
Sc  ------------------------------------------------------------

             250       260       270       280       290       300
Dm  HPPLS----------------LAAAKELWIFWYGEEPDLSELVDAELLRVAANQALWNGT
Pt  -----------------------DCKELWIFWWGDEPNLVGVIHHELQVVEEG------L
Dd  -------------------------MMGTKVPSYNNKQQSGSNAGGNVNNNNNGPNINNP
Cf  -----------------------GRRELWIFWWGEDPNFADLIHHDLSEEEDGVWENGLS
Bt  -----------------------GRRELWIFWWGEDPNFADLIHHDLSEEEDGVWENGLS
Hs  -----------------------GRRELWIFWWGEDPVLADLIHHDLSEEEDGVWEDGLS
Su  ------------------------------------------------------------
Gg  KDTASVLLLDAAGSIFVGDGGQPPLLKVSASLWLWDTPVNPVTNVLVGDSNAARLVVNAG
At  ------------------------------------------------------------
Sp  ------------------------------------------------------------
Dr  ------------------------------------------------------------
Ce  -----------------------ETPKLTNFSNDAPKELWVFWYDDEPALLQKNCEGLDN
Ca  ------------------------------------------------------------
Nc  ------------------------------------------------------------
Af  ------------------------------------------------------------
Sc  ------------------------------------------------------------

             310       320       330       340       350       360
Dm  WKG--------ALTYECRSLLFKALHNLMERFVLTKDIVRFGKWFVQPCTSSDRLFG---
Pt  WENGLS---YECRT-----LLFKAIHNLLERCLMDKNFVRIGKWFVRPYEKDEKPVN---
Dd  NNPNNTNSLPTTSGYNSSRFSVPNLDTDNFMTNIISMGVNKVGWALYRSNSNNNNNNINN
Cf  YECRT--------------LLFKAVHNLLERCLMNRNFVRIGKWFVKPYEKDEKPIN---
Bt  YECRT--------------LLFKAVHNLLERCLMNRNFVRIGKWFVKPYEKDEKPIN---
Hs  YECRT--------------LLFKAVHNLLERCLMNRNFVRIGKWFVKPYEKDEKPIN---
Su  ------------------------------------------------------------
Gg  CIMASPVPKPAAAHPLCAALYWAEKSLHSCFFSFGKFQFESAVELILKNCCYLLNALYLL
At  ------------------------------------------------------------
Sp  ------------------------------------------------------------
Dr  ------------------------------------------------------------
Ce  DDELSSANQMNIVSYEVRTIIFKALHVVLERDLTKDGFVRFGRWFTVPYDARENYLHFTY
Ca  ------------------------------------------------------------
Nc  ------------------------------------------------------------
Af  ------------------------------------------------------------
Sc  ------------------------------------------------------------

             370       380       390       400       410       420
Dm  RSSQHLSFSFTFFVHGDTVCAS-IDLREHPAVRPLTKEHLTEAAAAFAAASSPPGSNGSA
Pt  -KSEHLSCAFTFFLHGESNVCTSVEIAQHQPIYLINEEHIH-----------------MA
Dd  NSSSSSSSNTSNNINSNTLVNDPILLAYLKTIELGVPCLWRKTRSKKPINNSSNNSNITS
Cf  -KSEHLSCSFTFFLHGDSNVCTSVEINQHQPVYLLSEEHITLAQQS--------------
Bt  -KSEHLSCSFTFFLHGDSNVCTSVEINQHQPVYLLSEEHITLAQQS--------------
Hs  -KSEHLSCSFTFFLHGDSNVCTSVEINQHQPVYLLSEEHITLAQQS--------------
Su  ------------------------------------------------------------
Gg  SPSEHLSCAFTFFLHGESNVCTSVEIAQHQPIYLINEEHIHMAQSS--------------
At  ------------------------------------------------------------
Sp  ------------------------------------------------------------
Dr  ------------------------------------------------------------
Ce  PSHSPAIRFN-FFVHGSSTICASVQAQRQPTLITLARRHLECKTKRVVVVVGPWSMRGQL
Ca  ------------------------------------------------------------
Nc  ------------------------------------------------------------
Af  ------------------------------------------------------------
Sc  ------------------------------------------------------------
```

Med13

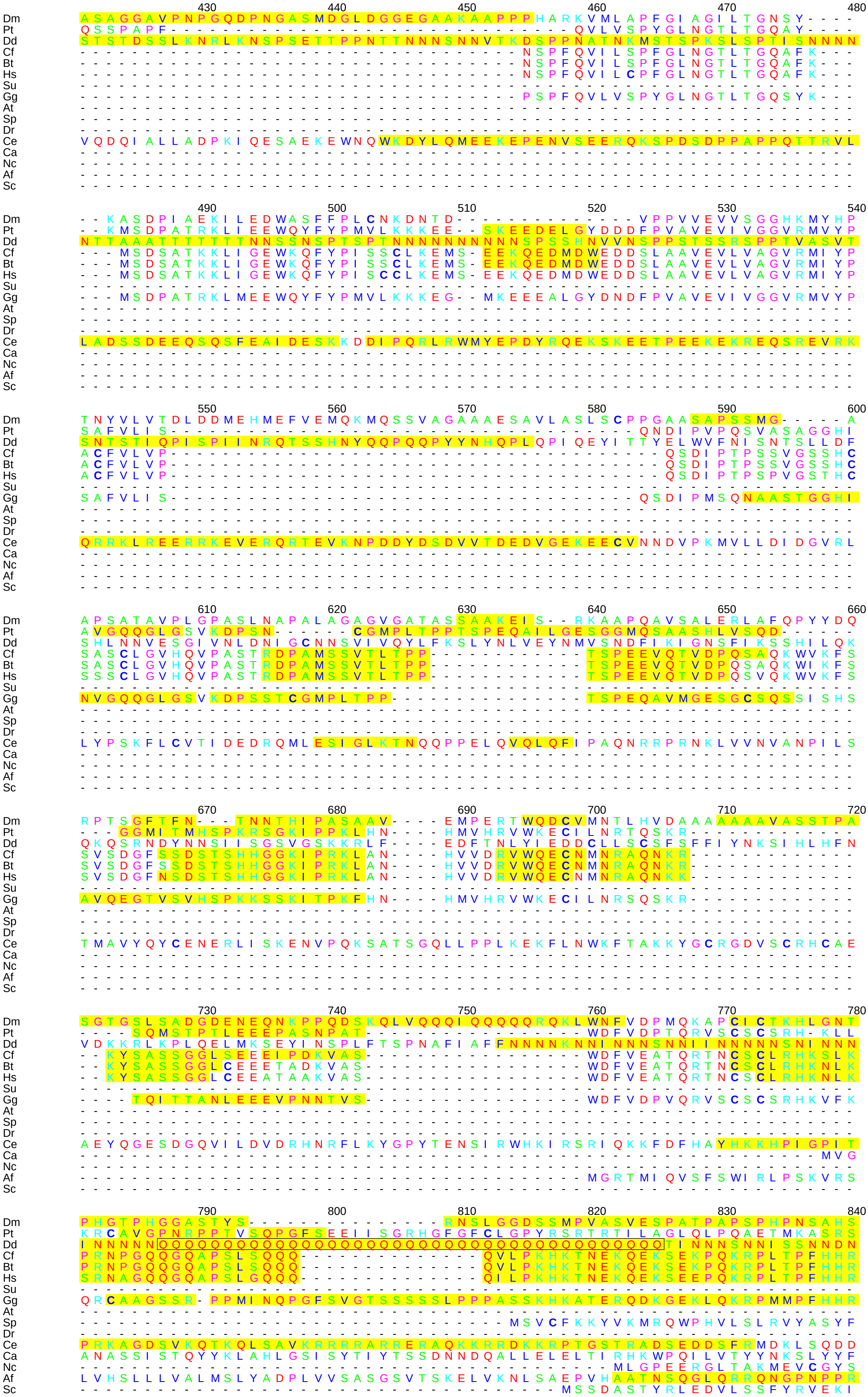

Med13

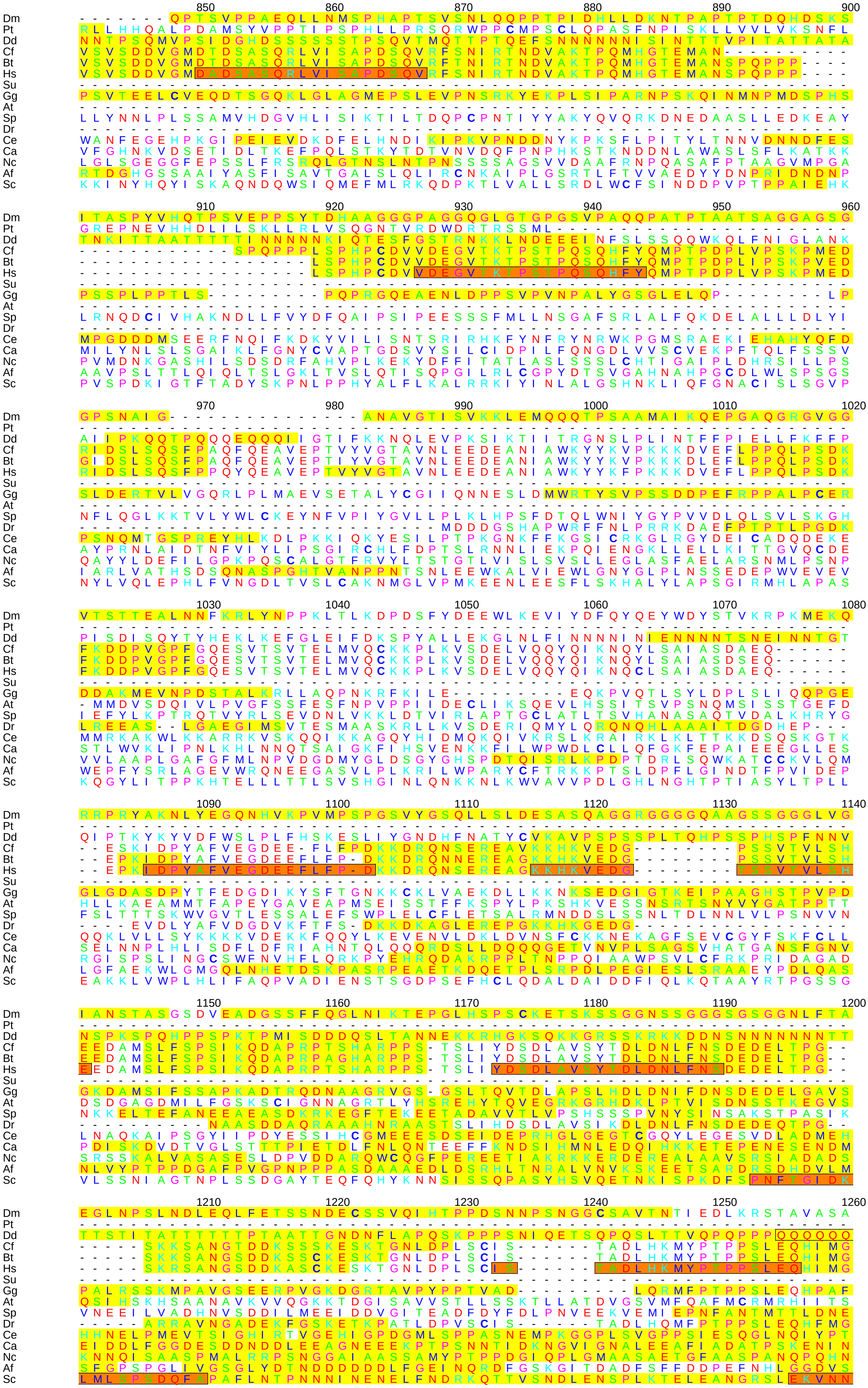
850 860 870 880 890 900 910 920 930 940 950 960 970 980 990 1000 1010 1020 1030 1040 1050 1060 1070 1080 1090 1100 1110 1120 1130 1140 1150 1160 1170 1180 1190 1200 1210 1220 1230 1240 1250 1260
Dm Pt Dd Cf Bt Hs Su Gg At Sp Dr Ce Ca Nc Af Sc

Med13

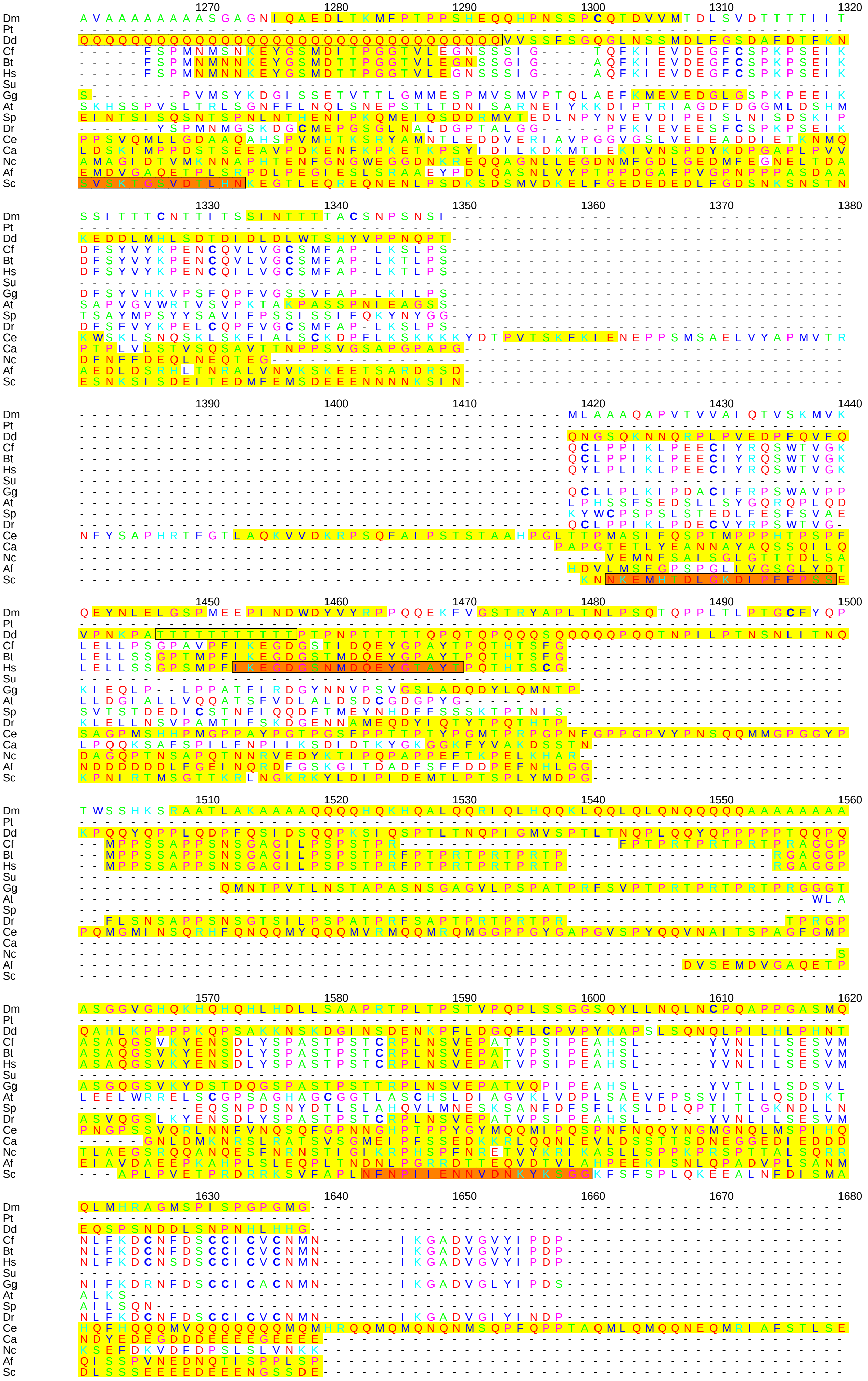

Med13

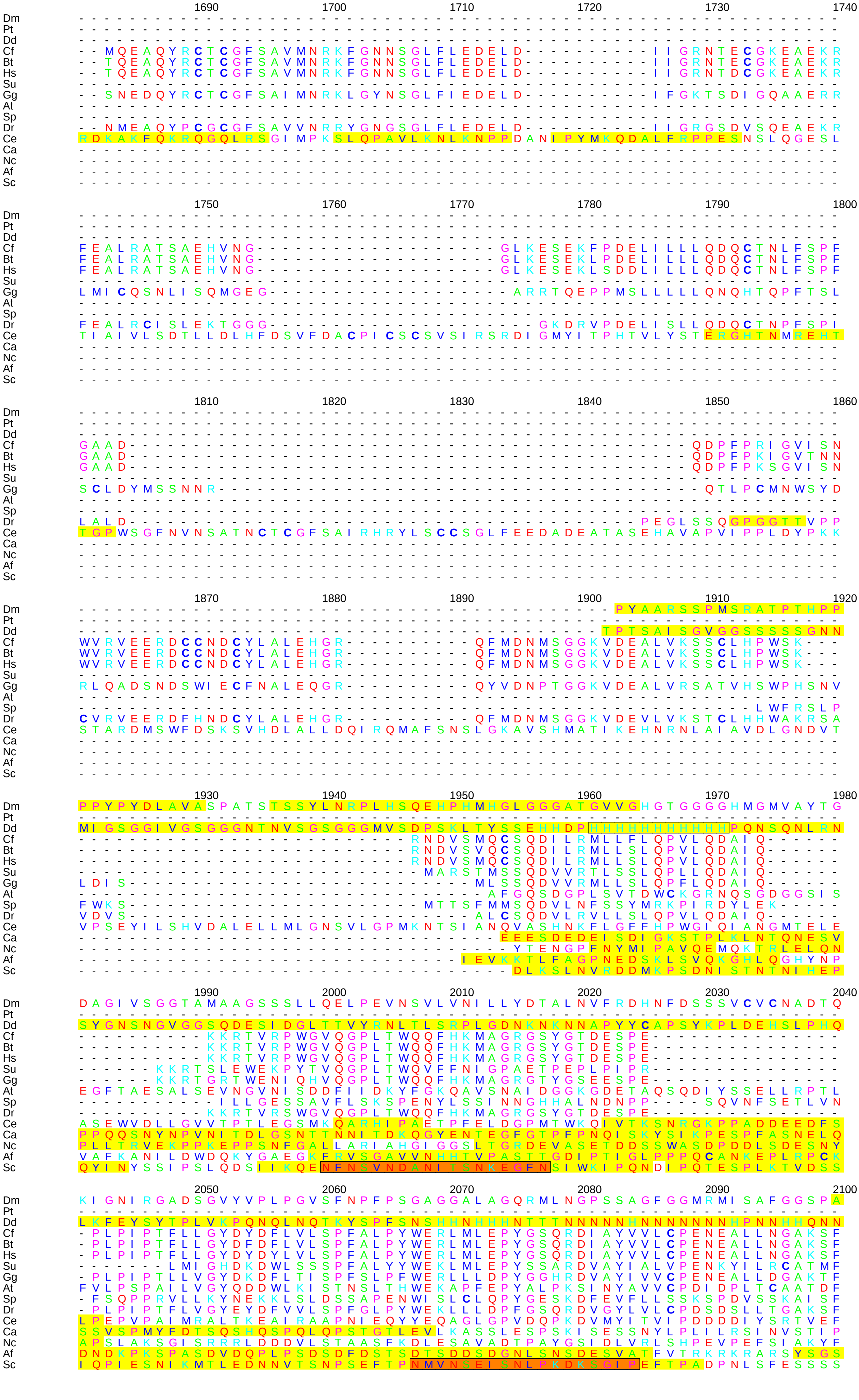

Med13

```
                                            2110      2120      2130      2140      2150      2160
Dm   SASMPGAGSGHGHGPNGGSNSSSCTPPSSNPHITGYVDDDPVECTCGFSAVVNRRLSHRA
Pt   ------------------------------------------------------------
Dd   NHHHLNITSSLFQPSNIQNVNSLPNIGSQKIMSISPPITPPMKGGHMIGTDINKNNNKKN
Cf   FRDLTAIYESCRLGQHR--PISRLLTDGIMRVGSTASKKLSEKLVAEWFSQAADG-----
Bt   FRDLTAIYECCRLGQHR--PISRLLTDGIMRVGSTASKKLSEKLVAEWFSQAADG-----
Hs   FRDLTAIYESCRLGQHR--PVSRLLTDGIMRVGSTASKKLSEKLVAEWFSQAADG-----
Su   FKELTAVYEGCRLGRHAP--IAKTFQDGIMKIGKKYAQKLSNEPVDVWFTDVAHLP----
Gg   FRDLSAVYEMCRLGQHKP--ICKVLRDGIMRVGKTVAQKLTDELVS---EWFNQPWGTEE
At   FQQLGTVYETCRLGTHLPQSLGNQMEKDVGRLSSSGFVLLDCPQSMKIESNNTSLLGSLS
Sp   FYDVQLAYENCKLGKLN--LSETSINERVMGFSTNIN--------------ETDNYDDNE
Dr   FRELTAVYEVRDLWSSIT------------------------------------------
Ce   MDTLTRTYEKMRLGRHIPFPVSTGTATRFREAFKSCQSQFPFQEQQHLAYTNQTLRNMPQ
Ca   SLYLMNNLISSKLLPSFTISDDDLENDLDITKSNEMIVKLGFLKEFLDFMSPNIIFDLGL
Nc   ADPEPARLQVSFSDTNFITIAQILTEQVATGSLQIAPRILSRKPQDVRRTLAKAVRYSMQ
Af   SVNLPQQKPLARSEQSIATSRTDDLVFLGNFLSTFSDWSMTGYFSAIQKQLAPVLIRKDA
Sc   LPFLLRHMPLASIPDIFITPTPVVTISEKEQDILDLIAEQVVTDYNILGNLGIPKIAYRG

                                            2170      2180      2190      2200      2210      2220
Dm   GLFYEDEVEITGIADDPGRNKQPTLLSIIQSLSRKNQNKQGPGETSSALDKIGAGGLPNG
Pt   ------------------------------------------------------------
Dd   NDEDENLEILSTIKQKDVNGLDSNLSSSALLSFFDQAINIKTTPPRSPVLSGYSGGGSNM
Cf   --NNEAFSKLKLYAQVCRYDLGPYLASQPLDSSLLSQPNLVAPASQSLITSPQMTN--TG
Bt   --NSEAFSKLKLYAQVCRYDLGPYLASQPLDSSLLSQPNLVAPTSQSLITPPQMTS--TG
Hs   --NNEAFSKLKLYAQVCRYDLGPYLASLPLDSSLLSQPNLVAPTSQSLITPPQMTN--TG
Su   -----EADKLKLYAQFCRAYVAPFLNQEQPDASMFRTSRHHSSSSGASSGSNVPGSGSYP
Gg   CDNHS---RLKLYAQVCRHHLAPYLATLQLDSSLLLPPKYQTPAPASQTQTIPGNTGPVP
At   DYFLSLSNGWNVNSYLKSLSKALKGLKLGSGLYTNQKEGSGSPCVVVYIVCPFPDPSAVL
Sp   TTQSDTATSYEQLASVCVNELSGKNVLFFYFLEDDSEKLLKACQHFICVKDSIKRLGDNK
Dr   ----------NSQQPKQTKGAAPYLATQPLDSSLLIQPTPVPSSTQSSSSQSSGSASSNS
Ce   TLADTPDPVNSDPKVWSRRAKRKLDFEEELKLPEAGSETIDESDDDEENEEVSKEENNEL
Ca   MKNDESDYYINGVNDLLLNLDPGISSDWILNSLSKVFPCTYPMKLIELLYDFKSLELEDQ
Nc   GLRKALPRSLADAIECSLKPYADVQDVPLVALAQPQRIQAKPMGSEMSTRNIYPIPTPHI
Af   QIEVAQLLVEQVTQSCLDHKVDGILNVSEFESKTYSLRTFLEDTAFMGEIERLDLNGFVS
Sc   VKDCQEGLITTTMLQLFSTFDRLNGNDTISKFYNMKQPYVFVKKHHELIKVKHDSQPFIK

                                            2230      2240      2250      2260      2270      2280
Dm   QLEQLGHAVFDLLLDQCSIIQTSSSSVHRALQSHRRRMSRQRRIFGNNGAPTASLASIAN
Pt   ------------------------------------------------------------
Dd   NNGINGNSGFIGNGNSGFNTISSYQQNNISIEESLDLAFTIQQMTITNYDSTFGFSIDNS
Cf   NANTPSATLASAASSTITVTSGVAMSSSVATANSTLTTTSTSSSSSSNLNSGVSSNKLPS
Bt   NANTPSATLASTVSSTMTMSSGVAMSSSVATANSTLTTTSTSSSLSSNLNSGMSSNKLPS
Hs   NANTPSATLASAASSTMTVTSGVAISTSVATANSTLTTASTSSSSSSNLNSGVSSNKLPS
Su   SSSSSSSYVVGSSNTSGSQGGAMHEQGSQGSDTSSASMPAPSSTPSRPANRQDS------
Gg   SNSTCLSTSGAPPTGSSFNSTPNGGTTSLPAGSSSSSGSSVPQLSASSATPGVNQMSATP
At   RTIVESSIALG-------------------------------------------------
Sp   FEDK--------------------------------------------------------
Dr   QTSMASGGSALNNNNNSAASNSGSSLPGSTNNTPSSSSSGPQGSSIQSAKPSSFPPFGSM
Ce   IDSEDVMDEVPKGFYEREGILRVGAPMEPSRSPHTVTNTPEFDNMTKYLSDENGFVSRLR
Ca   LDSQLNFLNEIADEEDFVGPKALYRKLKALEWDSFSLNELNKTSFEKYKNVMEKLSAEN-
Nc   EIRRYETPFSMLPTAISFWESLGLSPAKGAKDIVSVCLFPNVDG----------------
Af   LQENSGLSPPLTATMSRQSSQRKENGKSSILKLAPPHLRIRRGKDYLEVLPPAISFWETF
Sc   FLNFRPPNGIKN------------------------------------------------

                                            2290      2300      2310      2320      2330      2340
Dm   VLEFMDAHDVISLALEQSRLAFENQRMDNMMDF---------------------------
Pt   ------------------------------------------------------------
Dd   PLIKQSIDYYLENWLKIYTETGKVFNHHSLQFL---------------------------
Cf   FPPFGSMNSSATGSMSTQGN----------------------------------------
Bt   FPPFGTMNSSATGSMSTQAS----------------------------------------
Hs   FPPFGSMNSNAAGSMSTQAN----------------------------------------
Su   ------------------------------------------------------------
Gg   TPGFSGNIGSGQNTSTGGNAADRSQGS---------------------------------
At   ------------------------------------------------------------
Sp   ------------------------------------------------------------
Dr   GVQGNSSQSGALGQQAVTQN----------------------------------------
Ce   LYLQQMEDLVHFALSENPEAFDRRGYRYQLAVEGRLKRQKHKMDIIEERLRFEASKDFDE
Ca   ------------------------------------------------------------
Nc   ------------------------------------------------------------
Af   GLEPAYGSKDIYAYCIHPQTAAEAANDFLERLG---------------------------
Sc   ------------------------------------------------------------

                                            2350      2360      2370      2380      2390      2400
Dm   -----------------------HGNGSSSSHQQQQLTAFHAPPPALRHKLAGIGAGRLT
Pt   ------------------------------------------------------------
Dd   -----------------------TEESISDALDTVIDPLTTNTILETTVKTLFTFSNEIF
Cf   ------------------------------------------------------------
Bt   ------------------------------------------------------------
Hs   ------------------------------------------------------------
Su   ------------------------------------------------------------
Gg   ------------------------------------------------------------
At   ------------------------------------------------------------
Sp   ------------------------------------------------------------
Dr   ------------------------------------------------------------
Ce   QSDRKEPLEENDPEITPGIVISNNPVESWIDDELEEKRQQRQKENEQYPPEAQQAPSPVP
Ca   ------------------------------------------------------------
Nc   ------------------------------------------------------------
Af   ------------------------------------------------------------
Sc   ------------------------------------------------------------

                                            2410      2420      2430      2440      2450      2460
Dm   VHKWPYLPVGFTRSNKEIVRTMNAIQPMLQNAFHCKSRGGSGSKDASSYNTVSGPLTWRQ
Pt   ------------------------------------------------------------
Dd   KKIFPLSSFPLSIKEFCHLPSITLNNINNNIDLLNSQTYQNTNNSSSGGGIESQQYFSGS
Cf   -----------TVQNGQLGGQQSSALQTAGISGESSSLPAQPHPDVSESTMDRDKVG---
Bt   -----------TVPNSQLGGQPPSALQTAGISGESSSVPTQPHPDVSESTMDRDKVG---
Hs   -----------TVQSGQLGGQQTSALQTAGISGESSSLPTQPHPDVSESTMDRDKVG---
Su   --------------------------------------------KDGSSASSSENKENLP
Gg   --------------------------TGPGGDTEAGQNLSQQQQEGQEGVTERERIG--I
At   -----------------------------SVIQSDRDRRSILNSQVARAFSSSTAVDEAS
Sp   ------------------------------------------------------------
Dr   --------------------------SSLSGDNSAINSQTQGPSEPPESTMEREKVG---
Ce   AGMIHIPETLSKEERAVQPTLEMILADPATVAPINAQNIVWKQRDTRVPNFPSSNQPPVA
Ca   ------------------------------TVSDDDYFKLPMVKTRILKNGNIVNLNNIG
Nc   ---------------------------LRDSASAFLQRIRSVYESLKLGTFDILPTVPDL
Af   --------------LLYSSCNLGQHTRASRSTGFDRGMCSWNIGAPGEFNYHYIMQSLKS
Sc   ------------------------------FKSLLLSSSFKEDCLSFAPTLSQTYINQEL

                                            2470      2480      2490      2500      2510      2520
Dm   FHRLAGRASGQCEPQPIPSVVVGYEKDWISVAPHSIHYWDKFLLEPYSYARDVVYVVVCP
Pt   ------------------------------------------------------------
Dd   SGLNNSNDRNSMIPMDNSDDTSDLMSMDDHNSKNLITGTSLISTTASGAGAAITTNLINQ
Cf   IPTDGDSHAITYPP---AIVVYIIDPFTYENK-DESTNSSNVWTLGLLR-----------
Bt   IPTDGDSHAITYPP---AIVVYIIDPFTYENK-DESTNSSNVWTLGLLR-----------
Hs   IPTDGDSHAVTYPP---AIVVYIIDPFTYENT-DESTNSSSVWTLGLLR-----------
Su   KVTALETVTPRDSEQPPAVVVYIVDPFRRGEDAEEEGEVSVATLGLMHCFAEIFDKLQVS
Gg   PTEPESADSNAYPP---AVVIYMVDPFTYTAD--EESASTNFWLLSLMR-----------
At   ISHIPVLSGFSVPKLVLQVVSVDSIFRITSPSFNELVILKDTAFSVYNKARRISRGMPND
Sp   --------SLRICTIPNSIFDSPNSHTTNSNSFFTKVSLDIYNNDPLLMDG---------
Dr   VPTDGDSHAITYPP---AIMIYIVDPFSYEEAEPGTSQSSKWTLGLLR------------
Ce   FEAAGSPDTDAYSTLPHVIVVYVVNPFSYGPE-GHSALHMRIAILAFIR-----------
Ca   LKFWKYLNFSPVKKQKDFQILLIAETHRNTASYATEFLDQIIQNYKECNFGTISKVNLST
Nc   EDGLLSYFATQQPAVRDEWAVSPFNPMSCLANALKSVQMTEKNYVVFFVYP--------D
Af   ICDKLVTALLKSPPTRDSVVVYIINPFSHAAALADICSAFYDLFQKYID-----------
Sc   GFCELLKLTNEDPPGLMYLKAFDKNKLLLLAAQIVSYCSNNKNSIKNVPPILIILP----
```

Med13

```
          2530      2540      2550      2560      2570      2580
Dm  DNEHVVNCTRSYFRELSSTYEMCKLGKHTPIRGWDGFLQVGAARNNVPADRETTPLDDWL
Pt  ------------------------------------------------------------
Dd  NYEEFEWLPIPKIIVGYKEDWLEVPISTIKFWDKAQLEPYSPKKNITYFVLCPDTTAMRM
Cf  -------CFLEMVQTLPPHIKS-----------TVSVQIVPCQYLLQPVKHEDR------
Bt  -------CFLEMVQTLPPHIKN-----------TVSVQIVPCQYLLQPVKHEDR------
Hs  -------CFLEMVQTLPPHIKS-----------TVSVQIIPCQYLLQPVKHEDR------
Su  LQRNIAFQIIPLHQVLQVANSEVSSRFLN-------------------------------
Gg  -------CYTEMLDNLPENMRN-----------SFILQIVPCQYMLQTMKDEQVFY----
At  AFFSSSLPSRSSSALTPMNSISGIWKDCGGSRMTGSTHPRDGEIDVSLRTSGWDTS----
Sp  ------------------------------------------------------------
Dr  -------CYLEMLQALPTHIRN-----------SVFVQIVPCQYLLQPVRSEER------
Ce  -------AFNSIMCKIPYEKRP-----------QLQLEIVGMEGMDNVAKPIPDYFNDAK
Ca  VETRSDLEPISDGLVLVHKEHDQLYNDFYIQTNKKLISLVELIKLDLINKTNNFEFGKPL
Nc  DRPNSVVESCAAFHDLYERYKQCMADIGKPVTSELVLQLVPLDCIASDTTVTVMSP----
Af  -----------EVENQQAGQAS-----------ELVLQIVPFHFIMSTESLVVPP-----
Sc  ---LDNATLTELVDKANIFQVIKNEVCAKMPNIELYLKVIPMDFIRNVLVTVDQ------

          2590      2600      2610      2620      2630      2640
Dm  RTLEHAALAEQIRRYAVAFIHQLAPYLSRVPNDKTLLNPPDGSGNSHSKGGSSCSSNSSS
Pt  ------------------------------------------------------------
Dd  HVPAFFSDLSCIYETNHLGNHVTQQQSQVQQAAAAAVAASQPTQQSSQHQSQQQQQQAAQ
Cf  ---------------------QIYTQHLKSLAFSAFTQCRRPLP---TSTNVKTLTGFGP
Bt  ---------------------QIYTQHLKSLAFSAFTQCRRPLP---TSTNVKTLTGFGP
Hs  ---------------------EIYPQHLKSLAFSAFTQCRRPLP---TSTNVKTLTGFGP
Su  --------------------------QLKSVAFSVFSQCRRFQSPTIPGRSLTGFGPAAD
Gg  ------------------------IQHLKSLAFSVYCQCRRPLP---TQIHIKSLTGFGP
At  ---------------------------TSWQIPRSGGLSCDPNRNGDFYLNDEIFYLFEP
Sp  ------------------------------------------------------------
Dr  ---------------------HLYAQHLKSLAFSVFTQCRRPLP---TSTNVKSLTGFGP
Ce  IPFDLLNDRPIRVERPGE----SVQGELARSLSIAVYTHRVFFPDVYKSASARCMTAFGP
Ca  LLFFIDFNDSSNSNVQVCKIFRNFKVALTTHQLPLVDIFTKIVPSSLLVKKVHHETALKV
Nc  -------------------------VDYMKLCLETYDRCTLFGG----------------
Af  ------------------------QAQYLSLALEVYSRCPPKSLQS--------------
Sc  ---------------------------YVNVAISIYNMLPPKSVKFTHIAHT--------

          2650      2660      2670      2680      2690      2700
Dm  VSGLPGGDLPTDNIKLEPGTEPQVQPMETNEIKQEPGVGKGGTAAGETKPTLILGDPLGM
Pt  ------------------------------------------------------------
Dd  QAARAAAAAAAAQKNSSPFYNGIYYIPSAANTEFNPQTFINSYLLTCIQFGDYLKELSTK
Cf  GLAMETALKSP-----------------------------------------------DR
Bt  GLAMETALKSP-----------------------------------------------DR
Hs  GLAMETALRSP-----------------------------------------------DR
Su  LDRTIITKN-------------------------------------------------AE
Gg  AASIEMTLKNP-----------------------------------------------ER
At  LFILSEPGS------------------------------------------------VER
Sp  ------------------------------------------------------------
Dr  GLALDTSLRNP-----------------------------------------------DR
Ce  GSQLMNTINKIEALNKDSFARMAKRSKTTLDTMDMYRHPGMIQAQQSTEKKNYIAYRVPS
Ca  FSNYKLTKISMNLYN-------------------------------------------EC
Nc  ----------------------------------------------------------PM
Af  ------------------------------------------------------------
Sc  ---------------------------------------------------------LPE

          2710      2720      2730      2740      2750      2760
Dm  GETLEDINPSAIVLYVVNPFTFASDSCELERLALIALLRCYAELLKAVPDSVRSQMNIQI
Pt  ------------------------------------------------------------
Dd  YSENNCMVLYIVNPFSDLSQISLSNQNQNQNQNSNSNESQQQQQQQSNNKQKQTQTNEDI
Cf  PECIRLYTPPFILAP-----VKDKQTELG---ETFGEAGQKYN-----------VLFVGY
Bt  PECIRLYTPPFILAP-----VKDKQTELG---ETFGEPGQKYN-----------VLFVGY
Hs  PECIRLYAPPFILAP-----VKDKQTELG---ETFGEAGQKYN-----------VLFVGY
Su  HNEDRLYAPPFILAPIKDKQTELGESFGG-------------------LRQACGELFCSY
Gg  PSPIQLYSPPFILAP-----IKDKQTELG---ETFGEASQKYN-----------VLFVGY
At  GVSPTFTSLGSESSKPIPEDGGRGSGPGMNSMEGITSGSSSQGDVSQLEGKAIPSLHCCY
Sp  --SLKRREPAFLLKKPLLSTLNYQLKDINPRSSALGEYALHVTYTTVEEHLLICNWNDSY
Dr  PECLRLYTPPFILAP-----TKDKQTELG---ETFGEASQKYN-----------VLFVGY
Ce  NIAVLAPPPMVYQMDEKGKAIMNQLDEQT--------------------------LFISY
Ca  PNDLANKSIVKNLFTTIVKDPPSKIQFKFMNSSYRDNSSNDD-----------IFLHLAY
Nc  PAPAIVLEPQVLR---------TIDFKLNTNPS--PNLLQENS-----------CMHVAY
Af  --CLLNCAPPMLIAEPVPKTINFKLAPDS-----SSPLQEAK------------SLHIAC
Sc  KVNFRTMQQQQMQ--------QQQQQQQQQQNNSTGSSSIIYYDSY---------IHLAY

          2770      2780      2790      2800      2810      2820
Dm  ISLESVMELGPCGNRKRFSDEIRCLALNIFSQCRRHLVHAQSVKSLTGFGTAANMEAFLK
Pt  ------------------------------------------------------------
Dd  NHIRNSYLFMSSCFQTIVRDITCNQSLSITIQTIPIEQILFDDHPSIPVSKELAFSVFNK
Cf  CLSHDQRWILASCTDLYGELLETCIINIDVPNR--ARRKKGSARRFG----------LQK
Bt  CLSHDQRWILASCTDLYGELLETCIINIDVPNR--ARRKKGSARRFG----------LQK
Hs  CLSHDQRWILASCTDLYGELLETCIINIDVPNR--ARRKKSSARKFG----------LQK
Su  CLSHDQRWLLVSCTDSKGELMETQIINVDVQNS-----------------------KRRK
Gg  CLSHDQRWLLASCTDLHGELLETCIVNIDLPNR--SRRSKLSARKIG----------LQK
At  GWTEDWRWLVSIWTDARGELLDTHIFPFG------GISSRQDTKG------------LQC
Sp  GEFETERRYFLQDLEIEDALQQILEVTFTFLNSMHMDWIVIVMKIGEMSDAEYLFWDQAI
Dr  CLSHDQRWLLATCTDLYGELLETCIISVDVPNR--ARRKKGSARKMG----------LQK
Ce  CLVGTD-FLVATATDAQGKLIDNCISNIKPRRQSNQVYRYRNKTQILDG--------MGK
Ca  ERSIDNSWFVASWSDPLGRVVHVKSWYCSDSVSKSEKSTYRG--------------DIMD
Nc  AQSVDERWITAAWTDYRGTRQMTTSYCLG-------RRDRPLTRSIT--------EIIQQ
Af  SRSYDQRWVSVSWTDNTGTFQRTLSY-CLRFQNSNATRNILEVR--------------TE
Sc  SRSVDKEWVFAALSDSYGQGSMTKTWYVG-----NSRGKFDDACN--------------Q

          2830      2840      2850      2860      2870      2880
Dm  TKDEPNRRAYK--MYTAPFVLAPMHERNDKTDFSRSA--GSMHGQNEHRYSVMYCNYCLS
Pt  ------------------------------------------------------------
Dd  CRRISTISPMN--FSINMNNINNMIGNNNSNSNNSSG--WYYQLFN----KIYEPLFILS
Cf  LWEWCLG------LVQMSS-------LPWRVVIGRLG--RIG----------------HG
Bt  LWEWCLG------LVQMSS-------LPWRVVIGRLG--RIG----------------HG
Hs  LWEWCLG------LVQMSS-------LPWRVVIGRLG--RIG----------------HG
Su  KVSVRNIALYC--LWDFLMGVISKASIPWRLVIGRLGRLG------------------QG
Gg  LWEWCIG------IVQMTS-------LPWRVVIGRLG------------------RLGHG
At  LFVQVLQQGCQ--ILQACSSPDNGSFKPRDFVITRIG---NFFELEYQEWQKAIYSAGGP
Sp  IPENLQGNVSL--TVGYCSAEHGPGSTSKVFSRIPYS-----------------------
Dr  LWEWCLG------LVQMTS-------LPWRVVIGRLG------------------RIGHG
Ce  LWSFILG------IMASET-------KNWRLVVGRLG------------------RIGHG
Ca  IMSITDD------IWSIST-------DLFKVLNDELG------------------SFGGK
Nc  IWEATMD------HIHFAR-------VHWRVVVTKCS------------------PMEQQ
Af  IWAATKMIMEKTQ-------------ARWKIIIANTEP------------------VDQD
Sc  IWNIALN------LASKKFG-------KICLILTRLNG-----------------ILPDD

          2890      2900      2910      2920      2930      2940
Dm  EDQAWLLATATDERGEMLEKICINIDVPNRARRRKAPARYVALKKLMDFIMGIISQTSQM
Pt  ------------------------------------------------------------
Dd  NESSSSSSSTISTSTSASTSTSTSNTTTKNQQQQSQSQQSQQQSQQPKQLSTPTTPTTNS
Cf  ELKDWSSLLS--------------------------RRNLQSLSKRLKDMCRMCG-----
Bt  ELKDWSCLLS--------------------------RRNLQSLSKRLKDMCRMCG-----
Hs  ELKDWSCLLS--------------------------RRNLQSLSKRLKDMCRMCG-----
Su  ELKDWSMLLS--------------------------RKNLAHYSRKLREQCSMCS-----
Gg  ELKDWSILLG--------------------------ECSLQTISKQLKEVCRMCG-----
At  EIKKWPIQLR--------------------------RSAPSGIATNSNGSSLQQQ-----
Sp  ------------------------------------------------------------
Dr  ELRDWSILLS--------------------------RRNLQSVSRRLKENCKLCG-----
Ce  EFRAWTHLLN--------------------------KTSLLRYSGSLKDICGACR-----
Ca  KFLVLTRINSIIPDVELVH-----------------WKRLSLKHKEISLIVVSVR-----
Nc  EIEYWASLQQSE--------------------TYANVSLTLLTVDTNPSLQLIPP-----
Af  EVDTWTSLVEQYNTTKPFP-----------------SDLTILYVNTTPDLYLEPP-----
Sc  ELMNWRRLSGR-------------------------NIHLAVVCVDDNSKISFIDEDKLY
```

Med13

```
          2950      2960      2970      2980      2990      3000
Dm  WRLVIGRIGRIGHSELKSWSFLLSKQQLQKASKQFKDMCKQCTLMYPPTILSACLVTLEP
Pt  ------------------------------------------------------------
Dd  VSNSPSLQPQPLQTPNQLQPHQQEQKQQQQIPIIHFSYMLSDDNKWISCTIVDEQGELLE
Cf  --------ISAADSPSILSACLVAMEPQGSFVIMPDSVSTGSVFGRSTTLNMQTSQLNTP
Bt  --------ISAADSPSILSACLVAMEPQGSFVIMPDSVSTGSVFGRSSTLNMQTSQLNTP
Hs  --------ISAADSPSILSACLVAMEPQGSFVIMPDSVSTGSVFGRSTTLNMQTSQLNTP
Su  -------VPGTLDFPCILSACLVSTEPEPGLSIMADMVAADKCSKNSQRDQPSCQLHTPD
Gg  --------ISSADSPSILSACLVAMEPQGSFVVMPDAVTMGSVFGRSTALNLQSSQLNTP
At  ----------DLSLIQERASSTSTLYSSHSKQSTFVKGSMGQSAGRKQIMGG-QTISGTP
Sp  ------ASSTVIRNNSSHELSLVAFIREMAMPVPNDEFKKISTILARGYLALDEDESYLP
Dr  --------ISASDTPSILSACLVAMEPQGSFVIMPDSVSTGSVFGRSTTLNMQTSQLSTP
Ce  -------SPSVIGTPAILSACLITLEPEPSIRIMP--------EFHDQELSTKKSFLFQT
Ca  ------QTPRIVGSNESE----DLGPKSNDYAPTPMIQDKDIFFGFKQTFSTSNTSSPSA
Nc  ------AQRIQIPSAALSSNSPASTPQASVLSPDQSGLGPPTPIGTGSSNSANAATDNNN
Af  -----PQPLSMSVFNPQISSTPVATPNPSGNAFSPEQPGNAPTPPSGGNAPTPTEIPPDA
Sc  PSFKPIYKDTRFGGRMDMTRLYDYEIRDIDQDIHGIVFQHPFPLAHSQHRCAIRSGALIK

          3010      3020      3030      3040      3050      3060
Dm  DAKLRVMPDQFTPDERFSQISMQNPLATPQDVTCTHILVFPTSAVCAPFTRQFQNEPQVD
Pt  ------------------------------------------------------------
Dd  NKLIPLAYSQIIGDYIWKDSFQNAWFFLTEIIGLSLSGQPCQVVIGKFGIISMQEYNDWK
Cf  Q------------DTSCTHILVFPTSASVQVAS--------ATYTTENLDLAFNPNNDGA
Bt  Q------------DISCTHILVFPTSASVQVAS--------ATYTTENLDLAFNPNNDGA
Hs  Q------------DTSCTHILVFPTSASVQVAS--------ATYTTENLDLAFNPNNDGA
Su  D-------------ASCTHIMVFPTSATKQAAATNMPQDQADLTFNSELRLDDEDPFLVD
Gg  Q------------DASCTHILVFPTSATIQVAPANYPNEDGFSPTNDDMFDLPFPDDMDN
At  RGLFQWVHSISFASISLDHSLHFVLPAELVS------ADAHLHGYLHNYVGGGQSSTGMS
Sp  LLSIHLLISRNHDPYLMLNLILKHYLSMIYLQFRTYVSFSSLPLHISTVLYQKQLLQFMA
Dr  Q------------DTSCTHILVFPTSAFVQVNS--------SNYTHENIDIAFNPGNGEG
Ce  P------------DLSCTHILTFPVGTEIN------------------LEVQDQTADTKA
Ca  S---------------GGALVTSPNSLSLHSPQQFLNAPANFLS--PQDLIAPTSLG---
Nc  N-------------SNNNNNTTTTTTTTTDTDADSTLVDQLDMTWAVIAAHRLNNSTSLT
Af  ES-------------------------------ECLLTDICDESWGIILSHCLNSSPHLT
Sc  FKKCDGDTVWDKFAVNLLNCPHSDSTQLLETILEEFRNLAALNVWYGLSDGEDGHIPWHI

          3070      3080      3090      3100      3110      3120
Dm  DDFLTFEEEGNEDFSDADIGDLFWDTHMDRVSNHGSPGRMDD------------------
Pt  ------------------------------------------------------------
Dd  TTIIHFEDNLEVIKQHQQQSQSQNYSSTIDQIYKIENVIFLNLTLNNNIKSFLTEYLNDS
Cf  DGMG------IFDLLDTGDDLDPDIINILPASPTGSPVHS-------------PGSHYPH
Bt  DGMG------IFDLLDTGDDLDPDIINILPASPTGSPVHS-------------PGSHYPH
Hs  DGMG------IFDLLDTGDDLDPDIINILPASPTGSPVHS-------------PGSHYPH
Su  DLLLNIFMNTSNMMPDPSNIQLSDLDQEPIPTDGGPAPAAQG------------------
Gg  DIRILITGNLHSSPNSSPVPSPGSPSGIGVGSHYQHSRSQ--------------------
At  SVN--YIEGFTP------------VKSLGSTAFSYMMIPS-------------PNMRFLH
Sp  SDITHPVTS---------------------------------------------------
Dr  DPLFYSIFDLLEQENDLVDPD---IINILPASPTTSPVHS-------------PGSQYPH
Ce  DEN---WEFGDLDIMEGLDGDTEIMKDLGLETPSSAAIRQTG------------------
Ca  ----------ISGAKPGSSGIDPEVVLENQDNEVYGVIPKVP------------LPSFNS
Nc  ELNQALASGYLVKRGGPKLEDVPVTMEVNVIHCEG-------------------------
Af  DYRPALASGYLLRRKGPTDADGAFAMNVN-------------------------------
Sc  LAVKKMMNTLVHTRVKIANTSAATVHTATSSSIILSDK----------------------

          3130      3140      3150      3160      3170      3180
Dm  -----------NRSWQSAGGNNFKCTPPQEVEEVGSLNQQPISVGYMVSTAPTGRMPAWF
Pt  ------------------------------------------------------------
Dd  KLSNSNNIKSVSPLSGQSFAIYPESPIMYTYNFPMNDNNDDYNSILNNNNNNILPPLSTS
Cf  GGDAGK-------GQGTDRLLSTESHDEVTNILQQP-----LALGYFVSTAKAGPLPDWF
Bt  GGDAGK-------GQGTDRLLSTESHDEVTNILQQP-----LALGYFVSTAKAGPLPDWF
Hs  GGDAGK-------GQSTDRLLSTEPHEEVPNILQQP-----LALGYFVSTAKAGPLPDWF
Su  ----------PGNPTTRQQPNQEPGIPAEPEDVLRGILQQPLALGYFISTAKTGPLPKWF
Gg  ----------------GERLLSREAPEELKQQ--------PLALGYFVSTAKAENLPQWF
At  P------------SPLQLPTCLTAESPPLAHLLHSKGYAIPLSTGFVVSKA----VPSMR
Sp  ------------------------------------------------------------
Dr  GGDGSKVTEASSFGQSTDRMESHEEAPNILQQP--------LALGYFVSTAKAGPLPDWF
Ce  ---------------GPSMFFSEDSSSIEIQNQ-------PLASGYYISTAPAPELPAWF
Ca  PTRFCMK-------TGYLMMQVNQPSEEEYKDDIKKTYLVYEINLLSCSNYWNLDVLMKL
Nc  ------------INIPQPHQYPRVVYEQLLREMLSHFRGLGTLARARGVVDRETDVRPWH
Af  ---------------LIYSQKPASLYENLLREIIARYRDLATLARARGTRTVQQNTFPWH
Sc  ------------------------------------------------------------

          3190      3200      3210      3220      3230      3240
Dm  WSACPHLEDVCPVFLKTALHLHVPSIQSADDILNSTN-------------------AHQS
Pt  ------------------------------------------------------------
Dd  FVITSPSNLSGSVLTPTTTNSNVINNNQSKKEWPFTYMLSIVGIQSLQHQQQQQQQQQQQ
Cf  WSACPQAQYQCPLFLKASLHLHVPSVQSDELLHSKHS-------------------HPLD
Bt  WSACPQAQYQCPLFLKASLHLHVPSVQSDELLHSKHS-------------------HPLD
Hs  WSACPQAQYQCPLFLKASLHLHVPSVQSDELLHSKHS-------------------HPLD
Su  WSACPEAENSCPVFLKAALHVHNPSEQPDDILQTKHS-----------------------
Gg  WSSCPQAQNQCPLFLKASLHHHISIAQTDELLPARNS---------------QRVPHPLD
At  KDSRINVKEEWPSVLSVSLIDYYGGYDNAHDKILQGIVK-----------------QGGG
Sp  ------------------------------------------------------------
Dr  WSACPQAQNQCPLFLKASLHLHVSSVQSDELLHSKHS-------------------HPLD
Ce  WATCPSAKRHSPVHLKSSLHIHISEVKNDDIAMESTKEKEKDK-------------EKDE
Ca  IMLQYKQMIALNDILCMNPIVG--------------------------------------
Nc  VAVAEKAVRALYLLM---------------------------------------------
Af  IATALRAQELLSYVL---------------------------------------------
Sc  ------------------------------------------------------------

          3250      3260      3270      3280      3290      3300
Dm  GNDHPLDSNLTADVLRFVLEGYNALSWLALDSNTHDRLSCLPINVQTLMDLYYLTAAIA-
Pt  ------------------------------------------------------------
Dd  QQQQHPNIINHQELIRSITRSYHRLSYLNISPRYPDRLSILPIHFSISKRMSRTVCLISP
Cf  SNQTSD-------VLRFVLEQYNALSWLTCDPAIQDRRSCLPIHFVVLNQLYNFIMNML-
Bt  SNQTSD-------VLRFVLEQYNALSWLTCDPAIQDRRSCLPIHFVVLNQLYNFIMNML-
Hs  SNQTSD-------VLRFVLEQYNALSWLTCDPATQDRRSCLPIHFVVLNQLYNFIMNML-
Su  ---HPLDSDRTPEVLRYVLETYNSLSWLTVDPSSGDRRSCLPVHMVVLVQLYNTIASLQ-
Gg  SKTTSD-------VLRFVLEQYNALSWLTCNPATQDRSSCLPVHFVVLTQLYNAIMNIL-
At  TKETRDFEVESHLILESIAAELHALSWMTVSPAYLDRRTALPFHCDMVLRLRRLLHFADK
Sp  ------------------------------------------------------------
Dr  SNQTSD-------VLRFVLEQYNALSWLTCDPATQDRRSCLPIHFVVLNQMYNFIMTML-
Ce  KDAHPLESRQTEEVLRHVLESYNALSWLNLNRQTGDRYSCLPIHIQHLLRLYHSVARLLA
Ca  --------------------------ELDILGTHSRNNAIVPWHINAVGKLLDYLVHIYV
Nc  ------------------------------------------------------------
Af  ------------------------------------------------------------
Sc  ------------------------------------------------------------

          3310      3320      3330
Dm  -------------------------------------
Pt  -------------------------------------
Dd  LNPIPPPILFNRCPKLTPNSKQSPSPINSPHLNSNNT
Cf  -------------------------------------
Bt  -------------------------------------
Hs  -------------------------------------
Su  -------------------------------------
Gg  -------------------------------------
At  EVSRIPDKTGV--------------------------
Sp  -------------------------------------
Dr  -------------------------------------
Ce  -------------------------------------
Ca  DKD----------------------------------
Nc  -------------------------------------
Af  -------------------------------------
Sc  -------------------------------------
```

Med14

```
   1        10        20        30        40        50        60
Hs ---------------------------------MAPVQLENHQLVPPGGGGGGSGGPPSA
Su ------------------------------------------------------------
Ce ------------------------------------------------------------
Pt ------------------------------------------------------------
Bt -----------------------------------MAPVQLENHQLVPPGGSGSGGPASA
Dm -----------------------------------------------MAPTPLPLEQMPG
Dd ----------------MDQNQQQQQQQQQQQQQQQQQQQQQQQQQQQQQQQQQQQQQQQP
At ------------------------------------------------------------
Sp ------------------------------------------------------------
Ca ----------------------------------------------MNQHQDQSSMPNGK
Af ------MPGVIMDNATTGRPGHAPDTQTPSNGDNLRNGSFHINGAAKGDKDHDPDKESYA
Sc MTTTIGSPQMLANEERLSNEMHALKNRSEQNGQEQQGPVKNTQLHGPSATDPETTATQKE
Nc ---------------------------------------MSGSSVMMNGGGASFMDVDSQ

            70        80        90       100       110       120
Hs PAPPPPGAAVAAAAAAAASPGYRLSTLIEFLLHRAYSELMVLTDLLP-------------
Su ------------------------------------------------------------
Ce --MLQAQNPLPEVPANCGPPTIPLNVLLDFAIQHVYHEITVLAELMQR------------
Pt ------------------------------------------------------------
Bt PAPPPPGAAVAAAAAAAASPGYRLSTLIEFLLHRAYSELMVLTDLLPR------------
Dm VGGGGGGYLPAAQEGGPRINTMSMSVLIDFIIQRTYHELTVLAELLPR------------
Dd PNGMMPMNGEQTTPIIPQNRNISLSLVIHRLVEQSYNSLLGLTEGLPKAN----------
At -------------MAELGQQTVDFSALVGRAAEESFLSFKELVDKSK-------------
Sp --------MEPPAIPHITEGFYPLPEIVETFSHHVLQELVSLAEVLPSMSN---------
Ca QQHEVTELSSPPEIPHITANMIPLSNIIKYYTQEAYKQLTTAIENLSMN-----------
Af GKPRIDGHRALPEIPHITQGFFPFSTLVNRSVQQCWNELSDLITELAAIHVSPHSSVPLL
Sc SLEMVPKDTSAATMTSAPPPALPHVEINQVSLALVIRNLTVFTMKELAQYMKTN------
Nc PPGGTKFNDLPDEIQHITADIMPLSLLLTRLSQWTHTKLQDEIAYLASKPLPQGVLNG--

           130       140       150       160       170       180
Hs ----------------RKSDVERKIEIVQFASRTRQLFVRLLALVKWANNAGKVEKCAM-
Su ------------------------------------------------------------
Ce -------------KTNDQGEQERKMSLVHFAHATRSQFLKLVALVKWIRISKRMDVCYVS
Pt ------------------------------------------------------------
Bt -----------------KSDVERKIEIVQFASRTRQLFVRLLALVKWANNAGKVEKCAMI
Dm -----------------KTDMERKVEIYNYAARTRHLFTRLNALVKWGNSVSKVDKSSQ-
Dd -------------------DLERKKAIVDYLDGTREKFLRLMVLIKWSEHVPTLTKANN-
At --------------STELSDTEKKVSLLKYVAKTQQRMLRLNALAKWCKQVPLINYFQD-
Sp --------------------VEKKKKILDWLLRSRAFTMRLLVLARWVHLSPSVHRCID-
Ca --------------VNEESDIKRKKYFLNVIINLRQDFIKVYTLIKWASISKDVSKFID-
Af PVNG------KSPGNQSPENVQKKLRILDFAHAKRAEFIKLLVLSQWSRRARDVSKLID-
Sc -----------VHTQANEPNSAKKIRFLQLIIFLRTQFLKLYVLVKWTRTIKQNNFHVL-
Nc ----TTNHQSADNHDTSQESLEKKVHLLNFLQDMHSKWVKTLVITEWSRKAAQVGALID-

           190       200       210       220       230       240
Hs ----------------ISSFLDQQAILFVDTADRLASLAR--DALVHARLPSFAIPYAID
Su ------------------------------------------------------------
Ce LIRSISIEKKTLIFQSIDYLLDLQSQYFIDTADRLVAMT--RGDLELARLPEYHIAPAID
Pt ------------------------------------------------------------
Bt S-----------------SFLDQQAILFVDTADRLASLAR--DALVHARLPSFAIPYAID
Dm ----------------IMSFLDKQNMLFVETADMLARMS--RETLVRARLPNFHIPAAVE
Dd ----------------IIDILNLEDSYLREAADLLINTQ---FSLVNARAPIYDVPTAID
At ----------------LGSTLSAHDICFTQAADSLFFMH---EGLQQARAPVYDVPSAVE
Sp ----------------VVAFLQGQKFCFQNLVHVLQDIR---YQLSFARLRNSDLVTALD
Ca ----------------LLNWFRIQEFHFENLIFQLNALTG----YNAAKLPNSDIITALE
Af ----------------LQNFIRARHQAFVDALQRVGEMKR---DLVQAQVANPDLQTALE
Sc -----------------IDLLNWFRTTNMNVNNCIWALKSSLNSMTNAKLPNVDLVTALE
Nc ----------------IRVHLLTKQAHYQELFWELIRMK---QDLHWAKVPGPDLKTALE

           250       260       270       280       290       300
Hs VLTTGSYPRLPT------CIRDKIIPPDPITKIEKQATLHQLN---QILRHRLV------
Su ------------------------------------------------------------
Ce VLVLGTYNRMPS------KIKEAFIPPAKITPREQKLVTSRLN---QLIESRLS------
Pt ------------------------------------------------------------
Bt VLTTGSYPRLPT------CIRDKIIPPDPITKIEKQATLHQLN---QILRHRLV------
Dm VLTTGTYNRLPT------CIRERIVPADAITPAEKRQTLLRLN---QVIQHRLVTGKLLP
Dd VLTTGTYQRMPTN-------IKRVIPPPPLKPTQIESALERLN---DIIKYKLFIS----
At ILLTGSYQRLPK-------CLDDVGMQSSLDEHQQKPALRKLEVLVRSKLLEIT------
Sp ILSTGTSLRLAN----APTSKLYMLSESPLSTKQILQTLHALN---MLIRIRLS------
Ca VLYHGRPKLPSYN----------YIKSDNLSPQKILETLNDLN---LVLMTRFALMDN--
Af ILSKGRLESLADLG---------YKSSKLLTARGALRRLHKIN---RIISARLALHD---
Sc VLSLGRPNLPTHN-FKLSGVSNSMDMVDGMAKVPIGLILQRLKDLNLTVSIKIALMN---
Nc VLTTGQASWMPELG---------YIEPPPITWEEKEAFIDNIN---TMLSVRLT------

           310       320       330       340       350       360
Hs TTDLP------PQLANLTVANGRVKFRVEGEFEATLTVMG----DDPD---------VPW
Su ------------------------------------------------------------
Ce RLSSG----IPPNIKEIHINNGLATLLVPGEFEIKITLLG----ETEMT---------KW
Pt ------------------------------------------------------------
Bt TTDLP------PQLANLTVANGRVKFRVEGEFEATLTVMG----DDPD---------VPW
Dm ------------QMREFRIRNGRVTFEVKHEFSVSLTVMG----DNPT---------VPW
Dd --------DVPKEFQPITVSDGKAHIFVDDEYEAYLTIDG----GSEKS---------NW
At ---------LPKEITEVKISKGTVTLSVDGEFKVLVTLGY----RGHLS---------MW
Sp -----LYEIIPTPFQHFTIANGRCTFTVPNEFSVSLTTNS----QDPK----STGISFQW
Ca ----------IPKRFDYEIKDGRAYIRVSNEFEVSITVGNDLIIDNPEEYYKS-----PF
Af --------LIPHPFRTYRVHDGRVTFVVRGEFELDLSIGAESELS-------------QF
Sc ---------IPKPLNSYHIKNGRIYFTVPNEFEIQLSTVN----RQSP-----------L
Nc ---FDEHEKLPAAFRDYNIANGRVTFKVKGEFEVDLTIGD----EDFEQ---------QF

           370       380       390       400       410       420
Hs RLLKLEILVE--DKETGDGRA---LVHSMQISF----------------------IHQLV
Su ------------------------------------------------------------
Ce TLLNIKILV--EDYELGMGLP---LVHPLQLNQ----------------------LHGVL
Pt ------------------------------------------------------------
Bt RLLKLEILVE--DKETGDGRA---LVHSMQINF----------------------IHQLV
Dm RLLDIDVLVE--DKETGDGKS---LVHPLQVNY----------------------IHQLI
Dd VILSLNLFVYS--KRNLNGEGPIKVAYDNKMKY----------------------VLDRV
At RILHLDLLVG--------ERSGPIKLEVTRRHI----------------------LGDDL
Sp IVVDFQFHLP--DFSSTPAKYR----VFIELHLN------------------------EE
Ca YFIDFKFLFG-ANPESGLITFNDDKISTKLPTS---------------------LHKKLE
Af FFVDIRFLYS--------------PSSNIPKGR---------------------MSNEID
Sc FFVDLKLLFN-----TEAEQTVSAVTEATSTNGDSENNEENSSSNGNNLPLNKPRLEKLI
Nc WFIDFRFLFT--------------PAPAELSHG---------------------VRTFLE

           430       440       450       460       470       480
Hs QSRLFADEKP-LQDMYNCLHSFCLSLQLEVLHSQTLMLIRER------WGDLVQVERYHA
Su ------------------------------------------------------------
Ce QSRMNVSLNP-IKEAFSFLHSFCVSLQLDVLFCQT-SRLAAG--RLRDN--ITIEKYDPK
Pt ------------------------------------------------------------
Bt QSRLFADEKP-LQDMYNCLHSFCLSLQLEVLHSQTLMLIRER------WGDLVQVERYHA
Dm QARLVENPNA-LSEVYNCLHYFCQSLQLEVLYTQTLRLNYER---LDDNNITVEEYVPGV
Dd QNRIISSAQP-LFELHNIVRNVLKTSLFNNNNINHNNNINTTNSSY--------------
At ERRMSVAENP-FTILYAVLHELCVAIVMDTVIRQVRALLQGR----WKDAIRFDLISDTG
Sp IAAAFVLQKPILPLIYNILHKFCLYQRLNLLSQQTFQLSRES----WLGHLRGVYDEKPP
Ca KLVNQTLLTRGLQGLYELLHKYSNSFKIYLLAKQFQTLLINS--RWRGN-FQINYQTNKS
Af AKINEKLRDSGLTGCFNFLHGLVLTNKIHILFKQAIELAKGL--------WSETLRVELL
Sc NEILLKSNDP-LLSLYNFLHKYVLTLQLYMVHREFLKLANGG--KFSKSNLIHNYDSKKS
Nc NKVNTILGDQGLAGCYNYLHEFVLTQKIIEFRRQATELAMGR--------WVDTLKVEKL
```

Med14

```
           490       500       510       520       530       540
Hs  GKCLSLSVWNQQVLG-------------RKTGTASVHKVTIKIDE---------------
Su  ------------------------------------------------------------
Ce  ERVLVVGYWVKRSKS--------RRLTVGQVKCDAQYRVQIYEDP---------------
Pt  ------------------------------------------------------------
Bt  GKCLSLSVWNQQVLG-------------RKTGTASVHKVTIKIDE---------------
Dm  KLTVSYWRDLKSELG---------------------YRLTVQSDPS--------------
Dd  ------------------------------------------------------------
At  -TTPANQEGEADSVSL---RTPG-------------MKLFYWSDS----DKNSGPFIKIE
Sp  --RLRLYYWPQLNVKK---EGKPGKIGHYIHIFVNTQPISAFERTLSSKRSSCEYDHFLL
Ca  --LIVINYWSQHYLSRNWKSFIELGIDSHSNLNYRWFKNGQYCFGDQGNNELDKIFHLQR
Af  HRTLVIQYWTLKPGPK------------------SWIEIGVKSGNGDADSQ---------
Sc  --TITVRYWLNGKMDS--KGKITIGIQRTTESLILKWDNQSASRAKNMPVIYNNIVSNIE
Nc  NRAMSIQYWLKPPHG---------LEGPSSQTAKSWILLGVWSGKGVEGDLDSKPSSYIS

           550       560       570       580       590       600
Hs  -----------NDVSKPLQIFHDPPLPASDSKLVERAMKIDHLSIEKLLIDSVHARAHQK
Su  ------------------------------------------------------------
Ce  -------------NDKLGGLKVRHFPHAPQLGRLDSGAG--MLSIDRLLSETYVVRCKER
Pt  ------------------------------------------------------------
Bt  -----------NDASKPLQIFHDPPLPASDSKLVERAMKIDHLSIEKLLIDSVHARAHQK
Dm  -----------EIGRPLAVVHVPSLGAKESAEVADRAVRSEHLSMERLIVHTVYIRSVSR
Dd  ------------------------------------------------------------
At  PGSDLQIKCSHSTFVIDPLTG-----KEAEFSLDQSCID-----VEKLLLKAICCNRYTR
Sp  LVEWHHDGIVEHVPLDDHMDAQHLLLLITQKHAQLILEQIRKELHPNIFSEHVGGGLKIH
Ca  RNSNNGVTTSSNTISTSAMIAGIRRSTELSNETAIVDDNDNDNNDTNNTGETENEDLNVD
Af  ------------GLGVPCLGLRWMRDGQ-EVNSRDIEFDPEDLSMECLLRSVIALHISYL
Sc  GILDEIMFNHARIIRSELLARDIFQEDEENSDVLLFQLPTTCVSMAPIQLKIDLLSGQFY
Nc  LRWFRDNKEVKDFDIPLNVETISAEKLLTAVIASHVEYILRSISSKLLSKPRFAQKHARL

           610       620       630       640       650       660
Hs  LQELKAILRG------------------------FNANENSSIETALPALVVPILEPCGN
Su  ------------------------------------------------------------
Ce  LMRLRR---------------ILEAAEPR-------LEVKMTGISAPSLSLALLPDTSSK
Pt  ------------------------------------------------------------
Bt  LQELKAILRG------------------------VNANENSSIETALPALVVPILEPCGN
Dm  LSDLKLEFQAFLK------------------------DVDFNLQGTPAILTVPVLSPCLR
Dd  ------------------------------------------------------------
At  LLEIQKELLRNTRICRTPSDVILQALLDEPGIEGDNMVDSKERVEPEVLRVR-----AYG
Sp  VFDNEIIVKVNSVTGRLVLSSSASPLSPPRHLRAAEKNIALNTQPPAQILNRLYFFCIQT
Ca  LILNVVVNQHAKSIMSEIYSQLLTRFSETDVSMVSPHQLLLQISPKKSTVFAINPLTGFF
Af  LSSAYG------------------------------ILSEYSLFSSGTLSSHAILNVTEP
Sc  FRNPTPLLSNYASKINRAEGPEELARILQQLKLDKIIHVLTTMFENTGWSCSRIIKIDKP
Nc  DLEISKEEPQNSSLAVQLFDNDKAVIKIEPLTGFFTMFPRSPVFLKGQMKLNTSSNVAEE

           670       680       690       700       710       720
Hs  SECLHIFVDLHSGMFQLMLYGLDQATLDDMEKSVN--------DDMKRIIPWIQQLKFWL
Su  ------------------------------------------------------------
Ce  DEMMTVSVNSFCGKVLCNVHILSAEHEDVLAFG---KALYSSQCSAHTIQMYLRKLRVAL
Pt  ------------------------------------------------------------
Bt  SECLHIFVDLHSGMFQLMLYGLDQATLDDMEKSVN--------DDMKRIIPWIQQLKFWL
Dm  AEQIHITIDTHTGMFRCHVPKHLDCPIMEEMQDCLN-------GDRSKLPALLSELRFWI
Dd  ------------------------------------------------------------
At  SSFFTLGINIRTGRFLLQSSKSILTSSILEEFEDALNQGSISAVDAFINLRSKSILHFFA
Sp  QLLEVAQCAELHAVQGYYSFPYLTFSKGKWRKDGDSLWVLAYNVESNSWSVRLLNAAGQT
Ca  YFIDPTPIQTYITKKINSPPPTLSQVSIKQSFIPESDMISYVIDQIIQLRLEVFNKEVNT
Af  GECQLSVQLTGSRHLRVSIEPMSGAVILSATPGLLERFESDASLDRSTIDDLVARVSRLR
Sc  IRTQVNTGGESVVKKEDNKYAIAGNSTTNSDVSLLLQRDLFIRLPHWPLNWYLILSIISS
Nc  GANLMEQLRFDHAVKDLNSRVRSIGWFVCRPPITQEETKTIVYSDASGSREAFKAVWLRK

           730       740       750       760       770       780
Hs  GQQRCKQSIKHLPTISSETLQLSNYSTHPIGNLSKNKLFIKLTRLPQY-----YIVVEML
Su  ------------------------------------------------------------
Ce  VIERYRRSVKALPVREVQEAELLPFAKECLGDAPAQRIILQFLRSEDY-------YLLVT
Pt  ------------------------------------------------------------
Bt  GQQRCKQSIKHLPTITSETLQLSNYSTHPIGNLSKNKLFVRLTRLPQY-----YIVVEML
Dm  THRRCDKTLQHLPATATETLPFLVQPDQEILQPGRHKIYVKLHRHPNIVLVIIHLVVQLK
Dd  ------------------------------------------------------------
At  AIGKFLGLEVYEHGFGINKVPKSLLDGSSILTLGFPDCESSHLLLMELEKDFTPLFKLLE
Sp  LYTQDVHTTKGTLSIESFSRLSYLLEVQILLFNVQTACQARGMPFEYLPIPPKALIEDDF
Ca  KLATTAWINNGIIKLSDHELSKLTQFLIQNEEQEGDDNEEDSSTNTRLDSVLQSFKVQFY
Af  CIAAIEELESNVRILGFETVSPKGLRNDIRKVFPANVLRFSLFWHPLWERNWVVAATSSI
Sc  KTSCVVEKRIGKIVSQRGKWNLKYLDNSNVMTVKLESITYQKIMILQRTILNRIINHMLI
Nc  ANWMTRQWFVMISMSLGGDQWWLVDLSPPKPNFPAGRLRLFTKMPMTSNQLTLSDTFFRN

           790       800       810       820       830       840
Hs  EVPNKPTQLSYKYYFMSVNAADREDSPAMALLLQQFKENIQDLVFRTKTGKQTRTN----
Su  ------------------------------------------------------------
Ce  FSPDEKAVVKTHIQLLEVVGDRAQFIQLEDDEMN---------------GMHVKEAINQG
Pt  ------------------------------------------------------------
Bt  EVPNKPTQLSYKYYFLSVNAVDRD--------------------------DSPLMALLLQ
Dm  EKTTMPNEMEYTFHLGFVAYQKDELDVIDDSAMQLVSIVAQPHSDIPKCYTKLMRLIEFD
Dd  ------------------------------------------------------------
At  TQMDGSGKPQSFNDPSNILRAKKIDIGQIRILEDDLNLITSDVVKFVSSFSDAEGINQAS
Sp  TTYVQTGCLCIMMPSSNEDMLPVVFVRAHDGQLIFDSRIKGKLPYQSETETEKNCYIDWR
Ca  RRKNWPSSWFLINMISGVTTKSFWWVARIKSISGDWKIQWAQIIKFHGDNAKQELSPNES
Af  TSDNWWVVRLRRSSEVATDFSVSDTSVPLCSGHSMSDTFLATSHQTRSSSFPDLGYCLSG
Sc  DSLNQLEIRNKICSSEMINEQKLPQYIIQGSNTNDNISIITLELESFLEGSKALNSILES
Nc  LSVYAAGMISHITDLRKLHTLKKSHTALELPNHSLPPQVRLPTIYVRLSEMLQQRPGSSS

           850       860       870       880       890       900
Hs  ----------------------AKRKLSDDPCPVESKKTKRAGEMCAFNKVLAHFVAMCD
Su  ------------------------------------------------------------
Ce  TMRFSPSHKTSLQEECSR-----EQRLAFAVATVEDRITYMYLAAELMKKGIGVDVRKDS
Pt  ------------------------------------------------------------
Bt  QFKENLQESVYRTKNGKQPRTSTKRKLSDDLCPVEPKKTKRSGEMRAFNKVLAHFVAMCD
Dm  TFVATHGPGTEVDAEVSP-----HKRKSNGDLLAPPAKQQKTIFPAYFIPELAHVVAMCD
Dd  ------------------------------------------------------------
At  GHRQPGLVDEALTEMSGSQLSFSSVVDGVFGLQKVTSALMSIDGHGLVPKNLSAVTGHGK
Sp  TGRITIRVQNFSSFEKTWIGLLKLVALSKTSAFNVDCITLKHVDFTYLDDEKFRATIHDD
Ca  KVFDKPKRTVDCSIESEQLNFEFFKTLSTLSSNLILDHMILEELQVRSIKFIKLDWETID
Af  MVAIYANVSYLSDLHSVEFHPPLCALKVESDLQIPDIFIRYQVSNLPRALQLVLPAGLKR
Sc  SMFLRIDYSNSQIRLYAKFKRNTMMIQCQIDKLYIHFVQEEPLAFYLEESFTNLGIIVQY
Nc  RTLTWAKDFVPIIFKGVRSHTGDQEGPADATVRRDTPASIRAEARLTVIDRNKFKSLRGN

           910       920       930       940       950       960
Hs  TNMPFVGLRLELS--NLEIPHQGVQVEGDGFSHAIRLLKIPPCKG---------------
Su  ------------------------------------------------------------
Ce  AHVPGG-LALHITDVKNVVPFEASEFFECCIRCCLR------------------------
Pt  ------------------------------------------------------------
Bt  TNMPFVGLRLELSN--LEIPHQGVQMEGDGFSHAIRLLKIPPCKG---------------
Dm  EKIPFMNLAQTLSKHN--IPHSGLQVEANATSLVLKILALPQPGKTTFATQQQQQQGAPA
Dd  ------------------------------------------------------------
At  APMLTSYHSDSLYNRQGPLQSSSYNMLSSPPGKGSAMKKIAISNSDQELSLILSPSLSTG
Sp  NTFTLHFFNRHSPFHLISQFLQDTFSDGPSAIQPLRVIMDRTRG----------------
Ca  DNKIFLKFKQNHDISLKRKNSHNINIDVNVDVADNTSTTTNTKYIRAPKLKYESMFLIYN
Af  KNLLKDTVRLAFHGIDRHKNSAIFVAYGNLVGPWTDLCTLISKSD---------------
Sc  LTKFRQKLMQLVVLTDVVERLHKNFESENFKIIALQPNEISFKYLS--------------
Nc  VDHDVVYNHRTGQFSLKLRADMGTPVVDLLADRVQALERLMEFVEAIRLAG---------
```

Med14

```
           970       980       990      1000      1010      1020
Hs  ---------------ITEETQKALDRSLLDCTFRLQGRNN---RTWVAELVFANCPLNGT
Su  ------------------------------------------------------------
Ce  -------------------------------------LDNRNRYTFQFEMCFENIPLVRD
Pt  ------------------------------------------------------------
Bt  ---------------INEETQKALDRSLLDCTFRLQGRNN---RTWVAELVFANCP----
Dm  VAGENKPSGTSGLPKIDSHVWDDLMRRVLSISIRSQTNKNSQVRIWVVEFVFYSTPLQSS
Dd  ------------------------------------------------------------
At  NGVSESGSRLVTESSLSPLPLSQTADLATSSAGPLLRKDQKPRKRSASDLLRLIPSLQVV
Sp  ----------------------------------VLVAQELGYVVLARSLRQYRIILSKN
Ca  DKLLPIYNSATILFLKIELVESNKMYLKLFGNLRNLQIKNTSEDIQKLHLNIDEANQYFE
Af  -----------------------------------------------SSLVFKQGGSGFA
Sc  --------------------------------NNDEDDKDCTIKISTNDDSIKNLTVQLS
Nc  ----------SNAIPQSVALREVVFTYSNDPAEPVVGPPQGPRAWNVHLDLPKDAKVVLT

          1030      1040      1050      1060      1070      1080
Hs  STREQGPSR-----------HVYLTYENLLSEPVGGRKVVEMFLNDWNSIARLYECVLEF
Su  ------------------------------------------------------------
Ce  VPHGLPHRRDGEPKDRTSKDATWLQELNHINQS-SPEKLVELIIHRLMRYLYMYKVVHQF
Pt  ------------------------------------------------------------
Bt  -------LNGTSTREQGPSRHVYLTYENLLSEPVGGRKVVEMFLNDWNSIARLYECVLEF
Dm  HPKEQGSRR--------------TVYLTYEQANYDFSKTVEDLLNDWSKIVYLYTLVYDF
Dd  ------------------------------------------------------------
At  EGVASPNKRRKTSELVQSELVKSWSPASQTLSTAVSTSTKTIGCSYGNLIAEANKGNAPS
Sp  HGIQVLLNRHGCILQDLSYLSADSRYLEGTQTLTSQWEPCSWLNTVWEGDLGDDELNGQI
Ca  IDNTVDLSTVINEPKTLLLNLIFNTLNKLNSLIKILDQLNKSNVTVLDNSMDNITINIKD
Af  LRLLAPAGRPVIVQLFKSLQTLECTLSILDFLRQRRLTPQSLSLTHIAFAYGPGDLSATI
Sc  PSNPQHIIQPFLDNSKMDYHFIFSYLQFTSSLFKALKVILNERGGKFHESGSQYSTMVNI
Nc  LEKGNPHIRALDMLQDMAQSTKTELLPSYLPFSLPLYRVLDRIEDEWESIAAKNAGNVAI

          1090      1100      1110      1120      1130      1140
Hs  ARSLPDIPAHLNIFSEVRVYNYRKLILCYGTTKGSSISIQWNSIHQKFHISLGTVGPNSG
Su  ------------------------------------------------------------
Ce  S---LAYEKHFKNYCNIEAYTFHKLVVSYGDNRDMLMILAFNVKSQAPGSSEDFFFLNFG
Pt  ------------------------------------------------------------
Bt  ARSLPDIPSHLNIFSEVRVYNYRKLILCYGTTKGSSISIQWNSIHQKFHISLGTVGPNSG
Dm  AEQLLNKVSCN-IYWSVQSHGFRLTFVGG-------------------------------
Dd  ------------------------------------------------------------
At  SVFVYALLHVVRHSSLSIKHAKLTSQMEALDIQYVEEMGLRDAFSDIWFRLPFAQNDSWQ
Sp  EAAPEMHLIKMNKTADLTAILKRILAISRKK-----------------------------
Ca  KYNDNNDKLIIIKLPEQATDSIQLLMKNGSTTTTDEIDLRNNNILEFELILKFLNQYLRE
Af  EMGLSEVPSSAELDPVRRLAKTDPLLCLTL------------------------------
Sc  GLHNLNEYQIVYYNPQAGTKITICIELKTVLHNGRDKIQFHIHFADVAHITTKSPAYPMM
Nc  VVKAAEWVTVRFTLPAPTTRRLLCLDIKTMGRKGRLLWHVKRTDEKGHERPLNAPQDEFD

          1150      1160      1170      1180      1190      1200
Hs  CSN-------------------CHNTILHQLQEMFNKTPNVVQLLQVLFDTQAPLNAINK
Su  -----------------------------MAAVPHSVSLFPLEPDTGLCESQGPLHAICR
Ce  QSMPHRQFNSTEIDWHQKPRWNPHSMMSQLMRDDLKETNDLVFTMHFLCETIRPLVAIGN
Pt  ------------------------------------------------------------
Bt  -------------------CSNCHNTILHQLQEMFNKTPNVVQLLQVLFDTQAPLNAINK
Dm  -----------------MSAVNGHSMMRDQLAQHLNQQHSITQIAQILHETYNPLSSIAK
Dd  ------------------------------------------------------------
At  HICLQLGRPGSMCWDVKINDQHFRDLWELQKGSKTTPWGSGVHIANSSDVDSHIRYDPEG
Sp  ------------------------------------------------------------
Ca  SQLNNHNRQQISIIKIIQYLTEINPILQSTKAINQQLAELKRINSTNNNNNNAKRRSILK
Af  ------------------------------------------------------------
Sc  HQVRNQVFMLDTKRLGTPESVKPANASHAIRLGNGVACDPSEIEPILMEIHNILKVDSNS
Nc  TLLQRSVWHAHGDGRKTFGTSAAADPKVGIETLLWSISESVKSLVGTLPPPPGTSPHPGG

          1210      1220      1230      1240      1250      1260
Hs  LP-----------TVPMLGLTQRTNTAYQCFSILP-------QSSTHIRLAFRNMYCIDI
Su  LPLIPS-----LGLIAATNQVAGPI--HKFALCP--------QSSTHYRVVFHNAYCLDI
Ce  FSRIRFQSQKSLSQLIGPDVHFPFRLKYHLYALDQ-----------TTLRLMQGNVILEI
Pt  ----------MLGLTQRTNT------AYQCFSILP-------QSSTHIRLAFRNMYCIDI
Bt  LPTVP-----MLGLTQRTNT------AYQCFSILP-------QSSTHIRLAFRNMYCIDI
Dm  LPVLPFLG-------------------IPRPQVPVLSFCVLAQSPCLIRLTYQAVYCLEL
Dd  ------------------------------------------------------------
At  VVLSYQSVEADSIKKLVADIQRLSNARMFSLGMRKLLGIKPDEKTEECSANSTMKGSTGG
Sp  ------------------------------------------------------------
Ca  LSNGLYKLYFNLNIISLTHLQLVFFMNSNTGSNLKKIQRDKIMINLSLIKFDRFSKPNGN
Af  ------------------------------------------------------------
Sc  SSS---------------------------------------------------------
Nc  GTQDLQQGPQKTPQEMVAAQAAQAAQAAQAAQGMPPQRPKQQPPTPSQPQQQHRNVNQPQ

          1270      1280      1290      1300      1310      1320
Hs  YCRSRGVVAIRD---------GAYSLFDNSKLVEGFYPAPGLKTFLNMFVDSNQDARRRS
Su  HCKGKNTVAVRD---------GAFSLFDMRKVVEGLYTVPNLKAFLELFIDETVSYHHQH
Ce  KLLEGCKIAVRDVSRYRPRCAGLFQLFSNIDSETTAIMNDEIAIPQSDNPQTAGPTMWTP
Pt  YCRSRGVVAIRD---------GAYSLFDNSKLVEGFYPAPGLKTFLNMFVDSNQDARRRS
Bt  YCRSRGVVAIRD---------GAYSLFDNSKLVEGFYPAPGLKVMDFSVAELLNINGIPK
Dm  RFRANRLVSIRDG----------ASSRFERNVVEEFTPIQGLKAFLSKYVDESAVYRGRA
Dd  ------------------------------------------------------------
At  KGSGEPVDRWRAFKIEAVGLTSLWFSFGSGVLARFVVEWESGKDGCTMHVSPDQLWPHTK
Sp  ------------------------------------------------------------
Ca  FQDSTNGHLIKISFKDSLINENLKFKNLFEIIFKNINELLITKSKQIKSLNQTENQQQQQ
Af  ------------------------------------------------------------
Sc  ------------------------------------------------------------
Nc  AQAQPQLHTQVQLQQAQVAQLQAQRQAQARANNSSNNNNTFVNRAHPGQPPQQRQSGPQT

          1330      1340      1350      1360      1370      1380
Hs  VNEDDNPPSPIGGDMMDSLISQLQPPPQQQPFPKQPGTSGAYPLTSPPTSYHSTVNQSPS
Su  RNSRTLPANEDDDPYLPTQVSGYPSGASPMSRAGLGQGGSGGFAYPMTPPTAPGGPGSSN
Ce  EQFMDSLDERPEEIDPRMAITSQPILMSHDTIIKACDFKDTEGRITCPLDEYLCSISYLQ
Pt  VNEDDNPPSPIGGDMMDSLISQLQPPPQQQPFPKQPGTSGAYPLTSPPTSYHSTVNQFPS
Bt  TIPLRGK-----------------------------------------------------
Dm  SHEDDNPLSPMGMEDNFGGPSSVAGVSAGGSSPFLGTGMRGPQSPRDSGLRFPAPHTPPS
Dd  ------------------------------------------------------------
At  FLEDFINGAEVESLLDCIRLTAGPLHALAAATRPARASTATGMPVVPATASSRQSNQIQQ
Sp  ------------------------------------------------------------
Ca  PLKQEDNSDSAIIKKESQSIEDDLLDFGEYDNDIKPQQQEPQQNEKENSKTTSKENETVR
Af  ------------------------------------------------------------
Sc  ------------------------------------------------------------
Nc  QAQQQQQRYQIQQQAQAMQRMQQQQQQQAAQQHAQQQGQGPQGQRSVHGQAGPQGVPQGQ

          1390      1400      1410      1420      1430      1440
Hs  MMHTQSPGNLHAASSPSGALRAPSPASFVPTPPPSSHGISIGPGASFASPHGTLDPSSPY
Su  PATPASPHTSVLSQQQHYAMSPGAYPLASPPSIPGPSPSAVMKGTPSPGLVEGGSPFTSS
Ce  RALLTLERMSPRATLNKNSSSNLSCGFVTIIDAKPDFIRFRASQMNGDGVNATSMVHYKI
Pt  MMHTQSPGNLHAASSPSGALRAPSPASFVPTPPPSSHGISIGPGASFASPHGTLDPSSPY
Bt  ------------------------------------------------------------
Dm  SSNPHTPASPHPSAGAGGGSGPQGHGNFNLTSPPAPHMPHPSPSGLMPSSPLNPQPSPHM
Dd  ------------------------------------------------------------
At  TQGIIAPSTLAAPNATGQSASATSGNTVASSAPSPLGGGFHGVAMLAAAGRSGPGIVPSS
Sp  ------------------------------------------------------------
Ca  PDDILIKLNYDYLLSMNNLQLMINEITKSCFQYLQQQQE---------------------
Af  ------------------------------------------------------------
Sc  ------------------------------------------------------------
Nc  PGHGGGANGGMGGKNAPVVVID--------------------------------------
```

Med14

```
              1450      1460      1470      1480      1490      1500
Hs  TMVSPSGRAGNWPGSPQVSGPSPAARMPGMSPANPSLHSPVPDASHSPRAGTSSQTMPTN
Su  MGLTMPSPGSRQWPGSPSMPGPSPVQRFGMAQSPGGSMGPSTHSPGSSGMTGQQGQVGE-
Ce  YLCPVAMTLKIRIEFGMELYWKKKNIFQMRFFALKLLYLVSKRLSLVRIK----------
Pt  TMVSPSGRAGNWPGSPQVSGPSPAARMPGMSPANPSLHSPVPDASHSPRAGTSSQTMPTN
Bt  ------------------------------------------------------------
Dm  VHSPGPNTLYMQSHQDSPFTAMSPANNNWPGSPSMPRPSPRPGQSPDHKSTGGGAGVAGG
Dd  ------------------------------------------------------------
At  LLPIDVSVVLRGPYWIRIIYRKRFAVDMRCFAGDQVWLQPATPPKGGASIGGSLPCPQFR
Sp  ------------------------------------------------------------
Ca  ------------------------------------------------------------
Af  ------------------------------------------------------------
Sc  ------------------------------------------------------------
Nc  ------------------------------------------------------------

              1510      1520      1530      1540      1550      1560
Hs  MPPPRKLPQRSWAASIPTILTHSALNILLLPSPTPGLVPGLAGSYLCSPLERFLGSVIMR
Su  ------------------------------------------------------------
Ce  ------------------------------------------------------------
Pt  MPPPRKLPQRSWAASIPTILTHSALNILLLPSPTPGLVPGLAGSYLCSPLERFLGSVIMR
Bt  ------------------------------------------------------------
Dm  TDRGGSRGTLNRPWAGAVPTLLTHEALETLCRPSPYPNKDINVPDMSPLERFLGCVYMRR
Dd  ------------------------------------------------------------
At  PFIMEHVAQELNGLEPNLTGSQGATNPNSGNPTVNGVNRVNFSPSSARAAMNRVASVASG
Sp  ------------------------------------------------------------
Ca  ------------------------------------------------------------
Af  ------------------------------------------------------------
Sc  ------------------------------------------------------------
Nc  ------------------------------------------------------------

              1570      1580      1590      1600      1610      1620
Hs  RHLQRIIQQETLQLINSNEPGVIMFKTDALKCRVALSPKTNQTLQLKLTPENAGQWKPDE
Su  ------------------------------------------------------------
Ce  ------------------------------------------------------------
Pt  RHLQRIIQQETVWVPSDLLSGDC-------------------------------------
Bt  ------------------------------------------------------------
Dm  QLHRNIQSEETLTALNSTEPGVVLFKVDGLQCQVVLNQMHMQTLHLKVSQLPPMPDKHPP
Dd  ------------------------------------------------------------
At  SLVVSSGLPVRRTPGTAVPAHVRGELNTAIIGLGDDGGYGGGWVPLVALKKVLRGILKYL
Sp  ------------------------------------------------------------
Ca  ------------------------------------------------------------
Af  ------------------------------------------------------------
Sc  ------------------------------------------------------------
Nc  ------------------------------------------------------------

              1630      1640      1650      1660      1670      1680
Hs  LQVLEKFFETRVAGPPFKANTLIAFTKLLGAPTHILRDCVHIMKLELFPDQATQLKWNVQ
Su  ------------------------------------------------------------
Ce  ------------------------------------------------------------
Pt  ------------------------------------------------------------
Bt  ------------------------------------------------------------
Dm  PFQLSQDDLLVIEQYFDTRVAAPPYRPNSLHSICRLLNLPAQVLKDFVQIMRLDLKPELG
Dd  ------------------------------------------------------------
At  GVLWLFAQLPDLLREILGSILKDNEGALLNLDQEQPALRFFVGGYVFAVSVHRVQLLLQV
Sp  ------------------------------------------------------------
Ca  ------------------------------------------------------------
Af  ------------------------------------------------------------
Sc  ------------------------------------------------------------
Nc  ------------------------------------------------------------

              1690      1700      1710      1720      1730      1740
Hs  FCLTIPPSAPPIAPPGTPAVVLKSKMLFFLQLTQKTSVPPQEPVSIIVPIIYDMASGTTQ
Su  ------------------------------------------------------------
Ce  ------------------------------------------------------------
Pt  ------------------------------------------------------------
Bt  ------------------------------------------------------------
Dm  GDQLKWTVQICLRMPPSAVPIVPSGNACVVMGRMKILFFLQITRIPYGAVIGVGKDWKDS
Dd  ------------------------------------------------------------
At  LSVRRFHHQAQQNGSSAAAQEELTQSEIGEICDYFSRRVASEPYDASRVASFITLLTLPI
Sp  ------------------------------------------------------------
Ca  ------------------------------------------------------------
Af  ------------------------------------------------------------
Sc  ------------------------------------------------------------
Nc  ------------------------------------------------------------

              1750      1760      1770      1780      1790      1800
Hs  QADIPRQQNSSVAAPMMVSNILKRFAEMNPPRQECTIFAAVRDLMANLTLPPVGRP----
Su  ------------------------------------------------------------
Ce  ------------------------------------------------------------
Pt  ------------------------------------------------------------
Bt  ------------------------------------------------------------
Dm  PSLVLPIVYDIQTNVTQLAERTGQVISPTMTAASTLLRRFAEFNAQQNQCTLFPAITDLL
Dd  ------------------------------------------------------------
At  SVLREFLKLIAWKKGLSQSQQAGEIAPAQRPRIELCLENHSGTDLDNNCAAKSNIHYDRP
Sp  ------------------------------------------------------------
Ca  ------------------------------------------------------------
Af  ------------------------------------------------------------
Sc  ------------------------------------------------------------
Nc  ------------------------------------------------------------

              1810      1820      1830      1840      1850      1860
Hs  ------------------------------------------------------------
Su  ------------------------------------------------------------
Ce  ------------------------------------------------------------
Pt  ------------------------------------------------------------
Bt  ------------------------------------------------------------
Dm  TNLQLAAEMPQPPPNQSIGPPVGVGVGVGSSPNPMMPMQQLPQQVGPQGPVGPGGYPQMG
Dd  ------------------------------------------------------------
At  HNTVDFALTVVLDPVHIPHINAAGGAAWLPYCVSVRLRYTFGENPSVTFLGMEGSHGGRA
Sp  ------------------------------------------------------------
Ca  ------------------------------------------------------------
Af  ------------------------------------------------------------
Sc  ------------------------------------------------------------
Nc  ------------------------------------------------------------

              1870      1880      1890      1900      1910      1920
Hs  ------------------------------------------------------------
Su  ------------------------------------------------------------
Ce  ------------------------------------------------------------
Pt  ------------------------------------------------------------
Bt  ------------------------------------------------------------
Dm  PNPGGPQ-----------------------------------------------------
Dd  ------------------------------------------------------------
At  CWQRVDDWEKCKQRVSRTVEVNGSAAGDLTQGKLKLVADSVQRTLHLCLQGLREGGNNNN
Sp  ------------------------------------------------------------
Ca  ------------------------------------------------------------
Af  ------------------------------------------------------------
Sc  ------------------------------------------------------------
Nc  ------------------------------------------------------------
```

Med14

Hs	-	-	-	-	-	-	-	-	-
Su	-	-	-	-	-	-	-	-	-
Ce	-	-	-	-	-	-	-	-	-
Pt	-	-	-	-	-	-	-	-	-
Bt	-	-	-	-	-	-	-	-	-
Dm	-	-	-	-	-	-	-	-	-
Dd	-	-	-	-	-	-	-	-	-
At	N	T	H	Q	K	E	F	T	I
Sp	-	-	-	-	-	-	-	-	-
Ca	-	-	-	-	-	-	-	-	-
Af	-	-	-	-	-	-	-	-	-
Sc	-	-	-	-	-	-	-	-	-
Nc	-	-	-	-	-	-	-	-	-

Med15

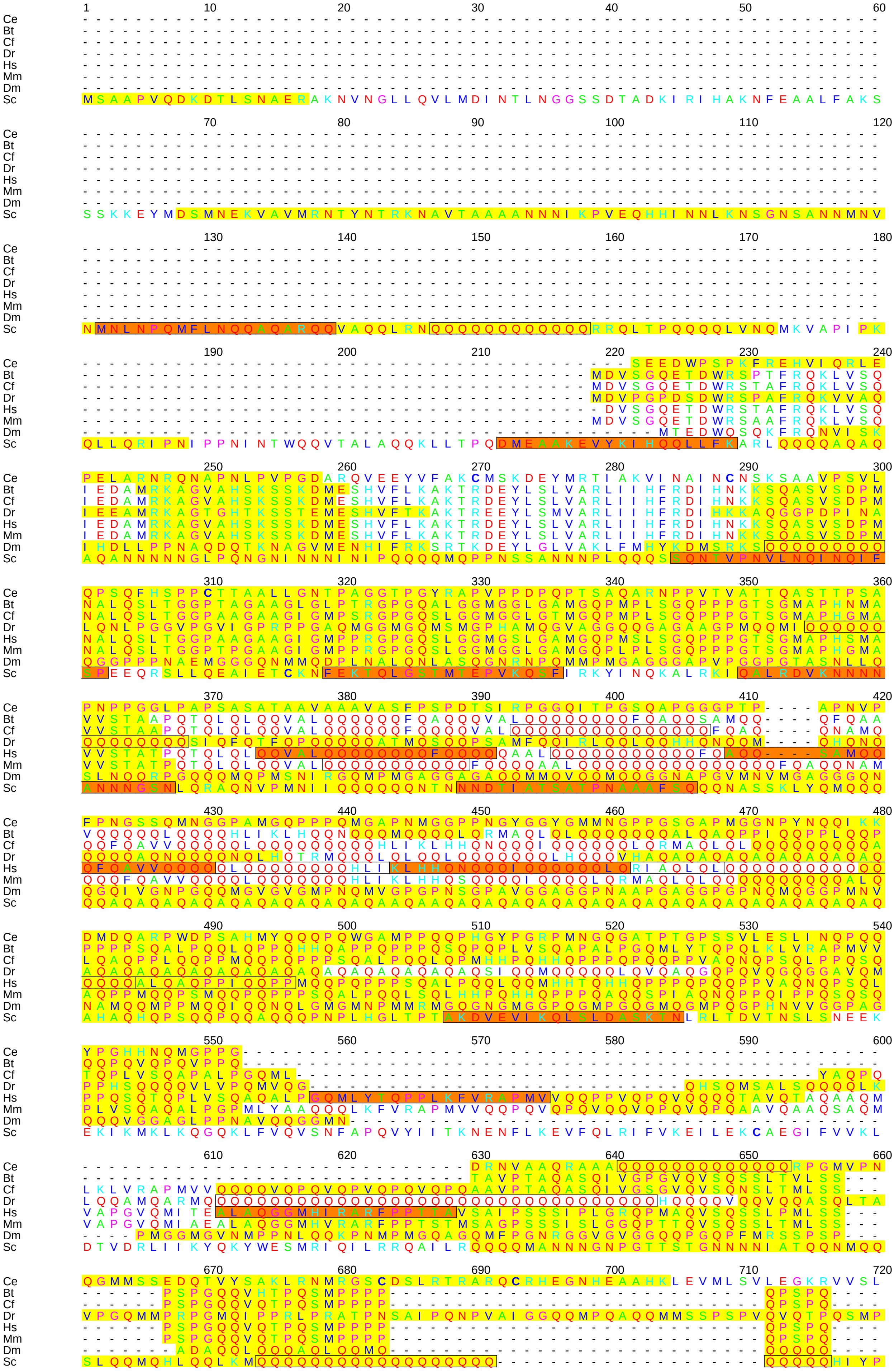

Med15

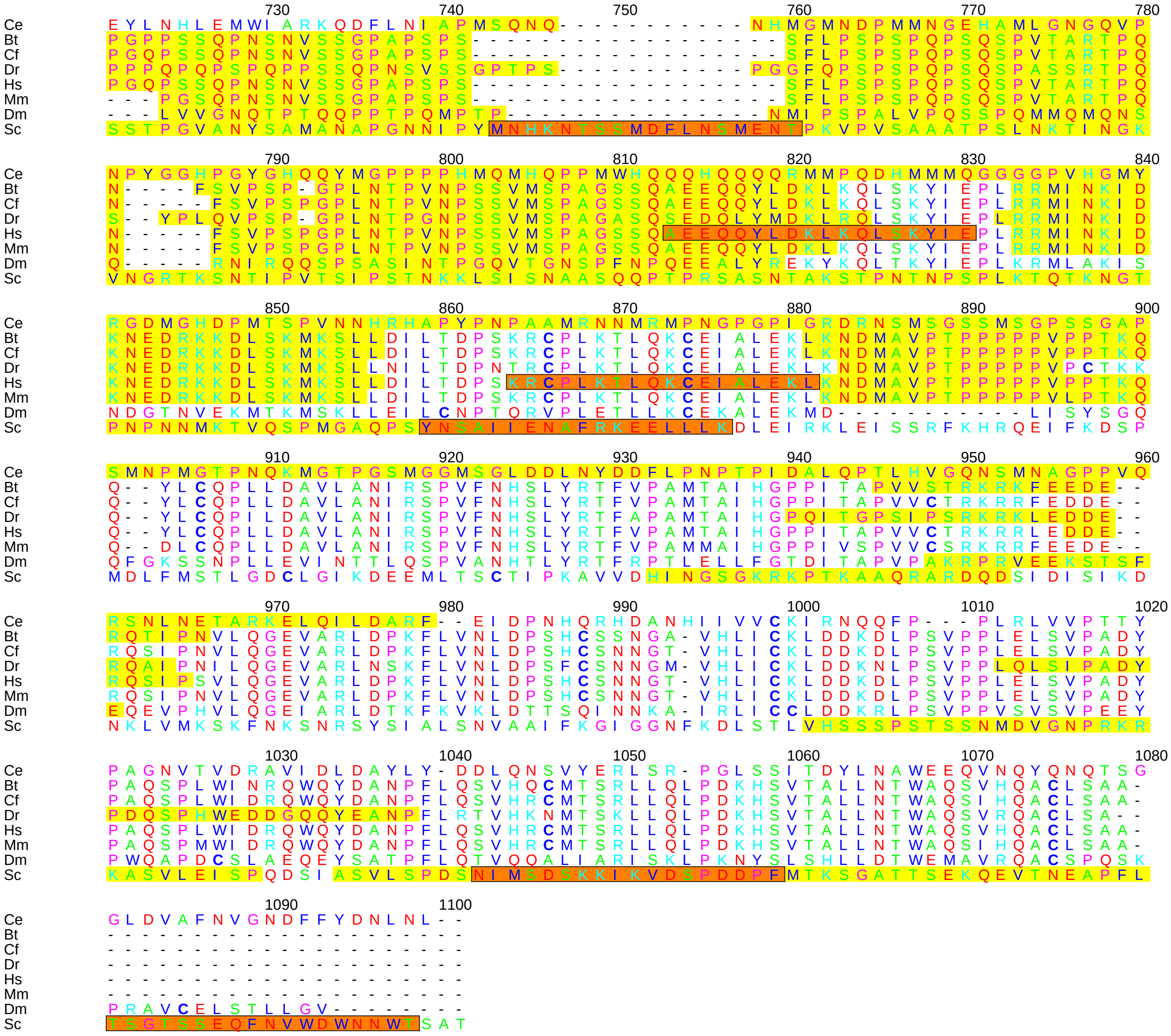

Med16

```
    1        10        20        30        40        50        60
Ca  --------------------------------------------------------------
Sc  --------------------------------------------------------------
Af  --------------------------------------------------------------
Dm  --------------------------------------------------------------
Nc  --------------------------------------------------------------
Gg  MEVHGTSLLSHPCRRIEDCLPPLESSPSKRFSPSKRKQYYINKAIRNSDLIPKAKGRKSLQR
Mm  --------------------------------------------------------------
Hs  --------------------------------------------------------------

           70        80        90        100       110       120
Ca  --------------------------------------------------------------
Sc  --------------------------------------------------------------
Af  --------------------------------------------------------------
Dm  --------------------------------------------------------------
Nc  --------------------------------------------------------------
Gg  LENTRYLMSLLERDECGSNEAELAHSATPSIFTEACSNETYVEIWNDFMNRSGEEQERVLLY
Mm  --------------------------------------------------------------
Hs  --------------------------------------------------------------

         130       140       150       160       170       180
Ca  --------------------------------------------------------------
Sc  --------------------------------------------------------------
Af  --------------------------------------------------------------
Dm  --------------------------------------------------------------
Nc  --------------------------------------------------------------
Gg  LEEEARKKQKRKLSRPLKEKEKWKVVAGPGYESARRDERPPPKAHHEPEWEFVWASCGCGGC
Mm  --------------------------------------------------------------
Hs  --------------------------------------------------------------

       190       200       210       220       230       240
Ca  --------------------------------------------------------------
Sc  --------------------------------------------------------------
Af  --------------------------------------------------------------
Dm  --------------------------------------------------------------
Nc  --------------------------------------------------------------
Gg  LGFERLLLHALCQYMDLVSASSDIEGKRQMKVSNKHHVFLPPELLLSDYLEHPCAFNDAKPV
Mm  --------------------------------------------------------------
Hs  --------------------------------------------------------------

     250       260       270       280       290       300       310
Ca  ------------------------MATQTDKQTIGRHLEQVPKASNLISWSRNGFIAYIPPI
Sc  ------------------------------------MMLGEHLMSWSKTGIIAYSDSQSSNA
Af  --------------------------------------------------------------
Dm  --------------------------------------------------------------
Nc  ----------------------------------MSGHDIPIQLLDGGSIDVQHHDQMNAMD
Gg  ACCWAGCFWAQPAEEPRDCGTAPCVAATEVSEHSPEAVLELAKPFFARFRARGLLGSSLCKF
Mm  --------------------------------------------------------------
Hs  --------------------------------------------------------------

             320       330       340       350       360       370
Ca  ITTTTTTPTTTTTTNNNKSNLLLTYIKNSDGKKWQLASPEPINIKLENNFLPQLSLVSWGSL
Sc  NICLTFLESINGINWRFHTPQKYVLHPQLHEVQYQESSSTLSTHSTTTSVNGSTTAGVGSTP
Af  --MPLIMEDGINVDDLFGEPGSLELGLSPSTTSPRGLAQRLDEMRLIGCCQKIAWSKLGCIA
Dm  --------------MTILYKVEGKEFSKDSVFPHKKVLCSVSARNIVAFSALQSAIFPASHG
Nc  TAMNLDDVDVDLFGDSVMVDTALDGLSAPRPMPSKHLRQRLDDLRTHGCHQAIAWSRQGTIA
Gg  GAGAQDLRLGCRRQCLKERPRGCALYARSLRTQSAVELFRSYSIWNDFMNRSGEEQERVLLY
Mm  ---------------------------------------MDLAYVCEWEKWAKSTYCPSLPL
Hs  ---------------------------MCDLRRPAAGGMMDLAYVCEWEKWSKSTHCPSVPL

           380       390       400       410       420       430
Ca  NTDLAVSDIYGNFYILLAGVGLLDPNHIPSTITTTSTIKTEGNTEKNKDTKQIGNGSGTNGH
Sc  NFGGNSNKSPPQFFYNISSIHWNNWFSLPGDMLAVCDELGNMTMLITGQRPDRATTYEKLTM
Af  YISQDGLRVNVRHLQCRPSDGKWVLSEET----PLLPVTDAHGGHTLVHLC--WNE--PGVE
Dm  GDAGDGSLPGAGLAVGASNSHVYVCDIVT----PWEYYKVCSSKSLISVLQ--WSP--NGEQ
Nc  SITKDGRSVEFRFLRCRPEDAGWELSEPYLYGPAIIPMHTTAG--PIVHLA--WSTTTPTPE
Gg  LEEEARKKQKRKLSRPLKEKEKWKVVAGPGYESARRDERPPPKAHHEPEWEFVWASGGCGGC
Mm  ACAWSCRNLTAFTTDLRNDDQDLTHMIHILDTEHPWEVHSVSSGHSEAITCLEWDQ--SGSR
Hs  ACAWSCRNLITFTMDLRSDDQDLTRMIHILDTEHPWDLHSIPSEHHEAITCLEWDQ--SGSR

         440       450       460       470       480       490
Ca  GDSPINTPSFELTSYNHMEMIYRDIINPDINSVVNPGASIVAFKWLN-----IEKPQ-----
Sc  VFQDNVYKIYNHVMPLKPVDKLKPMNIERKQTRKEYNTSILEFRWLT-----SSKSV-----
Af  LAVADSSGRVSIYSISIALNSIAGHRQAAFDPDDD-GAQIVGMMWLN-----TQRTV-----
Dm  LLLGYVGGRVEIWQPRNQSINLWVLQYYATVPSED---IIEAQFFQN-----GKGVIFNALK
Nc  LAVVDAVGRITILSLTIHLNRPFITRKWDADPVDD-LHAVVGCYWLPLLN--PRQLS-----
Gg  LGSLCKFGAGAQDLRLGCRRQCLKERRRGGSSPRGPGTAAHARWSSPGSVPLPPPFPGAAMD
Mm  LLSADADGQIKCWSMADHLANSWESSVG----SQVEGDPIVALSWLHNGVKLALHVE-----
Hs  LLSADADGQIKCWSMADHLANSWESSVGSLVEGDP----IVALSWLHNGVKLALHVE-----

       500       510       520       530       540       550
Ca  --IVNKAATRLAEN-----------PTTTSSNSSSSIYGYGIN----QYQPY-GVCHPIPT-
Sc  --IVSQFCAFDSSS---------------------NTYRSRA----QQVPPY-GVYHP-PFI
Af  --HSFYQAAKVQGR-----------------------WAYSP----FRRRPI-GPFHP--VN
Dm  KDHTYYAEKFERVE---------------------------------QQRPTLSGFGG--VA
Nc  --VMYGPAVWVQPEDAKTPGDG--------------LYKYES----SYTKPT-GPNHPNTN-
Gg  LAYVCEWEKRPKSDHCPPIPLVCAWSCRN-------LIAFTTDLRNEEERDLTHMVHIIDTE
Mm  ---KSGASSFGEKFS---------------------RVKFSPSLTLFGGKPM----------
Hs  ---KSGASSFGEKFS---------------------RVKFSPSLTLFGGKPM----------

     560       570       580       590       600       610       620
Ca  KQACVALRKNGQFILLFY------------------QGEHKVEYHKI-----------CCNL
Sc  KYACLAIRKNGQIDFW----YQFS--------NSKDHKKITLQLL---------------DT
Af  KAGLVCVTRSGIIRLLYQ-------------NPDGRWAEISAELK---------------NT
Dm  SEGCVLLPSSGLLAAFSLPAIQKTSAAG-ADGPAHEVVELTPALH---------------SI
Nc  KSALVSITTNGLLRLMFQ-------------NNNTRLEETAIELES--------------IT
Gg  HPWDVYSVNSGHVEAITCLEWDQSGSRLLSADADGHIKCWSMTDHLANSWENTMGSMVEGDP
Mm  -EGWIAVTVSGLVTVSLLK-----------------------------------------PS
Hs  -EGWIAVTVSGLVTVSLLK-----------------------------------------PS

             630       640       650       660       670       680
Ca  TDNIS----IIEKASIGFN-NDK----QIIVTAWDSLSN-----DINVYSIDINWGFLVESA
Sc  SNQRFKDLQWLEFARITPMNDDQ-----CMLITTY--SKLSKN--ISFYKLHVNWNLNATKP
Af  GYSDR----LLTHAALVST-------QGGILIATH---SACQK--LCLYRVHIAWTPTQYDP
Dm  GISRS----FIEHCSMTPS------PSGALNIAFS--CG-WQQQLVQCFKVSLSLEGELG--
Nc  SSDD-----LITHAALCND-------KNALLIALATASK-----QLRIVKATISWGNDK---
Gg  VVALSWLHNGVKLALHVEKASDADIFALPNLHSQSGASNFGEKFSRVKFSPSLTLFGGKPME
Mm  GQVLTSTESLCRLRGRVALADIAFTGGGNIVVAAADGSS---ASPVKFYKVCVSVVSEKCR-
Hs  GQVLTSTESLCRLRGRVALADIAFTGGGNIVVATADGSS---ASPVQFYKVCVSVVSEKCR-

           690       700       710       720       730       740
Ca  KRQKLDQHYHTPKEAQKPP---RLTLKKLHQMKPIQCFKEEDGESQSQLVVPEMGKLKVEEL
Sc  NVLN---------------------DPSLKIQFILSTTLDPTDDEGHVLKLENL---HVVSK
Af  GQQK-------PPVPWPVPSFRFLHCKV-ESQCDVRGTNRHAGDNAPGLPSFTN---SLYCL
Dm  --------------------------------LEQHLAIKSESQTSIFLGPLDG-----RRI
Nc  -----------PAEKQAHPQNAAITASISEQHVAVAPLLQHDSND----AVLDD---SMSQL
Gg  GWIAVTISGLVTVSLLKPNGQVLTSTESLCRLRCRVALADVAFTGGGNIVVATSDGSSASPV
Mm  ----------------------------IDTEILPSLFMRCTTDP-----NRKD---RFPAI
Hs  ----------------------------IDTEILPSLFMRCTTDLN-----RKD---KFPAI
```

Med16

```
     750 760 770 780 790 800
Ca   SSID----------IISSNP--------DPKSGLSILITYGSSIIY--RYALEKS------D
Sc   SSIE-----------KDPSP-EILVLYNVCDTSKSLVKRYRLAPTQ----LSAEYLVILKPD
Af   TGLD----------IVLPALDN-----PAGSTANPWVVAIYSAPLH--VTQDHPQ--QQGPA
Dm   SHLKWTRIANEDVIFIAYACPDG-----AGSQLEQWTLTRRHQSVHALLQGGGGAN--NKPN
Nc   SHIE----------FLPSIPKD----KNLQAHHNPVVLTVRS-----HLPAEGASLYEQEPT
Gg   QFYKVCVSVVNEKCKIDTEILPSLFMRCTTDPARKDKYPAITHLKFLARDMSEQVLLCASNQ
Mm   THLK----------------------FLARDMSEQVLLCASSQTSS----------------
Hs   THLK----------------------FLARDMSEQVLLCASSQTSS----------------

     810 820 830 840 850 860
Ca   SSNTTSGGET-SKNPIS--QAFYNLGVEKKIDWKGD--------DSSVRLVFKDKLMRRGQI
Sc   LNID--------------------RNNSTNQ--IFQ--------SRRYNLRRHSDIVLDKKV
Af   SVF--VRWQL-DTGPLTLHPKFDDVPSKKNN----------AQVKPKLELRRLDDVYSDKYA
Dm   EFVQSESWEQ--VGKVQLNASLADISVTRLS-------------------------------
Nc   SVID--RWEVVSEQPQTLHPAFAQLNTGNGMT----------NPNSMTRLRKLDAVVIPKIV
Gg   NNSIVECWSL-RKEGLPVNNIFQQISPVVGD--KQPMILKWRILSATNDLDRVSAVALPKLP
Mm   ---LVECWSL-RKEGLPVNNIFQQISPVVGD--KQPMILKWRILSATNDLDRVSAVALPKLP
Hs   ---IVECWSL-RKEGLPVNNIFQQISPVVGD--KQPTILKWRILSATNDLDRVSAVALPKLP

     870 880 890 900 910 920 930
Ca   ESIIN---GFL--------DLSFLIIYKDGTVDVVDTTSWQIVNNSKNNNSVTGDEMEL---
Sc   TLITS----------EMF-DAFVSFYFEDGTIESYNQND-------------WKLETERLIS
Af   ISIDQ----------IEY-GNVLAITYDDGSVVFYDPK--------------------TMAV
Dm   --VST----------PDS--CQVYAILQDNSVQVLEPNS-----------------------
Nc   VSVQVMY-----------LGKVVCFAFSDGTIQYRDRVT--------------------MME
Gg   ISLTNTDLKVANDTKFFP-GLGLALAFHDGSVHIVHRLS-----------------------
Mm   ISLTNTDLKVASDTQFYP-GLGLALAFQDGSVHMVHRLS-----------------------
Hs   ISLTNTDLKVASDTQFYP-GLGLALAFHDGSVHIVHRLS-----------------------

     940 950 960 970 980 990
Ca   -DNNDYPPKLISNLFDQGFQF--PKISHKNRLV---LAISPTMSSIVYT--EIYGETVNL-Q
Sc   QSQLGKFKNIIASPLSAGFNYGKLP-LPPSVEW---MKVSPSMCG--VIVKQYNKKWPQFYA
Af   VNGVDDANTVTS-LAQAGFHH---PPEPSAGLH---ISFSPNACA----AVMLDGEGQTHLR
Dm   ---------------MKQLNHTQFDRLSDASGAVKFVSGDLTPSS----QILLIFDSHAQLY
Nc   LFNEQNVHRVLS-PHDVGFQY----VNDTPCLQ---VAFSPTNCS----YAQISEDWDIKWN
Gg   -LQMMAVFHGSSSQRPVDEQTIKRQRTTGPLVHFKAMQLSWTSLA----LAGIDSHGKLS--
Mm   --LQTLAVFYSSAPRSLDEPALKRLRTTCPAVHFKAMQLSWTSLA----LVGIDNHGKLS--
Hs   -LQTMAVFYSSAAPKPVDEPAMKRPRTAGPAVHLKAMQLSWTSLA----LVGIDSHGKLS--

     1000 1010 1020 1030 1040 1050
Ca   LKPLERIDNFGTNSHDLYYTS--VAFAHRYAF--ALYTSTCSDDLLLLIQS-----------
Sc   AVQKNY------ADPEKDSIN---ATALAFGYVKSLHKQISAEDLTI-------AAKTHILR
Af   LTEHSYGAEGGLHDETLDKYS-AAIAALTLAFCRGCGSDVNTDDILL---------------
Dm   AMQAPLPKQGGSG-------------LLLLEYCIVTGCDASDVLLLNLGN------------
Nc   SMRYTLT-DASTALQSVAQQS-AVMAALTLALSSAVTTSINFDDILA---------------
Gg   -----MLRISPSMGHVLDMNTSLRHLLFLLEYCMVTGYDWWDILLHVQPNMVQN--------
Mm   -----MLRISPSLGHPLEPKLALQHLLFLLEYCMVTGYDWWDILLHVQPGMVQS--------
Hs   -----VLRLSPSMGHPLEVGLALRHLLFLLEYCMVTGYDWWDILLHVQPSMVQS--------

     1060 1070 1080 1090 1100 1110
Ca   ----------------EINRIKDAVDGDTITRNKLGKQLC---DLVIIE--CHKAINF----
Sc   ISFLDRKRAK----EFITTLLKSLY------------------SFFNIS-------------
Af   --ILVRQLTP----EAQATFINEAY------------------RALPIN--CN--------F
Dm   LEALVEKLTDNFTR--QPSFVRHFYYA----------------NFLALKS------NICRMQ
Nc   ---LARPYSDNPQF--AASWVKDLVN------------------LLKLN----------FDF
Gg   ---LVEKLHEEYTR--QSAALQQVLAT----------------RIVAMKAS---------LC
Mm   ---LVERLHEEYTR--QKPALQQVLST----------------RILAMKAS---------LC
Hs   ---LVEKLHEEYTR--QTAALQQVLST----------------RILAMKAS---------LC

     1120 1130 1140 1150 1160 1170
Ca   HLDTITKES--LDKLLSNNASLQ---KLLSLQLILGEFQQLSHFPNNYV----VPDIAWIVL
Sc   PDAP--------KEIMDKIITSRPLQKIMLLQLELG---------SCFSQEN-IEEMARVIL
Af   TVE---------QDKLMNHPYIP---RCLSIQAALG-------FKNKYTPRSFASSIPWAVL
Dm   QQE---------FDNLIILHAISTTFKSLLRPSDLG-------FQDKGPADNLAIKLAESIP
Nc   SDEG-------HYDSLVRSPLFQ---ISLSILNHMG-------FRGEFRPRPFEGKLSFLIL
Gg   KLSSSTIARVCDYHAKLFLIAISCTLKSLLRPHFLN-------TPDKSPGDRLTEVCSKITD
Mm   KLSPCTVARVCDYHTKLFLMAITSTLKSLLRPHFLN-------TPDKSPGDRLAEICAKITD
Hs   KLSPCTVTRVCDYHTKLFLIAISSTLKSLLRPHFLN-------TPDKSPGDRLTEICTKITD

     1180 1190 1200 1210 1220 1230 1240
Ca   NLRSASLGIMFTLSSIY-------------------RQVSKKKPSEDT----LQDSITRGEC
Sc   YLKN-------VLFAFNGVARNFHFAIEQISNNSNQQQNPKLFQTIFS----KQD------L
Af   QLRHASV-----LYAFF-------------------FQYNKGGTTEPHDP----------DV
Dm   DVDT---------VMLN--------------------LDAKDFTVEPV-------------M
Nc   QMRNFIV-----LVSLS-------------------LNAPKSLGISPLDEA---------EV
Gg   IDIDKVMIN--------------------------------LKTEEFVLEMN---------T
Mm   VDIDKVMIN--------------------------------LKTEEFVLDMN---------T
Hs   VDIDKVMIN--------------------------------LKTEEFVLDMN---------T

     1250 1260 1270 1280 1290 1300
Ca   IMSIIGNFKWLIDLLVYLNQELLQLIYVKNNFL-NNGNTTTSKLTLSNSIVLP---------
Sc   IHSLIPVAKWFVKFITYLTQEI----------------LILIN---DPTNKEYTLVHGIFGA
Af   LRMVLGNTKWALDFSFYVLNEL----------------FDLAD-------------------
Dm   LQSLQQLIQWVTDLALNMLHRLPEEVMKTKLSG-KRPSYDISR-------------------
Nc   VEVLVGCVTWAHTLIGWMVDCLFDLADNPAFIS-ILKEQKRFPDLANFLRARGDVSLHLLCC
Gg   LQSLQQLIQWVGDFVLYLLASLPNQGSPVRPG------HSFLR-------------------
Mm   LQALQQLLQWVGDFVLYLLVSLPNQGSPLRPG------HSFLR-------------------
Hs   LQALQQLLQWVGDFVLYLLASLPNQGSLLRPG------HSFLR-------------------

     1310 1320 1330 1340 1350 1360
Ca   ----------------------------------------------LILNKVSRLFLMYAIS
Sc   KMSR------------------------------------------TLILSILNEIKKVTQI
Af   -----------------------------------------------DFESLSGDQEAFTQK
Dm   -----------------------------------------------DIVAISSLRELLVMI
Nc   SSARGFIMAACRRLAHLEGMSNRAIRYWEASRLKTENNDGSGGGGKVLPDSLYFAYRKMQRA
Gg   -----------------------------------------------DGASLGMFRELMVVI
Mm   -----------------------------------------------DGTSLGMLRELMVVI
Hs   -----------------------------------------------DGTSLGMLRELMVVI

     1370 1380 1390 1400 1410 1420
Ca   AMGRTHEILKKLHKDLTEANKLFAPMKESLNRYFSISNNSPITVNLFESYLRECDALLNKEV
Sc   -----------VAKFPETSYPILNESSTFLKLVLSESPVDFEKFETFLVDVNNKFI-----A
Af   CSWGLASTYLHVSRIPAVHLPGFARD---LRRLRDRSASKRRCPRLLRGDLPNTGIRPNPYR
Dm   RIWGLLNTQC---------LPVYTKT---MDNIDVLVILFRLLTRLAQN-------------
Nc   TSSSLIKVQELNEVLQGFAQDVRTAYQASLSRRQSGNNQPQQQHPSPQQNKNQPQQQQNAED
Gg   RIWGLLKPSCLPVYTATSDTQDSMSLLFRLLTKLWLCCREES--------------------
Mm   RIWGLLKPSCLPVYTATSDTQDSMSLLFRLLTKLWICCRDEG--------------------
Hs   RIWGLLKPSCLPVYTATSDTQDSMSLLFRLLTKLWICCRDEG--------------------

     1430 1440 1450 1460 1470 1480
Ca   PQKILTANNSINGNVNGNSGTSTNKPYSALKFEQKLLIKGIDVNDDNDSSTKISNTIIEELS
Sc   LCEQQPSQEREFSLLVKAEIPPEYAKVGDFLLQYANNAVISHANAAAVYFADTSGLKISNSE
Af   RIREVPRGRPEKELLVNARVPPVLVPAVSTILRQTVPALKTEIDRITIYMGDYSWLGLSN--
Dm   ----------------PAEPDEMLLDECSLLSKQLLIPQPTKFNPTTLLSA-----------
Nc   QFIKKAQAHCELDMLLGANPPPCFREVLLKFFTQTLPVFKSHVDPAKLYFANYEVLEIEENP
Gg   ---------------HITEPDDTLIDECCLLPSQLLIPNIDWLPIN----------------
Mm   ---------------AASEPDEGLVDECCLLPSQLLVPNLDWLPASDG--------------
Hs   ---------------PASEPDEALVDECCLLPSQLLIPSLDWLPASDG--------------
```

Med16

```
     1490      1500      1510      1520      1530      1540      1550
Ca NMILDRYSISISRETKLSELMFYDTDWLNIGINKKEVPSYTIDALRKLIISDSSTSTSLAKG
Sc FFNPEIFHLLQPLE--EGLIIDTDKLPIK-NRTSKSFSKL--LYDDVTCDKLSVSEISDGK-
Af --DRRTEMYRRSRD-----VDIIKKIPCR-PTASALPETN--ANANANQNGKSSTQVQQRRR
Dm ----QGFAAVKSGQ--------LQFTSMEEPTCLQDMETEEVVFASSVKDGVSNLQLG-ARP
Nc RILAARKARRKYIDVFTKREIQFPTSSGDARGGGGGSGGGSAATATAAAAAAAAAAAGGEDD
Gg -----DGIISKLQNKQLVRLQFGKAPGLIGHTVSSQFDAFVRAPGQPKIDHLRRLHLGAYPT
Mm -------LVSRLQPKQPLRLRFGRAPTLPSSTSTLQLDGLTRAPGQPKIDHLRRLHLGAYPT
Hs -------LVSRLQPKQPLRLQFGRAPTLPGSAATLQLDGLARAPGQPKIDHLRRLHLGACPT

             1560      1570      1580      1590      1600      1610
Ca EKLRVCTRCRAVSLVGDVTGLWTMVFQRTCMCGNAWVNV-----------------------
Sc --LKRCSRCGSVTRAG--NIISSDKTIVPT--SIQTKRWPTMYTR----------LCICSGM
Af ----RCVRCCEVSSD--THPPRSLLSFRMIVKLGLLR------------------ACVCGGM
Dm SHIRRCARCG--------------FVFVNNASKVAKTSALKAWFS-------KWLHCHCGGF
Nc GKRGGITGTGEGQTPSQSTLAPGGGISIKIELGTGSTPNLKGVSTPAGGGGGGSSGNAKTAG
Gg EECKSCTRCGCVTMLK-----------SPNKVTAVKQWEQRWVKN-----------CLCGGL
Mm EECKACTRCGCVTMLK-----------SPNKTTAVTQWEQRWIKN-----------CLCGGL
Hs EECKACTRCGCVTMLK-----------SPNRTTAVKQWEQRWIKN-----------CLCGGL

           1620      1630      1640      1650      1660      1670
Ca --------------------------------------------------------------
Sc LFEMDG--------------------------------------------------------
Af WTLEPSVYSSAQPSGAPVGQATGRTPALVAAGLAGSS-------------------------
Dm WKQVH---------------------------------------------------------
Nc GNNAAAVGVNAANGGVGGGGGGGGNTNNNGTAENTSNQWRRCVRCTAVMEDFPGNHYRAGFT
Gg WRRMPLSYS-----------------------------------------------------
Mm WRRVPLSCS-----------------------------------------------------
Hs WWRVPLSYP-----------------------------------------------------

         1680      1690      1700
Ca -------------------------------
Sc -------------------------------
Af -------------------------------
Dm -------------------------------
Nc FVMAQQRKCACGAWWALVPKTTGGEGATEGW
Gg -------------------------------
Mm -------------------------------
Hs -------------------------------
```

Med17

```
   1        10        20        30        40        50        60
Mm ------------------------------------------------------------
Hs -------------------MGCVLGKLLGQLLCFQSERCVRFPEGLLRHRGCGLLSSRLS
Dm ------------------------------------------------------------
At ------------------------------------------------------------
Af ------------------------------------------------------------
Nc ------------------------------------------------------------
Ca ------------------------------------------------------------
Ce ------------------------------------------------------------
Sp ------------------------------------------------------------
Sc MTTEDPDSNHLSSETGIKLALDPNLITLALSSNPNSSLHSPTSDEPVPESAGKADTSIRL

            70        80        90        100       110       120
Mm -------------------------MSGVRAVRISIESACEKQVQEVGLDGTETYLQPLS
Hs AGKPPLRTSFFGSWGVLPPLADAASMSGVRAVRISIESACEKQVHEVGLDGTETYLPPLS
Dm ---------------------------MSNSVNISVETTCENQIREIGYDGTELYQPPPT
At --------------------------------------------------------MDSD
Af -------------------------------------------------------MADDR
Nc -----------------------MPLAFYGTTNNDTGTLHFQYLEVIIRTMDSPLNLRPR
Ca ----------------------------------------------------------PR
Ce -------------------MEDGDEVVEPIEPESEDIVNLTKRLGVDLVLESNDDWKIQE
Sp ---------------------------------MAEEANKDADISSLSLSLDPEIIGGQN
Sc EGDELENKTKKDNDKNLKFLKNKDSLVSNPHEIYGSMPLEQLIPIILRQRGPGFKFVDLN

            130       140       150       160       170       180
Mm MSQNLARLAQRIDFSQGSGSEEEEAAGPDGDAPDWGGAGAD----QDDEEGLVKFQP---
Hs MSQNLARLAQRIDFSQGSGSEEEEAAGTEGDAQDWPGAGSSAD--QDDEEGVVKFQP---
Dm LSESLAKCAARIDFSKTSLDDLKK-------------EEKSAAAAADEDKDATQFQES--
At MEISLDRLPIKRLESIEENGAERFPSDVDYDDKRVSLIRRIDFAWALEEEDELKKKKQKK
Af FTLPLRPLIEKRDRPDPLPLEIAQINAQWGSFRDVSEESLRAKIEEEKSKEYTIEEEEGE
Nc PPNSRGPQTIADFIRRVNAEPGGFRALNEEEVRRNVIAERNGLHHEDVEMVTDQEAD---
Ca DKLKLEELIPRILFERKSFLNVTEDSLRKEIDNSLKISEEDALDTEESREDTVEADQQE-
Ce IGFDGVERYLQPETFTDHVGKLARKVEWTKLVGTKSPYENSKVDPEDELDDEVEAGENQE
Sp NFLENNLQQIFQKIIQERGPFRDLKEEDLQKELQKESIKDESSAKSSETENVLEFATLDS
Sc EKELQNEIKQLGSDSSDGHNSEKKDTDGADENVQIGEDFMEVDYEDKDNPVDSRNETDHK

            190       200       210       220       230       240
Mm ------SLWPWDS-------------VRNNLRSALTE----MCVLYD----VLSIVRDKK
Hs ------SLWPWDS-------------VRNNLRSALTE----MCVLYD----VLSIVRDKK
Dm -------LWPWDA-------------VRNKLKDALTE----ICVLSD----VISIAKDKR
At SSKDSVEQWKWKG-------------MVENLQLAHQE----LTVIID----LIDTVQAND
Af GAGNHYSDVADRS-------------ILRSYRQAHMEAMFALDFVSM----LLSKHTPRQ
Nc --------DDSKKPDIIAARHNIIMSLGQAIQISSNF----LDFISL----LLSKEIPTQ
Ca ------VFNKHKF------------ELSKNINNALNETQLSLDFVSL----LISSVKPSL
Ce KSNNFREKENLKKILKNPSIIDFQNKKVEKFRFFRKKIFFFWRKSYNFEKKSTNFQFFQG
Sp KRNVNDTEVESMDS------QAYKKELIEQIMIAQTE----CSLALDMTSLLLSKFKENS
Sc TNENGETDDNIETVMTQEQFVKRRRDMLEHINLAMNESSLALEFVSL----LLSSVKEST

            250       260       270       280       290       300
Mm FMT---------------------------------------------------------
Hs FMT---------------------------------------------------------
Dm YLVLDP-------------------------------------------------LLEEA
At AVTVAG------------------------------------------------------
Af AETSMS---------------------------------------------------AYL
Nc AAVSISP-----------------------------------------------------
Ca AKSTIS------------------------------------------------------
Ce ILKNTLFFAIKRRFYQFLRFFAEKFVHFLMKLGKIPNVAKGGLNEELVTPEAGPWSSVAK
Sp IETIS-------------------------------------------------------
Sc GMSSMS------------------------------------------------------

            310       320       330       340       350       360
Mm -----------------------LDPVSQDALP--------------------PKQSPQ-
Hs -----------------------LDPVSQDALP--------------------PKQNPQ-
Dm DDTKPIVQVYS-------------------------------------------------
At -MTRPKP--------------MPNEILSDLAVS------------------TATKLQGYR
Af KQVAPLG---------------SLHSEIINPP---------------------PKSEAVV
Nc ---------------------WLRSQVPIGSIG--------------------ATQLDAP
Ca ----PHLS-------------KFVKPTSLNSDRLG----------------QDSNDNQES
Ce YLHESLNQLNSLLDVISVTKSTDYMKALTVLDPITVQEPTPETISTNRGTQWIWKRRALQ
Sp ----PFLKS------------TVPPSSLQFSR---------------------SQPPESK
Sc ----PFLR-------------KVVKPSSLNSDKIP----------------YVAPTKKEY

            370       380       390       400       410       420
Mm ------------TLQLIS------------KKKSLAGAA-----------------QILL
Hs ----------------TLQLIS--------KKKSLAGAA-----------------QILL
Dm ------------------------------RKKAISQAA-----------------QVLL
At YPEKLLET-----------LTW--------FRKELFNYIIVFGWYAYLTSEYLFPLQNLG
Af RDTKTVS------------RGW--------RLQSFNAAA-----------------DKLL
Nc TPLTQSR------------VADNKLITIGKRLVALNEAA-----------------DTAL
Ca KATDSFG------------QGW--------KLESLGKIT-----------------DLFR
Ce EAVQVLDMAQKQRQRASSNLGLSADYMAHLQRTKFFDFSMKNCVSTCFQAKSCKFEQKML
Sp ESDATLA------------KCWK--------EKSLTSSC-----------------KFLF
Sc IELDILN------------KGW--------KLQSLNESK-----------------DLLR

            430       440       450       460       470       480
Mm KGAERLTKSVAE--------NQENKLQR---------DFNSELLRLRQ---HWKLR---K
Hs KGAERLTKSVT--------ENQENKLQR---------DFNSELLRLRQ---HWKLR---K
Dm SGAERLRNAHSE---------QRNRNVS---------DFHIELLRLRQ---NWRLK---K
At NYFKQSAKALEQ---------KINREAR----------FYGALIRLQR---NWKVK----
Af KSASRLENEVAS-----------------------ETRYWHEVLAVKD--KGWKLC---R
Nc AAANRLRQEINS-----------------------ETKYWQEVLTVSQ--KGWSTA---R
Ca EASTNLNDQVIK----------ERRYWN---------MINLVLANDEV---LFRMRDPQN
Ce ENQNRLKKTLFFFGKLRCFLQNPHGKSSILAIFVENCGFLRELREMRE---IWRVR---K
Sp EAKERLTSVVET-----------------------EHEYYTELVKVKEAS----------
Sc ASFNKLSSILQN----------EHDYWN---------KIMQSISNKDV---IFKIRDR-T

            490       500       510       520       530       540
Mm VGDKILG-------------DLSYRSAGSLFPH-HGTFEVIKNTDIDLDKKIP-------
Hs VGDKILG-------------DLSYRSAGSLFPH-HGTFEVIKNTDLDLDKKIP-------
Dm VSNAIIG-------------DLSYRTAGSKFGM-SGTFEVTKAEETGD-EDTASSSNSSS
At -RQRMLASNASNEGFTIDLSDSSLYDPTSGFRP-STLSTIRVDHDSAGMLAINVPQDSWY
Af LPRQ-------------------GQTLGVQYGF-LEATPIFRDRGLAALRRS---EDGTL
Nc LPQEP-------------------HDMGVKFGF-SNAAPLFKNNSVAPLKRAE---DGSV
Ca NARAIGVK-------------YGYGDSGSNFHD-QGLALLRKDNQTGE-------ISFHP
Ce VGDYICG-------------DLSYHIFGWKYDT-PAIFDISRRSLSNN------------
Sp -------------------------------------WPLFNSQGSNHLSVQYS--CLGG
Sc SGQKLLAIK------------YGYEDSGSTYKHDRGIANIRNNIESQN--LDLIPHSSSV

            550       560       570       580       590       600
Mm ----------EDYCP-LDVQIPSDLEGSAYIKVSIQKQAP--DIGDLG----TVNLFKRP
Hs ----------EDYCP-LDVQIPSDLEGSAYIKVSIQKQAP--DIGDLG----TVNLFKRP
Dm SVSGNNGMQLKASSA-LRVIVPAELQGVAYIKVITQKDQEDLCTAQLN--------LMGH
At SLRFG-------------------YVGLNPIGNSN-ESDEHIDSTTG-----HDIPGTSE
Af ILDKGLVPTKAKTVR-VRVKDCGIITGCSKPYRSAAQDPD---SIEGR------------
Nc RLEYGRMGSKSERLQ--------------------------ATLLHNG-----EVVGRSS
Ca ISSINNAKIVEKVSRFIRVKILSQIDG------------DYMLTGQSIFN-------FDF
Ce ----------MENLSIIEVSVPKDLARRSMLAVSIVQDDIQSGNGLFRDPKDKKYTYSYR
Sp ISLGLGLIRMKPSKS--FEVQSSLLYSQAALKISILNKDR-DEIGSST-----WSWPSQN
Sc FKGTDFVHSVKKFLR-------------VRIFTKIESEDDYILSGESVMD----------
```

Med17

```
                                        610                 620                 630                 640                 650                 660
Mm   L P K S K P G S P - - H W Q T K - - - L E A A Q N V L L C K E - I F A Q L S R E A V Q I K S Q I P H I V V K N Q I I S Q
Hs   L P K S K P G S P - - H W Q T K - - - L E A A Q N V L L C K E - I F A Q L S R E A V Q I K S Q V P H I V V K N Q I I S Q
Dm   G P N I T A Q V G - - V W Q K T - - - L E F A Q N V L F C K E - L F A Q L A R E A I Q L Q A P I P H V V I G N Q I R A T
At   - K L S A S D D K - - Y V K E T H S L L R E V H K S I F A E Q V L F D M L N R E A F N E G V G F N I S G L R E N F M E M
Af   - - - - - - - - - - - - - - - - - - - I L Q A R D T L Y E E E - L F H E L F R E A R I M G S Q G - - V T T R Q N L V Q F
Nc   L P R P L P D D A P - - L D D R - - - - V K E A R N T I F A Q E - L W H E I N R E G R T L H G H - - H V R L E Q S A V T C
Ca   E K S K Q S I I N - - - - - - - - - - D I E K A R F F L F E E D - L F H Q L I R E A K L L V N Y N V S I I S N K I I I E I
Ce   - E S D S E K V K L L H W K D S - - - L K W A Q N T L L L R D - V F K T G E K K D F S E K A Q K N L F L Y R I F N H Y F
Sp   - C N S V L L K D - - - - - - - - - - I Y K L Q E I L F E M D - I W N S L L Q E A Q S C G N Q G V N F T G D E I L V P I
Sc   R D S E S E E A E T K D I R K Q - - - I Q L L K K I I F E K E - L M Y Q I K K E C A L L I S Y G V S I E N E N K V I I E

                                        670                 680                 690                 700                 710                 720
Mm   P F - - P S L Q L S I S L C H S S D - - - - - - - - - D K K S Q K C A A E K P G Q E D H L Y V L E H N L H L L I R E F H
Hs   P F - - P S L Q L S I S L C H S S N - - - - - - - - - D K K S Q K F A T E K Q C P E D H L Y V L E H N L H L L I R E F H
Dm   L L - - P N I Q L I I S L C H S T T F D - - - - - - - - - - - - - - - S S Q P A P I N D H D H V L E H S L H Q L L R E V H
At   S I G Q G A S L F V S L H P S G K N P S I K K S E S A T L L I E S S G R V E P A E G G D Y R L K K L G F P N R T S Y E I
Af   P V - S E E Q E I L L D L V D P D Q E V Y V D D - - - - E A T K S E E H N - - - - - V L A D A L A H L I R I L L C Y A H
Nc   A L D P N R T - I S F E L V G L D D Q - - - - - - - - D H S R A P L P G D - - - - - L D A E T A S I T L H L L L S N A H
Ca   N N I I I E I E S I V Y D E L N E E E L E N Y Y Q N V N E Y S T L H N K K C Q L I L N Y L K L M L C C Y Y K Y N L K L K
Ce   L V L F F L I K V K I F F S I F K K K V I S H T E K I E K K R L D F - - - - - - - - - - - - - - - - - - - - - - - - - -
Sp   S D D H V V R I T L E T S S K N T E S G - - - - - - - F T E D K K S N E D T S T N F V T I K Q E K E L L K C L C D T L
Sc   L P N E K F E I E L L S L D D D S I V N H E Q D L P K I N D K R A N L M L V M L R L L L V V I F K K T L R S R I S S P H

                                        730                 740                 750                 760                 770                 780
Mm   K Q T L S S I V M P H P A S A P F G H K R M R - L S G P Q A F D K N E I N S I Q S T E G L L E K I I K Q A K H I F L R S
Hs   K Q T L S S I M M P H P A S A P F G H K R M R - L S G P Q A F D K N E I N S L Q S S E G L L E K I I K Q A K H I F L R S
Dm   Y K N - S H H P F P H P A S A P L G P T K K R M L A G P M A A D R E T L L D M T K S Q T I L E Q I I A Q A Q H I F M R K
At   Y L Q Q I F H E H A F G K A K D Q L K S K S I R A S N Q T E K D S N S G L L D H F C L S L T H R I F S N R V L V H L E S
Af   R Q N L R R R T Q P P P A L S P K R R H I P E Y Q L L R P I M A Y L Q H S F H V R W L E T F M K D V Y G V L Q S A G L S
Nc   R Q N Q L K R S E R T A A N A M R G P P P P Y N L L L P I I T Y Y R H E K T L E D C T T F L A A F C G A L R S A G I Q S
Ca   Q K V P T A L T K W K Q S N S H P L I L R P L V G N M R H E L N L L N M K S V L D R L M H A H E S E L S Y S K L D V E K
Ce   - - - - - - - - - - - - - - - - - - - - - - - - - - - - - - - - - - - - - - - - - - - - - - - - - - - - - - - - - - - -
Sp   N A I A H I L F L K H C R K S D R R S Q Q P E L Y M A I D A N A P L I L R P L I F Y Y N L N Q E S L E F Q R W L K Q R D
Sc   G L I N L N V D D D I L I I R P I L G K V R F A N Y K L L L K K I I K D Y V L D I V P G S S I T E T E V E R E Q P Q E N

                                        790                 800                 810                 820                 830                 840
Mm   R T A A T I D S L A S R I E D P Q I Q A H W S N I N D V Y E S S V K V L I T S Q G Y E Q I C K S I Q L Q L N I G V E Q V
Hs   R A A A T I D S L A S R I E D P Q I Q A H W S N I N D V Y E S S V K V L I T S Q G Y E Q I C K S I Q L Q L N I G V E Q I
Dm   R T Q Y V L D T L A R D V K D P Q I V S H W N A M N S P T M S C V K I N I V T H G Y D A I G R - T S L V I H V K E R S L
At   V V C K V P Y L H L I S H P T W N S R T S S W T V F M T V P P S I I P Q G R S E T Q S P D G K R N L K T Q F R T K V V V
Af   C S F T A T P Y S S V N L S N I D R S V P K V E G L I R Q F L L P L E S T F S A D L L T P Q S S F K V K S R T N L S V P
Nc   S F S M T E S I N K G P P T A P P S E A L M K T L L H P S E V Q F D L T I T P A S R V R I L A K P T P V F G T R F S I Y
Ca   F I N L A T R S K K Q N P F Q K S I E K P I S K F H L V L C N K T S N M L D V N I Q L T T N E L F V N L I I N M T I I R
Ce   - - - - - - - - - - - - - - - - - - - - - - - - - - - - - - - - - - - - - - - - - - - - - - - - - - - - - - - - - - - -
Sp   I S F K F M P N Y P W E K A K D F L E L E N S L S I N R L S I S W R I M V S N F E P A I F I Q H T P T L H G T D K S V W
Sc   K N I D D E N I T K L N K E I R A F D K L L N I P R R E L K I N L P L T E H K S P N L S L M L E S P N Y C N A L I H I K

                                        850                 860                 870                 880                 890                 900
Mm   R V V H R D G R V I M L S H Q E Q E L Q D F L L S Q M S Q H Q V H A V Q Q L A K V M G W Q V L S F S N H V G L G P I E S
Hs   R V V H R D G R V I T L S Y Q E Q E L Q D F L L S Q M S Q H Q V H A V Q Q L A K V M G W Q V L S F S N H V G L G P I E S
Dm   K C I C R D G R V M R L S Y E P Q E L R D L I L C Q I N S H Q I S C L I S L A R C M S W T V L S N S N H L G I G K V E P
At   K D D C I S V E A E C T P N V V G L L K S S S C N L F S I N K Y E C D V A D L P V M I L Q Q V A S Q I V C W L L E E A R
Af   P F G T Q F E I L L N L P H Y P D V H P P H R I G L H D Q A A N I I T H F I M L D I V A A I E L Q T S P A L S I S K T E
Nc   L L H P Q S N H L T S S F P P N Q T D S V Y E N I K E L V R Y L S N A V P R A L T M Y Y Y P L V Q E M R N S G N K G N N
Ca   F E T E D D F K N N V N G I N V L Q L G F S D F N E I E E C L D W S I Q N F V - - - - - - - - - - - - - - - - - - - - -
Ce   - - - - - - - - - - - - - - - - - - - - - - - - - - - - - - - - - - - - - - - - - - - - - - - - - - - - - - - - - - - -
Sp   R C K D Q Y S S N Q F S S L K N V C Q Y I E H H I N S L S R R S K K - - - - - - - - - - - - - - - - - - - - - - - - - -
Sc   F S A G T E A N A V S F D T T F S D F K E V E D F L H F I V A E Y I Q Q K K V - - - - - - - - - - - - - - - - - - - - -

                                        910                 920                 930                 940                 950                 960
Mm   I G N A S A I T V A S P S G D Y A I S V R N G P E S G S K I M V Q F P R N Q C K D L P K S D V L Q D S K W S H L R G P F
Hs   I G N A S A I T V A S P S G D Y A I S V R N G P E S G S K I M V - - - - - - - - - - - - - - - - - - - - - - - - - - - -
Dm   L G N A S S C L L A S P N S D R M I A V Q I R C D P Q I D V K V Y I A R S P R Q D F F P S P L V P E K L W E N L G G T F
At   T V G T K A S R E F L S L S L E I V E G E R V S L V A H V N P E D A K G C I S W W L V M E N G C T E E R E G V S E S R K
Af   A K S V S W E A T Y P H H G E L L S V S I D G K Q K K M K V I L S R D E L T I Q T Y D V L G L E R Y S R A A P E T T P G
Nc   G E T P N P P A P N T T T W I I H P D D L G L V D D D T E T F G V R F A F T S N Y T R G D E V G D A A K E P E L R V T G
Ca   - - - - - - - - - - - - - - - - - - - - - - - - - - - - - - - - - - - - - - - - - - - - - - - - - - - - - - - - - - - -
Ce   - - - - - - - - - - - - - - - - - - - - - - - - - - - - - - - - - - - - - - - - - - - - - - - - - - - - - - - - - - - -
Sp   - - - - - - - - - - - - - - - - - - - - - - - - - - - - - - - - - - - - - - - - - - - - - - - - - - - - - - - - - - - -
Sc   - - - - - - - - - - - - - - - - - - - - - - - - - - - - - - - - - - - - - - - - - - - - - - - - - - - - - - - - - - - -

                                        970                 980                 990                 1000
Mm   K E V Q W N K M E G R N F V Y K M E L L M S A L S P C L L - - - - - - - - - - - - - - - - - - - - - - - - - - -
Hs   - - - - - - - - - - - - - - - - - - - - - - - - - - - - - - - - - - - - - - - - - - - - - - - - - - - - - - - -
Dm   K E V R F D K I E G K S F L N K M E F L M A S L T S N T A - - - - - - - - - - - - - - - - - - - - - - - - - - -
At   L L G H L S L D V L Y S V L M D L I N L C G T G R N A L E R L - - - - - - - - - - - - - - - - - - - - - - - - -
Af   L Q T H T W K A G P T T A P S L M E Y V A A V S Q R - - - - - - - - - - - - - - - - - - - - - - - - - - - - - -
Nc   D Y M E D G K R V Q H E W K W T A S G P A G T Q S G G S L D E I V K H I L A N G P S S S V L S A
Ca   - - - - - - - - - - - - - - - - - - - - - - - - - - - - - - - - - - - - - - - - - - - - - - - - - - - - - - - -
Ce   - - - - - - - - - - - - - - - - - - - - - - - - - - - - - - - - - - - - - - - - - - - - - - - - - - - - - - - -
Sp   - - - - - - - - - - - - - - - - - - - - - - - - - - - - - - - - - - - - - - - - - - - - - - - - - - - - - - - -
Sc   - - - - - - - - - - - - - - - - - - - - - - - - - - - - - - - - - - - - - - - - - - - - - - - - - - - - - - - -
```

Med18

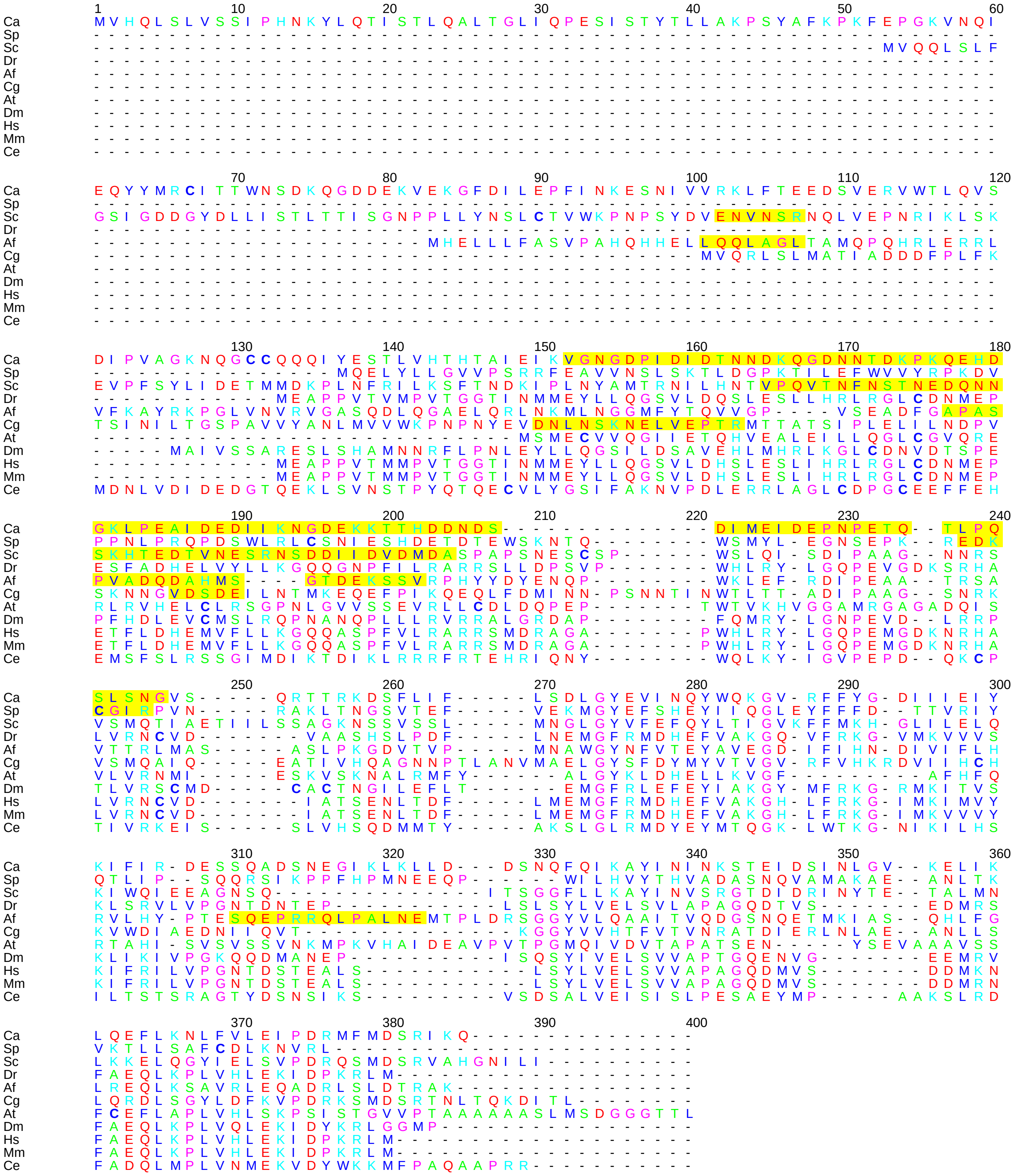

Med19

```
         1        10        20        30        40        50        60
Dr       MTEIFSSLYGQPDSQGPAGPSALGFGSGKPQVPQNMGPMCFPHQMMEEGAPVRKP-----
Hs       --MENFTALFGAQADPPPPPTALGFGPGKPPPPPPPPAGGGPGTAPPPTAATAPPGADKS
Mm       ----------------------------------PPPPGGGPGAAPPSTATSAPAGADKS
Ce       --------------------------------------------MNEPSACSGPPVKSKS

                  70        80        90       100       110       120
Dr       AAMNEPFYLLRELPMENELTGHTNLITHYNLEHAYNKFCG-KKVKEKLSNFLPELPGMID
Hs       GAGCGPFYLMRELPGSTELTGSTNLITHYNLEQAYNKFCG-KKVKEKLSNFLPDLPGMID
Mm       TAGSGPFYLMRELPGSTELTGSTNLITHYNLEQAYNKFCG-KKVKEKLSNFLPDLPGMID
Ce       SKNEPFYTLKALLPPYSEIQGNHDLLMSYELGPVEGGFSGSRRVKEKISSFLPHIIGEFH

                 130       140       150       160       170       180
Dr       SPGIQDNSSLRSLIEKPPVCNNSFSPLTGAMLTGFRLHTGPLPEQYRLMHIQPPKKKNKH
Hs       LPGSHDNSSLRSLIEKPPILSSSFNPITGTMLAGFRLHTGPLPEQCRLMHIQPPKKKNKH
Mm       LPGSHDNSSLRSLIEKPPILGGSFNPITGTMLSGFRLHTGPLPEQCRLMHIQPPKKKNKH
Ce       LDATKEASSLRALIEKPPIHK-EISNLTSSSMQGFKLSAGPVDERYRHLFERRKEEPNLA

                 190       200       210       220       230       240
Dr       KHKHHRPQDPLPPETPSDSDHKKKKKKKDDDPDRKKKKKDKKKKKNRHSPDHPGMTGAQP
Hs       KHKQSRTQDPVPPGKPS-------------------------------------------
Mm       KHKQSRTQDPVPPETPSDSDHKKKKKKKEEDPERKRKKKEKKKKKNRHSPDHPGMGSSQA
Ce       YSEKLNLIRVRQQYDAYG---FDDDETEKGFPKKHKKKKKDKKRKKEKEELGDAEKRKRA

Dr       STSSLR-
Hs       -------
Mm       SSSSSLR
Ce       ADEPMEF
```

Med20

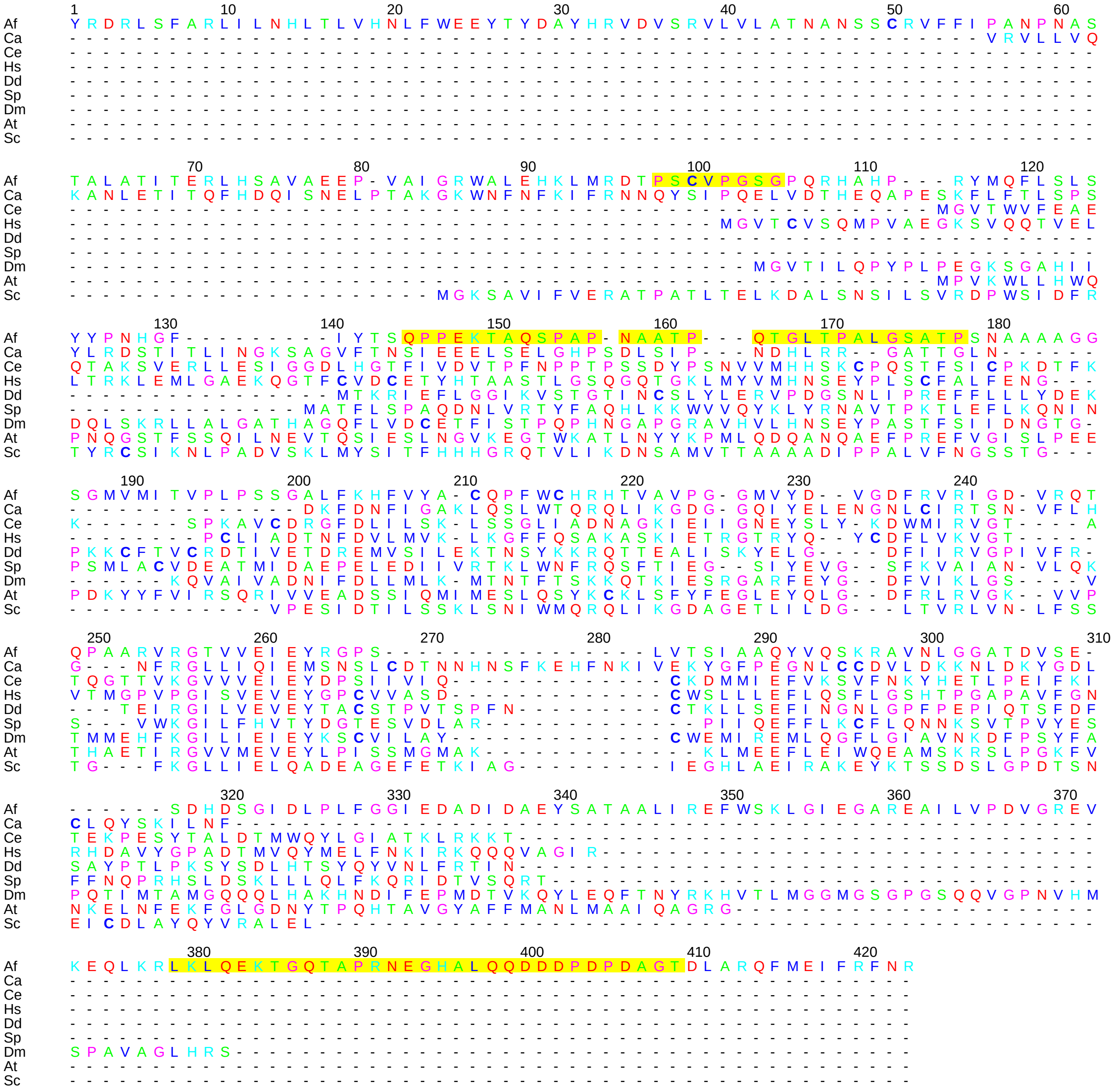

Med21

```
    1                   10                  20                  30                  40                  50                  60
Gg  M A P P F C S H A G S P G G A S G C R G D S R P C D R R R E I K R R R K P S V C C E S A A L S S A Q G C R P L N A A L L
Pt  - - - - - - - - - - - - - - - - - - - - - - - - - - - - - - - - - - - - - - - - - - - - - - - - - - - - - - - - - - - -
Mm  - - - - - - - - - - - - - - - - - - - - - - - - - - - - - - - - - - - - - - - - - - - - - - - - - - - - - - - - - - - -
Cf  - - - - - - - - - - - - - - - - - - - - - - - - - - - - - - - - - - - - - - - - - - - - - - - - - - - - - - - - - - - -
Hs  - - - - - - - - - - - - - - - - - - - - - - - - - - - - - - - - - - - - - - - - - - - - - - - - - - - - - - - - - - - -
Rn  - - - - - - - - - - - - - - - - - - - - - - - - - - - - - - - - - - - - - - - - - - - - - - - - - - - - - - - - - - - -
Bt  - - - - - - - - - - - - - - - - - - - - - - - - - - - - - - - - - - - - - - - - - - - - - - - - - - - - - - - - - - - -
Dr  - - - - - - - - - - - - - - - - - - - - - - - - - - - - - - - - - - - - - - - - - - - - - - - - - - - - - - - - - - - -
Su  - - - - - - - - - - - - - - - - - - - - - - - - - - - - - - - - - - - - - - - - - - - - - - - - - - - - - - - - - - - -
Os  - - - - - - - - - - - - - - - - - - - - - - - - - - - - - - - - - - - - - - - - - - - - - - - - - - - - - - - - - - - -
Dm  - - - - - - - - - - - - - - - - - - - - - - - - - - - - - - - - - - - - - - - - - - - - - - - - - - - - - - - - - - - -
At  - - - - - - - - - - - - - - - - - - - - - - - - - - - - - - - - - - - - - - - - - - - - - - - - - - - - - - - - - - - -
Af  - - - - - - - - - - - - - - - - - - - - - - - - - - - - - - - - - - - - - - - - - - - - - - - - - - - - - - - - - - - -
Sb  - - - - - - - - - - - - - - - - - - - - - - - - - - - - - - - - - - - - - - - - - - - - - - - - - - - - - - - - - - - -
Ca  - - - - - - - - - - - - - - - - - - - - - - - - - - - - - - - - - - - - - - - - - - - - - - - - - - - - - - - - - - - -
Cg  - - - - - - - - - - - - - - - - - - - - - - - - - - - - - - - - - - - - - - - - - - - - - - - - - - - - - - - - - - - -
Sc  - - - - - - - - - - - - - - - - - - - - - - - - - - - - - - - - - - - - - - - - - - - - - - - - - - - - - - - - - - - -
Sp  - - - - - - - - - - - - - - - - - - - - - - - - - - - - - - - - - - - - - - - - - - - - - - - - - - - - - - - - - - - -
Nc  - - - - - - - - - - - - - - - - - - - - - - - - - - - - - - - - - - - - - - - - - - - - - - - - - - - - - - - - - - - -
Ce  - - - - - - - - - - - - - - - - - - - - - - - - - - - - - - - - - - - - - - - - - - - - - - - - - - - - - - - - - - - -
Dd  - - - - - - - - - - - - - - - - - - - - - - - - - - - - - - - - - - - - - - - - - - - - - - - - - - - - - - - - - - - -

                        70                  80                  90                  100                 110                 120
Gg  R S T V T A D P L P A R P P R L S F T A R P S A S L W K S E Q G N A F L K E I S I P I S R V A A L S S H L S Q Y L S R R
Pt  - - - - - - - - - - - - - - - - - - - - - - - - - - - - - - - - - - - - - - - - - - - - - - - - - - - - - - - - - - - -
Mm  - - - - - - - - - - - - - - - - - - - - - - - - - - - - - - - - - - - - - - - - - - - - - - - - - - - - - - - - - - - -
Cf  - - - - - - - - - - - - - - - - - - - - - - - - - - - - - - - - - - - - - - - - - - - - - - - - - - - - - - - - - - - -
Hs  - - - - - - - - - - - - - - - - - - - - - - - - - - - - - - - - - - - - - - - - - - - - - - - - - - - - - - - - - - - -
Rn  - - - - - - - - - - - - - - - - - - - - - - - - - - - - - - - - - - - - - - - - - - - - - - - - - - - - - - - - - - - -
Bt  - - - - - - - - - - - - - - - - - - - - - - - - - - - - - - - - - - - - - - - - - - - - - - - - - - - - - - - - - - - -
Dr  - - - - - - - - - - - - - - - - - - - - - - - - - - - - - - - - - - - - - - - - - - - - - - - - - - - - - - - - - - - -
Su  - - - - - - - - - - - - - - - - - - - - - - - - - - - - - - - - - - - - - - - - - - - - - - - - - - - - - - - - - - - -
Os  - - - - - - - - - - - - - - - - - - - - - - - - - - - - - - - - - - - - - - - - - - - - - - - - - - - - - - - - - - - -
Dm  - - - - - - - - - - - - - - - - - - - - - - - - - - - - - - - - - - - - - - - - - - - - - - - - - - - - - - - - - - - -
At  - - - - - - - - - - - - - - - - - - - - - - - - - - - - - - - - - - - - - - - - - - - - - - - - - - - - - - - - - - - -
Af  - - - - - - - - - - - - - - - - - - - - - - - - - - - - - - - - - - - - - - - - - - - - - - - - - - - - - - - - - - - -
Sb  - - - - - - - - - - - - - - - - - - - - - - - - - - - - - - - - - - - - - - - - - - - - - - - - - - - - - - - - - - - -
Ca  - - - - - - - - - - - - - - - - - - - - - - - - - - - - - - - - - - - - - - - - - - - - - - - - - - - - - - - - - - - -
Cg  - - - - - - - - - - - - - - - - - - - - - - - - - - - - - - - - - - - - - - - - - - - - - - - - - - - - - - - - - - - -
Sc  - - - - - - - - - - - - - - - - - - - - - - - - - - - - - - - - - - - - - - - - - - - - - - - - - - - - - - - - - - - -
Sp  - - - - - - - - - - - - - - - - - - - - - - - - - - - - - - - - - - - - - - - - - - - - - - - - - - - - - - - - - - - -
Nc  - - - - - - - - - - - - - - - - - - - - - - - - - - - - - - - - - - - - - - - - - - - - - - - - - - - - - - - - - - - -
Ce  - - - - - - - - - - - - - - - - - - - - - - - - - - - - - - - - - - - - - - - - - - - - - - - - - - - - - - - - - - - -
Dd  - - - - - - - - - - - - - - - - - - - - - - - - - - - - - - - - - - - - - - - - - - - - - - - - - - - - - - - - - - - -

                        130                 140                 150                 160                 170                 180
Gg  V A V P R R P Q H S C S R T P V A A K Q D A S Q R T C P G L C S L L L A G R G R A W P G P P E G G Q R P A P P T G R R L
Pt  - - - - - - - - - - - - - - - - - - - - - - - - - - - - - - - - - - - - - - - - - - - - - - - - - - - - - - - - - - - -
Mm  - - - - - - - - - - - - - - - - - - - - - - - - - - - - - - - - - - - - - - - - - - - - - - - - - - - - - - - - - - - -
Cf  - - - - - - - - - - - - - - - - - - - - - - - - - - - - - - - - - - - - - - - - - - - - - - - - - - - - - - - - - - - -
Hs  - - - - - - - - - - - - - - - - - - - - - - - - - - - - - - - - - - - - - - - - - - - - - - - - - - - - - - - - - - - -
Rn  - - - - - - - - - - - - - - - - - - - - - - - - - - - - - - - - - - - - - - - - - - - - - - - - - - - - - - - - - - - -
Bt  - - - - - - - - - - - - - - - - - - - - - - - - - - - - - - - - - - - - - - - - - - - - - - - - - - - - - - - - - - - -
Dr  - - - - - - - - - - - - - - - - - - - - - - - - - - - - - - - - - - - - - - - - - - - - - - - - - - - - - - - - - - - -
Su  - - - - - - - - - - - - - - - - - - - - - - - - - - - - - - - - - - - - - - - - - - - - - - - - - - - - - - - - - - - -
Os  - - - - - - - - - M G P R A S Q R V A C A L S M C W A H Q F S L M G L D V I G P W W I E R R R R E K E R E K R R R R R S
Dm  - - - - - - - - - - - - - - - - - - - - - - - - - - - - - - - - - - - - - - - - - - - - - - - - - - - - - - - - - - - -
At  - - - - - - - - - - - - - - - - - - - - - - - - - - - - - - - - - - - - - - - - - - - - - - - - - - - - - - - - - - - -
Af  - - - - - - - - - M A D I L T Q L Q T C L D Q L A T Q F Y A T I G Y L S T Y H D N S P A I P P P D V P D A A P A L A K I
Sb  - - - - - - - - - - - - - - - - - - - - - - - - - - - - - - - - - - - - - - - - - - - - - - - - - - - - - - - - - - - -
Ca  - - - - - - - - - - - - - - - - - - - - - - - - - - - - - - - - - - - - - - - - - - - - - - - - - - - - - - - - - - - -
Cg  - - - - - - - - - - - - - - - - - - - - - - - - - - - - - - - - - - - - - - - - - - - - - - - - - - - - - - - - - - - -
Sc  - - - - - - - - - - - - - - - - - - - - - - - - - - - - - - - - - - - - - - - - - - - - - - - - - - - - - - - - - - - -
Sp  - - - - - - - - - - - - - - - - - - - - - - - - - - - - - - - - - - - - - - - - - - - - - - - - - - - - - - - - - - - -
Nc  - - - - - - - - - - - - - - - - - - - - - - - - - - - - - - - - - - - - - - - - - - - - - - - - - - - - - - - - - - - -
Ce  - - - - - - - - - - - - - - - - - - - - - - - - - - - - - - - - - - - - - - - - - - - - - - - - - - - - - - - - - - - -
Dd  - - - - - - - - - - - - - - - - - - - - - - - - - - - - - - - - - - - - - - - - - - - - - - - - - - - - - - - - - - - -

                        190                 200                 210                 220                 230                 240
Gg  T P C P A P L L S A M A D R L T Q L Q D A V N S L A D Q F C N A I G V L Q Q C G P P A S F S N I Q T A I N K D Q P A N P
Pt  - - - - - - - - - - M A D R L T Q L Q D A V N S L A D Q F C N A I G V L Q Q C G P P A S F N N I Q T A I N K D Q P A N P
Mm  - - - - - - - - - - M A D R L T Q L Q D A V N S L A D Q F C N A I G V L Q Q C G P P A S F S N I Q T A I N K D Q P A N P
Cf  - - - - - - - - - - M A D R L T Q L Q D A V N S L A D Q F C N A I G V L Q Q C G P P A S F S N I Q T A I N K D Q P A N P
Hs  - - - - - - - - - - M A D R L T Q L Q D A V N S L A D Q F C N A I G V L Q Q C G P P A S F N N I Q T A I N K D Q P A N P
Rn  - - - - - - - - - - M A D R L T Q L Q D A V N S L A D Q F C N A I G V L Q Q C G P P A S F S N I Q T A I N K D Q P A N P
Bt  - - - - - - - - - - M A D R L T Q L Q D A V N S L A D Q F C N A I G V L Q Q C G P P A S F S N I Q T A I N K D Q P A N P
Dr  - - - - - - - - - - M A D R L T Q L Q D A V N S L A D Q F C N A I G V L Q Q C G P P A S F S N I Q T A I N K D Q P S N P
Su  - - - - - - - - - - M A D R V T Q L Q D A V N Q L A E H M C N S I G V L Q Q S A Q P S K F P G C E K Q A S Q E Q Q P N H
Os  P A A R R G T R S P E M D I I S Q L Q E Q L N E M A M V A V N T F G T L Q R D A P P V R L S N N Y P D P L N P A A A A A
Dm  - - - - - - - - - - M A D R L T Q L Q D T V N Q Q A E H F C N A I G V I Q Q T S L P S K F V N F E R I G P Q T P I P C P
At  - - - - - - - - - - - M D I I S Q L Q E Q V N T I A A I T F N A F G T L Q R D A P P V Q L S P N Y P E P P A T T T V T D
Af  P K N S T A P P V P A G A P V P A L S Q A S P P A Q N P A H G A A G A G T S V G E G A Q T P G P A A G A G A D P N L P A
Sb  - - - - - - - - - - - M D I I T Q L Q D Q L D E M A V L A V N T F G T L Q R D A P P D R L S N S Y P D P L N P N P K P E
Ca  - - - - - - - - - - M A D R L T Q L Q I C L D Q L I Q Q F N S T I N Y V N T S A E P S L L D D D D V N S Y S N M A A N A
Cg  - - - - - - - - - - M T D R L T Q L Q R C L D Q V M E Q F C A T L N F I D K R H D F E P F N D K E P K M T D K H A S V A
Sc  - - - - - - - - - - M T D R L T Q L Q I C L D Q M T E Q F C A T L N Y I D K N H G F E R L T V N E P Q M S D K H A T V V
Sp  - - - - - - - - - - M A C R C T Q L Q D T I D E V A T Q F Y S S I H Y L S S H H D F V P L P G Q E K V S D S K V N P I S
Nc  - - - - - - - - - - M S D R L S Q L Q E A V D Q L M E Q F I A T Y F Y I D R H H D L K T F S P K D T I A P S K A D Q P P
Ce  - - - - - - - - - - M A D R M T Q L Q D M I N E M A G L M T N A I G V L Q A T A P P C E F G A I S Q E L E D E P N - - -
Dd  - - - - - - - - - - - M D L I T Q L Q D K L D H L L Y V F G T C I G V L Q R D A P P S F F N N P Q N Q Q Q Q Q Q Q Q Q Q

                        250                 260                 270                 280                 290                 300
Gg  T E E Y A Q L - - - - - - - - - - - - - - - - - - - - - - - - - - - - - - - - - - - - - - - - - - - - - - - - - F A A
Pt  T E E Y A Q L - - - - - - - - - - - - - - - - - - - - - - - - - - - - - - - - - - - - - - - - - - - - - - - - - F A A
Mm  T E E Y A Q L - - - - - - - - - - - - - - - - - - - - - - - - - - - - - - - - - - - - - - - - - - - - - - - - - F A A
Cf  T E E Y A Q L - - - - - - - - - - - - - - - - - - - - - - - - - - - - - - - - - - - - - - - - - - - - - - - - - F A A
Hs  T E E Y A Q L - - - - - - - - - - - - - - - - - - - - - - - - - - - - - - - - - - - - - - - - - - - - - - - - - F A A
Rn  T E E Y A Q L - - - - - - - - - - - - - - - - - - - - - - - - - - - - - - - - - - - - - - - - - - - - - - - - - F A A
Bt  T E E Y A Q L - - - - - - - - - - - - - - - - - - - - - - - - - - - - - - - - - - - - - - - - - - - - - - - - - F A A
Dr  T E E Y A Q L - - - - - - - - - - - - - - - - - - - - - - - - - - - - - - - - - - - - - - - - - - - - - - - - - F A A
Su  E D Y T Q L - - - - - - - - - - - - - - - - - - - - - - - - - - - - - - - - - - - - - - - - - - - - - - - - - - F A Q
Os  A N P N P D D P A Q P Q P G A A A A A A A G - - - - - - - - - - - - - - - - A P A A Q A Q A P P A Q A Q P P A L D L A E
Dm  P Q E D Y A Q - - - - - - - - - - - - - - - - - - - - - - - - - - - - - - - - - - - - - - - - - - - - - - - - L F A Q
At  D A T P - - - - - - - - - - - - - - - - - - - - - - - - - - - - - - - - - - - - - - - - - - - - - - - - - - - - - F P E
Af  P D S P R T - - - - - - - - - - - - - - - - - - - - - - - - - - - - - - - - - - - - - - - - - - - - - - - - - - F A S
Sb  D V S T K P Q V Q G Q - - - - - - - - - - - - - - - - - - - - - - - - - - - - - - - P G A P P P A Q A Q P P A P D L S E
Ca  P L P Q S Q Q Q R Q Q Q K K Q Q E P Q Q E I E Q P - - - - - - - - - - - - - Q Q Q S N P E S K S I S P P K E K V S F D N
Cg  T P E E F S N - - - - - - - - - - - - - - - - - - - - - - - - - - - - - - - - - - - - - - - - - - - - - - - - - - - - -
Sc  P P E E F S N - - - - - - - - - - - - - - - - - - - - - - - - - - - - - - - - - - - - - - - - - - - - - - - - - - - - -
Sp  A E E L Q F A - - - - - - - - - - - - - - - - - - - - - - - - - - - - - - - - - - - - - - - - - - - - - - - - - - - - -
Nc  E V D T L P P - - - - - - - - - - - - - - - - - - - - - - - - - - - - - - - - - - - - - - - - - - - - - - - - D V F Q A
Ce  - - - - - - - - - - - - - - - - - - - - - - - - - - - - - - - - - - - - - - - - - - - - - - - - - - - - - - C A I F A A
Dd  Q Q Q Q Q Q Q L L Q Q Q N P Q T Q Q Q L P P P P P P P P Q Q Q Q Q Q Q Q Q Q Q Q Q Q Q Q Q Q Q Q Q Q Q Q P Q P T E E W D
```

Med21

```
            310       320       330       340       350       360
Gg    LIARTAKD-------IDVLIDSLPSEESTAALQAASLYRLE------------------E
Pt    LIARTAKD-------IDVLIDSLPSEESTAALQAASLYKLE------------------E
Mm    LIARTAKD-------IDVLIDSLPSEESTAALQAASLYKLE------------------E
Cf    LIARTAKD-------IDVLIDSLPSEESTAALQAASLYKLE------------------E
Hs    LIARTAKD-------IDVLIDSLPSEESTAALQAASLYKLE------------------E
Rn    LIARTAKD-------IDVLIDSLPSEESTAALQAASLYKLE------------------E
Bt    LIARTAKD-------IDVLIDSLPSEESTAALQAASLYKLE------------------E
Dr    LIARTAKD-------VDVLIDSLPSEESTAALQAASLRQLE------------------E
Su    LIARTAKD-------IDVLIDSLPSEESTVELQNASLQRLE------------------E
Os    HPKAMSHALVLAAKKFDALVSALP--LSSEEDQLKRIKELQAEN-----------EVVGT
Dm    LIARCAKD-------IDTLIESLPNEDSSIELQNSSLKRLEIE-----------NQGTAR
At    QPKQLSAGLVKAAKQFDALVAALPLSEGGEGAQLKRIAELQVKQVTPICRVENDLVGQEL
Af    RQRELARDLIIKEQQIEYLISVLPGIDSSEAEQEKRIRELEG-----------ELRQVEE
Sb    QPKAMSHALVLAAKKFDALVAALP--LSSEEDQVKRIQELQAEN-----------EVVGL
Ca    VINELCTDLILKSRQIKMLIDSLPGIGVTPNEQMNLINELS-----------DKLQAIEE
Cg    GIDELSTDIIVKIRQITTLIDSLPGVGVSAEEQLHKIDSLQK-----------KLVDIED
Sc    TIDELSTDIILKTRQINKLIDSLPGVDVSAEEQLRKIDMLQK-----------KLVEVED
Sp    -QRDLAKDLVTKFMQIDTLINQLPGISTAPKHQLEKIKKLQN-----------SIEEKQL
Nc    GQLELARDLITREQQIEYLISSLPGLDNSEQDQLQSIKELEEELN-----------VAEK
Ce    GIAKAAKN-------IEILIDSFPIEAGNQEAEVEEKMIKN-----------------DE
Dd    APSKMALQVIETSKVIESYIEKLPGFDKTEDQQYEDLKNLN------------------T

            370       380       390       400       410       420
Gg    ENHEAAARLEEVVYRGDMLLEKIQSALADIAQSQLKTRSGAHSQPLPDS-----------
Pt    ENHEAATCLEDVVYRGDMLLEKIQSALADIAQSQLKTRSGEVSVTQAEVQFCDHGSLQLQ
Mm    ENHEAATCLEDVVYRGDMLLEKIQSALADIAQSQLKTRSVTHSHSLPDS-----------
Cf    ENHEAATCLEDVVYRGDMLLEKIQSALADIAQSQLKTRSGTHSQSLPDS-----------
Hs    ENHEAATCLEDVVYRGDMLLEKIQSALADIAQSQLKTRSGTHSQSLPDS-----------
Rn    ENHEAATCLEDVVYRGDMLLEKIQSALADIAQSQLKTRSVTHSQSLPDS-----------
Bt    ENHEAATSLEDVVYRGDMLLEKIQSALADIAQSQLKTRSGTHSQSLPDS-----------
Dr    ENQEAAARLEEVVYRGDALLEKIQTALADIAQSQLRTRSGAPSQQTPPES----------
Su    DNEIAAKRLGEVVERGELLLHRIQEALAEIADAQLKTKSSSSLAASLSSSSGAAASASSS
Os    ELQKQLEAAELELKQVEALFNEATDHCINLKKPE--------------------------
Dm    DLEEVVQKGELLLEKMQYSLECIAQAQLDMQITLKHDLQ---------------------
At    QKQLEAAEGAVAKVAEVVVAEVAKAEVVADNPRKRKTIGANYYIPDKRQRLF--------
Af    ERELKMRELKRLRRTLENVLTAVETGLYGDRELLEKYA----------------------
Sb    ELQKQLEAAERELKQVEVLFNEATDNCINFKRLD--------------------------
Ca    ERIQKIKEKDNLLNLLESMIKEVVNGITETRI----------------------------
Cg    EKIHAIKKKDDLLKQVDDLITVFVGGIADSRRGTSLAPENVQEDNDIKQEAEVPTSSEAI
Sc    EKIEAIKKKEKLLRHVDSLIEDFVDGIANSKKST--------------------------
Sp    ERKSLESENEDLKLQLAKRIETFGRLSCVLFQP---------------------------
Nc    QRQEAVKEKDEVLVKLDQTLRSIRRY----------------------------------
Ce    KQREKVNELVGLVGDSNRLVGVVQKKLAEISKTQMSSRPSE-------------------
Dd    QSKQVSNELLSSRRDASKF-----------------------------------------

            430       440       450       460       470
Gg    -----------------------------------------------------
Pt    PGFR-------------------------------------------------
Mm    -----------------------------------------------------
Cf    -----------------------------------------------------
Hs    -----------------------------------------------------
Rn    -----------------------------------------------------
Bt    -----------------------------------------------------
Dr    -----------------------------------------------------
Su    TPSSASSSSLNVSAASSMNGTNS------------------------------
Os    -----------------------------------------------------
Dm    -----------------------------------------------------
At    -----------------------------------------------------
Af    -----------------------------------------------------
Sb    -----------------------------------------------------
Ca    -----------------------------------------------------
Cg    EQKIANEKIESKIEGEYNDNINEDSDSKSADSELFMDKDDADNISESISPGKI
Sc    -----------------------------------------------------
Sp    -----------------------------------------------------
Nc    -----------------------------------------------------
Ce    -----------------------------------------------------
Dd    -----------------------------------------------------
```

Med22

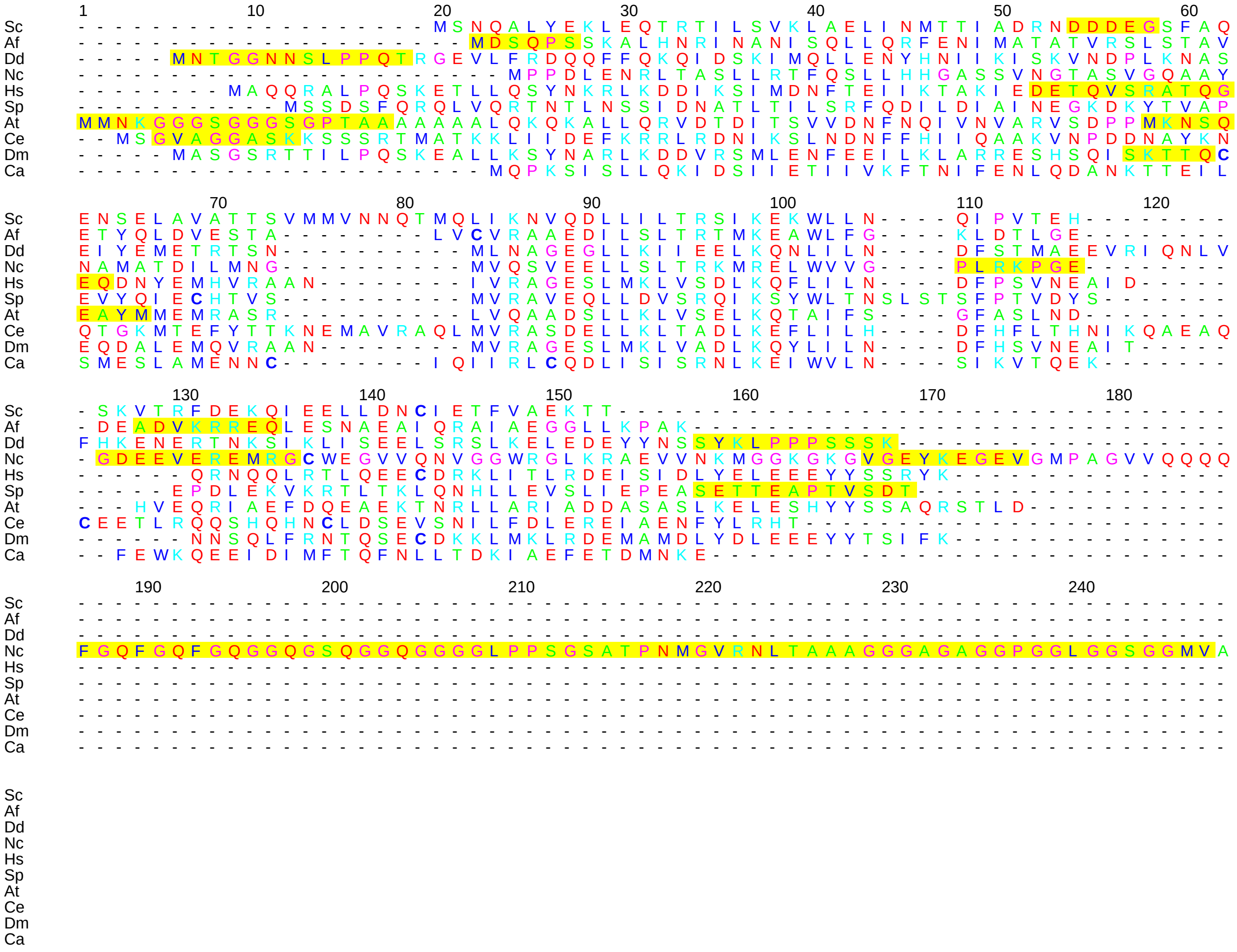

Med26

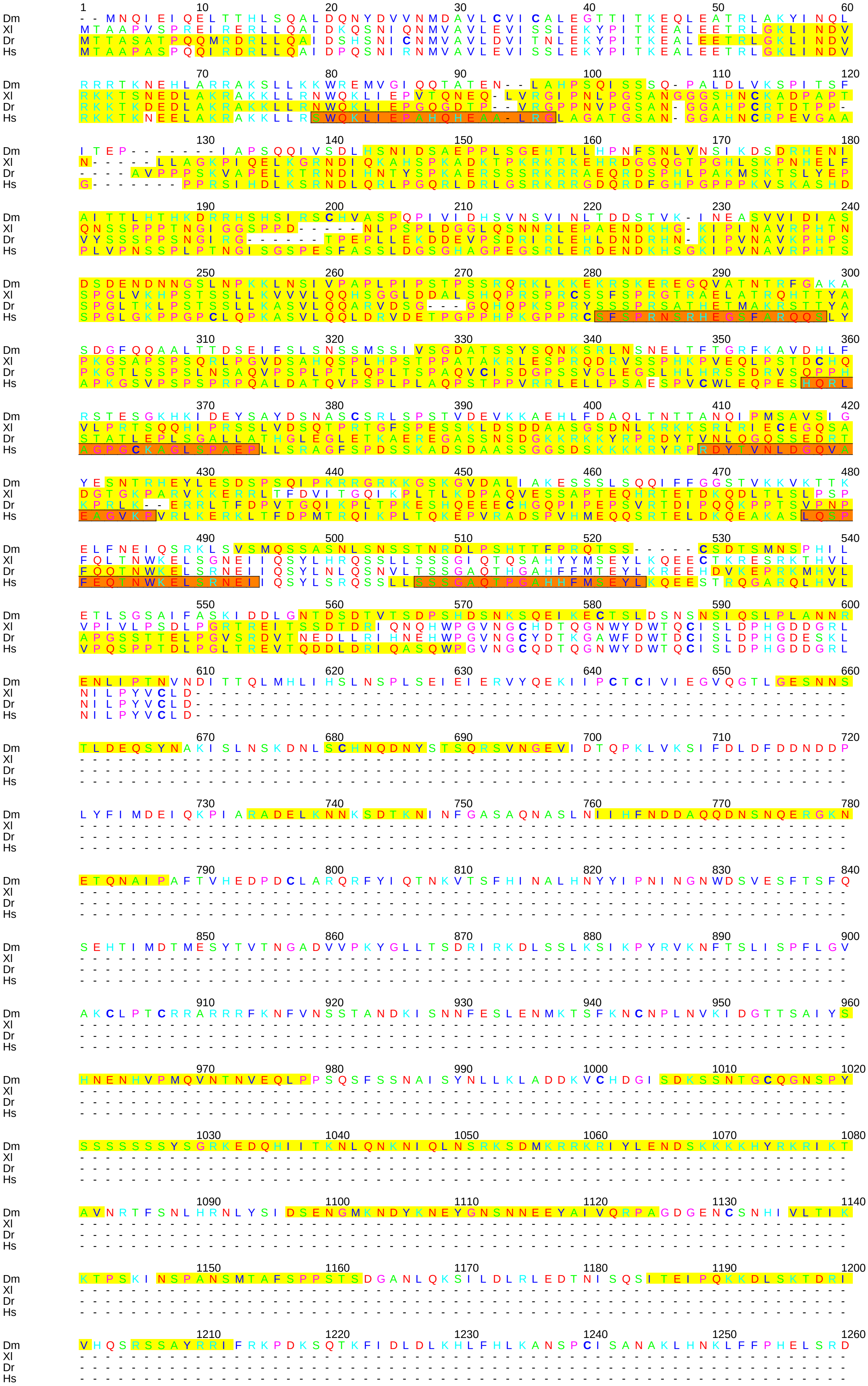

Med26

```
                                 1270                1280                1290                1300                1310                1320
Dm          N T S G I R E R L I N Y S S S S S S S Y D D D S E V E N T S F L K K A N V N K F S A S N N R N L K T E T E T C A A T A I
Xl          - - - - - - - - - - - - - - - - - - - - - - - - - - - - - - - - - - - - - - - - - - - - - - - - - - - - - - - - - - - -
Dr          - - - - - - - - - - - - - - - - - - - - - - - - - - - - - - - - - - - - - - - - - - - - - - - - - - - - - - - - - - - -
Hs          - - - - - - - - - - - - - - - - - - - - - - - - - - - - - - - - - - - - - - - - - - - - - - - - - - - - - - - - - - - -

                                 1330                1340                1350                1360                1370                1380
Dm          S E F K F E S D E D S I L T S L G D S D I D I Q D E I Q V I N D K V D Y C N D F Q N N K M L E S F N S I F V D S L R T N
Xl          - - - - - - - - - - - - - - - - - - - - - - - - - - - - - - - - - - - - - - - - - - - - - - - - - - - - - - - - - - - -
Dr          - - - - - - - - - - - - - - - - - - - - - - - - - - - - - - - - - - - - - - - - - - - - - - - - - - - - - - - - - - - -
Hs          - - - - - - - - - - - - - - - - - - - - - - - - - - - - - - - - - - - - - - - - - - - - - - - - - - - - - - - - - - - -

                                 1390                1400                1410                1420                1430                1440
Dm          T K N A S D N M G S S A H I T S L E S L K P E N A D A I T R Y C N N N N L G V N L S N E L S N I S A K P L K S F D S T M
Xl          - - - - - - - - - - - - - - - - - - - - - - - - - - - - - - - - - - - - - - - - - - - - - - - - - - - - - - - - - - - -
Dr          - - - - - - - - - - - - - - - - - - - - - - - - - - - - - - - - - - - - - - - - - - - - - - - - - - - - - - - - - - - -
Hs          - - - - - - - - - - - - - - - - - - - - - - - - - - - - - - - - - - - - - - - - - - - - - - - - - - - - - - - - - - - -

                                 1450                1460                1470                1480                1490                1500
Dm          K L P S L G I E H C T G I K T S S D M A S T N I A E K K L T R I Q Q F K E W H Q V L Q L R S Y N N E P L I V L P Y V L L
Xl          - - - - - - - - - - - - - - - - - - - - - - - - - - - - - - - - - - - - - - - - - - - - - - - - - - - - - - - - - - - -
Dr          - - - - - - - - - - - - - - - - - - - - - - - - - - - - - - - - - - - - - - - - - - - - - - - - - - - - - - - - - - - -
Hs          - - - - - - - - - - - - - - - - - - - - - - - - - - - - - - - - - - - - - - - - - - - - - - - - - - - - - - - - - - - -

Dm          E
Xl          -
Dr          -
Hs          -
```

Med28

```
       1        10        20        30        40        50        60
Rn     MAASLGGMFAGQPPGPPPPPPGLPGQASLLQAAPGAPRPSNSTLVDELESSFEACFASLVSQ
Su     -------------------------------MATHVGPSTDRHLVDELEAAFKACLSPLVAQ
Dm     ---------------------------------MASNESGGGNLMDEFEEAFQSCLLTLTKQ
Hs     MAAPLGGMFSGQPPGPPQAPPGLPGQASLLQAAPGAPRPSSSTLVDELESSFEACFASLVSQ

              70        80        90        100       110       120
Rn     DYVNGTDQEEIRTGVDQCIQKFLDIARQTECFFLQKRLQLSVQKPDQVIKEDVSELRSELQR
Su     ENVHVSDQVELKTSVDQSMQRYQELAKETENFFLQRQMLMATTKPESIIKEEIDELKAELTR
Dm     EPNSGTNKEEIDLEVQKTTNRFIDVARQMEAFFLQKRFLVSTLKPYMLIKDENQDLSIEIQR
Hs     DYVNGTDQEEIRTGVDQCIQKFLDIARQTECFFLQKRLQLSVQKPEQVIKEDVSELRNELQR

            130       140       150       160       170       180
Rn     KDALVQKHLTKLRHWQQVLEDINVQ-HKKPADMPQGSLAYLEQASANIPAPLKQT-------
Su     KNALLQKQQTKVNNWLGILQSLECGGPPQQQPHAQQQQLHHGQIQQQQQQLARGTMSIPSGN
Dm     KEALLQKHYNRLEEWKACLSDIQQGVHSRPTP-PIGSGMLQGPGGGMPPMGGTPPRPGMMPG
Hs     KDALVQKHLTKLRHWQQVLEDINVQ-HKKPADIPQGSLAYLEQASANIPAPLKPT-------

          190       200       210       220       230       240
Rn     --------------------------------------------------------
Su     IPQQQSGGQQHHGVVGAASIAGGGDATPISIATTPLSGPLAHLEQATSNIGGFDRR
Dm     MPPGAMQPGGPMQPSPHMLQAQQMQQLRMISRQMPPK-------------------
Hs     --------------------------------------------------------
```

Med30

```
                1        10        20        30        40        50        60
Gg  ------------------------------------------------------------
Pt  ------------------------------------------------------------
Dm  MWKYGQNQGNQGPSSGGGGGGGPNMMPMGGFGMQHGNMQQMHMSPQHQQQQQQMGMMGGP
Mm  ------------------------------------------------------------
Rn  --------------------------------MCLDSYSGSSHYRMKQGMKRNALALLVH
Cf  ------------------------------------------------------------
Bt  ------------------------------------------------------------
Hs  ------------------------------------------------------------
Su  ------------------------------------------------------------

                         70        80        90       100       110       120
Gg  ------------------------------------------------------------
Pt  ----------------------------MDIQLKKYVRNTFNNLLPSEAQSCSLEAMSVT
Dm  GSMQMNPQGPGGPGGLMPGMSPQHQMQQQQQQQMMQQQMMVPQQGVGVGVGMGGGVGMGG
Mm  ------------------------------------------------------------
Rn  REYHSSLRQSLYHRQSPSSRMLETSTALMTLRLEMENGNSEGHDRESASAWSIDSERLRR
Cf  ------------------------------------------------------------
Bt  ------------------------------------------------------------
Hs  ------------------------------------------------------------
Su  ------------------------------------------------------------

                        130       140       150       160       170       180
Gg  --------------------------MSTPPLAGAGMPPGAFSGTQAQAAREVNTASLCR
Pt  VTGFFTVNIPVENKTIIKKIGLTAKVTDSGEFGLLEMPVLIAEQGWMLTCYFTHQASSCH
Dm  GGVVPQQQQQQPQQNMPQQNIPQQQQQLNPVAGIPPGGAGGSNNMLAISQQNPHKEINIV
Mm  -------------------------MSTPPLAPTGMASGPFGGPQAQQAAREVNTATLCR
Rn  GRRKWRRLFEIGRVGSGRTRAAGGAMSTPPLAPTGMASGPFGGPQAQQAAREVNTATLCR
Cf  -------------------------MSTPPLAASGMAPGPFGGPQAQQAAREVNTASLCR
Bt  -------------------------MSTPPLAASGMAPGPFAGPQAQQAAREVNTASLCR
Hs  -------------------------MSTPPLAASGMAPGPFAGPQAQQAAREVNTASLCR
Su  -------------------------------------MSGVGQSLGGSSTKEVNAASLCR

                        190       200       210       220       230       240
Gg  IGQETVQDIVFRTMEIFQLLRNMQLPNGVTYHTGTYQDRLGKLQEHLRQLSILFRKLRLV
Pt  CGLHGKIYQAPEKPVIEECAFSTWLPNGVTYHTGTYQDRLTKLQDNLRQLSVLFRKLRLV
Dm  QLSRLGQETVQDIASRFQEVFASLKGIQPTSHRENSSEK--KVQEYFRTIRLLFKRVRII
Mm  IGQETVQDIVYRTMEIFQLLRNMQLPNGVTYHTGTYQDRLTKLQDHLRQLSILFRKLRLV
Rn  IGQETVQDIVYRTMEIFQLLRNMQLPNGVTYHTGTYQDRLTKLQDHLRQLSILFRKLRLV
Cf  IGQETVQDIVYRTMEIFQLLRNMQLPNGVTYHTGTYQDRLAKLQDHLRQLSILFRKLRLV
Bt  IGQETVQDIVYRTMEIFQLLRNMQLPNGVTYHTGTYQDRLAKLQDHLRQLSILFRKLRLV
Hs  IGQETVQDIVYRTMEIFQLLRNMQLPNGVTYHTGTYQDRLTKLQDNLRQLSVLFRKLRLV
Su  LGQETVQDLVTETQDMFSNLKGVQLPNGRSNTQQSHQDKITKLQDITKHIEVHFKKLRLL

                        250       260       270       280       290       300
Gg  YDKCNENCAGLDPIPIEQLIPYVEEDGSKHDDRGAASQLRFASEERR---EIMEVNKKL-
Pt  YDKCNENCGGMDPIPVEQLIPYVEEDGSKNDDRAGPP--RFASEERR---EIAEVNKVSY
Dm  YEKCND--AGMDYMSAESLIPYRDEPEPR-----IEP--SLCDEYRKVLQENHELIET--
Mm  YDKCNENCGGMDPIPVEQLIPYVDEDGSKNDDRAGPP--RFASEERR---EIVEVNKK--
Rn  YDKCNENCGGMDPIPVEQLIPYVEEDGSKNDDRAGPP--RFASEERR---EIAEVNKK--
Cf  YDKCNENCGGMDPIPVEQLIPYVEEDGSKNDDRAGPP--RFASEERR---EIAEVNKK--
Bt  YDKCNENCGGMDPIPVEQLIPYVEEDGSKDDRAGPPR---FASEERR---EIAEVNKK--
Hs  YDKCNENCGGMDPIPVEQLIPYVEEDGSKNDDRAGPP--RFASEERR---EIAEVNKK--
Su  YDKCIQISSGVHT--------------------ASGT--HTPSEQLQ-------------

                        310       320       330       340       350       360
Gg  ------KLKNQQLKQIMDQLRNLIWDINAMLAMRN-------------------------
Pt  YQALE-KEQGRMLKEVLRQSVTQAKLQEIGDESQPCLHLAASLCQLQQTVSACARLCSHH
Dm  -----VKLKNRQLREIIDRTRIIIWEINTMLAMRRS------------------------
Mm  -----LKQKNQQLKQIMDQLRNLIWDINAMLAMRN-------------------------
Rn  -----LKQKNQQLKQIMDQLRNLIWDINAMLAMRN-------------------------
Cf  -----LKQKNQQLKQIMDQLRNLIWDLNAMLAMRN-------------------------
Bt  -----LKQKNQQLKQIMDQLRNLIWDINAMLAMRN-------------------------
Hs  -----LKQKNQQLKQIMDQLRNLIWDINAMLAMRN-------------------------
Su  -------AKNRQLRQVMEQMRHLMWDINTMLATRHSTPDWK-------------------

                        370       380
Gg  ----------------------------
Pt  LPPPTGKLIMQIKAATVVQLKEPGRRNQ
Dm  ----------------------------
Mm  ----------------------------
Rn  ----------------------------
Cf  ----------------------------
Bt  ----------------------------
Hs  ----------------------------
Su  ----------------------------
```

Med31

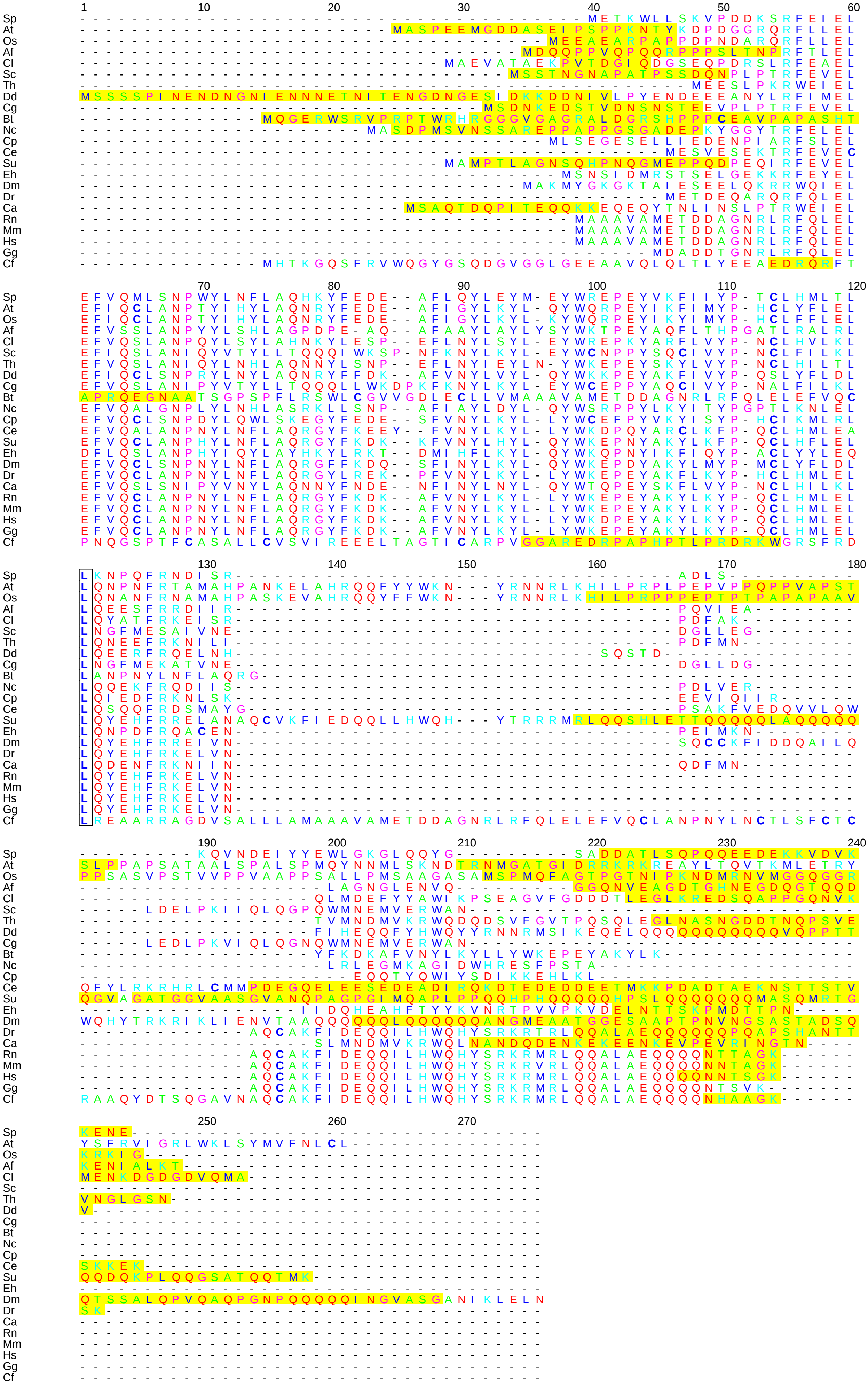
1 10 20 30 40 50 60
70 80 90 100 110 120
130 140 150 160 170 180
190 200 210 220 230 240
250 260 270
Sp
At
Os
Af
Cl
Sc
Th
Dd
Cg
Bt
Nc
Cp
Ce
Su
Eh
Dm
Dr
Ca
Rn
Mm
Hs
Gg
Cf

Cdk8

```
     1        10        20        30        40        50        60
Nc  ------------------------------------------------------------
Kl  --------------MYGNQQNNSNPYQMSYYRMNNGQGQGTNRWPQQLSHQEMLAGHSQQ
Ce  ------------------------------------------------------------
Af  ------------------------------------------------------------
Sc  -------------------------------------------MYQRPMQVQGQQQAQSF
Ca  MSYSSASFRKLNNVGISQPSQTTTTTTSANQPQSQSQQQPLQQSQQQHLHMKPNPHIPHH
Dr  ------------------------------------------------------------
Hs  ------------------------------------------------------------
Mm  ------------------------------------------------------------
At  ------------------------------------------------------------
Sp  ------------------------------------------------------------
Dm  ------------------------------------------------------------
Dd  ------------------------------------------------------------
```

```
              70        80        90       100       110       120
Nc  ------------------------------------------------------------
Kl  ILNTNKPAGNQSKPPIVMASNNVFSIGPYRQRKDSSRISVLQKYEIIGYIAAGTYGKVYK
Ce  ------------------------------------------------------------
Af  ------------------------------------------------------------
Sc  VGKKNTIGSVHGKAPMLMANNDVFTIGPYRARKDRMRVSVLEKYEVIGYIAAGTYGKVYK
Ca  QLPGTVGTRASIPQPALMASNSILTLGPFKHRKDLTRESVLSTYQIMGYIAAGTYGKVYK
Dr  ------------------------------------------------------------
Hs  ------------------------------------------------------------
Mm  ------------------------------------------------------------
At  ------------------------------------------------------------
Sp  ------------------------------------------------------------
Dm  ------------------------------------------------------------
Dd  ------------------------------------------------------------
```

```
             130       140       150       160       170       180
Nc  ------------------------------------PPSVSDQRSNAGPLNYQTKVRITD
Kl  AKARDYQNG------------MNRDNVIILDSPDSVSADSNLDINSINRSTRQQEANDNL
Ce  ------------------------------------------------------------
Af  --------------------------TPGFLQHADSLFKESLDSKRPSGSGYTSKVRVRD
Sc  AKRQINSGTNSANGSSLNGTNAKIPQFDSTQPKSSSSMDMQANTNALRRNLLKDEGVTPG
Ca  AKLKS------------------------------NKLNKTDDDSGIDGINNKDIFSESM
Dr  ----------------------------------------MGYDFKVKLTGERERVEDLF
Hs  ----------------------------------------MDYDFKVKLSSERERVEDLF
Mm  ------------------------------------------------------------
At  -------------------------------------MGDGSSSRSNSSNSTSEKPEWLQ
Sp  --------------------------------------------------------MKDG
Dm  ----------------------------------------MDYDFKMKTQIERTKVEDLF
Dd  ------------------MNTHNQSSNQNGGGSGGGGGGGGSSTFSPIVLTSTYRLDVQE
```

```
             190       200       210       220       230       240
Nc  RYKVIGFISSGTYGRVYKATGRQGQTGEFAIKKFKPDKEGEAVVN---------------
Kl  TTMDFRKPSHKRFTPPNNSNSTQIRSNSGSETNVRINSSSITNNSRKPSQIQFYAIKKFK
Ce  -----MTLMIDENFKKQLAQRRERVEDLFYFENSKEIGRGTYGLVYKAVPKKQNGQFPNK
Af  KYHIVGFISSGTYGRVYKAIGKDGRKGEYAIKKFKPDKEGE-------------------
Sc  RIRTTREDVSPHYNSQKQTLIKKPLTVFYAIKKFKTEKDG--------------------
Ca  NDLHHDNSPSIMINTTTNITINNSLPQFFAIKKFKSDNHHHHINNNN-NGGNHLSKGNNS
Dr  EYEGCKVGRGTYGHVYKAKRKDGKDDRDYALKQIEG------------------------
Hs  EYEGCKVGRGTYGHVYKAKRKDGKDDKDYALKQIEG------------------------
Mm  ------------------------------------------------------------
At  QYNLVGKIGEGTYGLVFLARTKTPPKRPIAIKKFKQSKDG--------------------
Sp  YKIIGFISSGTYGKVYKAVSSNSNDKRLFAIKKFKAESKQ--------------------
Dm  NYEGCKVGRGTYGHVYKAKWKETSDGKEYALKQIDG------------------------
Dd  KYTFSYEIGSGTYGMVYKADDKKRPNNKVAVKKFRSTKEG--------------------
```

```
             250       260       270       280       290       300
Nc  --------YTGISQSAVREMALCSELRNHANVIRLIEIILED--KCIFMVFEYAEHDLLQ
Kl  TEREGVEHYTGISQSACREMSLCRELD-NNHLTKLVEIFLEK--KSIYMVSEFAEHDLLQ
Ce  EYALKMIEGQGFSMSACREIALFRELR-HPNLICLQRVFLTN-EKKVWLLLDYAEHDLWH
Af  -----IIQYTGLSQSAIREMALCSELD-HPNVVQLAEIILED--KCIFMVFEYTEHDLLQ
Sc  ---VEQLHYTGISQSACREMALCRELH-NKHLTTLVEIFLER--KCVHMVYEYAEHDLLQ
Ca  IHQDEVLHYTGISQSAIREMSLCRELN-NKNITKLVDIILEN--KSIYMVFEFCEHDLLQ
Dr  ---------TGISMSACREIALLRELK-HPNVISLQKVFLSHADRKVWLLFDYAEHDLWH
Hs  ---------TGISMSACREIALLRELK-HPNVISLQKVFLSHADRKVWLLFDYAEHDLWH
Mm  ------------------------------------------------------------
At  ---------DGVSPTAIREIMLLREIS-HENVVKLVNVHINFADMSLYLAFDYAEYDLYE
Sp  --VSSNAQQTGVSQSAIREMMLCREIQ-HENIVSLVQVLLKD--GTISMVFEYAEHDLLQ
Dm  ---------TGLSMSACREIALWRELK-HQNVITLIRVFLSHNDRKVFLLIDYAEHDLWH
Dd  ---------EGLSLTAYREIGLLKELS-NENIVKLLDVCLNPKDKLLYLIFDYAEFDLFG
```

```
             310       320       330       340       350       360
Nc  IIHHHTQTPRHP----IAPGTIKSIMYQLLQGVQYLHTNWVLHRDLKPANIMVTSSGE--
Kl  IIHFHSHPEKR----LIPPRMLKSIMWQILDGVSYLHQNWILHRDLKPANIMVTVDGC--
Ce  VIKHHRTAKSKKVPIMVPRNMVKNILFQILSGMHYLHSNWVLHRDLKPANILLMGDGPPD
Af  IIHHHTQPQRH----AIPAPMIKSILFQLLNGLQYLHTNWVLHRDLKPANILVTSSGAVR
Sc  IIHFHSHPEKR----MIPPRMVRSIMWQLLDGVSYLHQNWVLHRDLKPANIMVTIDGC--
Ca  IIHYQSHPDFKP----IPCPTIKSLIWQILNGVTFLHKNWILHRDLKPANIMVSSQGVVK
Dr  IIKFHRASKANKKPLQLPRGMVKSLLYQILDGIHYLHANWVLHRDLKPANILVMGEGPER
Hs  IIKFHRASKANKKPVQLPRGMVKSLLYQILDGIHYLHANWVLHRDLKPANILVMGEGPER
Mm  ------------------------------------------------------------
At  IIRHHRDKVGHS----LNTYTVKSLLWQLLNGLNYLHSNWIIHRDLKPSNILVMGDAEEH
Sp  IIHFHSRSRTR----QIPPSILKSILWQIINGVAYLHENWIMHRDLKPANIMITATGK--
Dm  IIKFHRAAKATKKQVVVPRGMVKSLLYQILDGIHYLHSNWVLHRDLKPANILVMGDGNER
Dd  IIKYHRENGSHFS-----DATIKSLIWQVLNGIHYLHSNWVIHRDLKPSNILVMGEGKEC
```

```
             370       380       390       400       410       420
Nc  ----VKIGDLGLARVFKQPIHNLYMGDKVVVTIWYRAPELLLGSKHYTTAIDLWAVGCIF
Kl  ----VKIGDLGLARKFNNMVQTLYTGDKVIVTIWYRAPELILGARHYTPAIDLWAVGCIF
Ce  MRGRVKIADLGFSRIYANPLKPMAELDPVVVTFWYRAPELLLGAKHYTKAIDVWAIGCIF
Af  ------IGDLGLARLFYKPLNSLYSGDKVVVTIWYRAPELLMGSRHYTPAVDLWAVGCIF
Sc  ----VKIGDLGLARKFHNMLQTLYTGDKVVVTIWYRAPELLLGARHYTPAVDLWSVGCIF
Ca  ------IGDLGLARKFKSPLQSLYTGDKVVVTIWYRAPELLLGTRHYTPAVDLWAVGCIL
Dr  --GRVKIADMGFARLSNSPLKPLADLDPVVVTFWYRAPELLLGARHYTKAIDIWAIGCIF
Hs  --GRVKIADMGFARLFNSPLKPLADLDPVVVTFWYRAPELLLGARHYTKAIDIWAIGCIF
Mm  ------------------------------------------------------------
At  G--IVKIADFGLARIYQAPLKPLSD-NGVVVTIWYRAPELLLGSKHYTSAVDMWAVGCIF
Sp  ----VKIGDLGLGRLIRDPILPFYSSDRVVVTIWYRAPELLLGAHDYTPAIDVWAIGCIY
Dm  --GRVKIADMGFARLFNAPLKPLADLDPVVVTFWYRAPELLLGARHYTKAIDIWAIGCIF
Dd  G--TVKIGDFGLARIFQSPLKPLNE-NGVVVTIWYRSPELLLGSKHYTRAVDIWAIGCIF
```

```
             430       440       450       460       470       480
Nc  AELLSLRPIFKGEEAKMDS---KKTVPFQKHQMQKIVDVMGLPTRERWPLLASMPEYSQL
Kl  AELIGLRPIFKGEEAKMES---KKSVLFQANQFQKILEVMGSPDHKIWPNIDSYPEYLQL
Ce  AELLTAEPLFFCKEED-----IKAQNPYHYDQVKRIFHLLGYPSDADWPDMKKMPDHQRL
Af  AELLSLRPIFKGEEAKMDS---KKTVPFQRNQMMKIIDIMGLPRKETWPGLVSMPEFSQL
Sc  AELIGLQPIFKGEEAKLDS---KKTVPFQVNQLQRILEVLGTPDQKIWPYLEKYPEYDQI
Ca  AELLSLRPIFKGEEAKIDLNN-KKSVPFQKNQLQKIIEILGTPTTDIWNNLNKYPEYLSF
Dr  AELLTSEPIFHCRQED-----IKTSNPYHHDQLDRIFNVMGFPADKDWEDIKKMPEHSTL
Hs  AELLTSEPIFHCRQED-----IKTSNPYHHDQLDRIFNVMGFPADKDWEDIKKMPEHSTL
Mm  ----------------------------------------MYFTDKDWEDIKKMPEHSTL
At  AELLTLKPLFQGAEAK------SSQNPFQLDQLDKIFKILGHPTMDKWPTLVNLPHWQND
Sp  GEMLALSPLFKGDEIKMED---KKVVPFQSTQMLRIMELLGTPTEERWPGLKNYPEYYQL
Dm  AELLTSEPIFHCRQED-----IKTSNPYHHDQLDRIFNVMGFPQDKDWEDIKKMPEHHTL
Dd  AELITTKPLFPGKEKD-----PKIPSLFQDDQVEKIIRVLGKPTLDMWPDIKHLPEWKRL
```

Cdk8

```
           490        500        510        520        530        540
Nc  STLQPPLLST NTSHSHGGGH HPYGHRSQQQ PPASQNTHGN LQKWYHQTTV STPLTSLGAE
Kl  AKMP------ ---------- ---------- ---------- --------KY RDNLTAWYQT
Ce  LS-------- -------DAR NEGTPIQTFP N----SLHRY FDKWKINSQS SP--------
Af  QS-------- ---------- ---LAMSRGY INRQCNLEGW YQSCLKNNGY SP-----GSA
Sc  TKFP------ ---------- ---------- ---------- --------KY RDNLATWYHS
Ca  TQHFN----- ---------- ---------- ---------- ---QNYPNNL SNWFKLINGG
Dr  MKDFRRN--- ---------- ---------- ---------- ------TYTN CSLIKYMEKH
Hs  MKDFRRN--- ---------- ---------- ---------- ------TYTN CSLIKYMEKH
Mm  MK-------- -------DFR R-----NTYT N---CSLIKY ME----KHKV KP-----DSK
At  VQH------- ---------- ---------- ---------- -----IQAHK YDSVGLHNVV
Sp  SSFEVR---- ---------- ---------- ---------- --------YW NNLLPQWYQT
Dm  TKDFKRS--- ---------- ---------- ---------- ------TYST CSLAKYMERH
Dd  SS-------- ---------- ---------- ---------- --------ME AFPNSLAKCV

           550        560        570        580        590        600
Nc  G-------YK LLASLLEYDP EKRLTAADAL VHPFFTSPTS TPLASLNSNT ANSTSQGTG-
Kl  AGGKDKTALD ILYRLLQYDP IKRIDAIDAL DHVYFTNGDP P--------- ----------
Ce  --------YR LLVKLLTVDP TKRVSCEEAM NDIYFRKMER PPRETDDVFN KYPIPYAKKE
Af  AGTPGAEGFD LLSRLLEYDP TKRISAREAL EHPYFTTG-- ---------- ----------
Sc  AGGRDKHALS LLYHLLNYDP IKRIDAFNAL EHKYFTESDI P--------- ----------
Ca  NNQNSEKCLE LLSGLLKYDP ELRLTADQAL LHPYFLELPK VN-------- ----------
Dr  KVKPDSKAFH LLQKLLTMDP IRRITSEQAM QDPYFLEEPL P--------- ----------
Hs  KVKPDSKAFH LLQKLLTMDP IKRITSEQAM QDPYFLEDPL P--------- ----------
Mm  A-------FH LLQKLLTMDP IKRITSEQAM QDPYFLEDPL P--------- ----------
At  HLNQKSPAYD LLSKMLEYDP LKRITASQAL EHEYFRMDPL P--------- ----------
Sp  VKNRDPQGLD LLMKMLQYDP KSRITAKQAL EHVFFTSDKL WT-------- ----------
Dm  KIKPDSKAFH LLQKLLLMDP IKRITSEQAM QDQYFREEPQ P--------- ----------
Dd  GIDENSQAYD LLSKMILYDP SKRITASEAL DHPYFKELPL P--------- ----------

           610        620        630        640        650        660
Nc  ---AAAVNWS PTNCFEGLKN -------ETY PCRRVSQDDN DIGFGTGTGN QSGSRVNTAA
Kl  ---------V CENVFEGLN- -------YKY PPRRIHTNDN DITNVGNDNN QANHSQKQPM
Ce  QQMTVRASIV EYTANVPKIL LQVAPDQAQQ QHQQQQVQMQ QQPQMGQQQM MGQPQMVQPQ
Af  -------TPV TANCFAGYEG --------KY PHRRVTQDDN DIRSGSLPGT KRSGLPDDSL
Sc  ---------V SENVFEGLT- -------YKY PARRIHTNDN DIMNLGSRTK NNTQASGITA
Ca  ---------- -ENAFEGLN- -------YKY PNRKIYTDDN DIMTTAANNN NNNNNNNNNN
Dr  ---------- TSDVFAGCQ- -------IPY PKREFLTEEE -----PEDKA DKKNQQQQQG
Hs  ---------- TSDVFAGCQ- -------IPY PKREFLTEEE -----PDDKG DKKNQQQQQG
Mm  ---------- TSDVFAGCQ- -------IPY PKREFLTEEE -----PDEKG DKKTQQQQQG
At  ---------- GRNAFVASQP MEKN---VNY PTRPVDTNTD FEG----TTS INPPQAVAAG
Sp  ---------- ---------- -------TRY VFFLFLFIIE RNKLLLTHFL AHF-------
Dm  ---------- TQDVFAGCP- -------IPY PKREFLTDDD QEDKSDNKRQ QQQQQQQQQQ
Dd  ---------- LPNAFSKP-- -------IPY PPRLPINKKK REFDD----- ----------

           670        680        690        700        710        720
Nc  AGTGTGLVMG QAQSQVQAMG GSLGNIGGHG HGHGHGQMGG GGMGQGMGGL GQGHGGMAQM
Kl  HGNNNNKNGN MNGLGVNKRI LAAAAAAAAA AAVSGNGNNP TSNTATGGSA RKKRK-----
Ce  MGQPPMGGAH PGVVAPDGHP HQMMQQQQHP QQHHVSFLNI QNEKLTFSVQ MQYQGMHDPM
Af  MGRAAKRLKE ---------- ---------- ---------- ---------- ----------
Sc  GAAANALGGL GVNRRILAAA AAAAAVSGNN ASDEPSRKKN RR-------- ----------
Ca  NNNNNNNNNN NNNNSGHQLS QQQNVQIQQV HQMQQQIHSQ QLQSHGANST YKRSGIDDLP
Dr  NNHTNGAGHT GNPDNSHAQG PPLKKVRVVP PTATSSGLIM TSDYQRSNPH AAYQNPGPST
Hs  NNHTNGTGHP GNQDSSHTQG PPLKKVRVVP PTTTSGGLIM TSDYQRSNPH AAYPNPGPST
Mm  NNHTNGTGHP GNQDSGHAQG PPLKKVRVVP PTTTSGGLIM TSDYQRSNPH AAYPNPGPST
At  NVAGNMAGAH GMGSRSMPRP MVAHNMQRMQ QSQGMMAYNF PAQAGLNPSV PLQQQRGMAQ
Sp  ---------- ---------- ---------- ---------- ---------- ----------
Dm  QQQQQQQQQQ QQQQQQQMNA EPNAKRVRLS GAGNQQDFHH QQQQQQQQQQ QQQQQQQQMM
Dd  ---------- ---------- ---------- ---------- ---------- ----------

           730        740        750        760        770        780
Nc  AGMKRMHDAA AAAAAAAAAA VQQSHPNKRM KGSQG----- ---------- ----------
Kl  ---------- ---------- ---------- ---------- ---------- ----------
Ce  QGGMDEGPQA KMMRMGNVPV GRYAPMPPPY GAPQDYHPQQ GPPMVQMMQQ PGPSGYYPQR
Af  ---------- ---------- ---------- ---------- ---------- ----------
Sc  ---------- ---------- ---------- ---------- ---------- ----------
Ca  GGIRKKRG-- ---------- ---------- ---------- ---------- ----------
Dr  SLPQSSMGYS --STSQQPPQ YSHQTHRY-- ---------- ---------- ----------
Hs  SQPQSSMGYS ATSQQPPQYS HQTHRY---- ---------- ---------- ----------
Mm  SQ-PQSSMGY SATSQQ-PPQ YSHQTHRY-- ---------- ---------- ----------
At  PHQQQQLRRK DPGMGMSGYA PPNKSRRL-- ---------- ---------- ----------
Sp  ---------- ---------- ---------- ---------- ---------- ----------
Dm  FNQQQNFQRF N--------- ---------- ---------- ---------- ----------
Dd  ---------- ---------- ---------- ---------- ---------- ----------

           790        800        810        820        830        840
Nc  ---------- ---------- ---------- ---------- ---------- ----------
Kl  ---------- ---------- ---------- ---------- ---------- ----------
Ce  PGQPTGAVPG PGPQGYMNPQ MGMQMGMRAP GVPPQGYMPG RGMAPPQMGQ QQPGPNQQQQ
Af  ---------- ---------- ---------- ---------- ---------- ----------
Sc  ---------- ---------- ---------- ---------- ---------- ----------
Ca  ---------- ---------- ---------- ---------- ---------- ----------
Dr  ---------- ---------- ---------- ---------- ---------- ----------
Hs  ---------- ---------- ---------- ---------- ---------- ----------
Mm  ---------- ---------- ---------- ---------- ---------- ----------
At  ---------- ---------- ---------- ---------- ---------- ----------
Sp  ---------- ---------- ---------- ---------- ---------- ----------
Dm  ---------- ---------- ---------- ---------- ---------- ----------
Dd  ---------- ---------- ---------- ---------- ---------- ----------

Nc  ---------
Kl  ---------
Ce  QQWQQQYHR
Af  ---------
Sc  ---------
Ca  ---------
Dr  ---------
Hs  ---------
Mm  ---------
At  ---------
Sp  ---------
Dm  ---------
Dd  ---------
```

CycC

```
    1        10        20        30        40        50        60
Ca  MSADYWNSSQRNQWQLTRFSLLEARRRVLLLERKMIQNGLIKD-----------------YP
Ce  ------------------------------MALNFWKSSHCQQWIFDKTEIWKQRAEDMKIY
Af  ---------------------------------MAANYWASTQR------------------
Nc  -----------------MAANYWESTQRKHWLFTKDELAAMRAKLEAEEPNLVASFPLPQLR
Sc  MSGSFWTSTQRHHWQYTKASLAKERQKLWLLECQLFPQGLNIVMDSKQNGIEQSITKNIPIT
Hs  ------------------------------MAGNFWQSSHYLQWILDKQDLLKERQKDLKFL
Dd  ------------------------------MAANFWESSHCTEWLLDRNKIEESNPKDKQYL
Sp  ------------------------------MAANYWASSQLTQLFLS-------------TD
Dm  ------------------------------MAGNFWQSSHSQQWILDKPDLLRERQHDLLAL
At  ------------------------------MASNFWTSTHYKELKDPEEVNVVHPLDAQRGI

           70        80        90        100       110       120
Ca  NIIYDYNMR----IYLHNLLIKL---GRR------LNIRQIALATAEIYLTRFLTR-VSLKE
Ce  NEEEYNRLN----IFWANFITAVATEGAHSQANVGCKLRQQVIATAIIYFKRFYLR-QSFRD
Af  ----RHWLFTREKLAEIREIFQLIKLGKR------MSTRQQALATAQVYIKRFYTK-NEIRH
Nc  HLNIYFNQQSPTYFCFTLEINRL---GKR------MGLRQQALATAQVYIKRFYTK-VEIRR
Sc  HRDLHYDKDYNLRIYCYFLIMKL---GRR------LNIRQYALATAHIYLSRFLIK-ASVRE
Hs  SEEEYWKLQ----IFFTNVIQAL---GEH------LKLRQQVIATATVYFKRFYAR-YSLKS
Dd  TPIELKKLR----THYCFVIQNLG---------NALKLRQRAISTAIVYFKRFYLK-NSFVD
Sp  LESLEPTCLSKDTIYQWKVVQTFG---DR------LRLRQRVLATAIVLLRRYMLKKNEEKG
Dm  NEDEYQKVF----IFFANVIQVLG---------EQLKLRQQVIATATVYFKRFYAR-NSLKN
At  SVEDFRLIK----LHMSNYISKLAQ---------HIKIRQRVVATAVTYMRRVYTR-KSLTE

         130       140       150       160       170       180
Ca  INVYLLITTCIYVACKIEE--CPQHIRLILSEARNI-------WPEYIP--HDVTKLAEFEF
Ce  MCPFLVASTALFLACKVEEHTTLSVSSFLKNTAIVLPKRWGVTFETTST---KNGVVYDSEF
Af  TNPYLVLTTAFYLACKMEE--CPQHIRFVVGEARSL-------WPEFIT--PDVSKLGECEF
Nc  TNPHHVLVTALYLACKMEE--CPQHIRLMANEARGF-------WPTDFQSQTEVARIGECEF
Sc  INLYMLVTTCVYLACKVEE--CPQYIRTLVSEARTL-------WPEFIP--PDPTKVTEFEF
Hs  IDPVLMAPTCVFLASKVEEFGVVSNTRLIAAATSVLKTRFSYAFPKEFP--YRMNHILECEF
Dd  CEPRLVAVTCLYLSSKVEE--CITQAKKCAAKMKEIDHSFN----------YLMNDILECEF
Sp  FSLEALVATCIYLSCKVEE--CPVHIRTICNEANDL-------WSLKVK--LSRSNISEIEF
Dm  IDPLLLAPTCILLASKVEEFGVISNSRLISICQSAIKTKFSYAYAQEFP--YRTNHILECEF
At  YEPRLVAPTCLYLACKAEESVVHAKLLVFYMKKLYADEKFR----------YEIKDILEMEM

       190       200       210       220       230       240
Ca  YLIEEMDLYLLLHHPYKS---------------------------LMQINEFLS-------N
Ce  ILVEILDCCLVVHHASRPMCPPPPPREIFDKNWNSGIFLIFFLNFLVKKMENCG---IFSEK
Af  SLISEMNSQLIVHHPYRT---------------------------LSELQPELS--LTSD--
Nc  YLISEMSSHLIVHSPYRT---------------------------LTSLQGELG-------L
Sc  YLLEELESYLIVHHPYQS---------------------------LKQIVQVLK--------
Hs  YLLELMDCCLIVYHPYRP---------------------------LLQYVQDMG-------Q
Dd  FVLEELDFCLIIYHPYKS---------------------------LPFYLQSSG-------L
Sp  EIISVLDAFLIVHHPYTS---------------------------LEQAFHDGIIN------
Dm  YLLENLDCCLIVYQPYRP---------------------------LLQLVQDMG-------Q
At  KVLEALNFYLVVFHPYRS---------------------------LPEFLQDSG-------I

     250       260       270       280       290       300       310
Ca  NYNVFGFKLTV--EELQN-------AWSLINDSYITDLHLLLPPHTIAVAAIYITVVLKKNL
Ce  LEN----------FEFFNLEAIEAQCQKVANDSLRCDVSLIFPPHVIGLSSIMVAMELMG--
Af  ---------EVAL------------AWSVINDHYLTDLPLLYAPHVIAVMAIIVAVVFK---
Nc  AQEDVN------------------LAWSVINDHYMTDLPLLHPPHVIALTAILLALVLRQDP
Sc  ----------QPPFQITLSSDDLQNCWSLINDSYINDVHLLYPPHIIAVACLFITISIHG--
Hs  EDMLLP------------------LAWRIVNDTYRTDLCLLYPPFMIALACLHVACVVQ---
Dd  DPAS------------------IEIIWGIVNDSYRTDVCLLYPPFVVGLGCILLGSYLLK--
Sp  -------------------QKQLEFAWSIVNDSYASSLCLMAHPHQLAYAALLISCCNDE--
Dm  EDQLLT------------------LSWRIVNDSLRTDVCLLYPPYQIAIACLQIACVILQK-
At  NDTSMT-----------------HLTWGLVNDTYRMDLILIHPPFLITLACIYIASVHKEK-

             320       330       340       350       360       370
Ca  SRVRQGNNNDNN----ININTMHISTGSSTNPININNNNNTNTSNNNGTTSTTTTTTAQETQ
Ce  ------------------------------------RGEEL---------------------
Af  --------------------------------------PNSGNFHGSAAPVLAGAMRDGGMN
Nc  SGRLPGTAASASGLVAASAALAQAQAQAQARAAMMAGGGQTVPGLTPQSSSGLQAMLPPQSP
Sc  ---------------------------------------KPTKGSSLASAASEAIRDPKNSS
Hs  -------------------------------------QKDAR--------------------
Dd  --------------------------------------------------------------
Sp  --------------------------------------------------------------
Dm  --------------------------------------------------------------
At  --------------------------------------------------------------

           380       390       400       410       420       430
Ca  VLGQDDNTEMNIDDLMNLTKSSNNSQDKSDDKMDIDNPLQLQVNLSQIQNQTQHQHQESTHN
Ce  --------------------EAWLVEV-DTDFEKVTDCVEQIYKMY--TLWKSFDEKEEVKK
Af  VLAALGDRTGSGPPLKIQKLINWLAES-EVDIKGVIECTQELVSLY--EVWEQYSEKTCKEL
Nc  AGEGPAEGNKNPRMAKVHRFAAWLSDS-NIDIEAMVDCTQELISFY--ECHEQYNDKNTREQ
Sc  SPVQIAFN-------------RFMAES-LVDLEEVMDTIQEQITLY--DHWDKYHEQWIKFL
Hs  ---------------------QWFAEL-SVDMEKILEIIRVILKLY--EQWKNFDERKEMAT
Dd  -----------------KDIKQWLSEL-NVEMKDIWEVSKDLIDYYEFEKQQSLQNQSELDL
Sp  -----------------NTIPKLLDLIKSTDAFKVILCVQRIISIYYFEDIE----------
Dm  -----------------DATKQWFAEL-NVDLDKVQEIVRAIVNLY--ELWKDWKEKDEIQM
At  ------------------DIKTWFEEL-SVDMNIVKNIAMEILDFY--ENHRLFTEERVHAA

         440       450       460       470       480       490
Ca  NTSSTNTGRNGINGQISQSNQELNNFDLDILDEDTIKINKFMNFLEHSHINLDEVVEAVQDM
Ce  LMAKLPKPNQQPPPQQQHQHQQGYHL------------------------------------
Af  LGRMVKAKNLDK--------------------------------------------------
Nc  INRFVKARGLDK--------------------------------------------------
Sc  LHTLYLRPASAI--------------------------------------------------
Hs  ----ILSKMPKPKPPPNSEGEQGPNGSQNSSYSQS---------------------------
Dd  LYNKLPIRNKK---------------------------------------------------
Sp  --------------------------------------------------------------
Dm  LLSKIPKPKPPPQR------------------------------------------------
At  FNKLATNP------------------------------------------------------

       500       510       520
Ca  MNMYVLWNRYNEQGVKKALQVMLLNRI
Ce  ---------------------------
Af  ---------------------------
Nc  ---------------------------
Sc  ---------------------------
Hs  ---------------------------
Dd  ---------------------------
Sp  ---------------------------
Dm  ---------------------------
At  ---------------------------
```
