# Supplementary material for: Malleable Machines in Transcription Regulation: The Mediator Complex
Source: PLoS Comput Biol. 2008 Dec 19;4(12):e1000243. doi: 10.1371/journal.pcbi.1000243 (PMC2588115; doi:10.1371/journal.pcbi.1000243)
Supplement: Table S2 — α-Helical molecular recognition features (MoRFs) predicted in the Mediator complex in Homo sapiens (0.10 MB DOC) [file pcbi.1000243.s008.doc]

**Table S2 α**- MoRFs predicted in human Mediator

| **Mediator subunit** | **MoRF_start** | **MoRF_end** | **Reference** |
| --- | --- | --- | --- |
| **MED1** | 815 | 832 |  |
|  | 1377 | 1394 |  |
|  | 644 | 661 | [68] |
|  | 952 | 969 |  |
|  | 20 | 37 | [67] |
|  | 1399 | 1416 |  |
|  | 714 | 731 | [68] |
|  | 1305 | 1322 |  |
|  | 567 | 584 | [68] |
|  | 1550 | 1567 |  |
|  | 766 | 783 | [68] |
|  | 1337 | 1354 |  |
|  | 592 | 609 |  |
| **MED3** | 1116 | 1133 |  |
|  | 661 | 678 |  |
|  | 2 | 19 |  |
|  | 962 | 979 |  |
|  | 292 | 309 |  |
|  | 738 | 755 |  |
|  | 80 | 97 |  |
|  | 987 | 1004 |  |
|  | 528 | 545 |  |
|  | 796 | 813 |  |
|  | 176 | 193 |  |
|  | 1091 | 1108 |  |
|  | 594 | 611 |  |
|  | 906 | 923 |  |
|  | 260 | 277 |  |
| **MED4** | 77 | 94 |  |
|  | 205 | 222 |  |
|  | 225 | 242 |  |
| **MED6** | 212 | 229 |  |
| **MED7** | 205 | 222 |  |
|  | 33 | 50 |  |
|  | 157 | 174 |  |
| **MED8** | 132 | 149 |  |
|  | 174 | 191 |  |
|  | 251 | 268 |  |
| **MED9** | 156 | 173 |  |
|  | 485 | 502 |  |
|  | 191 | 208 |  |
|  | 536 | 553 |  |
|  | 222 | 239 |  |
|  | 334 | 351 |  |
| **MED11** | 158 | 175 |  |
| **MED12** | 1402 | 1419 |  |
|  | 2041 | 2058 |  |
|  | 84 | 101 |  |
|  | 1462 | 1479 |  |
|  | 2173 | 2190 |  |
|  | 714 | 731 |  |
|  | 1640 | 1657 |  |
|  | 749 | 766 |  |
|  | 1751 | 1768 |  |
| **MED13** | 825 | 842 |  |
|  | 695 | 712 |  |
|  | 960 | 977 |  |
|  | 724 | 741 |  |
|  | 1551 | 1568 |  |
|  | 489 | 506 |  |
|  | 772 | 789 |  |
|  | 544 | 561 |  |
| **MED14** | 1 | 18 |  |
| **MED15** | 155 | 172 |  |
|  | 190 | 207 |  |
|  | 221 | 238 |  |
|  | 335 | 352 |  |
|  | 388 | 405 |  |
|  | 526 | 543 |  |
|  | 577 | 594 |  |
| **MED17** | 220 | 237 |  |
| **MED19** | 141 | 158 |  |
|  | 1 | 18 |  |
|  | 54 | 71 |  |
| **MED23** | 1351 | 1368 |  |
| **MED24** | 840 | 857 |  |
| **MED26** | 348 | 365 |  |
|  | 400 | 417 |  |
|  | 79 | 96 |  |
|  | 468 | 485 |  |
|  | 272 | 289 |  |
|  | 498 | 515 |  |
| **MED30** | 158 | 175 |  |
| **Cdk8** | 343 | 360 |  |
|  | 447 | 464 |  |
