# Supplementary material for: Malleable Machines in Transcription Regulation: The Mediator Complex
Source: PLoS Comput Biol. 2008 Dec 19;4(12):e1000243. doi: 10.1371/journal.pcbi.1000243 (PMC2588115; doi:10.1371/journal.pcbi.1000243)
Supplement: Table S3 — Conservation scores computed on Homo sapiens and Saccharomyces cerevisiae sequences aligned by the reference [27] and also by the present iterative alignment scheme. Scores were obtained using groups of similar amino acid residues: R/K/H, A/S/T, I/L/V/M/C/F/Y/W, G/P and E/D/N/Q. (0.03 MB DOC) [file pcbi.1000243.s009.doc]

**Table S3**

|  | **Present** | **REF1** |
| --- | --- | --- |
| **Med4** | 37 | 33 |
| **Med6** | 37 | 34 |
| **Med7** | 51 | 40 |
| **Med8** | 31 | 31 |
| **Med9** | 34 | 33 |
| **Med10** | 55 | 39 |
| **Med11** | 44 | 25 |
| **Med17** | 32 | 17 |
| **Med18** | 51 | 22 |
| **Med20** | 38 | 30 |
| **Med21** | 32 | 44 |
| **Med22** | 38 | 35 |
| **Med31** | 58 | 50 |
