# Supplementary material for: Malleable Machines in Transcription Regulation: The Mediator Complex
Source: PLoS Comput Biol. 2008 Dec 19;4(12):e1000243. doi: 10.1371/journal.pcbi.1000243 (PMC2588115; doi:10.1371/journal.pcbi.1000243)
Supplement: Table S4 — Conservation scores computed on full sequences aligned by the reference [21] and the present iterative algorithm using the same sequences. AAcons was obtained using individual amino acid residues. For consistency, sequences only from those organisms were used that were found to be homologous by the present algorithm. (0.04 MB DOC) [file pcbi.1000243.s010.doc]

**Table S4**

|  | **Organism** | **AAcons** | |
| --- | --- | --- | --- |
|  |  | Present | REF2 |
| **Med1** | *Af, Ca, Hs, Nc, Sc, Sp* | 16 | 15 |
| **Med4** | *Af, At, Ca, Ce, Dd, Dm, Hs, Nc, Sc, Sp* | 14 | 15 |
| **Med6** | *Af, At, Ca, Ce, Dd, Dm, Hs, Nc, Sc, Sp* | 21 | 23 |
| **Med7** | *Af, At, Ca, Ce, Dd, Dm, Hs, Nc, Sc, Sp* | 14 | 18 |
| **Med8** | *Af, At, Ca, Ce, Dd, Dm, Hs, Nc, Sc, Sp* | 10 | 11 |
| **Med9** | *Ce, Dm, Hs* | 26 | 25 |
| **Med10** | *Af, At, Ca, Ce, Dd, Dm, Hs, Nc, Sc, Sp* | 20 | 20 |
| **Med11** | *At, Dm, Hs* | 27 | 26 |
| **Med13** | *Af, At, Ca, Ce, Dd, Dm, Hs, Nc, Sc, Sp* | 12 | 12 |
| **Med15** | *At, Ce, Dd, Dm, Hs* | 19 | 20 |
| **Med16** | *Af, Ca, Dm, Hs, Nc, Sc* | 19 | 18 |
| **Med17** | *Af, Ca, Ce, Dm, Hs, Nc, Sc, Sp* | 15 | 17 |
| **Med18** | *Af, At, Ca, Ce, Dm, Hs, Sc, Sp* | 18 | 19 |
| **Med19** | *Af, Ca, Nc, Sc, Sp* | 15 | 19 |
| **Med20** | *Af, At, Ca, Ce, Dd, Dm, Hs, Sc, Sp* | 15 | 16 |
| **Med22** | *Af, At, Ca, Ce, Dd, Dm, Hs, Nc, Sc, Sp* | 22 | 22 |
| **Med31** | *Af, At, Ca, Ce, Dd, Dm, Hs, Nc, Sc, Sp* | 22 | 21 |

**Af:** *Argia fumigata*, **At**: *Arabidopsis thaliana*, **Ca:** *Candida albicans*, **Ce:** *Caenorhabditis elegans*, **Dd:** *Dictyostelium discoideum*, **Dm:** *Drosophila melanogaster*, **Dr:** *Danio rero*, **Hs:** *Homo sapiens*, **Nc:** *Neurospora crassa*, **Sc:** *Saccharomyces cerevisiae*, **Sp:** *Saccharomyces pombe*
